# Analysis of High Molecular Mass Compounds from the Spider *Pamphobeteus verdolaga* Venom Gland. A Transcriptomic and MS ID Approach

**DOI:** 10.3390/toxins13070453

**Published:** 2021-06-29

**Authors:** Sebastian Estrada-Gómez, Leidy Johana Vargas-Muñoz, Cesar Segura Latorre, Monica Maria Saldarriaga-Cordoba, Claudia Marcela Arenas-Gómez

**Affiliations:** 1 Grupo de Toxinología—Serpentario, Facultad de Ciencias Farmacéuticas y Alimentarias, Universidad de Antioquia UdeA, Carrera 53 No. 61-30, Medellín 050010, Colombia; cesar.segura@udea.edu.co; 2 Facultad de Ciencias Farmacéuticas y Alimentarias, Universidad de Antioquia UdeA, Calle 70 No. 52-21, Medellín 050010, Colombia; 3 Facultad de Medicina, Universidad Cooperativa de Colombia, Calle 50 A No. 41-20, Medellín 050012, Colombia; leidy.vargasmu@campusucc.edu.co; 4 Unidad de Espectrometría de Masas, Sede de Investigación Universitaria, Universidad de Antioquia UdeA, Carrera 53 No. 61-30, Medellín 050010, Colombia; 5 Centro de Investigación en Recursos Naturales y Sustentabilidad, Universidad Bernardo O’Higgins, Avenida Viel 1497, Santiago 7750000, Chile; msaldarriaga@docente.ubo.cl; 6 Grupo de Génetica, Regeneración y Cáncer, Universidad de Antioquia UdeA, Carrera 53 No. 61-30, Medellín 050010, Colombia; claudia.arenas@udea.edu.co

**Keywords:** Theraphosidae, *Pamphobeteus*, transcriptomic, high-molecular-mass compounds, phospholipases, kunitz-type, hyaluronidases, lycotoxins

## Abstract

Nowadays, spider venom research focuses on the neurotoxic activity of small peptides. In this study, we investigated high-molecular-mass compounds that have either enzymatic activity or housekeeping functions present in either the venom gland or venom of *Pamphobeteus verdolaga*. We used proteomic and transcriptomic-assisted approaches to recognize the proteins sequences related to high-molecular-mass compounds present in either venom gland or venom. We report the amino acid sequences (partial or complete) of 45 high-molecular-mass compounds detected by transcriptomics showing similarity to other proteins with either enzymatic activity (i.e., phospholipases A_2_, kunitz-type, hyaluronidases, and sphingomyelinase D) or housekeeping functions involved in the signaling process, glucanotransferase function, and beta-N-acetylglucosaminidase activity. MS/MS analysis showed fragments exhibiting a resemblance similarity with different sequences detected by transcriptomics corresponding to sphingomyelinase D, hyaluronidase, lycotoxins, cysteine-rich secretory proteins, and kunitz-type serine protease inhibitors, among others. Additionally, we report a probably new protein sequence corresponding to the lycotoxin family detected by transcriptomics. The phylogeny analysis suggested that *P. verdolaga* includes a basal protein that underwent a duplication event that gave origin to the lycotoxin proteins reported for *Lycosa sp.* This approach allows proposing an evolutionary relationship of high-molecular-mass proteins among *P. verdolaga* and other spider species.

## 1. Introduction

Spider venom is a complex mixture of pharmacologically active peptides, proteins, and inorganic compounds. Most spider venom toxins can be classified into one of three groups, depending on their molecular mass i.e., low (<1 kDa), medium (1–10 kDa), and high (>10 kDa) [1]. To date, the vast majority of transcriptomic and proteomic analyses have focused on medium-molecular-mass peptides, since these compounds are the main ones responsible for the toxic effects produced by spider venoms (except in the Theridiidae family).

High-molecular-mass compounds (HMMC) are more commonly distributed among spider venoms than is described in the literature [1]. Most of the HMMC reported correspond to proteins with housekeeping activity, and venom proteins with enzymatic activities have been described in the Theraphosidae species *Ornithoctonus huwena*, *Grammostola iheringi*, *Chilobrachys jingzhao*, *Haplopelma hainanum,* and *Haplopelma schmidti* [2,3,4,5,6]. The transcriptomic analysis of these species´ venom glands showed different sequences clustered as kunitz-type toxins (KTTs), phospholipases, and zinc metalloproteinases, among others. The primary role of these compounds is the degradation of the extracellular matrix (collagenase, hyaluronidase, and proteases) and the underlying cell membrane [1].

*Pamphobeteus verdolaga* (Cifuentes, Perafán, and Estrada-Gomez 2016) is a recently described species endemic to Colombia that is native to the Andean region from the Aburrá Valley (Medellín) to the southwest region of Antioquia in the municipality of Jardin located at 2100 m above sea level (m.a.s.l) [7]. Taxonomically, the male *P. verdolaga* has a palpal bulb with broad embolus, poorly developed apical keel, prolateral inferior keel and prolateral accessory keel present but poorly developed, and retrolateral keel of similar length as the apical keel. Females are distinguished by the morphology of spermatheca with a wide base and very short oval seminal receptacles, which are curved toward the center [7]. A previous *P. verdolaga* venom report suggests the presence of peptides such as EF-hand proteins, jingzhaotoxins, theraphotoxins, hexatoxins, and inhibitory cysteine knots (ICK) peptides and uncommon proteins in the Theraphosidae family such as sphingomyelinases (sicaritoxin), barytoxins, hexatoxins, latroinsectotoxins, and linear (zadotoxins) peptides [8,9]. The last analysis of *P. verdolaga* venom indicated that this tarantula is an important source of active disulfide-rich peptides that potentially modulate voltage-gated calcium and sodium channels [10].

In this study, we conducted a more detailed transcriptomic and proteomic analysis focused on the description of HMMC in both the venom gland and venom itself. Our main goal is to describe the HMMC (with enzymatic activity or housekeeping functions) encoded by the venom gland-cells and to confirm their presence in the venom (secretion).

## 2. Results

### 2.1. Transcriptomic Findings

In the venom gland transcriptome of *P. verdolaga,* we identified 45 transcripts (with e-values > 1 × 10^−5^ and scores >50) coding for the putative protein ORFs previously reported, corresponding to phospholipases A_2_, phospholipases D, phospholipases B, kunitz-type serine protease inhibitors (KTSPI), hyaluronidases, lycotoxins toxins, CRISP proteins, hephaestin-like protein and venom metalloproteinase (see Table 1A–I, respectively). All translated sequences correspond to partial or full-length sequences, as shown in Section S1 on Supplementary File S1. Transcriptome abundance can be found on Supplementary File S2.

**Figure 1:**
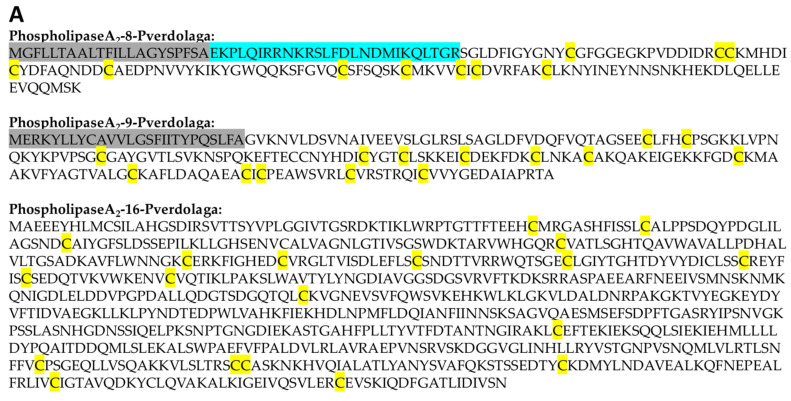

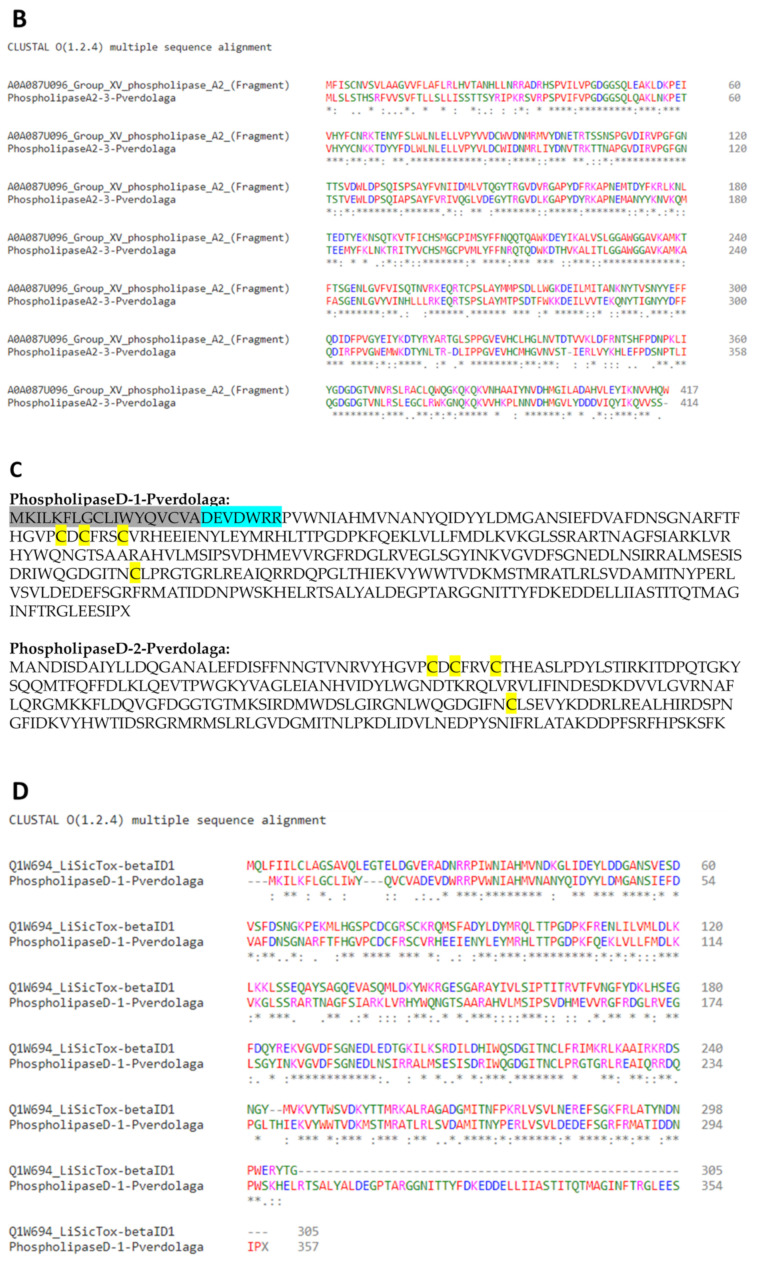
*Pamphobeteus verdolaga* phospholipases sequence. (**A**) PLA_2_ complete sequences of contigs c12950, c18752, and c60448. For all sequences, residues highlighted in grey indicate the signal peptide, while magenta highlighted residues indicate the propeptide according to Spider|ProHMM, from the arachnoserver. Residues highlighted in yellow show cysteines potentially forming disulfide bridges. (**B**) Pairwise sequence alignment of mature phospholipaseA2-3-pverdolaga (c15998) from *P. verdolaga* with a phospholipase A_2_ from *Stegodyphus mimosarum* (UniProtKB—A0A087U096). For all alignments, * (asterisk) indicates positions which have a single, fully conserved residue; : (colon) indicate conservation between groups of strongly similar properties, scoring >0.5; and . (period) indicates conservation between groups of weakly similar properties, scoring ≤0.5. (**C**) PLD complete sequences of contigs c46024 and c14372. (**D**) Pairwise sequence alignment of phospholipaseD-1-pverdolaga (c46024) from *P. verdolaga* with a phospholipase D from *Loxosceles intermedia* (UniProtKB—Q1W694).

Sixteen contigs shared a significant resemblance with phospholipase A_2_ from Groups II, XV, and XIIA from various organisms, some of them corresponding to fragments (see Table 1A and Section S1 on Supplementary File S1). Sequences phospholipaseA_2_-8-pverdolaga, phospholipaseA_2_-9-pverdolaga, and phospholipaseA_2_-16-pverdolaga fulfill the parameters to be considered complete PLA_2_ sequences indicating possible complete sequences (see Figure 1A and Section S1 on Supplementary File S1). PhospholipaseA_2_-9-pverdolaga showed 14 Cys and was annotated by hmmr and cdd domain (NCBI) (Supplementary Files S1 and S3). Although most of the *P. verdolaga* phospholipases A_2_ matched phospholipases reported in the spider *Stegodyphus mimosarum*, six sequences matched phospoholipases from three different ants (*Lasius niger*, *Harpegnathos saltator,* and *Cam0ponotus floridanus*), one lizard (*Anolis carolinensis*), and one tick (*Ixodes scapularis*). Phospholipases matching the spider *S. mimosarum* showed a high degree of similarity, particularly in the sequence alignment of phospholipaseA2-3-pverdolaga (c15998) that matched a Group XV phospholipase A_2_ from *S. mimosarum* (UniProtKB—A0A087U096) with a >80% similarity (see Figure 1B). The phylogenetic analysis suggests multiple duplication events that led to the huge diversification of the phospholipase A_2_ in *P. verdolaga* and supports (branch support >50%) the high similarity of these sequences with proteins reported for different spider families, e.g., Theridiidae, Araneidae, and Sicariidae (Figure S1 on Supplementary File S1).

In addition, the analysis revealed the presence of four different contigs matching phospholipase D proteins (PLDs) reported in other spiders (*Loxosceles* sp., *Sicarius* sp., and *Stegodyphus* sp.) and mites (*Metaseiulus occidentalis*) (see Table 1B). At least two sequences, phospholipaseD-1-pverdolaga and phospholipaseD-2-pverdolaga, may correspond to the complete sequence conserving the cysteine residues necessary to form disulfide bridges characteristic in PLD isolated from *Loxoceles* (see Table 1B, Figure 1C,D, and Section S1 on Supplementary File S1). One of the MS/MS fragments (Fragment H) matched one of the PLD transcribed sequences detected (c14372_g1_i1) (see Table 2). Additionally, one more sequence showed relation to a phospholipase B reported in spider *Parasteatoda tepidariorum* (see Table 1C).

**Figure 2:**
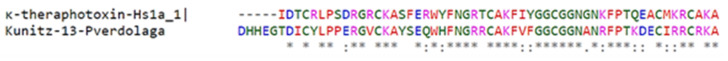
*Pamphobeteus verdolaga* sequence alignments. Pairwise sequence alignment of the protein sequence encoded in transcript c9496 (kunitz-13-pverdolaga) and the kunitz-type serine protease inhibitor huwentoxin-11 (κ-theraphotoxin-Hs1a) from *H. schmidti* (UniProt P68425). * (asterisk) indicates positions which have a single, fully conserved residue; : (colon) indicates conservation between groups of strongly similar properties, scoring >0.5; and . (period) indicates conservation between groups of weakly similar properties, scoring ≤0.5.

In the *P. verdolaga* venom gland transcriptome, we also found 13 contigs encoding members of the less common family of venom proteins known as kunitz-type serine protease inhibitors (KTSPI) (see Table 1D and Section S1 on Supplementary File S1). At least five sequences, kunitz-2-pverdolaga, kunitz-3-pverdolaga, kunitz-6-pverdolaga, kunitz-9-pverdolaga, and kunitz-13-pverdolaga, may correspond to the complete sequence conserving the cysteine residues necessary to form both disulfide bridges characteristics to kunitz toxins from Theraphosidaes. Out of all the described sequences, ten transcripts showed similarity to KTSPI reported in spiders, six of them matching different KTSPI from *H. schmidti* (Theraphosidae). Only three transcripts matched KTPSI from organisms that are not spiders (ant, tick, and mite). The match between the KTPSI and an external protein is presented in Figure 2, showing kunitz-13-pverdolaga from *P. verdolaga* aligned with a KTPSI from *H. schmidti*. The presence of this class of proteins in *P. verdolaga* venom was supported by the MS/MS identification of a peptide exhibiting 42.6% similarity with the amino acid sequence translated from contig 66767 (kunitz-9-pverdolaga) (see Table 2). Furthermore, the phylogenetic analysis suggested that the kunitz-type protein in *P. verdolaga* underwent multiple duplication events, diversifying this protein family in this species, indicating that sequences c41726 and c66767 are orthologous (branch supports > 50%) with *Araneus ventricosus* (see Figure S2 on Supplementary File S1).

**Figure 3:**
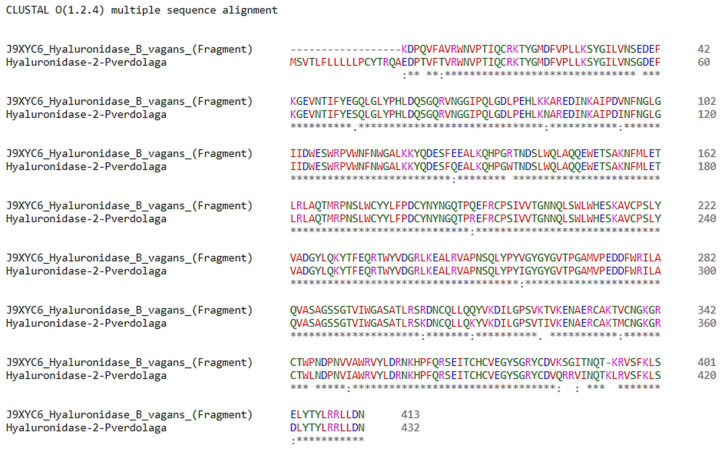
*Pamphobeteus verdolaga* sequence alignments. Pairwise sequence alignment of the amino acid sequence encoded in transcript c51925 (hyaluronidase-2-pverdolaga) and hyaluronidase J9XYC6 from *Brachypelma. vagans*. * (asterisk) indicates positions which have a single, fully conserved residue, : (colon) indicate conservation between groups of strongly similar properties, scoring >0.5 and . (period) indicates conservation between groups of weakly similar properties, scoring ≤0.5.

Two additional translated sequences of hyaluronidase-like proteins were detected and one of the contigs disclosed shows a sequence similarity of ~94% to hyaluronidase J9XYC6 from *B. vagans* (Theraphosidae) (see Figure 3) and had high transcriptional support (see Table 1E and Section S1 on Supplementary File S1).

**Figure 4:**
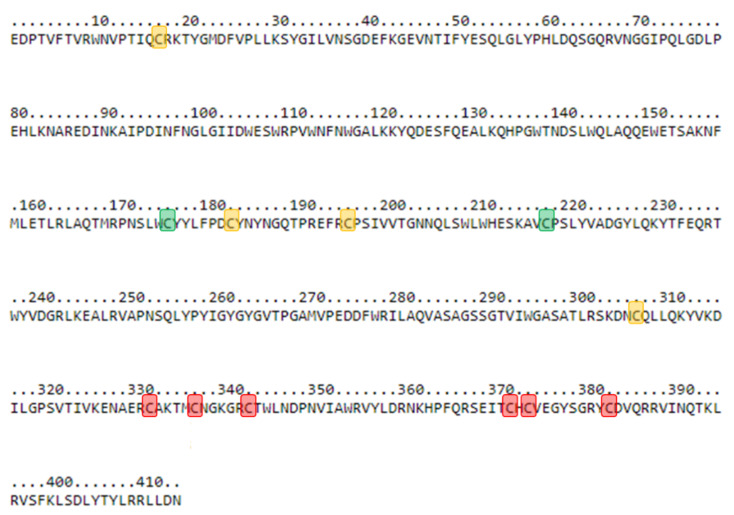
Mature hyaluronidase-2 translated from *Pamphobeteus verdolaga*. Cysteines’ pattern distribution of mature hyaluronidase-2-pverdolaga protein. Cysteines’ position are Cys17, Cys176, Cys183, Cys196, Cys218, Cys307, Cys332, Cys337, Cys343, Cys371, Cys373, and Cys383. Yellow highlighted residues correspond to cysteines’ residues involved in the catalytic domain according to the work in [11]. Red highlighted residues correspond to cysteines’ residues involved in the EGF-like domain according to the work in [11]. Green highlighted residues correspond to any known hyaluronidase domain according to the work in [11].

One MS/MS fragment matching the hyaluronidase sequence of hyaluronidase-2-pverdolaga (c51925) was detected with a similarity of 100% (see Table 2 below). Hyaluronidase-2-pverdolaga sequence contains two characteristic domains present in other hyaluronidases, the EGF-like domain, comprised of Cys332, Cys343, Cys337, Cys371, Cys373, and Cys383, and the catalytic domain comprised of Cys17, Cys183, Cys196, and Cys307 (see Figure 4, Supplementary Files S1 and S3).

**Figure 5:**
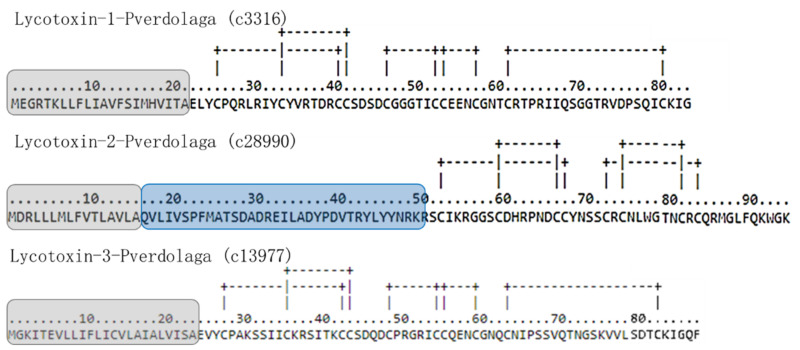
Amino acid sequences of lycotoxin translated from *Pamphobeteus verdolaga*. Prediction of disulfide bridges, commonly described in the ICK peptides, according to DISULFIND. Residues highlighted in grey indicate the signal peptide, while blue highlighted residues indicate the propeptide according to Spider|ProHMM from the arachnoserver.

Additionally, three translated sequences showed similarity to lycotoxins from *Lycosa singoriensis* and *S. minmosarum*, as well as to lycotoxins-like isoforms from different organisms, i.e., flies (see Table 1F and Section S1 on Supplementary File S1). The characteristic cysteine ICK motif is maintained in all sequences, potentially forming 4–5 disulfide bridges (see Figure 5). The phylogenetic analysis of the main members of the lycotoxin protein family reported in Uniprot suggested that *P. verdolaga* includes a basal protein (c28990) of the lycotoxin protein family, which underwent a duplication event that led to the origin of the lycotoxin ortholog protein from *Lycosa* sp. (see Figure S4 on Supplementary File S1). Additionally, *P. verdolaga* has two proteins closely related (c3316 and c13977) to the lycotoxin U20 of *Lycosa singoriensis* (Uniprot B6DD61). One of the MS/MS fragments, showing 100% similarity with the lycotoxin U16-lycotoxin-Ls1a, is highly similar to the contig c40556 (see Section S1 on Supplementary File S1).

Furthermore, we identified three more transcripts similar to the toxin family of cysteine-rich secretory proteins (CRISPs) showing high similarity (up to 95% similarity) with other CRISPs reported on *Grammostola rosea* (Theraphosidae) (see Table 1G). One transcript was identified by MS/MS (see Table 2) and had high transcriptional support with a TPM of 18191.29.

Finally, we found two more sequences that have the multicopper oxidase domine present (PF07732) (see Table 1H) and one more similar to the zinc metalloproteases that would be an important modulator of the hemostatic system [7] (see Table 1I). Based on phylogenetic analysis, *P. verdolaga* metalloproteinase is related to *A. ventricosus* (branch support 44%) (see Figure S3 on Supplementary File S1).

In addition to the previously detected HMMC, different proteins with catalytic activity, such as hydrolase, lipases, oxidoreductases, and peptidase, were identified by gene ontology terms using the Panther database (Supplementary File S1).

### 2.2. MS Findings

**Figure 6:**
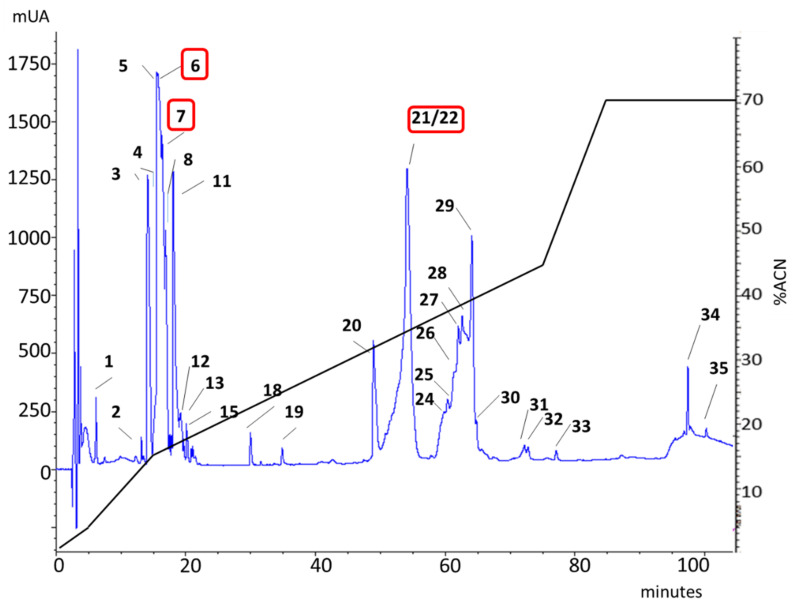
*P. verdolaga* venom profile. rp-HPLC (C18 column, 250 × 4.6 mm) chromatographic profile of *P. verdolaga’s* venom. Red squares indicate the HMMC detected by MS/MS similar to proteins with enzymatic activity.

*P. verdolaga* crude venom fractionation by reverse-phase (rp-HPLC) yielded 35 fractions, distributed in two main regions, in accordance with the previous description of *P. verdolaga* venom (see Figure 6).

After the local search of similarity analysis, using the FASTA program (fastm36) and the transcriptomic information as the database, 81 MS/MS fragments showed similarities to different HMMC. From this group, 10 fragments had similarities with proteins enhancing enzymatic activities including hyaluronidase, lycotoxin, cysteine-rich secretory protein (CRISP), phospholipase D, and kunitz-type (see Table 2 and Section S1 on Supplementary File S1). The MS/MS approach allowed the identification of three fragments that are 100% similar to four contigs identified by transcriptomic analysis (see Table 2 and Section S1 on Supplementary File S1).

**Table 2:** Assignment of the rp-HPLC fractions from *P. verdolaga* venom, isolated as observed in Figure 6, matching high-molecular-mass compound protein families with enzymatic activities from venom gland transcriptomic database.

rp-HPLC	Peptide Sequence	Similarity	Best Match	Protein Family
6	Y-GMDFVPLLKSYGILV-N	100%	c51925_g1_i1	Hyaluronidase
6	R-TIKDWYK-G	80%	c40556_g1_i1	Lycotoxin
21–22	K-SFPTVLTSSSMSFTK-K	84.6%	c9919_g1_i1	CRISP
	R-TGPQVKGEK-S	77.8%	c9919_g1_i1	
	K-DWYKEIK-D	55.2%	c9919_g1_i1	
	K-VATGKETQYSMPK-A	100%	c9919_g1_i1	
7	R-DSANGFINK-I	73%	c14372_g1_i1	Phospholipases D
	K-ESGYNDK-Y			
6	P-STYGGGLSVSSR-F	42.6%	c66767_g1_i1	Kunitz-type

The local and non-redundant external database Basic Local Alignment Search Tool (BLASTP) was used to search for similarities in the MS/MS fragments and match HMMC with enzymatic functions (see Table 3). BLASTP showed high similarity with other proteins reported in spiders from different genera, matching the same protein family as those identified by the local search using the transcriptomic info. Only two fragments did not match any fragments previously reported in the Araneae order (see Table 3).

Seventy-one more fragments showed a similarity above 75% with proteins matching housekeeping and cellular process proteins such as actin, ubiquitin, protein phosphatase, and heat shock protein (see Table 4). Twenty-two of these MS/MS fractions showed a similarity of 100% with proteins such as actin and heat shock proteins (see Table 4 and Section S2 on Supplementary File S1). The complete sequences of all transcripts are presented in Section S2 on Supplementary File S1. Other HMMC were detected in the rp-HPLC fractions with a similarity below 75% (data not shown).

## 3. Discussion

Spider venoms are a rich source of molecules with a diverse range of antibacterial, antifungal, antiviral, antimalarial, and anticancer bioactivities [12,13,14]. In this study, we described a combined venom gland transcriptomic and proteomic analysis of the Colombian Tarantula *P. verdolaga*, which revealed the presence of a wide array of novel proteins, including phospholipase D, phospholipase B, phospholipase A_2_, lycotoxin-like, kunitz-type serine protease inhibitors, and hyaluronidases.

Transcriptomic analysis showed the venom gland expression of 16 different PLA_2_-coding-like transcripts. Although no MS/MS fragments of any of the PLA_2_ transcripts were detected, the presence of these toxins is supported by previous studies of the *P. verdolaga* venom, where an estimated a minimum hemolytic dose (MHeD) of 307.1 µg was previously detected [8]. Three sequences (phospholipaseA2-8-pverdolaga, phospholipaseA2-9-pverdolaga, and phospho-lipaseA2-16-pverdolaga) fulfilled some characteristics to be considered as complete PLA_2_ sequences, including sequence similitude, molecular weight, number of cysteines, and cysteine–cysteine assembling pattern to form the respective disulfide bridges. PhospholipaseA_2_-8-pverdolaga has a similar cysteine pattern to a PLA_2_ from *S. mimosarum*, although, only showed the possible formation of 5 disulfide bridge including the characteristic Cys11-Cys77 disulfide bridge of Group I PLA_2_ (Supplementary File S3) [15,16]. PhospholipaseA2-9-pverdolaga is a 16-cysteine residue Group XIIA PLA_2_ of 216 amino acid residues length (~23.5 KDa), containing a signal peptide, with a mature protein potentially forming eight disulfide bridges (between cysteines 1 and 8, 2 and 12, 3 and 4, 5 and 16, 6 and 13, 7 and 10, 9 and 11, and 14 and 15) similar to the cloned Group XIIA-1 PLA_2_, indicating that phospholipaseA2-9-pverdolaga may be part of this PLA_2_ family [17]. However, the position and distribution of the cysteine residues is distinct from this PLA_2_. PhospholipaseA_2_-16-pverdolaga’s 774 amino acid residue length (~85 KDa) is similar to the phospholipase A-2-activating protein from *Ixodes scapularis*, a 805 amino acid residue length (~87 KDa) with 25 cysteine residues. Mature phospholipaseA_2_-16-pverdolaga with 23 cysteine residues that can potentially form 11 disulfide bridges (between cysteines 1 and 4, 2 and 6, 3 and 7, 5 and 17, 9 and 14, 10 and 18, 11 and 12, 13 and 21, 15 and 22, 16 and 23, and 19 and 20) with a different cysteine pattern when compared with the phospholipase A-2-activating protein cysteine pattern from *Ixodes scapularis*. The phylogenetic analysis showed multiple duplication events undergone during the evolution of phospholipase A_2_ in *P. verdolaga* (see Figure S1). Functional analysis could help to identify whether these gene redundancies are proteins with different functions. However, duplication events have been reported in spider venom proteins, which may help the species to adapt to the environment (e.g., prey capture and defense) [18]. Other kinds of lipases corresponding to PLD proteins were detected, although these proteins are not commonly distributed or reported in the Theraphosidae family and instead have been widely studied in the Sicariidae family [19]. The presence of these lipase proteins has been reported in the venom and venom gland of *H. hainanum* (Theraphosidae), together with different PLA_2_ and PLB proteins fragments and coding transcripts [2]. PhospholipaseD-1-pverdolaga (358 amino acids length) and phospholipaseD-2-pverdolaga (275 amino acids length) have the same number of cysteines and disulfide bridges, but the pattern is different (between cysteines 1 and 2, 3 and 4, 3 and 1, 3 and 4, and 2 and 3) when compared to the respective similar PLD. Additionally, the phospholipaseD-1-pverdolaga amino acid sequence conserved the active site amino acid residues (H32, E52, D54, H68, and D112) required to bind Mg^2+^ indicating that phospholipaseD-1-pverdolaga may be a complete PLD sequence including the disulfide bridges and active sites [20,21]. In arachnids, the presence of phospholipases may be associated with their ability to destroy lipid membranes, allowing other venom components to spread across tissues. The physio-pathological activity of these kind of proteins is clinically relevant, because these are the ones responsible for said venom’s dermonecrotic and inflammatory effects, the latter impairing the kidney’s normal function [22]. However, envenomation that involves infant patients or causes acute renal failure may result in a life-threatening situation, even if the amount of venom injected is considerably low (1–6 mg) [8]. This clinical picture strongly suggests the hitherto unreported presence of these proteins in *P. verdolaga* venom.

KTSPI proteins are HMMCs that have mainly been described and biologically characterized in snakes and bees (including wasps), and they impair enzymatic activities by blocking ion channels, altering blood coagulation, and interfering with inflammatory processes [23,24,25,26,27]. The biological function of these proteins in the spider venoms comprises activity in trypsin or chymotrypsin inhibition, K+ channel blocking, plasmin inhibitor, and an elastase inhibitor [28]. Although KTSPI proteins have been identified in species of the Mygalomorphae families, i.e., the brush-foot trapdoor spider *Trittame loki* (Barychelidae) [3,29,30,31], nearly all the KTSPI were isolated and reported from the species *H. hainanum* and *H. schmidti* [2]. From *H. hainanum*, 16 sequences were clustered as belonging to the kunitz-type toxins venom family and showed a native kunitz-type architecture, according to their number of cysteine residues [2]. In the venom gland transcriptome of *P. verdolaga*, 13 different KTSPI sequences were found. Seven of these *P. verdolaga* KTSPIs showed resemblance (e-values > 1 × 10^−10^) to that of *T. loki* (Barychelidae), *H. hainanum*, and to other KTSPI proteins from other species (see Figure 2). The presence of this group of proteins was confirmed by proteomics of *P. verdolaga* venom. Sequences from kunitz-2-pverdolaga, kunitz-3-pverdolaga, kunitz-6-pverdolaga, kunitz-9-pverdolaga and kunitz-13-pverdolaga showed the same cysteine–cysteine pattern forming all three disulfide bridges between cysteines 1 and 6, 2 and 4, and 3 and 5, according to Zweckstetter et al. and the disulfide prediction [32].

Hyaluronidases are the most commonly reported enzyme in spider venoms, and these proteins embody other HMMCs found in *P. verdolaga* venom [1]. These extracellular matrix-degrading proteins denote hyaluronan and facilitates venom spreading across vertebrate’s tissues. However, hyaluronidase is unlikely to play a key role in predation of invertebrates, indicating that it might play a defensive role [1]. According to Arachnoserver (http://arachnoserver.org/mainMenu.html accessed on 29 August 2019), three separate hyaluronidases belonging to three different spider families (Ctenidae, Sicariidae and Theraphosidae) have been reported to date. We had the opportunity to find two different contigs encoding two separate hyaluronidases. The first (hyaluronidase-2-pverdolaga) showed 94% similarity to *B. vagans* hyaluronidase-1 (*Bv*Hyal—J9XYC6). The second was the hyaluronidase-2-pverdolaga, a 414-amino acid protein with 12 cysteines that can potentially form six disulfide bonds. This hyaluronidase from *P. verdolaga* has the well preserved cysteine scaffold described on hyaluronidase-1-*Brachypelma vagans*, with the cysteine residues essential for catalytic activity in the same position (Cys17, Cys183, Cys196, and Cys307), which are essential for the catalytic activity [11]. In addition, the EGF-like domain, comprised of Cys332, Cys343, Cys336, Cys371, Cys373, and Cys383, is also present in the hyaluronidase-2 from *P. verdolaga* with one difference on amino acid position 332, where an asparagine (N) takes the place of a cysteine (C), which is in the position 337 [11]. Cysteine residues Cys176 and Cys218 are also present, allowing the potential formation of a sixth disulfide bond [11]. These two residues have been proposed to have a role in reinforcing the stability of the catalytic site in Arachnida hyaluronidases [11]. The same cysteine residues on hyaluronidase-2-pverdolaga are highlighted in Figure 4. The presence of this group of proteins was confirmed by proteomics of *P. verdolaga* venom.

Additionally, transcriptomic analysis of *P. verdolaga*, revealed three medium-molecular-mass compoundeds corresponding to “lycotoxin-like” peptides previously reported in the Eresidae and Lycosidae families, both unrelated or alien to the Mygalomorphae suborder to which *P. verdolaga* is taxonomically assigned. Two sequences showed an ICK motif previously described in the Theridiidae *Latrodectus tredecimguttatus* [33]. One more contig (c40556) may correspond to a lycotoxin from *P. verdolaga*. Although this contig is not similar to any reported protein, one MS/MS fragment similar to c40556 showed similarity to U16-lycotoxin-Ls1a from *Lycosa singoriensis*, indicating that c40556 may be a new member (new sequence) of the lycotoxin family. In addition, the phylogenetic analysis (see Figure S4) suggests (branch supports > 50%) that this sequence could be related to the lycotoxin protein family. In the phylogenetic tree, one of the lycotoxin-like sequence in *P. verdolaga* was located with good branch support (100%) as a basal protein from the lycotoxin protein family, which could be supported with the divergence time among spider families, since Theraphosidae (*P. verdolaga* family) diverged from a common ancestor 200 MYA (million years ago), while the family of Lycosa diverged 57 MYA [34]. Lycotoxins are a wide group of peptides that function as insecticides and pore formers, increasing the membrane permeability and cell lysis widely reported in the *Lycosa* genera [35,36]. Other HMMCs found in *P. verdolaga* venom with clinical importance are those with the domain multicopper oxidase and the venom metalloproteinases, which can attack the hemostatic system of prey [7].

Combined transcriptomic and proteomic analysis showed the presence of different HMMCs with housekeeping or cellular process proteins in *P. verdolaga* venom. Those housekeeping HMMCs detected by transcriptomic and proteomic analysis (previously reported in spider venoms) correspond to proteins where their biological function in spiders has not been determined. There were important cytoskeleton and structure transcripts expressed in the venom gland of *P. verdolaga*, including PDZ and LIM domain proteins and actin proteins, which are the main proteic components involved in the formation of filaments of the cytoskeleton [37,38]. The presence of actin in the venom gland of spiders, as well as other organisms, is proposed to be a structural component that allows venom gland contractile activity; these proteins are considered as ubiquitous components of the cytoskeleton, as previously reported in Theraphosidae spiders *C. jingzhao* and *Citharischius crawshayi* [4,37,39,40]. The presence of this HMMC or nontoxic compounds, is still unknown, but Yuan et al. [4] proposed that these nontoxic compounds may play a synergic role on the toxins in the venom, have some kind of unknown role in the venoms, or house-keeping proteins are secreted during the secretion of toxins to keep toxin-producing cells and the venom gland functional [4]. They could also be a contaminant, as proposed by Duan et al. in the venom of *Latrodectus tredecimguttatus* [41]. Heat shock proteins are chaperonins proteins that are involved in the direct folding and assembly of cellular proteins, as previously reported in the transcriptome of the Theraphosidae spiders *C. jingzhao*. According to Chen et al., this protein may be important for the secretion of toxin and regenerative proteins [37,42]. Other proteins are involved in nucleic acid metabolic process, including histones and zinc finger proteins, as previously described by Borges et al. in the venom of a *G. iheringi* (Theraphosidae) [5]. Although proteins such as cytosolic purine 5′-nucleotidase, dystonin, intersectin, and other enzymes which correspond to different proteins were previously described in other arachnids from the Theraphosidae family, e.g., *Grammostola rosea* and *Brachypelma smithi,* their function remains unknown. Additionally, contigs c16774_g1_i2, c5016_g1_i1, and c34105_g1_i1 showed 98–99% similarity with polyubiquitins and ubiquitins reported in other organisms (Section S2 on Supplementary File S1). Ubiquitin is a small and highly conserved polypeptide of 76 amino acids reported in different organisms that is involved in proteins degradation [43] and (in mammals) in the posttranslational modifications of plasma membrane proteins and voltage-gated sodium channels (Na_v_) in a process called ubiquitylation [44]. Although the role of this protein in spider venoms is not clear (besides the role in silk glands and proteins degradation [43,45]), it may be involved in the Nav activation after envenomation, since *P. verdolaga* ubiquitin’s similarity and a conserved domain at the α-subunit C-terminal make Na_v_ potential targets for the ubiquitins. The activation of these channels may play an important role during envenomation, facilitating the action of toxins and affecting Na_v_, which plays a synergic role, the later being previously reported *in P. verdolaga* [10].

## 4. Conclusions

Here, we present an update on the venom gland transcriptome and proteome from *Pamphobeteus verdolaga*. We report the amino acid sequences of different HMMCs with enzymatic activity or housekeeping functions present in their venom gland and venom, some of which are described for the first time in a species of the Theraphosidae family.

## 5. Materials and Methods

### 5.1. Spider Collection and Venom Extraction

Female *P. verdolaga* specimens were collected in the locality of La Estrella-Pueblo Viejo, Antioquia Province, Colombia. Venom from five specimens was obtained as previously described [8]. Identification was carried out according to the taxonomic description by Cifuentes et al. [7]. Venom extraction was conducted in accordance with: (a) the ethical principles in animal research adopted by the World Health Organization for the characterization of venoms [46,47]; and (b) the “Comité Institucional para el Cuidado y Uso de Animales” (CICUA). After each extraction, all animals were kept alive in captivity. Specimen collection was performed under National Agency for Ambient Licenses (ANLA according to the initials in Spanish) resolution 00908 2019 emitted to the University of Antioquia as a framework agreement.

### 5.2. Venom Fractionation

The venom profile of *P. verdolaga* was obtained using a combination of reverse-phase high-pressure liquid chromatography (rp-HPLC) and mass spectrometry (MS). First, 1 mg of crude venom was dissolved in 200 μL of solution A (0.1% trifluoroacetic acid in water) and centrifuged at 3500× *g* for 5 min at room temperature. Then, the supernatant was fractionated using a C_18_ rp-HPLC analytical column (250 × 4.6 mm RESTEK), balanced, and eluted initially at a flow rate of 1.0 mL/min isocratically using 5% of solution B (acetonitrile 99%) for 5 min, followed by a linear gradient of 5–15% B for 10 min, 15–45% B for 60 min, and 45–70% B for 12 min [48]. The chromatographic separation was monitored at 215 nm and fractions were collected manually, lyophilized, and stored at −20 °C until used.

### 5.3. Proteomic Analysis

#### LC-MS/MS

rp-HPLC fractions were run on a nano-Eksigent 425 HPLC system paired to a Triple-TOF 5600 plus (Sciex, Framingham, MA, USA) mass spectrometry system. The RP-HPLC system was run for 120 min at 300 nL/min over the cHiPLC nanoflex system. The trap column was a nano-cHiPLC (200 μm × 0.5 mm ChromXP C18-CL 3 μm 120 Å) and the analytical column, a nano-cHiPLC (75 μm × 15 cm ChromXP C18-CL 5 μm 120 Å). Elution was done with a gradient of 0.1% formic acid in water (A) and acetonitrile (B), of 5–35% B for 90 min, 35–80% B for 2 min, 80% B for 5 min, and 80–5% B for 20 min. The sample was sprayed to the Triple-TOF 5600 plus through a Nanospray III source equipped with an emission tip from New Objective.

### 5.4. Data Analysis

MS/MS spectra were interpreted manually or using a licensed version of ProteinLynx Global (Server version 2.5.2 software from Waters, Waters, Manchester, UK) or a free version of MASCOT (http://www.matrixscience.com, accessed on 29 August 2019). The ProteinLynx searches were made using tryptic digestion with 2 missed cleavages. The peptide tolerance was set to 10 ppm, while fragment tolerance and estimated calibration error were set to 0.05 and 0.005 Da, respectively. Carbamidomethyl cysteine and oxidation of methionine were fixed as well as variable modifications [49]. Triple-TOF MS/MS spectra acquired for 50 precursor ions at 250 ms/scan were analyzed using Mascot Daemon v.2.4.0 (Matrix Science, Boston, MA, USA) against different databases i.e., UniProt, NCBI or ArachnoServer [49,50,51]. The proteomic raw material can be found on the Supplementary Materials (see Section S3 on Supplementary File S1).

### 5.5. Transcriptomic Analysis

For the transcriptomic analysis, we used the DNA material isolated and amplificated from our previous report [10]. The transcriptomic analysis used to identify the HMMCs was performed similarly as carried out in our previous report with some modifications. Briefly, we used all contigs/singlets and translated them in six frames. Ortholog proteins were recovered using tBLASTX and tBLASTN programs (Sweet Version 2.28). Cleaving signals for each transcript were predicted using the stand-alone tool Spider|ProHMM (http://arachnoserver.org/peptides.html) (accessed on 29 August 2019), which uses a combination of SignalP v4.1 (http://www.cbs.dtu.dk/services/SignalP/ (accessed on 29 August 2019) and an HMM to predict signal and propeptide sites, respectively. After the prediction of hypothetical cleavage sites, mature peptides were aligned using the Clustal omega program [52]. The abundance of transcript abundance was measured by TPM using RSEM as was described by Estrada-Gomez et al. [10]. The MS/MS fragments identified from the venom of *P. verdolaga* by rp-HPLC matching were aligned to the reference transcriptome of *P. verdolaga* to identify the peptide similarity using the pipeline of FASTA program (fastm36) [53]. The best model for amino acid substitution and the phylogenetic analysis was estimated by Maximum-Likelihood using branch supports with the ultrafast bootstrap IQ-TREE [54]. All trees were run sampling 1000 replicates and trees were edited using iTOL [55]. The quality data of the transcriptomic material can be consulted at [10].

Finally, toxins validated via transcriptomic and proteomic analysis were uploaded into the European Nucleotide Archive ENA under accession: PRJEB21288/ERS1788422/ERX2067777-ERR2008012.

### 5.6. Nomenclature

Peptides and proteins identified by proteomic or transcriptomic experiments were named following the rational nomenclature proposed by King et al. [56], with some modifications for proteins (masses above 20 kDa), i.e., - protein group, followed by the isoform number and the species name.

### 5.7. Signal Peptide and Disulfide Bond Prediction

Signal peptides were predicted using the on-line software SignalP 5.0 Server available at http://www.cbs.dtu.dk/services/SignalP/ (accessed on 25 May 2021) [57]. Disulfide bonds were predicted using the Cysteines Disulfide Bonding State and Connectivity Predictor, DiANNA and DISULFIND available at http://clavius.bc.edu/~clotelab/DiANNA/ (accessed on 25 May 2021) and http://disulfind.dsi.unifi.it/ (accessed on 19 September 2020), respectively, web-based tools for disulfide engineering in proteins [58,59,60].

### 5.8. Data Availability

The transcriptomic datasets generated during and/or analyzed during the current study are available in the European Nucleotide Archive (ENA) repository under accession: PRJEB21288/ERS1788422/ERX2067777-ERR2008012. The proteomic raw material can be found in the Supplementary Materials.

## Figures and Tables

**Table 1:** Contigs from *P. verdolaga* venom gland transcriptome matching: A, phospholipases A_2_ (PLA_2_); B, phospholipases D (PLD); C, phospholipases B; D, kunitz-type serine protease inhibitors (KTSPI); E, hyaluronidases; F, lycotoxin-like peptides; G, CRISP proteins; H, hephaestin-like; and I, metalloproteinase. Accession numbers beginning with “XP” correspond to the NCBI database, while others correspond to UniProt. TPM, transcripts per million.

**A. Phospholipase A_2_-Like Proteins.**							
**Contig Number**	**Given Name**	**Similarity with**	**Accession Number**	**Organism**	**Score**	**E-Value**	**TPM**
c3142	PhospholipaseA_2_-1-pverdolaga	Calcium-independent phospholipase A_2_	A0A087UHX4	*Stegodyphus mimosarum*	173.33	7.09 × 10^−50^	5.79
c4865	PhospholipaseA_2_-2-pverdolaga	Cytosolic phospholipase A_2_	XP_003214621.2	*Anolis carolinensis*	112.08	2.93 × 10^−25^	5.45
c15998	PhospholipaseA_2_-3-pverdolaga	Group XV phospholipase A_2_	A0A087U096	*S. mimosarum*	560.84	0.00 × 10^+00^	28.76
c33599	PhospholipaseA_2_-4-pverdolaga	Cytosolic phospholipase A_2_	A0A087UL94	*S. mimosarum*	139.04	5.33 × 10^−39^	1.51
c45513	PhospholipaseA_2_-5-pverdolaga	Calcium-independent phospholipase A_2_	A0A087UHX4	*S. mimosarum*	275.02	7.49 × 10^−88^	3.09
c10524	PhospholipaseA_2_-6-pverdolaga	Calcium-independent phospholipase A_2_	A0A0J7L0J5	*Lasius niger*	101.29	1.51 × 10^−18^	6.49
c11106	PhospholipaseA_2_-7-pverdolaga	Phospholipase A_2_	A0A087SVA4	*S. mimosarum*	149.06	3.02 × 10^−41^	8.04
c12950	PhospholipaseA_2_-8-pverdolaga	Phospholipase A_2_	A0A087TLC5	*S. mimosarum*	87.04	2.14 × 10^−17^	58.98
c18752	PhospholipaseA_2_-9-pverdolaga	Group XIIA secretory phospholipase A_2_	XP_011150082.1	*Harpegnathos saltator*	135.58	3.87 × 10^−35^	138.53
c21159	PhospholipaseA_2_-10-pverdolaga	Calcium-independent phospholipase A_2_	E2B1P4	*Camponotus floridanus*	114.39	3.47 × 10^−24^	2.68
c42153	PhospholipaseA_2_-11-pverdolaga	Cytosolic phospholipase A_2_	A0A087UL97	*S. mimosarum*	117.86	1.08 × 10^−31^	2.65
c20465	PhospholipaseA_2_-12-pverdolaga	Calcium-independent phospholipase A_2_	XP_002399324.1	*Ixodes scapularis*	342.81	1.19 × 10^−105^	2.66
c29120	PhospholipaseA_2_-13-pverdolaga	Group 3 secretory phospholipase A_2_	XP_012259279.1	*Athalia rosae*	137.89	6.44 × 10^−36^	1.4
c57457	PhospholipaseA_2_-14-pverdolaga	Phospholipase A_2_	A0A087UYP4	*S. mimosarum*	118.24	1.83 × 10^−29^	2.01
c61053	PhospholipaseA_2_-15-pverdolaga	Calcium-independent phospholipase A_2_-γ	A0A087TW26	*S. mimosarum*	479.56	1.22 × 10^−158^	10.53
c60448	PhospholipaseA_2_-16-pverdolaga	Phospholipase A_2_-activating protein	B7PWX1	*Ixodes scapularis*	624	0.00 × 10^+00^	5.76
**B. Phospholipase D-Like Proteins.**							
**Contig Number**	**Given Name**	**Similarity with**	**Accession Number**	**Organism**	**Score**	**E-Value**	**TPM**
c46024	PhospholipaseD-1-pverdolaga	Phospholipase D LiSicTox-betaID1	Q1W694	*Loxosceles intermedia*	319.70	3.17 × 10^−100^	9.24
c14372	PhospholipaseD-2-pverdolaga	Phospholipase D StSicTox-betaIF1	C0JB54	*Sicarius terrosus*	214.54	4.16 × 10^−61^	16.94
c45658	PhospholipaseD-3-pverdolaga	Phospholipase D1	KFM64830.1	*S. mimosarum*	165	1 × 10^−48^	1.98
c54699	PhospholipaseD-4-pverdolaga	Phospholipase D1	XP_003744259.1	*Metaseiulus occidentalis*	353	4.94 × 10^−118^	2.34
**C. Phospholipase B-Like Proteins.**							
**Contig Number**	**Given Name**	**Similarity with**	**Accession Number**	**Organism**	**Score**	**E-Value**	**TPM**
c17591	Phospholipase-B-1-pverdolaga	Putative phospholipase B-like 2	XP_015925352.1	*Parasteatoda tepidariorum*	752	0.00 × 10^+00^	31.29
**D. KTPSI-Like Proteins.**							
**Contig Number**	**Given Name**	**Similarity with**	**Accession Number**	**Organism**	**Score**	**E-Value**	**TPM**
c1808	Kunitz-1-pverdolaga	Kunitz-type serine protease inhibitor huwentoxin-11g11	B2ZBB6	*H. schmidti*	75.87	9.22 × 10^−15^	5.22
c8989	Kunitz-2-pverdolaga	Kunitz-type serine protease inhibitor kunitz-1	W4VSH9	*Trittame loki*	66.24	4.76 × 10^−10^	23.43
c12801	Kunitz-3-pverdolaga	Kunitz-type serine protease inhibitor HWTX-XI-IS4	P0DJ76	*H. schmidti*	99.37	5.62 × 10^−23^	157.37
c15163	Kunitz-4-pverdolaga	Kunitz-type serine protease inhibitor kunitz-1	W4VSH9	*Trittame loki*	128.26	2.03 × 10^−32^	38.26
c16277	Kunitz-5-pverdolaga	Kunitz-type serine protease inhibitor 6-like	KFM65460.1	*S. mimosarum*	137.50	1 × 10^−38^	13.39
c30159	Kunitz-6-pverdolaga	Kunitz-type serine protease inhibitor huwentoxin-11g11	B2ZBB6	*H. schmidti*	92.82	4.50 × 10^−22^	76.26
c41726	Kunitz-7-pverdolaga	Protein with kunitz domain	XP_002435922.1	*Ixodes scapularis*	74.33	1.38 × 10^−16^	2.85
c59058	Kunitz-8-pverdolaga	Kunitz-type serine protease inhibitor huwentoxin-11g11	B2ZBB6	*H. schmidti*	64.70	1.40 × 10^−09^	4.26
c66767	Kunitz-9-pverdolaga	Kunitz-type protease inhibitor AXPI-I-like	XP_011135446.1	*Harpegnathos saltator*	63.16	4.85 × 10^−10^	1.58
c43290	Kunitz-10-pverdolaga	Kunitz-type protease inhibitor kalicludine-3-like	XP_012273912.1	*Orussus abietinus*	51.99	1.71 × 10^−06^	1.66
c52646	Kunitz-11-pverdolaga	Kunitz-type serine protease inhibitor huwentoxin-11g11	B2ZBB6	*H. schmidti*	84.73	2.64 × 10^−17^	144.34
c6182	Kunitz-12-pverdolaga	Kunitz-type serine protease inhibitor kunitz-1	W4VSH9	*Trittame loki*	64.31	9.75 × 10^−10^	3.02
c9496	Kunitz-13-pverdolaga	Kunitz-type serine protease inhibitor huwentoxin-11	P68425	*H. schmidti*	119.78	1.02 × 10^−30^	325.15
**E. Hyaluronidase-Like Proteins.**							
**Contig Number**	**Given Name**	**Similarity with**	**Accession Number**	**Organism**	**Score**	**E-Value**	**TPM**
c17398	Hyaluronidase-1-pverdolaga	Hyaluronidase-3	A0A0F8AST4	*Larimichthys crocea*	79.34	4.21 × 10^−13^	14.8
c51925	Hyaluronidase-2-pverdolaga	Hyaluronidase	J9XYC6	*Brachypelma vagans*	816.99	0.00 × 10^+00^	1107.75
**F. Lycotoxin-Like Peptides.**							
**Contig Number**	**Given Name**	**Similarity with**	**Accession Number**	**Organism**	**Score**	**E-Value**	**TPM**
c3316	Lycotoxin-1-pverdolaga	U15-lycotoxin-Ls1d	B6DD42	*Lycosa singoriensis*	50.1	6 × 10^−06^	7.36
c28990	Lycotoxin-2-pverdolaga	U16-lycotoxin-Ls1b	B6DD53	*Lycosa singoriensis*	42	8 × 10^−7^	2.35
c13977	Lycotoxin-3-pverdolaga	U20-lycotoxin-Ls1c-like	A0A087UBG5	*S. mimosarum*	52.37	2.96 × 10^−06^	6.38
**G. CRISP Proteins.**							
**Contig Number**	**Given Name**	**Similarity with**	**Accession Number**	**Organism**	**Score**	**E-Value**	**TPM**
c9788	CRISP-1- pverdolaga	GTx-CRISP1	BAN13537.1	*Grammostola rosea*	518	0.00 × 10^+00^	1388.9
C9919	CRISP-2- pverdolaga	GTx-VA1	BAN13538.1	*G. rosea*	590	0.00 × 10^+00^	18191.29
c18710	CRISP-3- pverdolaga	GTx-CRISP1	BAN13537.1	*G. rosea*	322	4 × 10^−108^	12.97
**H. Hephaestin-Like Protein.**							
**Contig Number**	**Given Name**	**Similarity with**	**Accession number**	**Organism**	**Score**	**E-Value**	**TPM**
c5907	hephaestin-1- pverdolaga	Hephaestin-like protein	XP_021003833.1	*Parasteatoda tepidariorum*	1017	0.00 × 10^+00^	4.18
c20814	hephaestin-2- pverdolaga	Hephaestin	PRD23536.1	*Nephila clavipes*	193	1 × 10^−59^	3.16
**I. Venom Metalloproteinase.**							
**Contig Number**	**Given Name**	**Similarity with**	**Accession Number**	**Organism**	**Score**	**E-Value**	**TPM**
c728	Metalloproteinase-1- pverdolaga	A disintegrin and metalloproteinase with thrombospondin motifs 1	KFM63257.1	*S. mimosarum*	758	0.00 × 10^+00^	20.99

**Table 3:** Assignment of the rp-HPLC fractions from *P. verdolaga* venom, isolated as observed in Figure 2, matching high-molecular-mass protein families with enzymatic activities from a non-redundant external database.

rp-HPLC	Protein Family	Protein Name	Organism
6	Hyaluronidase	Hyaluronidase, partial	*Brachypelma vagans*
6	Lycotoxin	U16-lycotoxin-Ls1a	*Lycosa singoriensis*
21–22	CRISP	GTx-VA1	*Grammostola rosea*
	CRISP	GTx-VA1	
	CRISP	GTx-VA1	
	CRISP	GTx-VA1	
7	Phospholipases D	Phospholipase D isoform 1	*Loxosceles laeta*
	Phospholipases D	Phospholipase D LlSicTox-alphaIII1i	
6	No match	No match	No match

**Table 4:** MS/MS fragments identified from the venom of *P. verdolaga* rp-HPLC matching high-molecular-mass compound with housekeeping and cellular process activities. ID, transcript identification number.

Sequences	% Similarity	ID	Match
AGFAGDDAPR	100	c6436_g1_i1	Actin
AVFPSIVGRPR			
DSYVGDEAQSKR			
HQGVMVGMGQKDSYVGDEAQSK			
RGILTLK			
EITALAPSTMK	100	c62193_g1_i1	Actin
VAPEEHPVLLTEAPLNPK	100	c13011_g1_i1	Actin
MTQIMFETFNSPAMYVAIQAVLSLYASGR	96.55		
ESRSE	100	c15096_g1_i3	Cytosolic purine 5′-nucleotidase
GKPKIQVEYK	100	c27174_g1_i1	Heat shock protein
LSKEEIER	100		
SENVQDLLLLDVAPLSLGIETAGGVMTALIK	90.32		
SENVQDLLLLDVAPLSLGIETAGGVMTSLIK	90.32		
GVPQIEVTFDLDANGILQVSAQDKSTGK	89.29		
QTQIFTTYSDNQPGVLIQVYEGER	95.8		
QTQTFITYSDNQPGVLIQVYEGER	95.8		
GVPQIEVTFDIDANGILNVTATDK	91.67		
EIAEAYLGYPVTNAVITVPAYFNDSQR	88.89		
LLQDFFNGR	88.89		
SENVQDLLLLDVAPLSLGLETAGGVMTALIK	87.1		
NQVALNPQNTVFDAK	86.67		
SENVQDLLLLDVAALSLGLETAGGVMTALIK	83.87		
DVLLVDVAPLSLGIETAGGVMTK	100	c15743_g1_i1	Heat shock protein
KLFNPEEISAMVLTK	100		
LFNPEEISAMVLTK	100		
DAGVIAGLNVLR	91.67		
TTPSYVAFTDTER	100	c10792_g1_i2	Heat shock protein
YRPGTVALREIR	100	c2143_g1_i2	Histone
TITLEVEPSDTIENVK	100	c16774_g1_i2	Polyubiquitin-B
AGFAGDDAPRAVFPSIVGRPR	100	c17180_g4_i1	Actin
DLYANTVLSGGTTMYPGIADRMQK	100		
MQKEITALAPSTMK	100		
SYELPDGQVITIGNER	100		
YSVWIGGSI	100		
TTGIVLDSGDGVSHTVPIYEGYALPHAILWLDLAGRDLTDYLMK	97.73		
MQKEITALAPSQMK	92.86		
MQKEITALAPSWMK	92.86		
MQKEITALAPSYMK	92.86		
SINPDEAVAYGAAVQAAILMGDK	95.65	c16820_g1_i1	Heat shock protein
IINEPTAAALAYGLDR	93.75	c9831_g1_i1	Heat shock protein
TITLEVEPSDTAENVK	93.75	c16774_g1_i2	Polyubiquitin-B
FELSGIPPAPR	90.91	c14336_g1_i1	Heat shock protein
AASSSSTEK	88.89	c51913_g1_i1	Actin
MAATKQTAR	88.89	c2143_g1_i2	Histone
KSAMATGGVK	80		
MAGTKQTAR	88.89	c4667_g1_i1	Histone
MANTKQTAR	88.89		
AVTKQTAR	87.5		
KSAAATGGVK	80		
KSACATGGVK	80		
KSAEATGGVK	80		
KSAGATGGVK	80		
KSAHATGGVK	80		
KSASATGGVK	80		
KSASATGGVK	80		
KSAWATGGVK	80		
KSAYATGGVK	80		
SAIATGGVKKPHR	84.62		
CNDMMNVGRLQGFEGK	87.5	c15300_g1_i1	Triple functional domain protein
ELEEAER	85.71	c34105_g1_i1	Ubiquitin
QAAEAAPEDK	80	c5964_g1_i2	PDZ and LIM domain protein Zasp
GSSSGGGYSSGSSSYGSGGR	80	c14908_g1_i2	Protein phosphatase
LENEIQTYR	77.78	c4688_g1_i1	Dystonin
CKINFCLK	75	c14491_g1_i2	AN1-type zinc finger protein
SPATREGK	75	c9864_g1_i1	Cip1-interacting zinc finger protein
KVLPLPQR	75	c62910_g1_i1	Developmental protein
EDQEQRER	75	c29740_g1_i1	Intersectin-1
KLMEMVNN	75	c11572_g1_i1	Serine/threonine-protein kinase
IPCCGKSR	75	c5016_g1_i1	Ubiquitin
VQGHSHSK	75	c17992_g1_i1	Uncharacterized protein

## Data Availability

Not applicable.

## Supplementary Materials

The following are available online at https://www.mdpi.com/article/10.3390/toxins13070453/s1, Supplementary File S1: Section S1: Full-length translated sequences for putative protein ORFs corresponding to phospholipases A2, phospholipases D, phospholipases B, kunitz-type, hyaluronidases, lycotoxins toxins, CRISP proteins, Hephaestin-like protein, and venom metalloproteinase. Section S2: Full-length translated sequences for putative protein ORFs corresponding to proteins matching housekeeping and cellular process proteins. Section S3: Raw material of proteomic reports. Section S4: Gene ontology terms using the Panther database of the different proteins with catalytic activity. Figure S1. Phylogenetic tree of phospholipase A2. The tree includes sequences of phospholipase A2 reported for different spider families. *P. verdolaga* sequences (green) are orthologous with different species, grouped with good branch supports (>50%). Multiple duplication events occurred during the evolution of phospholipase A2 in P. verdolaga. The triangle represents collapsed sequences from other species used to build the tree. Phospholipase A2 from Ixodes scapularis was used as outgroup. Figure S2. Phylogenetic tree of kunitz-like protein family. The tree includes the kunitz reported in databases for different spider families. The Phylogenetic tree show that *P. verdolaga* sequences (green) are orthologous with different species, grouped with good branch supports (>50%). The triangle represents collapsed sequences from other species used to build the tree. Kunitz from Ixodes scapularis was used as outgroup. Figure S3. Phylogenetic tree of the metalloproteinase. The tree includes the metalloproteinase reported in databases for different spider families. The Phylogenetic tree show that *P. verdolaga* sequence (green) is orthologous with the sequences reported for Araneus ventricosus with a branch support of 44%. The triangle represents collapsed sequences from other species used to build the tree. Metalloproteinase sequence from Ixodes scapularis was used as outgroup. Figure S4. Phylogenetic tree of lycotoxin-like protein. The tree includes the lycotoxin-like proteins reported in databases for different spider families. The phylogenetic tree supports (branch support 100%) that *P. verdolaga* sequence c28990 is a basal protein that belong to the lycotoxin-like family in spiders and multiple duplication events allowed the diversification of the protein in different spider species. The triangle represents collapsed sequences from other species used to build the tree. lycotoxin from Tentranychus urticae was used as outgroup. Supplementary File S2: Transcriptome abundance. Supplementary File S3: Prediction of Cys-Cys formation of putative protein ORFs corresponding to phospholipases A2, phospholipases D, phospholipases B, kunitz-type, hyaluronidases, lycotoxins toxins, CRISP proteins, Hephaestin-like protein and venom metalloproteinase.
