# Supplementary material for: Analysis of High Molecular Mass Compounds from the Spider Pamphobeteus verdolaga Venom Gland. A Transcriptomic and MS ID Approach
Source: Toxins (Basel). 2021 Jun 29;13(7):453. doi: 10.3390/toxins13070453 (PMC8309857; doi:10.3390/toxins13070453)
Supplement: Supplementary file 1 [file toxins-13-00453-s001.zip › toxins-1210969 File S1.pdf]

Supplementary Materials: Analysis of High Molecular Mass Compounds from the Spider *Pamphobeteus verdolaga* Venom Gland. A Transcriptomic and MS ID Approach

Sebastian Estrada-Gomez, Leidy Johana Vargas-Muñoz, Cesar Segura Latorre, Monica Maria Saldarriaga-Cordoba and Claudia Marcela Arenas-Gómez

Section S1. Full-length translated sequences for putative protein ORFs corresponding to phospholipases A2, phospholipases D, phospholipases B, kunitz-type, hyaluronidases, lycotoxins toxins, CRISP proteins, Hephaestin-like protein and venom metalloproteinase.

Pairwise alignments and amino acid sequences of phospholipases A2 translated from *Pamphobeteus verdolaga*. Residues highlighted in grey indicate the signal peptide, while magenta blue highlighted residues, indicates the propeptide according to Spider|ProHMM from the arachnoserver. Residues highlighted in yellow shows cysteines potentially forming disulfide bridges.

PhospholipaseA2-1-Pverdolaga:
XLGTGKSPVVAVETIDVFRPDSLWDACKMVALGVSAIGQLLIDQATQTDGRVIGRAQAMCSM
LNIPYYRLNPQLTENVGLDTTDNKTLVKMLWETTAYMHSMRQELEQLCNNYEDSGLD

```
CLUSTAL O(1.2.4) multiple sequence alignment


A0A087UHX4_Ca-ind_PLA2_(Fragment)      MVLIQMLDELERILGKPVVQHFDLIAGTSTGGILALVLATGKSMKECRCLYFRLKDKVFV     60
PhospolipaseA2-1-Pverdolaga            ------------------------------------------------------------     0


A0A087UHX4_Ca-ind_PLA2_(Fragment)      GTRPYDADLLESFLKKELGESTVMGDIEKPKIMITATRGDRKPADLHIFRNYKSPLEILN     120
PhospolipaseA2-1-Pverdolaga            ------------------------------------------------------------     0


A0A087UHX4_Ca-ind_PLA2_(Fragment)      HIEKDILTNEPLPNPREQLIWQAARATGAAPTYFRASGPYIDGGLISNNPTLDALTEIHQ     180
PhospolipaseA2-1-Pverdolaga            ------------------------------------------------------------     0


A0A087UHX4_Ca-ind_PLA2_(Fragment)      CNQSLTAVGKRDQVCKIKVIVSMGTGRPPLVAVDTIDVFRPDSLWGACRM-ALGVTNLGQ     239
PhospolipaseA2-1-Pverdolaga            ---------------------XLGTGKSPVVAVETIDVFRPDSLWDACKMVALGVSAIGQ     39
                                                             :***: *:***:***********.**:* ****: :**

A0A087UHX4_Ca-ind_PLA2_(Fragment)      LLVDQATQTDGRVTARALAVCGMLNIPYFRLNPQLTENVALDETNTKTLVKMLWETTAYM     299
PhospolipaseA2-1-Pverdolaga            LLIDQATQTDGRVIGRAQAMCSMLNIPYYRLNPQLTENVGLDTTDNKTLVKMLWETTAYM     99
                                       **:**********  .** *:*.******:**********.** *:.**************

A0A087UHX4_Ca-ind_PLA2_(Fragment)      RCMKDELEELKKLLTT----     315
PhospolipaseA2-1-Pverdolaga            HSMRQELEQLCNNYEDSGLD     119
                                       :.*::***:* :
```

PhospholipaseA2-2-Pverdolaga:
MAAESVADDVLRCCVNLSSEFRDFYCPYDIPSVEGPPTPLEFARNWVSPNKPVIFRNAVKHWP
ALKKWTVSYLR

```
CLUSTAL O(1.2.4) multiple sequence alignment


XP_003214621.2_cytosolic_PLA2_(predicted)      MAEAEAAVEAVRRCLASFPEEARELCFPESVPYLDSPPSPLQFFREWVCPNKPCVIRNAF    60
PhospholipaseA2-2-Pverdolaga                   -MAAESVADDVLRCCVNLSSEFRDFYCPYDIPSVEGPPTPLEFARNWVSPNKPVIFRNAV    59
                                                  **:..: * **  ..: .* *::  *  .:* ::.**:**:* *:**.**** ::***.

XP_003214621.2_cytosolic_PLA2_(predicted)      NHWPALKRWTLDYLREIMGEKLVSVAVTPNGYADAVYQDWFVMPEERLTPFSAFLDILEK    120
PhospholipaseA2-2-Pverdolaga                   KHWPALKKWTVSYLR---------------------------------------------    74
                                               :******:**:.***

XP_003214621.2_cytosolic_PLA2_(predicted)      KVTSPGVFYVQKQCSNLTEEFPELMDDLEPEIPWMSEALGKKPDAVNFWLGESAAVTSLH    180
PhospholipaseA2-2-Pverdolaga                   ------------------------------------------------------------    74

XP_003214621.2_cytosolic_PLA2_(predicted)      KDHYENLYCVISGEKHFLLHPPSDRPFIPHELYPPATYHISEDGNFEIVMDKMSEKVPWI    240
PhospholipaseA2-2-Pverdolaga                   ------------------------------------------------------------    74

XP_003214621.2_cytosolic_PLA2_(predicted)      PLDPLNPDLERYPEYAQAKPLRCTVKSGEMLYLPSLWFHHVQQSHGCIAVNYWYDMEYDL    300
PhospholipaseA2-2-Pverdolaga                   ------------------------------------------------------------    74

XP_003214621.2_cytosolic_PLA2_(predicted)      KYSYYQLLDSLTKAMTPTYADTMILYLRQYPQWGIHSYGEVDSLYPLSCKGTGVQYLEVY    360
PhospholipaseA2-2-Pverdolaga                   ------------------------------------------------------------    74

XP_003214621.2_cytosolic_PLA2_(predicted)      GFHIIERNTYFNALKRFLPPLPSIFHAMATQMPMVGTFPCYTLFVKIIQARHIPARDLWS    420
PhospholipaseA2-2-Pverdolaga                   ------------------------------------------------------------    74

XP_003214621.2_cytosolic_PLA2_(predicted)      FSDCYVTLWLTSTSKKKAVTKTISNTSNPVWNESFQFVIQTQVKNVLELKLYDEDVVTKD    480
PhospholipaseA2-2-Pverdolaga                   ------------------------------------------------------------    74

XP_003214621.2_cytosolic_PLA2_(predicted)      DLIFIVTYDISKVKPGETIQENFTLNAKGPESLEVEFKMEKICCGFEQIITNDILVAREV    540
PhospholipaseA2-2-Pverdolaga                   ------------------------------------------------------------    74

XP_003214621.2_cytosolic_PLA2_(predicted)      SCLEIQMDKGKNETCLKEHNNIELVVNESFEEAEKINQDSEAFQFHYVKNGEPILKAKLK    600
PhospholipaseA2-2-Pverdolaga                   ------------------------------------------------------------    74

XP_003214621.2_cytosolic_PLA2_(predicted)      SNFFKEKLFGDTPAHSHVLLKTLPLEEETEVALSITENAELKLQLKVNDCLGDLDVRLEC    660
PhospholipaseA2-2-Pverdolaga                   ------------------------------------------------------------    74

XP_003214621.2_cytosolic_PLA2_(predicted)      DLCAAEQDFLCKRKKHVARALENLLEQKLQNHEVPVIAVMATGGGARAMSAFYGHLSALQ    720
PhospholipaseA2-2-Pverdolaga                   ------------------------------------------------------------    74

XP_003214621.2_cytosolic_PLA2_(predicted)      KLNLLDCITYLCGASGSTWTMRSLYEDNDWSQKDLIGPIHKAQGHIVRNKSNVFSLEALQ    780
PhospholipaseA2-2-Pverdolaga                   ------------------------------------------------------------    74

XP_003214621.2_cytosolic_PLA2_(predicted)      FYDQELRRRRQEGYSVSFTDMWSLIIDRMFHDESNSKLSDQQQAVNKGQNPLPLYVALNV    840
PhospholipaseA2-2-Pverdolaga                   ------------------------------------------------------------    74

XP_003214621.2_cytosolic_PLA2_(predicted)      KEDAQTTPEFKEWCEFSPYEVGFSKYGAFIRSEDFGSEFYMGATDEDKPESRICFLEGIW    900
PhospholipaseA2-2-Pverdolaga                   ------------------------------------------------------------    74

XP_003214621.2_cytosolic_PLA2_(predicted)      SNIFSLNLVDVWNLYQLWQWPFSGQEEDNSKGSTSTTQVSEYHATCDTPSIFHGVLTRRP    960
PhospholipaseA2-2-Pverdolaga                   ------------------------------------------------------------    74

XP_003214621.2_cytosolic_PLA2_(predicted)      IGEGKPNFLRGLQLHKDYYQNKSFSMWQDSYLDQLPNHLTPLEKELCLVDAGYFINTSFP    1020
PhospholipaseA2-2-Pverdolaga                   ------------------------------------------------------------    74

XP_003214621.2_cytosolic_PLA2_(predicted)      PLLRKERNVDVIISLDYHLMETKFKSIENMSKYCIDQKIPFPKIVLTEEERSNPKECYLF    1080
PhospholipaseA2-2-Pverdolaga                   ------------------------------------------------------------    74

XP_003214621.2_cytosolic_PLA2_(predicted)      EDKENPEAPIILHFPLVNGSFKEYRKPGIKRKSAEKFEGEVDLDSSMSPYKMTDLTYTED    1140
PhospholipaseA2-2-Pverdolaga                   ------------------------------------------------------------    74

XP_003214621.2_cytosolic_PLA2_(predicted)      EFNKLLSLCDYNIQNNRELILQALWSAIEGGNISKL    1176
PhospholipaseA2-2-Pverdolaga                   ------------------------------------    74
```

PhospholipaseA$_2$-3-Pverdolaga:

MLSLSTHSRFVVSVFTLLSLLISSTTSYRIPKRSVRPSPVIFVPGDGGSQLQAKLNKPETVHYYCN

KKTDYYFDLWLNLELLVPYVLDCWIDNMRLIYDNVTRKTTNAPGVDIRVPGFGNTSTVEWLD
PSQIAPSAYFVRIVQGLVDEGYTRGVDLKGAPYDYRKAPNEMANYYKNVKQMTEEMYFKLN
KTRITYVCHSMGCPVMLYFFNRQTQDWKDTHVKALITLGGAWGGAVKAMKAFASGENLGV
YVINHLLLRKEQRTSPSLAYMTPSDTFWKKDEILVVTEKQNYTIGNYYDFFQDIRFPVGWEMW
KDTYNLTRDLIPPGVEVHCMHGVNVSTIERLVYKHLEFPDSNPTLIQGDGDGTVNLRSLEGCL
RWKGNQKQKVVHKPLNNVDHMGVLYDDDVIQYIKQVVSS

```
CLUSTAL O(1.2.4) multiple sequence alignment


A0A087U096_Group_XV_PLA2_(Fragment)      MFISCNVSVLAAGVVFLAFLRLHVTANHLLNRRADRHSPVILVPGDGGSQLEAKLDKPEI   60
PhospolipaseA2-3-Pverdolaga              MLSLSTHSRFVVSVFTLLSLLISSTTSYRIPKRSVRPSPVIFVPGDGGSQLQAKLNKPET   60
                                         *:  .. * :...*. *  * :  *:.: : :*: * ****:*********:***:***

A0A087U096_Group_XV_PLA2_(Fragment)      VHYFCNRKTENYFSLWLNLELLVPYVVDCWVDNMRMVYDNETRTSSNSPGVDIRVPGFGN   120
PhospolipaseA2-3-Pverdolaga              VHYYCNKKTDYYFDLWLNLELLVPYVLDCWIDNMRLIYDNVTRKTTNAPGVDIRVPGFGN   120
                                         ***:**:**: **.************:***:****::*** **.::*:************

A0A087U096_Group_XV_PLA2_(Fragment)      TTSVDWLDPSQISPSAYFVNIIDMLVTQGYTRGVDVRGAPYDFRKAPNEMTDYFKRLKNL   180
PhospolipaseA2-3-Pverdolaga              TSTVEWLDPSQIAPSAYFVRIVQGLVDEGYTRGVDLKGAPYDYRKAPNEMANYYKNVKQM   180
                                         *::*:*******:******.*:: ** :*******::*****:*******::*:*.:*::

A0A087U096_Group_XV_PLA2_(Fragment)      TEDTYEKNSQTKVTFICHSMGCPIMSYFFNQQTQAWKDEYIKALVSLGGAWGGAVKAMKT   240
PhospolipaseA2-3-Pverdolaga              TEEMYFKLNKTRITYVCHSMGCPVMLYFFNRQTQDWKDTHVKALITLGGAWGGAVKAMKA   240
                                         **: * * .:*::*::*******:* ****:*** *** ::***::*************:

A0A087U096_Group_XV_PLA2_(Fragment)      FTSGENLGVFVISQTNVRKEQRTCPSLAYMMPSDLLWGKDEILMITANKNYTVSNYYEFF   300
PhospolipaseA2-3-Pverdolaga              FASGENLGVYVINHLLLRKEQRTSPSLAYMTPSDTFWKKDEILVVTEKQNYTIGNYYDFF   300
                                         *:*******:**.:  :******.****** *** :* *****::* ::***:.***:**

A0A087U096_Group_XV_PLA2_(Fragment)      QDIDFPVGYEIYKDTYRYARTGLSPPGVEVHCLHGLNVTDTVVKLDFRNTSHFPDNPKLI   360
PhospolipaseA2-3-Pverdolaga              QDIRFPVGWEMWKDTYNLTR-DLIPPGVEVHCMHGVNVST-IERLVYKHLEFPDSNPTLI   358
                                         *** ****:*::****. :* .* ********:**:**:  : :* ::: ..  .**.**

A0A087U096_Group_XV_PLA2_(Fragment)      YGDGDGTVNVRSLRACLQWQGKQKQKVNHAAIYNVDHMGILADAHVLEYIKNVVHQW   417
PhospolipaseA2-3-Pverdolaga              QGDGDGTVNLRSLEGCLRWKGNQKQKVVHKPLNNVDHMGVLYDDDVIQYIKQVVSS-   414
                                          ********:***..**:*:*:***** *  : ******:* * .*::***:** .
```

PhospholipaseA$_2$-4-Pverdolaga:
XFVNNICTSSVLDTRKGRAGLILNPLRGLSLIPCFNFSPFSPTSPSDDMLFKGLTEAVPTQSKTLY
LVDGGLTFNLPFPLLLRPQRGIDVYLAFDFSSRDADH

```
CLUSTAL O(1.2.4) multiple sequence alignment


A0A087UL94_Cytosolic_PLA2_(Fragment)     MLNPGFWKDFINNIFTSSVLDTRKGRAGRVFNPLRGLSLIPCFPFSPFSPTSPSDNTLFK  60
PhospolipaseA2-4-Pverdolaga              --------XFVNNICTSSVLDTRKGRAGLILNPLRGLSLIPCFNFSPFSPTSPSDDMLFK  52
                                                  *:*** ************* ::************ ***********: ***

A0A087UL94_Cytosolic_PLA2_(Fragment)     GLTEPAPTNSKTLYLVDGGLTFNLPFPLLLRSQRAVDIYISFDFSSREHDNSPPFKELLL  120
PhospolipaseA2-4-Pverdolaga              GLTEAVPTQSKTLYLVDGGLTFNLPFPLLLRPQRGIDVYLAFDFSSRDADH---------  103
                                         **** .**:********************** **.:*:*::******: *:

A0A087UL94_Cytosolic_PLA2_(Fragment)     SEKWARLNNCLFPPIHDLAAEYIKHPPKECYVFKDPV 157
PhospolipaseA2-4-Pverdolaga              ------------------------------------- 103
```

PhospholipaseA$_2$-5-Pverdolaga:
XPSQSKKCTCSFDCSGGDSMEIVPQNCRVLCLDGGGIRGLVLIQLLDQLEKVLGIPVNLCFDWI
AGTSTGGVLALLLAQGKSVKECRCLYFRLKDRVFVGMRPYDAEPLEKILQKELGYETMMSDV
TGARVMVTATKSDRHPAELHVFRNYDSPMEILTQEDLDPFHNTPLPKPSEQLVWKVARATGS
APTYFRAFGAFLDGGLISNNPTLDALTEIHQCNQAYRVTHQEEKIKEIDX

```
CLUSTAL O(1.2.4) multiple sequence alignment


A0A087UHX4_Ca-ind_PLA2_(Fragment)      ---------------------------------------MVLIQMLDELERILGKPVVQH   21
PhospolipaseA2-5-Pverdolaga            XPSQSKKCTCSFDCSGGDSMEIVPQNCRVLCLDGGGIRGLVLIQLLDQLEKVLGIPVNLC   60
                                                                              :****:**:**::** **

A0A087UHX4_Ca-ind_PLA2_(Fragment)      FDLIAGTSTGGILALVLATGKSMKECRCLYFRLKDKVFVGTRPYDADLLESFLKKELGES   81
PhospolipaseA2-5-Pverdolaga            FDWIAGTSTGGVLALLLAQGKSVKECRCLYFRLKDRVFVGMRPYDAEPLEKILQKELGYE   120
                                       ** ********:***:** ***:************:**** *****: **.:*:**** .

A0A087UHX4_Ca-ind_PLA2_(Fragment)      TVMGDIEKPKIMITATRGDRKPADLHIFRNYKSPLEILNHIEKDILTNEPLPNPREQLIW   141
PhospolipaseA2-5-Pverdolaga            TMMSDVTGARVMVTATKSDRHPAELHVFRNYDSPMEILTQEDLDPFHNTPLPKPSEQLVW   180
                                       *:*.*:    ::*:***:.**:**:**:****.**:***.: : * : * ***:* ***:*

A0A087UHX4_Ca-ind_PLA2_(Fragment)      QAARATGAAPTYFRASGPYIDGGLISNNPTLDALTEIHQCNQSLTAVGKRDQVCKIKVIV   201
PhospolipaseA2-5-Pverdolaga            KVARATGSAPTYFRAFGAFLDGGLISNNPTLDALTEIHQCNQAYRVTHQEEKIKEIDX--   238
                                       :.*****:******* * ::**********************:  .. :.::: :*.

A0A087UHX4_Ca-ind_PLA2_(Fragment)      SMGTGRPPLVAVDTIDVFRPDSLWGACRMALGVTNLGQLLVDQATQTDGRVTARALAVCG   261
PhospolipaseA2-5-Pverdolaga            ------------------------------------------------------------   238


A0A087UHX4_Ca-ind_PLA2_(Fragment)      MLNIPYFRLNPQLTENVALDETNTKTLVKMLWETTAYMRCMKDELEELKKLLTT   315
PhospolipaseA2-5-Pverdolaga            ------------------------------------------------------   238
```

PhospholipaseA2-6-Pverdolaga:
MTICTSNSKYLFVLQILWIILPFSLAFNVRKWANNSDDCNYDLQVENDGPVTLDVPITFYAVLE
CASAEEHYVYIFQDNAVPPHRIQVDGSTGANVSFVYNANIYRPGVYILKVSVFSGMWSPVLVG
IASTSSTFVISEYIPGSLNISDIKVRNQGGSLYISSGSVTNLTINLHYPSSVYPLLETSYSWNVEKDQ
FITVDPFIIYNFTQPGTYRISVSAVARVPVYNVLAVPTQIMKYKWGYFNTVATVKDSMTAVNLT
GNTYLKHGQLLNLDVSCTGSGPFEYCWKIFQPFENVTDLTCPSPIVTTKCSFPIIYYFQESGNYQ
VAILVDNYITSIQRNIEVHVYDVSLKPQLSTVILPLVCAVLAIFIITIGIVIHIRENQQFDIETADFD
FLQSDVIVVETFWEKMYHSILQVLCLRREVQSNYYLRIVSPDASSSSHYGSVPXIGAVRRIIIVTV
WMIGRCHHVSKSLTAEFLYWFLRLRFQHTVKEKLFDSPNASTWNWPHFRNCNYLLVL

```
CLUSTAL O(1.2.4) multiple sequence alignment


A0A0J7L0J5_Ca-ind_PLA2           MYMVDVEVSY-RTWGIIWTFDTSARIEFEITEYLNGNVTAKQTNKTIAGDYISSVNETKL   59
PhospolipaseA2-6-Pverdolaga      MTICTSNSKYLFVLQILWIIL-PFSLAFNVRKWANNSDDCNY--------DLQVENDGPV   51
                                 * :   : .*  .  *:* :      : *:: :: *..  .:           :.  *:  :

A0A0J7L0J5_Ca-ind_PLA2           MIDIRKGDYDFIQKATAISTYWFI------------DCKYYGQTNDFVFAYNFTSPGMTH   107
PhospolipaseA2-6-Pverdolaga      TLDVPITFYAVLECASAEEHYVYIFQDNAVPPHRIQVDGSTGANVSFVYNANIYRPGVYI   111
                                  :*:   * .:: *:* . * :*                  * . .**:  *:  **:

A0A0J7L0J5_Ca-ind_PLA2           -------------------EIGALVIASYNPQTTTTVLPPTTTTAPINVTTVSPNAIMTN   148
PhospolipaseA2-6-Pverdolaga      LKVSVFSGMWSPVLVGIASTSSTFVISEYIPGSL--------NISDIKVRNQGGSLYISS   163
                                                     .::**:.* * :           . : *:* . . .  ::.

A0A0J7L0J5_Ca-ind_PLA2           PNGTRITVP--LTTTMLPT------------TVASADPSIKPATAAP---PIAVSTIDAA   191
PhospolipaseA2-6-Pverdolaga      GSVTNLTINLHYPSSVYPLLETSYSWNVEKDQFITVDPFIIYNFTQPGTYRISVSAVARV   223
                                  . *.:*:     ::: *               . :.** *    : *    *:**::  .

A0A0J7L0J5_Ca-ind_PLA2           NIYLPYICSNTSLIPPDPNKTYGYFYKKIHVRAPVTNISVEGTNWIQPWDMLSLNVTCKG   251
PhospolipaseA2-6-Pverdolaga      PVYNV-----LAVPTQIMKYKWGYFNTVATVKDSMTAVNLTGNTYLKHGQLLNLDVSCTG   278
                                  :*         ::     : .:*** .    *:   :* :.: *..:::   ::*.*:*:*.*

A0A0J7L0J5_Ca-ind_PLA2           SGPFSKCLQFHHGKYNVTGNETCNNAEQLRSCNFSILHYFLEPSVYTILIILNNEVNKQV   311
PhospolipaseA2-6-Pverdolaga      SGPFEYCWKIFQPFEN-VTDLTCPSPIVTTKCSFPIIYYFQESGNYQVAILVDNYITSIQ   337
                                 ****. * ::.:    *  . : ** .      .*.* *::** * . * : *:::* :..

A0A0J7L0J5_Ca-ind_PLA2           YPLTINIYKVTTKPQLSVIVVPVSCSLVAVVLIVFGVAYYIQSRARFTVEVADFDFGQSN   371
PhospolipaseA2-6-Pverdolaga      RNIEVHVYDVSLKPQLSTVILPLVCAVLAIFIITIGIVIHIRENQQFDIETADFDFLQSD   397
                                   : :::*.*: *****.:::*: *:::*:.:*.:*:. :*:.. :* :*.***** **:
```

```
A0A0J7L0J5_Ca-ind_PLA2          PEMEYKTFTERLRDSFNNTMCNVGKIQKLCDT------------------------LVEY   407
PhospolipaseA2-6-Pverdolaga     VIVV-ETFWEKMYHSILQVLCLRREVQSNYYLRIVSPDASSSSHYGSVPXIGAVRRIIIV   456
                                  :  :** *:: .*: :.:*   ::*.                            ::

A0A0J7L0J5_Ca-ind_PLA2          PTWTLAHL----------AAYFALHDAFMHAVVNSQLNSGDLETGISPLQVAVQTNNLRT   457
PhospolipaseA2-6-Pverdolaga     TVWMIGRCHHVSKSLTAEFLYWFLRLRFQHTVKEKLFDSP--------------------   496
                                 .* :.:             *: *:  * *:* :. ::*

A0A0J7L0J5_Ca-ind_PLA2          VEMLIAAKSSLEHLDHNANTVYHYAATSTKEIILALGSGLPNSLNSRNSNGYTPIHVACQ   517
PhospolipaseA2-6-Pverdolaga     -----------------NASTWNWPHFRNCNYLLVL------------------------   515
                                                   :.:::    . : :*.*

A0A0J7L0J5_Ca-ind_PLA2          NDKPECVKALLLIGADVNMPATEGQPSSPGYVGDFLHDKPNVLHAEDMKFGGTPLHWSRS   577
PhospolipaseA2-6-Pverdolaga     ------------------------------------------------------------   515

A0A0J7L0J5_Ca-ind_PLA2          REVITALIDTNCDIDALNFDGRTALHVMVMRKRLPCVVALLSHMASVNIVDNDGNTPLHL   637
PhospolipaseA2-6-Pverdolaga     ------------------------------------------------------------   515

A0A0J7L0J5_Ca-ind_PLA2          AVEAETPAIVQTLIGFGADIDAQNWKSETPSHKANIDTTEGNKIIYLLHAIGAERCSSEM   697
PhospolipaseA2-6-Pverdolaga     ------------------------------------------------------------   515

A0A0J7L0J5_Ca-ind_PLA2          TGCHLGCKYGENYTGIAPSEPPHYVPRTILDQMLHVSGMEKMAERGCDKRIKGGRLLCLD   757
PhospolipaseA2-6-Pverdolaga     ------------------------------------------------------------   515

A0A0J7L0J5_Ca-ind_PLA2          GGGIRGLVLIQTLLEIESVLQKPVVHCFDWIAGTSTGGILALGLAAGKSLRECQALYFRI   817
PhospolipaseA2-6-Pverdolaga     ------------------------------------------------------------   515

A0A0J7L0J5_Ca-ind_PLA2          KEDAFVGSRPYNSEGLERVLQDCLGKYTVMANIEKPKIMITGVLADRKPVDLHLFRNYES   877
PhospolipaseA2-6-Pverdolaga     ------------------------------------------------------------   515

A0A0J7L0J5_Ca-ind_PLA2          PSALLKVPENTMFKSTLLPRQQLLWKAARATGAAPSYFRAFGRFLDGGLIANNPTLDAMT   937
PhospolipaseA2-6-Pverdolaga     ------------------------------------------------------------   515

A0A0J7L0J5_Ca-ind_PLA2          EIHEYNLALKATDREKEAIPLSLVVSIGTGLVPTTLTVNEIDVYRPESLWDTAKLAFGIS   997
PhospolipaseA2-6-Pverdolaga     ------------------------------------------------------------   515

A0A0J7L0J5_Ca-ind_PLA2          ALGTLLVDQATASNGRVVDRARTWCSMIGIPYYRFNPQLTSDVAMDEKSDEILANMIWTA   1057
PhospolipaseA2-6-Pverdolaga     ------------------------------------------------------------   515

A0A0J7L0J5_Ca-ind_PLA2          KAFMHANRNQVKELAAIIDRDTNLDND      1084
PhospolipaseA2-6-Pverdolaga     ---------------------------      515
```

PhospholipaseA$_2$-7-Pverdolaga:

MTLGAPNIRRKIKRDLLDLADMFEELTQLDPTEYIPYGNWCGYGGDGEILDRIDRCCEIHDRC
YGKVSENVCSNEQVHIINYQWNRENDTITCDGNTSKCEMEACMCDRDVVLCIHKHNGDYSH
EVRYVDSTKPKTSGVPQWSSMNKDCTKRSDGSEIPLMGRMGNMSLIFX

```
CLUSTAL O(1.2.4) multiple sequence alignment


A0A087SVA4_PLA2_(Fragment)        MLPDKFCVIFLWMSLAFAYVSSGKLRRRQRDLLDLADMFDTLTGLDPTDYIPYGNWCGYG    60
PhospholipaseA2-7-Pverdolaga      ----------------MTLGAPNIRRKIKRDLLDLADMFEELTQLDPTEYIPYGNWCGYG    44
                                                  ::  : .  *: :**********: ** ****:***********

A0A087SVA4_PLA2_(Fragment)        GQGKPVDHIDSCCQIHDECYGQS-EKKCSNVQVHVVQYAWKINNATIICSD-EANCEASI    118
PhospholipaseA2-7-Pverdolaga      GDGEILDRIDRCCEIHDRCYGKVSENVCSNEQVHIINYQWNRENDTITCDGNTSKCEMEA    104
                                  *:*: :*:** **:***.***:  *: *** ***:::* *: :* ** *..  ::** .

A0A087SVA4_PLA2_(Fragment)        CKCDKEVVECIAQHSHTYSEHYRFIRKSR-------------------------------    147
PhospholipaseA2-7-Pverdolaga      CMCDRDVVLCIHKHNGDYSHEVRYVDSTKPKTSGVPQWSSMNKDCTKRSDGSEIPLMGRM    164
                                  * **::** ** :*.  **.. *:: .::

A0A087SVA4_PLA2_(Fragment)        --------      147
PhospholipaseA2-7-Pverdolaga      GNMSLIFX      172
```

PhospholipaseA2-8-Pverdolaga:

MGFLLTAALTFILLAGYSPFSAEKPLQIRRNKRSLFDLNDMIKQLTGRSGLDFIGYGNYCGFGGE
GKPVDDIDRCCKMHDICYDFAQNDDCAEDPNVVYKIKYGWQQKSFGVQCSFSQSKCMKVV
CICDVRFAKCLKNYINEYNNSNKHEKDLQELLEEVQQMSK

```
CLUSTAL O(1.2.4) multiple sequence alignment


A0A087TLC5_PLA2_(Fragment)        -----------MVTLAWAVSSGVLLFFVKGCSSSVLDLGNMLRVMTGRNPLDFVNYGNWC    49
PhospholipaseA2-8-Pverdolaga      MGFLLTAALTFILLAGYSPFSAEKPLQIRRNKRSLFDLNDMIKQLTGRSGLDFIGYGNYC    60
                                             ::  .::  *.   : ::  . *::**.:*:: :***. ***:. ***:*

A0A087TLC5_PLA2_(Fragment)        GLGGSGPVVDKIDRCCKMHDFCYDKASDTVCGEEKPHL--AAYTWKYAAGKIHCNKVDRP    107
PhospholipaseA2-8-Pverdolaga      GFGGEGKPVDDIDRCCKMHDICYDFAQNDDCAEDPNVVYKIKYGWQQKSFGVQCSFSQSK    120
                                  *:**.*  ** .*********.*** *.:   *.*:   :    * *: : :*.  :

A0A087TLC5_PLA2_(Fragment)        CGLASCRCDAKFAICVSIYKDSYDPRNKRTRPIFSIISNIAQLHPTRKSSESGISIRIG    166
PhospholipaseA2-8-Pverdolaga      CMKVVCICDVRFAKCLKNYINEYNNSNKHEKDLQELLEEVQQMSK--------------    165
                                  *   . * **.:** *:. * :.*:  **: : : .::.:: *:
```

PhospholipaseA2-9-Pverdolaga:

MERKYLLYCAVVLGSFIITYPQSLFAGVKNVLDSVNAIVEEVSLGLRSLSAGLDFVDQFVQTAG
SEECLFHCPSGKKLVPNQKYKPVPSGCGAYGVTLSVKNSPQKEFTECCNYHDICYGTCLSKKEI
CDEKFDKCLNKACAKQAKEIGEKKFGDCKMAAKVFYAGTVALGCKAFLDAQAEACICPEA
WSVRLCVRSTRQICVVYGEDAIAPRTA

```
CLUSTAL O(1.2.4) multiple sequence alignment


XP_011150082.1_Group_XIIA_Sec_PLA2_(Predicted)      MDLSRYRKFIIYVLTFLAYAWSGYGSGLLSNLRDAVLAAESVFHDFFENAITVARKIKDI      60
PhospholipaseA2-9-Pverdolaga                        ----MERKYLLYCAVVLGSFIITYPQSLFAGVKNVLDSVNAIVEEVSLGLRSLSAGLDFV      56
                                                        **:::*  ..*.    * ..*::.:::.: :.:::..:.  .  :::  :. :

XP_011150082.1_Group_XIIA_Sec_PLA2_(Predicted)      HEVFDAAVEENCVFQCPDGSAPKPDWNHKPRSNGCGSLGIEVSQEYLPLAEMTKCCDFHD      120
PhospholipaseA2-9-Pverdolaga                        DQFVQTAGSEECLFHCPSGKKLVPNQKYKPVPSGCGAYGVTLSVKNSPQKEFTECCNYHD      116
                                                    .:..::* .*:*:*:**.*.   *: ::**  .***: *: :* :  *  *:*:**:**

XP_011150082.1_Group_XIIA_Sec_PLA2_(Predicted)      ICYDTCNTDKEKCDLEFKRCLYKRCDTY-QTTGLTIVNACKGAAKVLFTGTTALGCKSFL      179
PhospholipaseA2-9-Pverdolaga                        ICYGTCLSKKEICDEKFDKCLNKACAKQAKEIGEKKFGDCKMAAKVFYAGTVALGCKAFL      176
                                                    ***.** :.** ** :*.:** * *  .  :  *  . .. ** ****:::** .*****:**

XP_011150082.1_Group_XIIA_Sec_PLA2_(Predicted)      DAQKEACYCPDKGSTRKNKKPKKAA-QAGGEL-------      210
PhospholipaseA2-9-Pverdolaga                        DAQAEACICPEAWSVRLCVRSTRQICVVYGEDAIAPRTA      215
                                                    *** ***.**:  *.*   : .:   . **
```

PhospholipaseA2-10-Pverdolaga:

FGYIMVLKNGSLSNDSEEVATGENLTMIAKILDGNGYLKDADIRCTWSIDMERFDLEGTSLNY
TYYDPGMSYIAVAVFATLPSSKTVFGLFTKELVVKVPVSDITISGNPFIHHNEVLNLNVSWTGTP
PFEYCWDIINSNETVEGNFTCMVIVTYDTSFPVTRYFQKNGTYTMAIHVSNDVKLVKRNMEIIV

FSVLPKSQLSTVIIPIVCSLLTLVIIAIGIAYYRQQRRQLIVEVASFDFHDNSDSYRERTFFEQLWDS FRCRGCCSPSLSVRSDCLPSENEPLLT

```
CLUSTAL O(1.2.4) multiple sequence alignment


E2B1P4_Ca-ind_PLA2               MQTAYFYCQFIWGVWCFLQVTATDYVKLSYDGPTVLGANITFRADLYTEQSWFPQKQYKY  60
PhospolipaseA2-10-Pverdolaga     ------------------------------------------------------------  0


E2B1P4_Ca-ind_PLA2               VWSDSLKNTYMIITLNRTVFWNTSYPRENNTSGNYKMTLQVYNKIHKLFPIWIFVTSDSI  120
PhospolipaseA2-10-Pverdolaga     ------------------------------------------------------------  0


E2B1P4_Ca-ind_PLA2               DFEITEYLNGNMTIKQNNKTIMRDYISSINEAKLIADIHKEDYDFIIQKAMTISTFWFID  180
PhospolipaseA2-10-Pverdolaga     ------------------------------------------------------------  0


E2B1P4_Ca-ind_PLA2               CQYYGQTNDFTFAYNFTNPGTTHEIEALVIASDNPPTTTTILPPTTTTATNVTTVPPNIT  240
PhospolipaseA2-10-Pverdolaga     ---------FGYIMVLKNGSLSNDSEEV------------------ATGENLTMIA----  29
                                          * :   :.* . ::: * :                  :*. *:* :

E2B1P4_Ca-ind_PLA2               TASLNGTHVTAVTTTMLPTTIASANPAIIASPITVS----MTDATNISFP-------YVC  289
PhospolipaseA2-10-Pverdolaga     -KILDGNGYL--------------KDADIRCTWSIDMERFDLEGTSLNYTYYDPGMSYIA  74
                                    *:*.                 : * * .  ::.      :.*.:.:        *:.

E2B1P4_Ca-ind_PLA2               SNISFIPPDPKKIYGYFHKKIYVRVPISNISVEGTNWIQPWDMLSLNVTCKGSGPFSKCL  349
PhospolipaseA2-10-Pverdolaga     VAVFATLPSSKTVFGLFTKELVVKVPVSDITISGNPFIHHNEVLNLNVSWTGTPPFEYCW  134
                                   :    *. *.::* * *:: *:**:*:*::.*. .*:  ::*.***: .*: **. *

E2B1P4_Ca-ind_PLA2               QFHHGKYNVTGNETCSNVEHLHSCNFSILHYFLEPSVYTILIILNNEIGAHIYPLTINIY  409
PhospolipaseA2-10-Pverdolaga     DIINSNETVEGNFTCMVIVTY-DTSFPVTRYFQKNGTYTMAIHVSNDVKLVKRNMEIIVF  193
                                 :: :.: .* ** **  :    . .* : :** : ..**: * :.*::      : * ::

E2B1P4_Ca-ind_PLA2               KVTTKPQLSVIVVPVTCSLVAVVLIVFGVAYYIQSRARFTVEVANFDFGQKASGLIVPLP  469
PhospolipaseA2-10-Pverdolaga     SVLPKSQLSTVIIPIVCSLLTLVIIAIGIAYYRQQRRQLIVEVASFDFHDNSDSYRERT-  252
                                 .*  * ***.:::*:.***:::*:*.:*:*** *.* :: ****.***  :::..

E2B1P4_Ca-ind_PLA2               QNIVTHHKISTMAWLGTIANNVLRN--IVFSDVPPNVVQEVRPEQYSNRRIHSREDGIVL  527
PhospolipaseA2-10-Pverdolaga     --------FFEQLWDSFRCRGCCSPSLSVRSDCLPSENE---P--------------LLT  287
                                         :    * .  ...       * **  *.  :   *              ::

E2B1P4_Ca-ind_PLA2               YGPGEIKNQDKYEIVLHRPCTETLHQAYSLFRSETLEEADARFLIYKDKVPVLVQIAREI  587
PhospolipaseA2-10-Pverdolaga     ------------------------------------------------------------  287


E2B1P4_Ca-ind_PLA2               CNVGKIQKLCDTLVEHPAWTLAHLAAYFALHDAFMHAAVNSQLNSGDLETGISPLQVAIQ  647
PhospolipaseA2-10-Pverdolaga     ------------------------------------------------------------  287


E2B1P4_Ca-ind_PLA2               TNNLRTVELLIASKSSLEHLDHNANTVYHYAATSTKEIILALGSGLPNSLNSRNSNGHTP  707
PhospolipaseA2-10-Pverdolaga     ------------------------------------------------------------  287


E2B1P4_Ca-ind_PLA2               IHVACQNDKPECVKALLLIGADVNIPATEGQPSSPGYVGDFLHNKPNVLHAEDMKFGGTP  767
PhospolipaseA2-10-Pverdolaga     ------------------------------------------------------------  287


E2B1P4_Ca-ind_PLA2               LHWSRSREVITALIDTNCDIDALNFEGRTALHVMVMRKRLPCVVALLSHMASVNIVDNDG  827
PhospolipaseA2-10-Pverdolaga     ------------------------------------------------------------  287


E2B1P4_Ca-ind_PLA2               NTPLHLAVEAETLAIVQTLIGFGADIDARNWKSETPRHKANIDTTEGNKIIYLLHAVGAE  887
PhospolipaseA2-10-Pverdolaga     ------------------------------------------------------------  287
```

```
E2B1P4_Ca-ind_PLA2              KCPAEMTGCHLGCKYGENYTGIAPSEPPRYVPRTILDQMLHVSSMEKMAEQGRDKRIKGG  947
PhospholipaseA2-10-Pverdolaga   ------------------------------------------------------------  287

E2B1P4_Ca-ind_PLA2              RLLCLDGGGIRGLVLIQTLLEIESVLRKPVVHCFDWIAGTSTGGILALGLAAGKSLRECQ  1007
PhospholipaseA2-10-Pverdolaga   ------------------------------------------------------------  287

E2B1P4_Ca-ind_PLA2              ALYFRIKEDAFVGSRPYNSEGLEKVLKECLGTYTVMADIDKPKIMITGVLADRKPVDLHL  1067
PhospholipaseA2-10-Pverdolaga   ------------------------------------------------------------  287

E2B1P4_Ca-ind_PLA2              FRNYESPSALLKVPGNTMFKTTLSSREQLLWKAARATGAAPSYFRAFGRFLDGGLIANNP  1127
PhospholipaseA2-10-Pverdolaga   ------------------------------------------------------------  287

E2B1P4_Ca-ind_PLA2              TLDAMTEIHEYNLALKATGREKEAIPLSLVVSIGTGLMPTTVTLNEIDVFRPESLWDTAK  1187
PhospholipaseA2-10-Pverdolaga   ------------------------------------------------------------  287

E2B1P4_Ca-ind_PLA2              LAFGISALGTLLVDQATASDGRVVDRARTWCSMIGIPYYRFNPQLTEDVAMDEKSDEILA  1247
PhospholipaseA2-10-Pverdolaga   ------------------------------------------------------------  287

E2B1P4_Ca-ind_PLA2              HMIWTAKAFMHANRDQVKELAAIIDRDTNLDND    1280
PhospholipaseA2-10-Pverdolaga   ---------------------------------    287
```

PhospholipaseA2-11-Pverdolaga:
XGDLVDTPDPYVVLKVPGSPNGKKRTKYFNNTINPTWKETFTFVLDPEKNYELEVILMDANYT
IDQRLGX

```
CLUSTAL O(1.2.4) multiple sequence alignment


A0A087UL97_Cytosolic_PLA2_(Fragment)     MECETLADQLLLSEQPISEVPPLPSLQVFQVTPDSCNILNVTVVEAHKITKGWVGDLVDK  60
PhospholipaseA2-11-Pverdolaga            -----------------------------------------------------XGDLVDT  7
                                                                                              *****.

A0A087UL97_Cytosolic_PLA2_(Fragment)     PDPYVILRIPGSPNGMKRTKHFNNTSSPTWNEEFTFVLDPQKEYELEITLMDANYTIDEK  120
PhospholipaseA2-11-Pverdolaga            PDPYVVLKVPGSPNGKKRTKYFNNTINPTWKETFTFVLDPEKNYELEVILMDANYTIDQR  67
                                         *****:*::****** ****:**** .***:* *******:*:****: *********::

A0A087UL97_Cytosolic_PLA2_(Fragment)     MG-   122
PhospholipaseA2-11-Pverdolaga            LGX   70
                                         :*
```

PhospholipaseA2-12-Pverdolaga:
MSIIRDILGGFRKVSAQVDDPFRVLEVNVEDYLTYDVVCREDCLVLYKANDRGVMKLEIVVQL
HINHSSNKNNKVYSLHRSEDETNCQILFSQMCQKIPILIDYVPEVGLSKMALQNVSQVIRENLA
WNAAHIAAHFGYTDCFKYKTMASEISEPCEGTLQTPLHVAIKASQFPSVVALVALDVVMDIV
DCNGDSIFHYAATTTKEIIQALSVKPCVPVINMLNHDGHTPLHLACMADKPECVKELLRAGA
DVNMASIVDVDEVDRAQAAEMPSKLLSDVMHTHAQRLYMDDMKTGGTPLHWSKTSELTAI
LIEYGCHIDAKNFEGNTALHVMVLRNRISCAVTLLSHGANVDIQGADGNTPLHLAVKSGDIY
LVYAFVAFGANVNAINNKGETPRHILATEKRPGFEEMLYALHIVGAERCQRRTPWCKDGCEP
GQHFNGIPSENPPVLNKTTLLDDLLGAT

```
CLUSTAL O(1.2.4) multiple sequence alignment


XP_002399324.1_Ca-ind_PLA2_(partial)      ------------------------------------------------------------ 0
PhospholipaseA2-12-Pverdolaga             MSIIRDILGGFRKVSAQVDDPFRVLEVNVEDYLTYDVVCREDCLVLYKANDRGVMKLEIV 60


XP_002399324.1_Ca-ind_PLA2_(partial)      ----VSIPPADGAVSCSLLRTKDQVAAEVSFVQLRDKLPLLLECVPDLL-SKDLVQEVVL 55
PhospholipaseA2-12-Pverdolaga             VQLHINHSSNKNNKVYSLHRSEDETNCQILFSQMCQKIPILIDYVPEVGLSKMALQNVSQ 120
                                              :.    ..    ** *::*:. .:: * *: :*:*:*:: **::  **  :*:*

XP_002399324.1_Ca-ind_PLA2_(partial)      LVRNNPSWTLAHLAAHLGLVDCFKNQKVAAQICQPAQDTLATPLHIAVRAQKLNSVQVLM 115
PhospholipaseA2-12-Pverdolaga             VIRENLAWNAAHIAAHFGYTDCFKYKTMASEISEPCEGTLQTPLHVAIKASQFPSVVALV 180
                                          ::*:* :*. **:***:* .**** ..:*::*.:*.:.** ****:*::*.:: ** .*:

XP_002399324.1_Ca-ind_PLA2_(partial)      GMDAPLNLTDHNGDTIYHAAAVTTKELIKALSVRPAPTAVINQVNNDGYTPLQLACLTDK 175
PhospholipaseA2-12-Pverdolaga             ALDVVMDIVDCNGDSIFHYAATTTKEIIQALSVKPCV-PVINMLNHDGHTPLHLACMADK 239
                                          .:*. :::.* ***:*:* **.****:*:****:*.    *** :*:**:***:***::**

XP_002399324.1_Ca-ind_PLA2_(partial)      PECVRELLKEGADVNSASSRGSNCT-----FFFFFSLGRDHVEQNGHNFQIEDMKHGGTP 230
PhospholipaseA2-12-Pverdolaga             PECVKELLRAGADVNMASIVDVDEVDRAQAAEMPSKLLSDVMHTHAQRLYMDDMKTGGTP 299
                                          ****:***: ***** **   . : .        :  .*  * :. :.:.: ::*** ****

XP_002399324.1_Ca-ind_PLA2_(partial)      LHWAKTTQCLETMIELGCDLDAKNFQGNTALHIMVARGRLACVISLLSHGASVNAVGCDG 290
PhospholipaseA2-12-Pverdolaga             LHWSKTSELTAILIEYGCHIDAKNFEGNTALHVMVLRNRISCAVTLLSHGANVDIQGADG 359
                                          ***:**::    :** **.:*****:******:** *.*::*.::******.*:  *.**

XP_002399324.1_Ca-ind_PLA2_(partial)      DTPLHAAVR-GDVSLIHALIVFGADVNQPNQKGETARHLAATSKLSKRDSVLYTLHAVGA 349
PhospholipaseA2-12-Pverdolaga             NTPLHLAVKSGDIYLVYAFVAFGANVNAINNKGETPRHILATEKRPGFEEMLYALHIVGA 419
                                          :**** **: **: *::*::.***:**  *:**** **: **.*    :.:**:** ***


XP_002399324.1_Ca-ind_PLA2_(partial)      RRCDRD-QGCSEGCSPEGSFDGVAPEKPNFFKASRREWAGLGGGASAIFLPLKLNRRSLA 408
PhospholipaseA2-12-Pverdolaga             ERCQRRTPWCKDGCEPGQHFNGIPSENPPVLNKTTLLDDLLGAT---------------- 463
                                          .**:*    *.:**.*   *:*:  *:* .:: :      **.

XP_002399324.1_Ca-ind_PLA2_(partial)      VGSKWRLTDGRCVAGKTPRQCLQLYFSLKDKMGVGRRRSRELTPMGSKWRLNDGRCVAGK 468
PhospholipaseA2-12-Pverdolaga             ------------------------------------------------------------ 463


XP_002399324.1_Ca-ind_PLA2_(partial)      TPRQCLQLYFSLKDKVFIGNRPHDADSLEKFLQREMGETTLMTDIKHPKLMITGVLADRH 528
PhospholipaseA2-12-Pverdolaga             ------------------------------------------------------------ 463


XP_002399324.1_Ca-ind_PLA2_(partial)      PAALHLFRNYDSPKKILGVTEDESDFPSCTPPHEQLVWRAARASGAAPTYFRPFGRFLDG 588
PhospholipaseA2-12-Pverdolaga             ------------------------------------------------------------ 463


XP_002399324.1_Ca-ind_PLA2_(partial)      GLISNNPTLDAMTEICEYNEALKATVRLWLPFNIFENELFCIFDITYHINIHRDVNVKVR 648
PhospholipaseA2-12-Pverdolaga             ------------------------------------------------------------ 463


XP_002399324.1_Ca-ind_PLA2_(partial)      TQQATQANGRLVDRAQAWCRTIGVPYFRLNAPISEDVCLNETDNRLLVRVLWETLVYMRG 708
PhospholipaseA2-12-Pverdolaga             ------------------------------------------------------------ 463


XP_002399324.1_Ca-ind_PLA2_(partial)      RRAELDELAQLVRP         722
PhospholipaseA2-12-Pverdolaga             --------------         463
```

PhospholipaseA$_2$-13-Pverdolaga:
XRLHELDEPADAEPLIQNSGLPAKYPSFKMMLELINNCSQLELPVHKVASSIATNQSEPNNTIS
KWSPWALWNGIVPGTKWCGVGDIASTFEELGSQAVVDSCCRAHDHCPVKLKAFRVGYGMI
NLSFYTKSHCDCDRLFHSCLKQTKNKLANAVGNFYFNFIRVQCLKERKVYVCVENRTDVDGL
NECIRWSVDPDSRKX

```
CLUSTAL O(1.2.4) multiple sequence alignment


XP_012259279.1_Secretory_PLA2_isoform_X2      MGLAVIFLLVITCSVQTLQQPTIFRNTYKGLRGVIPKVRLPDEEIRAAYFHDQTVAVIDL     60
PhospolipaseA2-13-Pverdolaga                  ------------------------------------------------------------     0


XP_012259279.1_Secretory_PLA2_isoform_X2      SNSNQLKNCDIIEVYEPLEAMEVIKNLSTEVQPQQVSFIEMTTLMRQCEMLEDMKEDAIS     120
PhospolipaseA2-13-Pverdolaga                  --------XRLHELDEPADAEPLIQNSG--LPAKYPSFKMMLELINNCSQLELPVHKVAS     50
                                                        : *: ** :*  :*:* .  :  :  **  *  *:.:*. **   ... *

XP_012259279.1_Secretory_PLA2_isoform_X2      TLPI-DTKNRGSKPGVNPLSLLFGILPGTKWCGTGDIADNYHDLGQEARIDRCCRSHDLC     179
PhospolipaseA2-13-Pverdolaga                  SIATNQSEPNNTISKWSPWALWNGIVPGTKWCGVGDIASTFEELGSQAVVDSCCRAHDHC     110
                                              ::   ::: ..:     .* :*  **:*******.****..:.:**.:* :* ***:** *

XP_012259279.1_Secretory_PLA2_isoform_X2      PVKVRAQKSRYNLTNNSIYTKSHCVCDEMLYHCLKKAYHPTADVMGRIYFNFIRVPCVED     239
PhospolipaseA2-13-Pverdolaga                  PVKLKAFRVGYGMINLSFYTKSHCDCDRLFHSCLKQTKNKLANAVGNFYFNFIRVQCLKE     170
                                              ***::* :  *.: * *:****** **.::: ***:: :  *:.:*.:******* *:::

XP_012259279.1_Secretory_PLA2_isoform_X2      VTNGTQGSVKRFID---VKMNY--------     258
PhospolipaseA2-13-Pverdolaga                  RKVYVCVENRTDVDGLNECIRWSVDPDSRK     200
                                               .  .  . :  :*     :.:
```

PhospolipaseA2-14-Pverdolaga:

XCRAHDLCDDTLAPGETKHNLTNRSTFTKLNCQCDQEFYECLQKVDSLVSNSIGNLYFNVLR
RGCYEYDHPLTKCKSYRT

```
CLUSTAL O(1.2.4) multiple sequence alignment


A0A087UYP4_PLA2_(Fragment)      MEHIVRCLLTFCLVVIVSCKSAFREKMFFLQRPSGEENKTLLVVTWSEGRQPEATGCEIF  60
PhospolipaseA2-14-Pverdolaga    ------------------------------------------------------------  0


A0A087UYP4_PLA2_(Fragment)      TNEELIQNILKLSPDQSVKKPSPEEMKSLLEDCTEISIRRKRSAEHETRRTGDDRRIYGN  120
PhospolipaseA2-14-Pverdolaga    ------------------------------------------------------------  0


A0A087UYP4_PLA2_(Fragment)      EPHTRNDRRSHSYESHTVNSRGKDFNNGNNRRVVIEESWSNGDRSSINENGANYDNTEDY  180
PhospolipaseA2-14-Pverdolaga    ------------------------------------------------------------  0


A0A087UYP4_PLA2_(Fragment)      ESTTMIPPNEMEGPKKESSTTEGYDGLPVIFPGTKWCGAGDIAKSYDDLGLHQDTDRCCR  240
PhospolipaseA2-14-Pverdolaga    ---------------------------------------------------------XCR  3
                                                                                          **

A0A087UYP4_PLA2_(Fragment)      AHDLCNDTLAPGETRNNLTNNSPFTKLSCKCDQDFYDCLDRINSVTANTIGNMYFNLLKR  300
PhospolipaseA2-14-Pverdolaga    AHDLCDDTLAPGETKHNLTNRSTFTKLNCQCDQEFYECLQKVDSLVSNSIGNLYFNVLRR  63
                                *****.*********::****.* ****.*:***:**:**::::*:.:*:***:***:*:*

A0A087UYP4_PLA2_(Fragment)      ECYKKDYPRSSKCKRYRSLLKITCKEYEKDANAPEVYQWVPAKRYKNLPVPGPFSVTLPF  360
PhospolipaseA2-14-Pverdolaga    GCYEYDHPL-TKCKSYRT------------------------------------------  80
                                 **: *:*  :*** **:
```

PhospolipaseA2-15-Pverdolaga:

MPRSQNAFSRNSSIYSSLYTRSVNISKEKRKLLLHKTYCTSKRSSDLNVSKPSSDKIPGNSIFSTIW
KSVVVASSMLRPGLSVTPPKSIPIREFISKVGNVVSTEKYSKLLQPYLKLRSVNSEENVRQTTEKS
RTCSKPGVSQAGNVISGEKESVFEQAAAEWITSANLQEKKQENLSNQKEKKSENLPKVLISKAS
LASRSRFLVRSLSCASSCSSQMLRLEEVCKHLLQHPQEKGTLVKEGLIRVALRLRRKSSNTDIQT
QACVALTLLGYHEPPGGQGIRILSIDGGGTRGLMAIEILRQLQARTGKTVHEMFDYICGVSSGA
ILTFLLGGLRLSPDECESLYRELSLEVFKASGIWGAGRLMWYHAYYDTSMWVDVLRKTFGDK
MLIDSVKEKSSPKLAAISAVMNLPALRAFVFRNYDYPIRVQSQYIGSANYRMWEAIRASGAAP
GYFEEFHLNHLLHQDGGIMINNPTALAIHEARLLWPSDYIQCVFSLGSGRFTPATNTAFTSTTL
KTKVQKVIDSATDTEAVHISMNDLLSPGTYFRFNPYLTEFLHLDENRPDKLHQLKMDAQMYL
RRNEHKLEQSIKVLTRPRSTLKKINDWIQLQKTLLL

```
CLUSTAL O(1.2.4) multiple sequence alignment


A0A087TW26_Ca-ind_PLA2-gamma_(Fragment)      -MKAHGVLSQAISWRSFPIFRAVSSSKRKKRFWFSSHYGTSSKPASSNLNSSKNNFLNLI      59
PhospholipaseA2-15-Pverdolaga                MPRSQNAFSRNSSIYSSLYTRSVNISKEKRKLLLHKTYCTSKRSSD--LNVSKPSS---D      55
                                               :::..:*:  *  *    *:*. **.*::: : . * **.: :.  ** ** .

A0A087TW26_Ca-ind_PLA2-gamma_(Fragment)      RNTTDNLRPMIWKSVTVASSLFNNVTLRFSKNKIVEEPKSVSV-----------------      102
PhospholipaseA2-15-Pverdolaga                KIPGNSIFSTIWKSVVVASSMLRPGLS-------VTPPKSIPIREFISKVGNVVSTEKYS      108
                                             :   :.:   *****.****::.           *  ***: :

A0A087TW26_Ca-ind_PLA2-gamma_(Fragment)      --------------STNIQKLSEKTSTSGG-KMTVIPGGQSDKREASLEQGFAEWLTGDE      147
PhospholipaseA2-15-Pverdolaga                KLLQPYLKLRSVNSEENVRQTTEKSRTCSKPGVSQAGNVISGEKESVFEQAAAEWITSAN      168
                                                           . *::: :**: *..   ::   .  *.::*: :**. ***:*. :

A0A087TW26_Ca-ind_PLA2-gamma_(Fragment)      LQTKKKEFFG-KEEKYVEKSKKVVISKSSLQQRSRFLVSSLIDATSSGSQLLRLEEICKH      206
PhospholipaseA2-15-Pverdolaga                LQEKKQENLSNQKEKKSENLPKVLISKASLASRSRFLVRSLSCASSCSSQMLRLEEVCKH      228
                                             ** **:* :. ::**  *:  **:***:** .****** **  *:*..**:*****:***

A0A087TW26_Ca-ind_PLA2-gamma_(Fragment)      LLLYPDQKSTMCKAGLVRTALHMKCNSSDNAVRAQARCTLSLVGYNESPKGHGIRILSID      266
PhospholipaseA2-15-Pverdolaga                LLQHPQEKGTLVKEGLIRVALRLRRKSSNTDIQTQACVALTLLGYHEPPGGQGIRILSID      288
                                             **  :*::*.*: * **:*.**::: :**:. :::**  :*:*:**:* * *:********

A0A087TW26_Ca-ind_PLA2-gamma_(Fragment)      GGGTRGIIAIEVLRQLEARTNKRIYELFDFMCGVSSGAVLSLLLGGLRLSLDECEALYRR      326
PhospholipaseA2-15-Pverdolaga                GGGTRGLMAIEILRQLQARTGKTVHEMFDYICGVSSGAILTFLLGGLRLSPDECESLYRE      348
                                             ******::***:****:***.* ::*:**::*******:*::******** ****:***.

A0A087TW26_Ca-ind_PLA2-gamma_(Fragment)      LSDEVFSQSSFWGTSRLMWSHAYYDTTMWVKVLKKTFGEKLLIDTCKEEFAPKLAAVSAL      386
PhospholipaseA2-15-Pverdolaga                LSLEVFKASGIWGAGRLMWYHAYYDTSMWVDVLRKTFGDKMLIDSVKEKSSPKLAAISAV      408
                                             ** ***. *.:**:.**** ******:***.**:****:*:***: **: :*****:**:


A0A087TW26_Ca-ind_PLA2-gamma_(Fragment)      MNLPHLQAFVFRNYDFPPHVQSYYHGSCRYRMWEAIRASGAAPGYFEEYCLDDYLHQDGG      446
PhospholipaseA2-15-Pverdolaga                MNLPALRAFVFRNYDYPIRVQSQYIGSANYRMWEAIRASGAAPGYFEEFHLNHLLHQDGG      468
                                             **** *:********:* :*** * **..*******************: *:. ******

A0A087TW26_Ca-ind_PLA2-gamma_(Fragment)      IMVNNPTALAIHEAQLLWPSDGIQCVMSLGSGRYIPPVQPNFTSTSLKTKVLKVIDSATD      506
PhospholipaseA2-15-Pverdolaga                IMINNPTALAIHEARLLWPSDYIQCVFSLGSGRFTPATNTAFTSTTLKTKVQKVIDSATD      528
                                             **:***********:****** ****:******: *  .:  ****:***** ********

A0A087TW26_Ca-ind_PLA2-gamma_(Fragment)      ------------------------------------------------------------      506
PhospholipaseA2-15-Pverdolaga                TEAVHISMNDLLSPGTYFRFNPYLTEFLHLDENRPDKLHQLKMDAQMYLRRNEHKLEQSI      588

A0A087TW26_Ca-ind_PLA2-gamma_(Fragment)      -------------------------  506
PhospholipaseA2-15-Pverdolaga                KVLTRPRSTLKKINDWIQLQKTLLL  613
```

PhospholipaseA$_2$-16-Pverdolaga:

MAEEEYHLMCSILAHGSDIRSVTTSYVPLGGIVTGSRDKTIKLWRPTGTTFTEEHCMRGASHFIS
SLCALPPSDQYPDGLILAGSNDCAIYGFSLDSSEPILKLLGHSENVCALVAGNLGTIVSGSWDKT
ARVWHGQRCVATLSGHTQAVWAVALLPDHALVLTGSADKAVFLWNNGKCERKFIGHEDC
VRGLTVISDLEFLSCSNDTTVRRWQTSGECLGIYTGHTDYVYDICLSSCREYFISCSEDQTVKVW
KENVCVQTIKLPAKSLWAVTYLYNGDIAVGGSDGSVRVFTKDKSRRASPAEEARFNEEIVSMN
SKNMKQNIGDLELDDVPGPDALLQDGTSDGQTQLCKVGNEVSVFQWSVKEHKWLKLGKVL
DALDNRPAKGKTVYEGKEYDYVFTIDVAEGKLLKLPYNDTEDPWLVAHKFIEKHDLNPMFLD
QIANFIINNSKSAGVQAESMSEFSDPFTGASRYIPSNVGKPSSLASNHGDNSSIQELPKSNPTGN
GDIEKASTGAHFPLLTYVTFDTANTNGIRAKLCEFTEKIEKSQQLSIEKIEHMLLLLDYPQAITD
DQMLSLEKALSWPAEFVFPALDVLRLAVRAEPVNSRVSKDGGVGLINHLLRYVSTGNPVSNQ
MLVLRTLSNFFVCPSGEQLLVSQAKKVLSLTRSCCASKNKHVQIALATLYANYSVAFQKSTSSE
DTYCKDMYLNDAVEALKQFNEPEALFRLIVCIGTAVQDKYCLQVAKALKIGEIVQSVLERCEV
SKIQDFGATLIDIVSN

```
CLUSTAL O(1.2.4) multiple sequence alignment


EDL30937.1_PLA2_isoform         MASGASRYRLSCSLPGHELDVRGLVCCLYPPGAFVSVSRDRTTRLWAPDSPNRGFTEMHC  60
PhospolipaseA2-16-Pverdolaga    --MAEEEYHLMCSILAHGSDIRSVTTSYVPLGGIVTGSRDKTIKLWRPTG--TTFTEEHC  56
                                   . ..*:* **: .*  *:*.:. .   * *.:*: ***:* :** * .     *** **

EDL30937.1_PLA2_isoform         MSGHSNFVSCVCIIPSSDIYPHGLIATGGNDHNICIFSLDSPMPLYILKGHKDTVCSLSS  120
PhospolipaseA2-16-Pverdolaga    MRGASHFISSLCALPPSDQYPDGLILAGSNDCAIYGFSLDSSEPILKLLGHSENVCALVA  116
                                * * *:*:*.:* :* ** **.*** :*.**  *  *****  *:  * **.:.**:* :

EDL30937.1_PLA2_isoform         GKFGTLLSGSWDTTAKVWLNDKCMMTLQGHTAAVWAVKILPEQGLMLTGSADKTIKLWKA  180
PhospolipaseA2-16-Pverdolaga    GNLGTIVSGSWDKTARVWHGQRCVATLSGHTQAVWAVALLPDHALVLTGSADKAVFLWNN  176
                                *::**::*****.**:**  .::*: **.*** *****  :**::.*:*******:: **:

EDL30937.1_PLA2_isoform         GRCERTFLGHEDCVRGLAILSETEFLSCANDASIRRWQITGECLEVYFGHTNYIYSISVF  240
PhospolipaseA2-16-Pverdolaga    GKCERKFIGHEDCVRGLTVISDLEFLSCSNDTTVRRWQTSGECLGIYTGHTDYVYDICLS  236
                                *:***.*:*********:::*: *****:**:::****  :****  :* ***:*:*.*.:

EDL30937.1_PLA2_isoform         PNSKDFVTTAEDRSLRIWKHGECAQTIRLPAQSIWCCCVLENGDIVVGASDGIIRVFTES  300
PhospolipaseA2-16-Pverdolaga    SCREYFISCSEDQTVKVWKENVCVQTIKLPAKSLWAVTYLYNGDIAVGGSDGSVRVFTKD  296
                                   : *:: :**:::::**.. *.***:***:*:*.    * ****.**.*** :****:.

EDL30937.1_PLA2_isoform         EERTASAEEIKAFERELSQATIDSKTGDLGDINAEQLPGREHLSEPGTREGQTRLIRDGE  360
PhospolipaseA2-16-Pverdolaga    KSRRASPAEEARFNEEIVSMNSKNMKQNIGDLELDDVPGPDALLQDGTSDGQTQLCKVGN  356
                                :.* **  *   *:.*: . . .. . ::**:: :::** : * : ** :***:* : *:

EDL30937.1_PLA2_isoform         RVEAYQWSVSDGRWIKIGDVVGSSGANQQTSGKVLYEGKEFDYVFSIDVNEGGPSYKLPY  420
PhospolipaseA2-16-Pverdolaga    EVSVFQWSVKEHKWLKLGKVLDAL-DNRPAKGKTVYEGKEYDYVFTIDVAEG-KLLKLPY  414
                                .*..:****.: :*:*:*.*:.:   *: :.**.:*****:****:*** **    ****

EDL30937.1_PLA2_isoform         NVSDDPWLVAYNFLQKNDLNPMFLDQVAKFIIDNTKGQTLG-LGNTSFSDPFTGAGRYMP  479
PhospolipaseA2-16-Pverdolaga    NDTEDPWLVAHKFIEKHDLNPMFLDQIANFIINNSKSAGVQAESMSEFSDPFTGASRYIP  474
                                *  ::******::*::*:*********.*:***:*:*.   :    . :.********.**:*

EDL30937.1_PLA2_isoform         GSAGMDTTMTGVDPFT---------GNSAYRSAASKTVNIYFPKKEALTFDQANPTQILG  530
PhospolipaseA2-16-Pverdolaga    SNVGKPSSLASNHGDNSSIQELPKSNPTGNGDIEKASTGAHFPLLTYVTFDTANTNGIRA  534
                                ...*  ::::. .  .          . :.  .  . :.. :**     :*** **  . * .

EDL30937.1_PLA2_isoform         KLKELNGTAPEEKKLTEDDLVLLEKILSLICNNSSEKPTAQQLQILWKAINWPEDIVFPA  590
PhospolipaseA2-16-Pverdolaga    KLCEFTEKIEKSQQLSIEKI---EHMLL--LLDYPQAITDDQMLSLEKALSWPAEFVFPA  589
                                ** *:. .  :.::*: :.:   *::*      :  :  * :*:  * **:.** ::****

EDL30937.1_PLA2_isoform         LDILRLSIKHPNVNENFCNEKGDQFSSHLINLLNPKGKPANQLLALRTFCNCFVSQAGQK  650
PhospolipaseA2-16-Pverdolaga    LDVLRLAVRAEPVNSRVSKDGGVGLINHLLRYVSTGNPVSNQMLVLRTLSNFFVCPSGEQ  649
                                **:***:::   **....:: *  : .**:. :.  .   :**:*.***:.* **. :*::

EDL30937.1_PLA2_isoform         LMMSQRESLMSHAIELKSGSNKNIHIALATLTLNYSVCFHKDHNIEGKAQ---CLSVIST  707
PhospolipaseA2-16-Pverdolaga    LLVSQAKKVLSLTRSCCASKNKHVQIALATLYANYSVAFQKSTSSEDTYCKDMYLNDAVE  709
                                *::**  :.::* : .  :..**:::******  ****.*:*. . *..       *.

EDL30937.1_PLA2_isoform         ILEVVQDLEATFRLLVALGTLISDDSNAIQLAKSLGVDSQIKKYVSVSEPAKVSECCRLV  767
PhospolipaseA2-16-Pverdolaga    ALKQFNEPEALFRLIVCIGTAVQDK-YCLQVAKALKIGEIVQSVLERCEVSKIQDFGATL  768
                                 *: .:: ** ***:*.:** :.*.  .:*:**:* :.. ::. :. .* :*:.:    :

EDL30937.1_PLA2_isoform         LHLL--       771
PhospolipaseA2-16-Pverdolaga    IDIVSN       774
                                :.::
```

Pairwise alignments and amino acid sequences of phospholipases D translated from *Pamphobeteus verdolaga*. Residues highlighted in grey indicate the signal peptide, while magenta blue highlighted residues, indicates the propeptide according to Spider|ProHMM from the arachnoserver. Residues highlighted in yellow shows cysteines potentially forming disulfide bridges. Residues highlighted in red shows amino acids implicated in the active site.

PhospholipaseD-1-Pverdolaga:

MKILKFLGCLIWYQVCVADEVDWRRPVWNIAHMVNANYQIDYYLDMGANSIEFDVAFDNS
GNARFTFHGVPCDCFRSCVRHEEIENYLEYMRHLTTPGDPKFQEKLVLLFMDLKVKGLSSRAR
TNAGFSIARKLVRHYWQNGTSAARAHVLMSIPSVDHMEVVRGFRDGLRVEGLSGYINKVGV
DFSGNEDLNSIRRALMSESISDRIWQGDGITNCLPRGTGRLREAIQRRDQPGLTHIEKVYWWTV
DKMSTMRATLRLSVDAMITNYPERLVSVLDEDEFSGRFRMATIDDNPWSKHELRTSALYALDE
GPTARGGNITTYFDKEDDELLIIASTITQTMAGINFTRGLEESIPX

```
CLUSTAL O(1.2.4) multiple sequence alignment


Q1W694_LiSicTox-betaID1         MQLFIILCLAGSAVQLEGTELDGVERADNRRPIWNIAHMVNDKGLIDEYLDDGANSVESD    60
PhospolipaseD-1-Pverdolaga      ---MKILKFLGCLIWY---QVCVADEVDWRRPVWNIAHMVNANYQIDYYLDMGANSIEFD    54
                                   : ** : *. :     ::  .:..* ***:******** :  ** *** ****:* *

Q1W694_LiSicTox-betaID1         VSFDSNGKPEKMLHGSPCDCGRSCKRQMSFADYLDYMRQLTTPGDPKFRENLILVMLDLK    120
PhospolipaseD-1-Pverdolaga      VAFDNSGNARFTFHGVPCDCFRSCVRHEEIENYLEYMRHLTTPGDPKFQEKLVLLFMDLK    114
                                *:**..*: .  :** **** *** *: .: :**:***:*********:*:*:*:::***

Q1W694_LiSicTox-betaID1         LKKLSSEQAYSAGQEVASQMLDKYWKRGESGARAYIVLSIPTITRVTFVNGFYDKLHSEG    180
PhospolipaseD-1-Pverdolaga      VKGLSSRARTNAGFSIARKLVRHYWQNGTSAARAHVLMSIPSVDHMEVVRGFRDGLRVEG    174
                                :* ***.   .** .:* ::: :**:.* *.***::::***:: :: .*.** * *: **

Q1W694_LiSicTox-betaID1         FDQYREKVGVDFSGNEDLEDTGKILKSRDILDHIWQSDGITNCLFRIMKRLKAAIRKRDS    240
PhospolipaseD-1-Pverdolaga      LSGYINKVGVDFSGNEDLNSIRRALMSESISDRIWQGDGITNCLPRGTGRLREAIQRRDQ    234
                                :. * :************:.  : * *..* *:***.******* *   **: **::**.

Q1W694_LiSicTox-betaID1         NGY--MVKVYTWSVDKYTTMRKALRAGADGMITNFPKRLVSVLNEREFSGKFRLATYNDN    298
PhospolipaseD-1-Pverdolaga      PGLTHIEKVYWWTVDKMSTMRATLRLSVDAMITNYPERLVSVLDEDEFSGRFRMATIDDN    294
                                 *   : *** *:*** :*** :** ..*.****:*:******:* ****:**:** :**

Q1W694_LiSicTox-betaID1         PWERYTG-----------------------------------------------------    305
PhospolipaseD-1-Pverdolaga      PWSKHELRTSALYALDEGPTARGGNITTYFDKEDDELLIIASTITQTMAGINFTRGLEES    354
                                **.::

Q1W694_LiSicTox-betaID1         ---      305
PhospolipaseD-1-Pverdolaga      IPX      357
```

PhospholipaseD-2-Pverdolaga:

MANDISDAIYLLDQGANALEFDISFFNNGTVNRVYHGVPCDCFRVCTHEASLPDYLSTIRKITD
PQTGKYSQQMTFQFFDLKLQEVTPWGKYVAGLEIANHVIDYLWGNDTKRQLVRVLIFINDES
DKDVVLGVRNAFLQRGMKKFLDQVGFDGGTGTMKSIRDMWDSLGIRGNLWQGDGIFNCLS
EVYKDDRLREALHIRDSPNGFIDKVYHWTIDSRGRMRMSLRLGVDGMITNLPKDLIDVLNEDP
YSNIFRLATAKDDPFSRFHPSKSFK

```
CLUSTAL O(1.2.4) multiple sequence alignment


C0JB54_StSicTox-betaIF1_(Fragment)      WIMGHMVNDLEMVDYYVDKGANGLEIDITFNSNGIAEYTYHGVPCDCFRNCRRNTTLSTY    60
PhospolipaseD-2-Pverdolaga              -----MANDISDAIYLLDQGANALEFDISFFNNGTVNRVYHGVPCDCFRVCTHEASLPDY    55
                                             *.**:. . * :*:***.**:**:* .** .: .********** * ::::*  *

C0JB54_StSicTox-betaIF1_(Fragment)      LNYVRQLTTPGDQKFRQNLIFIIMDLKLNRLKSQALFNAGLSIADRLTQYYWKDDGKARA    120
PhospolipaseD-2-Pverdolaga              LSTIRKITDPQTGKYSQQMTFQFFDLKLQEVTPWGKYVAGLEIANHVIDYLWGNDTKRQL    115
                                        *. :*::* *   *: *:: * ::****:.:.  . : ***.**::: :* * :* * :

C0JB54_StSicTox-betaIF1_(Fragment)      YFLL-SVPYVRQAAFIRGFQSRFEEKGLKKYYEKIGWDFSANEDLNRIREAYQKLNISGH    179
PhospolipaseD-2-Pverdolaga              VRVLIFINDESDKDVVLGVRNAFLQRGMKKFLDQVGFDGGT-GTMKSIRDMWDSLGIRGN    174
                                          :*  :    :  .: *.:. * ::*:**: :::*:* .:   :: **: ::.*.* *:

C0JB54_StSicTox-betaIF1_(Fragment)      IWQSDGITNCLTRR--TRRLKEAIRKRDSPGWYINKVYSWSLDRYKSIKYALDLGVDGVM    237
PhospolipaseD-2-Pverdolaga              LWQGDGIFNCLSEVYKDDRLREALHIRDSPNGFIDKVYHWTIDSRGRMRMSLRLGVDGMI    234
                                        :**.*** ***:.     **:**:: ****. :*:*** *::*    :: :* *****::

C0JB54_StSicTox-betaIF1_(Fragment)      SNYADRLVKILSKGTYKRRFRLATHEDNPWETFTP-----       272
PhospolipaseD-2-Pverdolaga              TNLPKDLIDVLNEDPYSNIFRLATAKDDPFSRFHPSKSFK       274
                                        :*  . *:.:*.:. *.. ***** :*:*:. * *
```

PhospholipaseD-3-Pverdolaga:
EKRSGGYAVTSFVKSFRGIFTECCGNWKTRWLLVKDNFVAYIKPSDGQLKCVLLMDHDFSVK
SGKAETGKSNSLFISNMSRHLRLKCRSERQATEWAAEIERVVEKSGFEFTKVSRHGSFAPPRPH
SPCRWIIDGATYFDSVASALDRAKEEIFIADWWLTPEIYLKRPTFHGHYWQLDHILK

```
CLUSTAL O(1.2.4) multiple sequence alignment


KFM64830.1_Phospholipase_D1_(partial)     -----------------------------------------MKPKDGRVRCILLMDQGFK 19
PhospolipaseD-3-Pverdolaga                EKRSGGYAVTSFVKSFRGIFTECCGNWKTRWLLVKDNFVAYIKPSDGQLKCVLLMDHDFS 60
                                                                                 :**.**:::*:****:.*.

KFM64830.1_Phospholipase_D1_(partial)     VDCGFVTTGIHHGLQISNLTRRLLVRCWTRRKAREWTECLVDTAKTTGRDFTQPNRYGAF 79
PhospolipaseD-3-Pverdolaga                VKSGKAETGKSNSLFISNMSRHLRLKCRSERQATEWAAEIERVVEKSGFEFTKVSRHGSF 120
                                          *..* . **  :.* ***::*:* ::* :.*:* **:  :   ..:.:* :**: .*:*:*

KFM64830.1_Phospholipase_D1_(partial)     APVRINNECRWFVDGATYFEAVADALEKAKEEIFIADWWLSPELYMKRPVIQGEIWRLDH 139
PhospolipaseD-3-Pverdolaga                APPRPHSPCRWIIDGATYFDSVASALDRAKEEIFIADWWLTPEIYLKRPTFHGHYWQLDH 180
                                          ** * :. ***::******::**.**::************:**:*:***.::*. *:***

KFM64830.1_Phospholipase_D1_(partial)     ILRRKA      145
PhospolipaseD-3-Pverdolaga                ILK---      183
                                          **:
```

PhospholipaseD-4-Pverdolaga:
XALPISKLDIKRHTSSIVPPPLFPVHKRSRSDSNVMTVPKKNQEFYQSRPHDDYHIKTLLKNIPS
KPQQPVHKLQAKVKHDENRAKQRWRVAVKKIQAISAFQNLESQVLLEQVRDGAHQDVCLS
SLPTPADVHRTIQEIALVHLGLERTYRLWHGKDYSNFIFKDLNKLNEPYTDSVNRYETPRMP
WHDVSCFLQGPAARDVARHFIQRWNFTKLRTAKFDDVYPLLLPKCYEFPDPIPPILSSEVGSIL
MADCQVLRSTSMWSAGIITTEYSILNAYKDAIMKAEHFIYIENQFFVSLQHGNKDVFNDISEC
LYQRIMKAHQENKRFCVYVIMPLLPAFEGEVGTGTGTLIQAVTHWNYSSICRGPRSLCQRLA
KSIQDPLSYISFYGLRNFGVLNNKLVTELIYVHSKLMIVDDKKAIIGSANINDRSLLGRRDSEIA
VLVNDSVFVESVMDGKPYKAGHFCSSLRKALFKEHLGLLGEKHSKVEX

```
CLUSTAL O(1.2.4) multiple sequence alignment


XP_003744259.1_Phospholipase      MDLRRENSDGSEDVEEFEELELEDDEEDENLRNSPLDTDCGIATHLSTHFGSIPFARIYD  60
PhospholipaseD-4-Pverdolaga       ------------------------------------------------------------  0


XP_003744259.1_Phospholipase      SSQSVTDSSDGFFVPGVPMCFKLLGFERDPRPSHMVHPNLYVIEVTHGDFVWVLKKRYKH  120
PhospholipaseD-4-Pverdolaga       ------------------------------------------------------------  0


XP_003744259.1_Phospholipase      FLELHTILKLFRASLALPLPNESHQMRRKSFFDDRAKRKEVPQFPKRPESILSSAQVQAR  180
PhospholipaseD-4-Pverdolaga       ------------------------------------------------------------  0


XP_003744259.1_Phospholipase      GEQLVSYLNKVVKVANYRNHPKMTEFLEVSPYSFLNSTGPKGKEGAVSKRAGGHNPNSCF  240
PhospholipaseD-4-Pverdolaga       ------------------------------------------------------------  0


XP_003744259.1_Phospholipase      IYMKSVWYKICKRWRRRWLIAKDTYILYIHPKTGAVKSVILMDGRFRVHEDGRPTGSRSG  300
PhospholipaseD-4-Pverdolaga       ------------------------------------------------------------  0


XP_003744259.1_Phospholipase      VIISNLSRELLIDVWTKRQRMEWVKHIEELVKTRAKDFVAENRHGSYAPVRSRTLARWYV  360
PhospholipaseD-4-Pverdolaga       ------------------------------------------------------------  0


XP_003744259.1_Phospholipase      DGAMYMRAVANAIENAREEIFITDWWLSPELFLKRSHNKNNEDWRLDLLLRRKASSGVRI  420
PhospholipaseD-4-Pverdolaga       ------------------------------------------------------------  0

XP_003744259.1_Phospholipase      FIMLYKEVSSALAINSLYSKQKISTHPNIKVFRHPDHVNAAVYLWAHHEKLVIVDQKYAF  480
PhospholipaseD-4-Pverdolaga       ---------XALPISKLDIK------------RHTSSIV---------------------  18
                                            ** *..*  *            ** . :

XP_003744259.1_Phospholipase      LGGIDLCYGRWDDAGHRLTDLVPDENKRQGVCDESDVDEVDGYMVKPSCCVSETRFLNVA  540
PhospholipaseD-4-Pverdolaga       -----------------PPPLFPVHKRSRS---DSNV------MTVPK---KNQEFY-QS  48
                                                      *.* .:: :.   :*:*      *. *.   .: .*   :

XP_003744259.1_Phospholipase      GPPHNFERCLV---VPEYFDQAEGKIVN-SKSPSDRVSRRLMLQRVKAKLKALRAFKHLH  596
PhospholipaseD-4-Pverdolaga       RPHDDYHIKTLLKNIPSKPQQPVHKLQAKVKHDENRAKQRW--RVAVKKIQAISAFQNLE  106
                                   * .::.   :   :*.  :*   *:    *  .:*..:*   : .  *::*: **::*.

XP_003744259.1_Phospholipase      YLEGSTAEIMYEDPHSGYLNSQFECLE-------------E-EKLEPFVTDGTKYWIGKD  642
PhospholipaseD-4-Pverdolaga       ------SQVLLEQVRDGAH--QDVCLSSLPTPADVHRTIQEIALVHLGLERTYRLWHGKD  158
                                        :::: *: :.*    *  **.             *   :.  :    : * ***

XP_003744259.1_Phospholipase      YSNFIYKDVTNLEKPFDDHIDRTKIPRMPWHDIGAVVLGRAARDLARHFIQRWNSIKIQK  702
PhospholipaseD-4-Pverdolaga       YSNFIFKDLNKLNEPYTDSVNRYETPRMPWHDVSCFLQGPAARDVARHFIQRWNFTKLRT  218
                                  *****:**:.:*::*: * ::* : *******:...: * ****:*********  *::.

XP_003744259.1_Phospholipase      AKKAKSYPWLIPKSYDSAEDIEV---KLFGKLYRCEVQILRSASSWSAGISETENSIHSA  759
PhospholipaseD-4-Pverdolaga       AKFDDVYPLLLPKCYEFPDPIPPILSSEVGSILMADCQVLRSTSMWSAGIITTEYSILNA  278
                                  **  . ** *:**.*:  : *     . .*.:  .: *:***:* *****  ** ** .*

XP_003744259.1_Phospholipase      YVDLILKAKHFIYIENQFFISLQSPDNIVYNEVADALFTRIVGAFKAKEKFRVYIVLPLL  819
PhospholipaseD-4-Pverdolaga       YKDAIMKAEHFIYIENQFFVSLQHGNKDVFNDISECLYQRIMKAHQENKRFCVYVIMPLL  338
                                  * * *:**:**********:***  :: *:*::::.*: **: *.: :::* **:::***

XP_003744259.1_Phospholipase      PAFEGEVGTPSGVSIQAITHWNYNSLCRGEYSLLERLKREVDDPKEYIGFYGLRKHDMLN  879
PhospholipaseD-4-Pverdolaga       PAFEGEVGTGTGTLIQAVTHWNYSSICRGPRSLCQRLAKSIQDPLSYISFYGLRNFGVLN  398
                                  ********* :*. ***:*****.*:***  ** :** :.::** .**.*****:..:**
```

```
XP_003744259.1_Phospholipase     GIPVTELIYVHSKLMIVDDVQAIIGSANINDRSLLGERDSEIAKVVSDQHFLRATFDGKP   939
PhospolipaseD-4-Pverdolaga       NKLVTELIYVHSKLMIVDDKKAIIGSANINDRSLLGRRDSEIAVLVNDSVFVESVMDGKP   458
                                 .  **************** :**************.****** :*.*.  *:.:.:****

XP_003744259.1_Phospholipase     VKAGLYVSSLRRWIFREHLGIRDVEDPVADSFYSDVWKKIARRNTEIFEEVFKPLPTDCV   999
PhospolipaseD-4-Pverdolaga       YKAGHFCSSLRKALFKEHLGLLGEKHSKVEX-----------------------------   489
                                  *** : ****: :*:****: . :.  .:

XP_003744259.1_Phospholipase     KTFAELRAYSRVSPLAIEDPSAALEKLRDVQGHLVELPERFLEDENLTPAPTTKESLMPT   1059
PhospolipaseD-4-Pverdolaga       ------------------------------------------------------------   489

XP_003744259.1_Phospholipase     RLWT   1063
PhospolipaseD-4-Pverdolaga       ----   489
```

Pairwise alignments and amino acid sequences of phospholipases B translated from *Pamphobeteus verdolaga*. Residues highlighted in grey indicate the signal peptide, while magenta blue highlighted residues, indicates the propeptide according to Spider|ProHMM from the arachnoserver. Residues highlighted in yellow shows cysteines potentially forming disulfide bridges.

PhospholipaseB-1-Pverdolaga:
LEAIVLSLFRDNVVFLYGVFAPPRLVSASCSSVSLCMKNLSPEASDWKFCMMLTLWVAIALLV
HVCAASTEQKAYVTWDSSSYKFTVHSDPVENFVAYATFTNEINATGWSYLEVWTNETFPDSV
QAYSAGLAEGVLTADLLKKHWYNTVATYCDGEESYCDRLKIFLETNLDFMNYNIARRRKYV
PYWHQVALALEQLSGLEDGYNNVSGKPHTKLNVTGVLMVNIFGDLEDLEGILNKTVSSRPL
GSGSCSGLIKVLPNNEDLYVSQDSWNTYSSMLRVLKKYNISVHSGMDRGSPVIPGQVMSFSSY
PGLICSGDDFYTISSGLATMETTIGNGNSSLWKYIRAKGTVLEWLRSIVANRMARSGREWSRW
FSIMNSGTYNNQWMVVDYNKFLPGAPLQNDLLWVLEQLPGYIHSDDLTDVLRKQGYWPSY
NTPYFKDIFNLSGSQENADKYGDWFTYDKTPRALIFKRDHGTVTDVKSMIKLMRYNDYTHD
PLSRCNCTPPYSAENAISARCDLNPANGTYPFGALGHRSHGGIDMKLTTGSLFKNFEFVAFG
GPTYDSLPPFKWSESDFRTTERHEGHPDLWKFEPIVRKWSQ

```
CLUSTAL O(1.2.4) multiple sequence alignment


XP_015925352.1_Phospholipase_B-like_Putative      --------------------------------------------------MLVSSVVLC   9
PhospolipaseB-1-Pverdolaga                        LEAIVLSLFRDNVVFLYGVFAPPRLVSASCSSVSLCMKNLSPEASDWKFCMMLTLWVAIA   60
                                                                                                  **.   *.:.

XP_015925352.1_Phospholipase_B-like_Putative      LL---IALVKAKNASVTYDQTTGKFQIHDYIANDPVAYGTFTDEIFKVGWSYLEIKSYEK   66
PhospolipaseB-1-Pverdolaga                        LLVHVCAASTEQKAYVTWDSSSYKFTVHSDPVENFVAYATFTNEINATGWSYLEVWTNET   120
                                                  **    *   . ::* **:*.:: ** :*.   .:: ***.***:**  .******: : *.

XP_015925352.1_Phospholipase_B-like_Putative      YPDPIQAYAAGVVEGYLTADLLRKHFSNLVDGYCDGEEIYCQRLTSFLQENWDFIDSNVK   126
PhospolipaseB-1-Pverdolaga                        FPDSVQAYSAGLAEGVLTADLLKKHWYNTVATYCDGEESYCDRLKIFLETNLDFMNYNIA   180
                                                  :** :***:**:.** ******:**: * *  ****** **:**. **: * **:: *:

XP_015925352.1_Phospholipase_B-like_Putative      VRRKYDVYWHQIALTFEQLHGLEDGYKNVTSQPSTKVDLMGLLLLNIMGDLEDLEVVLKK   186
PhospolipaseB-1-Pverdolaga                        RRRKYVPYWHQVALALEQLSGLEDGYNNVSGKPHTKLNVTGVLMVNIFGDLEDLEGILNK   240
                                                   ****  ****:**::*** ******:**:.:* **::: *:*::**:******* :*:*

XP_015925352.1_Phospholipase_B-like_Putative      NDMRRVLGSGHCSGLIKVLPNNKDILFSQVTWSTYTSMLRILKKYSLKLHTSLADGSPVI   246
PhospolipaseB-1-Pverdolaga                        TVSSRPLGSGSCSGLIKVLPNNEDLYVSQDSWNTYSSMLRVLKKYNISVHSGMDRGSPVI   300
                                                  .    * **** ***********:*:  .** :*.**:****:****.:.:*:.:  *****

XP_015925352.1_Phospholipase_B-like_Putative      PGHTSTFSSQPGVLYSGDDFYVLSSGLVAIETTFGNGNDSLWKYVVP-QTILEWQRNIIA   305
PhospolipaseB-1-Pverdolaga                        PGQVMSFSSYPGLICSGDDFYTISSGLATMETTIGNGNSSLWKYIRAKGTVLEWLRSIVA   360
                                                  **:. :*** **:: ******.:****.::***:****.*****:     *:*** *.*:*

XP_015925352.1_Phospholipase_B-like_Putative      NRLAKTGKQWVTLFGILNSGTYNNQWMVVDYNKFKPGSPLQDGLLYVLEQLPGYLHSEDR   365
PhospolipaseB-1-Pverdolaga                        NRMARSGREWSRWFSIMNSGTYNNQWMVVDYNKFLPGAPLQNDLLWVLEQLPGYIHSDDL   420
                                                  **:*:.*:.*    *.*:***************** **:***:.**:********:**:*
```

```
XP_015925352.1_Phospholipase_B-like_Putative        TDVLRNQGYWPSYNVAYFKDIFVMSGAQANADKYGDWFTYERNPRALIFQRDHKKVQDID  425
PhospolipaseB-1-Pverdolaga                          TDVLRKQGYWPSYNTPYFKDIFNLSGSQENADKYGDWFTYDKTPRALIFKRDHGTVTDVK  480
                                                    *****:********. ****** :**:* ***********::.******:*** .* *:.

XP_015925352.1_Phospholipase_B-like_Putative        SMIKLMRYNDYTNDPLSRCNCTPPYSAENAISARCDLNPVNGTYPFAALGHRQHGATDMK  485
PhospolipaseB-1-Pverdolaga                          SMIKLMRYNDYTHDPLSRCNCTPPYSAENAISARCDLNPANGTYPFGALGHRSHGGIDMK  540
                                                    ************:************************.******.*****.**. ***

XP_015925352.1_Phospholipase_B-like_Putative        LTSYELQKNYEFVAISGPTYDPLPPFQWSKSDFDKKVKHEGHPDLWQFKPIVHKWL-     541
PhospolipaseB-1-Pverdolaga                          LTTGSLFKNFEFVAFGGPTYDSLPPFKWSESDFRTTERHEGHPDLWKFEPIVRKWSQ     597
                                                    **:  .* **:****:.***** ****:**:***  .. :********:*:***:**
```

Pairwise alignments and amino acid sequences of kunitz-type serine protease inhibitors translated from *Pamphobeteus verdolaga*. Residues highlighted in grey indicate the signal peptide, while magenta blue highlighted residues, indicates the propeptide according to Spider|ProHMM from the arachnoserver. Residues highlighted in yellow shows cysteines potentially forming disulfide bridges.

Kunitz-1-Pverdolaga:
MGIRNICIVISVFTVLFVLTVPPVFSADEKMDICKLPPESCIIDLRAQLKIPRWYFNGRLCTRFVYSGCDASENNFISRAQCRAKCGQARRRIRVPKT

```
CLUSTAL O(1.2.4) multiple sequence alignment


B2ZBB6_Kunitz-type-theraphotoxin-Hs1b        MGIARILSAVLFLSVLFVVTFPALLSADHHDGRIDTCRLPSDRG----RCKASFERWYFN 56
Kunitz-1-Pverdolaga                          MGIRNICIVISVFTVLFVLTVPPVFSAD---EKMDICKLPPESCIIDLRAQLKIPRWYFN 57
                                             *** .*  .: .::****:*.* ::***    ::* *:** :       *.: .: *****

B2ZBB6_Kunitz-type-theraphotoxin-Hs1b        GRTCAKFIYGGCGGNGNKFPTQEACMKRCGKA---------    88
Kunitz-1-Pverdolaga                          GRLCTRFVYSGCDASENNFISRAQCRAKCGQARRRIRVPKT    98
                                             ** *::*:*.**... *:* ::  *  :**:*
```

Kunitz-2-Pverdolaga:
MGVRTVFLLFVAFSFAAGDLQNPIRDEVCSLKAEPGIPGKNIMCLAYFPKYYFNSAVGYCEKFIFGGCGGNANSFSTKEECEKFCGSDDKR

```
CLUSTAL O(1.2.4) multiple sequence alignment


W4VSH9_Kunitz-type_U19-barytoxin-Tl1a        MNFELIYVSSLLLGICLANQADVVPSDCNLPADAGMCYAYFPMFFYDASSRKCLNFIYGG 60
Kunitz-2-Pverdolaga                          MGVRTVF------------------------------LLFVAFSF------------- 15
                                             *... ::                              *  * :

W4VSH9_Kunitz-type_U19-barytoxin-Tl1a        CGGNANRFWSEAECMEKCGGGGGGGGSSDGSQKTAKMLNLDLGDICSLEK------KVGP 114
Kunitz-2-Pverdolaga                          --------------------------------AAGDLQNPIRDEVCSLKAEPGIPGKNIM 43
                                                                             :..: *   .::***:        *

W4VSH9_Kunitz-type_U19-barytoxin-Tl1a        CKAHMPRYYFNRETGLCEEFIYGGCSGNHNNFQTKEQCESFCAPGNSPRPEEETRKRTKQ 174
Kunitz-2-Pverdolaga                          CLAYFPKYYFNSAVGYCEKFIFGGCGGNANSFSTKEECEKFCGSDDKR------------ 91
                                             * *::*:****   .* **:**:***.** *.*.***:**.**. .:.

W4VSH9_Kunitz-type_U19-barytoxin-Tl1a        SY   176
Kunitz-2-Pverdolaga                          --   91
```

Kunitz-3-Pverdolaga:
MGIRNICSVISVFTVLFALTFPPVFLAGYHLDICRQRPDRGMCLVNMERWFFNGRFCSTFVYGGCGGNGNNFISKAQCMARCARG

```
CLUSTAL O(1.2.4) multiple sequence alignment


P0DJ76|VKT4_Kunitz-type-theraphotoxin-Hs1e      MGIARILSAVLFLSVLFVVTFPALLLADHHDGRIDTCRLPSDRGRCKASFERWYFNGRTC    60
Kunitz-3-Pverdolaga                             MGIRNICSVISVFTVLFALTFPPVFLAGYH---LDICRQRPDRGMCLVNMERWFFNGRFC    57
                                                ***  .* *.:  .:***.:***  ::**.:*    :* **    *** *  ..:***:**** *

P0DJ76|VKT4_Kunitz-type-theraphotoxin-Hs1e      AKFIYGGCGGNGNKFPTEKACMKRCAKA    88
Kunitz-3-Pverdolaga                             STFVYGGCGGNGNNFISKAQCMARCARG    85
                                                :.*:*********:*  ::  ** ***:.
```

Kunitz-4-Pverdolaga:

MLLMILVVASFQHSNADANGIDLCELDKEPGSCTSVINRYYFNRYSRRCERFIYTDCGGNSNNF HYEFECERTCPGDLYIGDVCSLPKKVGPCRAAMPRYYFNKETGRCETFTYGGCSGNYNNFETK EQCNSYCFQG

```
CLUSTAL O(1.2.4) multiple sequence alignment


W4VSH9_Kunitz-type_U19-barytoxin-Tl1a      MNFELIYVSSLLLGICLANQADVVPSDCNLPADAGMCYAYFPMFFYDASSRKCLNFIYGG 60
Kunitz-4-Pverdolaga                        MLLMILVVA----SFQHSNADANGIDLCELDKEPGSCTSVINRYYFNRYSRRCERFIYTD 56
                                           * : :: *:     .:  :*       . *:*  : * * : :  ::::  **:* .*** .

W4VSH9_Kunitz-type_U19-barytoxin-Tl1a      CGGNANRFWSEAECMEKCGGGGGGGGSSDGSQKTAKMLNLDLGDICSLEKKVGPCKAHMP 120
Kunitz-4-Pverdolaga                        CGGNSNNFHYEFECERTCPG-----------------DLYIGDVCSLPKKVGPCRAAMP 98
                                           ****:*.*  * **  ..* *                 :* :**:*** ******:* **

W4VSH9_Kunitz-type_U19-barytoxin-Tl1a      RYYFNRETGLCEEFIYGGCSGNHNNFQTKEQCESFCAPGNSPRPEEETRKRTKQSY     176
Kunitz-4-Pverdolaga                        RYYFNKETGRCETFTYGGCSGNYNNFETKEQCNSYCFQG----------------     137
                                           *****:*** ** * *******:***:*****:*:*  *
```

Kunitz-5-Pverdolaga:

XYFLRNGCPSSTVCETTHSLGISRGNCCDSINSCHLIAKFPNXAEMHFTLACVFSLFCGICFANH NVPDTDCSLPPDAGMCYAYFPMFFYDAPSGACINFIYGGCGGNANRFWTEEECMNRCAGVG GTTKEPADEEKGPIFLGPVDEGKGTISQKPIDEGKEVIIQKPVDEGKGAIYPQPTGGGKGVIFPQ PVAGGKGVIFQQPVGGGKGVTVQHAPQADICNQEKQPGNCSSQIIRYYFDKDSKKCDTFMYS GCGKNDNNFNYKFECERTCSGEHDIGDTCNFKQDSGPCRAFFPRFYFSGESGQCEQFIYGGCQ GNHNNFKTKEECLQFCTSGKGSPLP

```
CLUSTAL O(1.2.4) multiple sequence alignment


KFM65460.1_kunitz-type_serine_protease_inhibitor      MRALILLCAVTVAFASQDVCPENEHFEQCGTACPDNCENYRDTARPCVLMCISGCFCDKG      60
Kunitz-5-Pverdolaga                                   ------------------------------------------------------------      0


KFM65460.1_kunitz-type_serine_protease_inhibitor      FVRGEDGRCIKPESCPSQAEIVCPANQHFERCGTACPDT---CEN---YQ-DTLRPCVLM      113
Kunitz-5-Pverdolaga                                   -------XYFLRNGCPSST--VCETTHSLGISRGNCCDSINSCHLIAKFPNXAEMHFTLA      51
                                                             :  :.***.:  ** :.: :  .   * *:   *.    :   :    .*

KFM65460.1_kunitz-type_serine_protease_inhibitor      CVPGCFCDKGLVKAKDGSCIKPESCPSQAKPLVQNCEDKPERGMCLAYIPSYYYDKETGT      173
Kunitz-5-Pverdolaga                                   CVFSLFCG---------ICFAN------HNVPDTDCSLPPDAGMCYAYFPMFFYDAPSGA      96
                                                      ** . **.         *:         :    :*.  *: *** **:* ::**  :*:

KFM65460.1_kunitz-type_serine_protease_inhibitor      CKKFIYGGCQGNGNRYATEEECMEKCKGVTLAQTKSDDVCELPAVTGPCRALFHRYY---      230
Kunitz-5-Pverdolaga                                   CINFIYGGCGGNANRFWTEEECMNRCAGVGGTTKEPADEEKGPIFLGPVDEGKGTISQKP      156
                                                      * :****** **.**: ******::* **  : .:  *  : * . **

KFM65460.1_kunitz-type_serine_protease_inhibitor      --------FDSASGQCKKFIYGGCRGNENN--FKT--------LKECERTCGAGGVLGLA      272
Kunitz-5-Pverdolaga                                   IDEGKEVIIQKPVDEGKGAIYPQPTGGGKGVIFPQPVAGGKGVIFQQPVGGGKGVTVQHA      216
                                                              ::.  .: *  **    *. :.  *          : :     * * .:  *

KFM65460.1_kunitz-type_serine_protease_inhibitor      LDRPDCDKAPETGVCRAYIRRFYYDQKEGMCKTFIYGGCGGNRNNFVTEEECYNKCGALA      332
Kunitz-5-Pverdolaga                                   PQADICNQEKQPGNCSSQIIRYYFDKDSKKCDTFMYSGCGKNDNNFNYKFECERTCSGEH      276
                                                       :   *::  : * * : * *:*:*:..  *.**:*.*** * ***  : **  ..*..

KFM65460.1_kunitz-type_serine_protease_inhibitor      S-MSACQQEKETGPCKAAFRRFYYNKQTGECEPFIYGGCQGNSNNFLDKEDCEAVCKA--      389
Kunitz-5-Pverdolaga                                   DIGDTCNFKQDSGPCRAFFPRFYFSGESGQCEQFIYGGCQGNHNNFKTKEECLQFCTSGK      336
                                                      .  .:*: ::::***:* * ***:. ::*:** ********* ***  **:*  .*.:

KFM65460.1_kunitz-type_serine_protease_inhibitor      -----     389
Kunitz-5-Pverdolaga                                   GSPLP     341
```

Kunitz-6-Pverdolaga:
VISVFTVLLALTFPPLFSADHHLDICELPADSGTCFVRLHRWYFNGESCTKFLYRGCGGNENHF MTEVECMAKCGGA

```
CLUSTAL O(1.2.4) multiple sequence alignment


B2ZBB6_Kunitz-type-theraphotoxin-Hs1b    MGIARILSAVLFLSVLFVVTFPALLSADHHDGRIDTCRLPSDRGRCKASFERWYFNGRTC 60
Kunitz-6-Pverdolaga                      --------VISVFTVLLALTFPPLFSADHH---LDICELPADSGTCFVRLHRWYFNGESC 49
                                                 .:  .::**:.:*** *:*****   :* *.**:* * *  . :.******.:*

B2ZBB6_Kunitz-type-theraphotoxin-Hs1b    AKFIYGGCGGNGNKFPTQEACMKRCGKA 88
Kunitz-6-Pverdolaga                      TKFLYRGCGGNENHFMTEVECMAKCGGA 77
                                         :**:* ***** *:* *:   ** :** *
```

Kunitz-7-Pverdolaga:
XTSCKGIKESCTLDRAEAKEICMMPKEIGPCRGYFHRWYFDVNTLTCVTFVYGGCRGNNNNF EFQRDCVRTCEPLFKASGNEDPSNVSTHDVAQGNSPIDCMVTPWX

```
CLUSTAL O(1.2.4) multiple sequence alignment


XP_002435922.1_secreted_protein_Kunitz_domain_(Partial)     ----------------FPEICLLPKDIGPCRGYFPRWYYDSTKRMCLQFVYGGCRGNRN      43
Kunitz-7-Pverdolaga                                         XTSCKGIKESCTLDRAEAKEICMMPKEIGPCRGYFHRWYFDVNTLTCVTFVYGGCRGNNN      60
                                                                            ***::**:******** ***:*  ..  *: *********.*

XP_002435922.1_secreted_protein_Kunitz_domain_(Partial)     RFERYSECNKMCEVTISR---------------------------      61
Kunitz-7-Pverdolaga                                         NFEFQRDCVRTCEPLFKASGNEDPSNVSTHDVAQGNSPIDCMVTPWX      107
                                                            .**   :* : **  :.
```

Kunitz-8-Pverdolaga:
DAGTCFASIPRWYFTGSKCRSFIYGGCGGNANNFDTELXMPKEMWKEMKRVTSHFTTETGTA QNPSSGYLEVFEEVRIV

```
CLUSTAL O(1.2.4) multiple sequence alignment


B2ZBB6_Kunitz-type-theraphotoxin-Hs1b    MGIARILSAVLFLSVLFVVTFPALLSADHHDGRIDTCRLPSDRGRCKASFERWYFNGRTC 60
Kunitz-8-Pverdolaga                      ------------------------------------------DAGTCFASIPRWYFTGSKC 19
                                                                                   * * * **: ****.*  *

B2ZBB6_Kunitz-type-theraphotoxin-Hs1b    AKFIYGGCGGNGNKFPTQEACMKRCGKA-------------------------------- 88
Kunitz-8-Pverdolaga                      RSFIYGGCGGNANNFDTELXMPKEMWKEMKRVTSHFTTETGTAQNPSSGYLEVFEEVRIV 79
                                          .*********.*:* *:     *.   *
```

Kunitz-9-Pverdolaga:
QVATLHPPEAEKENLRNYVQRFPTGVVMKSSLGVCELEKNSGPCAKSYKRWYYDAISKDCLP FSYGGCLGNENRFRTKAACEETCKN

```
CLUSTAL O(1.2.4) multiple sequence alignment


XP_011135446.1_kunitz-type_serine_protease_inhibitor    ------------MGLKSCLLFTLIIVGILSHEIVAKPSSICQLPKVVGPCRASLKRYRYD  48
Kunitz-9-Pverdolaga                                     QVATLHPPEAEKENLRN------YVQRFPTGVVMKSSLGVCELEKNSGPCAKSYKRWYYD  54
                                                                    .*:.        :  : :  :: .  .:*:* *  ***  * **: **

XP_011135446.1_kunitz-type_serine_protease_inhibitor    STTGQCEEFTYGGCKGNENNFITREVCQENCINN    82
Kunitz-9-Pverdolaga                                     AISKDCLPFSYGGCLGNENRFRTKAACEETCKN-    87
                                                        : : :*  *:**** ****.* *: .*:*.* *
```

Kunitz-10-Pverdolaga:
XLKLPPKMMKLLWVNLLLLVLATCLCSEKTDKTNNGICNQRMDSGNGNQRITHFYYDTGRQ KCHPFPYSGRGGNKNNFSTMQECKKRMP

```
CLUSTAL O(1.2.4) multiple sequence alignment


XP_012273912.1_kappaPI-actitoxin-Avd3d      ----------MIANFVLPLVLLACLCAAAPSKTAPTVCELPMVKGNCRALMPRYRYDSRA      50
Kunitz-10-Pverdolaga                        XLKLPPKMMKLLWVNLLLLVLATCLCSEKTDKTNNGICNQRMDSGNGNQRITHFYYDTGR      60
                                                      ::   :* *** :***:    .**    :*:  *  .**  .   : :: **:

XP_012273912.1_kappaPI-actitoxin-Avd3d      KDCLPFNYGGCGGNRNNFLSREQCMETCKGV      81
Kunitz-10-Pverdolaga                        QKCHPFPYSGRGGNKNNFSTMQECKKRMP--      89
                                            :.* ** *.* ***:*** : ::* :
```

Kunitz-11-Pverdolaga: MRKSIMGTQVQGFRSSSPGALMSRVMASTTLCFFSGKVYLQA

```
CLUSTAL O(1.2.4) multiple sequence alignment


B2ZBB6_Kunitz-type-theraphotoxin-Hs1b      -----MG-----IARILSAVLFLSVLFVVTFPALLSADHHDGRIDTCRLPSDRGRCKASF 50
Kunitz-11-Pverdolaga                       MRKSIMGTQVQGFRSSSPGALM-------------------------------SRVMAST 29
                                                **     :     ..*:                                .*  **

B2ZBB6_Kunitz-type-theraphotoxin-Hs1b      ERWYFNGRTCAKFIYGGCGGNGNKFPTQEACMKRCGKA      88
Kunitz-11-Pverdolaga                       TLCFFSGKVYLQA--------------------------      42
                                              :*.*:.  :
```

Kunitz-12-Pverdolaga:
MLLRNDVSRRRIMIKVSRQRFSLLTFDLEDFCTAKDENNTDCQTLPAGDPTCYYDNCPMFYYDTSSSTCMNVEYGGCGGKANRFWTEKECTDQCKNKEDDVANGAASEYSGIVSWLKVKYSPFL

```
CLUSTAL O(1.2.4) multiple sequence alignment


W4VSH9_Kunitz-type_U19-barytoxin-Tl1a      ------------------MNFELIYVSSLLLGICLANQADVVPSDC-NLPAD-AGMCYAY 40
Kunitz-12-Pverdolaga                       MLLRNDVSRRRIMIKVSRQRFSLL--TFDLEDFCTAKDEN--NTDCQTLPAGDPTCYYDN 56
                                                              .*.*:  :  *  .:* *:: :   :** .***.     *

W4VSH9_Kunitz-type_U19-barytoxin-Tl1a      FPMFFYDASSRKCLNFIYGGCGGNANRFWSEAECMEKCGGGGGGGGSSDGSQKTAKMLNL 100
Kunitz-12-Pverdolaga                       CPMFYYDTSSSTCMNVEYGGCGGKANRFWTEKECTDQCKNKEDDVANGAASEYSGIV--- 113
                                            ***:**:**  .*:*. ******:*****:* ** ::*  .  .. ... .*: :. :

W4VSH9_Kunitz-type_U19-barytoxin-Tl1a      DLGDICSLEKKVGPCKAHMPRYYFNRETGLCEEFIYGGCSGNHNNFQTKEQCESFCAPGN 160
Kunitz-12-Pverdolaga                       -----SWLKVKYSPFL-------------------------------------------- 124
                                                . *: *  .*

W4VSH9_Kunitz-type_U19-barytoxin-Tl1a      SPRPEEETRKRTKQSY      176
Kunitz-12-Pverdolaga                       ----------------      124
```

Kunitz-13-Pverdolaga:
MGIARIFSVVSLFSVFLALTFPPLFSADHHEGTDICYLPPERGVCKAYSEQWHFNGRRCAKFVFGGCGGNANRFPTKDECIRRCRKA

```
CLUSTAL O(1.2.4) multiple sequence alignment


P68425_Kunitz-type-theraphotoxin-Hs1a      MGIARILSAVLFLSVLFVVTFPALLSADHHDGRIDTCRLPSDRGRCKASFERWYFNGRTC 60
Kunitz-13-Pverdolaga                       MGIARIFSVVSLFSVFLALTFPPLFSADHHEG-TDICYLPPERGVCKAYSEQWHFNGRRC 59
                                           ******:*.* ::**::.:*** *:*****:*  * * ** :** ***  *:*:**** *

P68425_Kunitz-type-theraphotoxin-Hs1a      AKFIYGGCGGNGNKFPTQEACMKRCAKA 88
Kunitz-13-Pverdolaga                       AKFVFGGCGGNANRFPTKDECIRRCRKA 87
                                           ***::******.*:***:: *::** **
```

Amino acid sequences of Hyaluronidases translated from *Pamphobeteus verdolaga*. Residues highlighted in grey indicate the signal peptide, while magenta blue highlighted residues, indicates the propeptide according to Spider|ProHMM from the arachnoserver. Yellow highlighted residues indicate

the cysteine position on hyaluronidase-like sequence (Cys17, Cys176, Cys183, Cys196, Cys218, Cys307, Cys332, Cys337, Cys343, Cys372, Cys374 and Cys383).

Hyaluronidase-1-Pverdolaga:
PRMYQSKLLQGLFPNSKDLPDSVLLLDESEGAVKVIGHSRLSRVLEDNRGCWIASVIISKEKRG
QGLGKFLMMKTEEYAKVLGLTTAYLNTRDKQGFYEHLGYSYCNPVSPHKGSFSMNGVGHLS
NFHRQVLRRCEEGETNHSGSPVGDPKIAKSSSVSSAKTSTPPLPPPPPPPPSSSNVKTDCFTSATG
HNWMKKYL

Hyaluronidase-2-Pverdolaga:
MSVTLFLLLLLPCYTRQAEDPTVFTVRWNVPTIQCRKTYGMDFVPLLKSYGILVNSGDEFKGEV
NTIFYESQLGLYPHLDQSGQRVNGGIPQLGDLPEHLKNAREDINKAIPDINFNGLGIIDWESWR
PVWNFNWGALKKYQDESFQEALKQHPGWTNDSLWQLAQQEWETSAKNFMLETLRLAQTM
RPNSLWCYYLFPDCYNYNGQTPREFRCPSIVVTGNNQLSWLWHESKAVCPSLYVADGYLQKY
TFEQRTWYVDGRLKEALRVAPNSQLYPYIGYGYGVTPGAMVPEDDFWRILAQVASAGSSGTVI
WGASATLRSKDNCQLLQKYVKDILGPSVTIVKENAERCAKTMCNGKGRCTWLNDPNVIAWR
VYLDRNKHPFQRSEITCHCVEGYSGRYCDVQRRVINQTKLRVSFKLSDLYTYLRRLLDN

Amino acid sequences of Lycotoxin translated from *Pamphobeteus verdolaga*. Residues highlighted in grey indicate the signal peptide, while magenta blue highlighted residues, indicates the propeptide according to Spider|ProHMM from the arachnoserver. Yellow highlighted residues indicate the cysteine disposition commonly described in the ICK peptides.

Lycotoxin-1-Pverdolaga:
MEGRTKLLFLIAVFSIMHVITAELYCPQRLRIYCYVRTDRCCSDSDCGGGTICCEENCGNTCRTP
RIIQSGGTRVDPSQICKIG

Lycotoxin-2-Pverdolaga:
MDRLLLMLFVTLAVLAQVLIVSPFMATSDADREILADYPDVTRYLYYNRKRSCIKRGGSCDHR
PNDCCYNSSCRCNLWGTNCRCQRMGLFQKWGK

Lycotoxin-3-Pverdolaga:
MGKITEVLLIFLICVLAIALVISAEVYCPAKSSIICKRSITKCCSDQDCPRGRICCQENCGNQCNIP
SSVQTNGSKVVLSDTCKIGQF

Lycotoxin-4-Pverdolaga:
XNARARSMDFLLDADNREATLPPENKLGSGQGRVKSEHELRIERSLQNLTIPDWYKQSPW
SKKPKEGFILRRX

Amino acid sequences of CRISP proteins translated from *Pamphobeteus verdolaga*. Residues highlighted in grey indicate the signal peptide, while magenta blue highlighted residues, indicates the propeptide according to

Spider|ProHMM from the arachnoserver. Red highlighted residues indicate the cysteine disposition commonly described in the ICK peptides.

CRISP-1- Pverdolaga:
MNHVVLLVLLAVTICYAVPRLSRPKIYGNAIPYRDLDTSNGATKKKIVLMHNFFRSRVQPSAS
DMLAMSWHEGAAEDAQRWAESCQLLLHDNTTGRWTQDFGTCGQNIFVANVQVPWFFAAK
VWFLERDNFTYGKNVNDPDVVGHYTQMVWYSTHRVGCGFHYCGPDVTKVPYYSYVCNYCP
IGNHPERFDRPYTRGKPCSACPGQCKFKKLCTNTCPHADTWINCRELNETWHDWLCGNEQN
EGHQACKATCYCEGAIR

CRISP-2- Pverdolaga:
MRIRVILMLSWLWLGVSDGSCPALYRRYSKAHTYCLPPKSSSTILKSGISKSDIETIVRVHNELRS
KVATGEETTYSMPKASNMRQMVWDSELAAVAQKHANQCLFKHDCNNCRKVKNFDVGQN
LFKRNPFSVPPQPTWAQAVTDWYSEVNVFDQGQIDGFIDGEGPPQTGHFTQDIWAESWRVGC
GYSVCEEGNVLLELYTCNYGPAGNGENDPIYERGDPCTNCPLNSCCGSSCSGGTSYPGLCRISG
ENAPQYNRPEGLTFYCSFNNEHDCAKTVTGHNKWQVSKTLSGSYIGIVLNGGESSTLSFKNPA
KVPQKPLCFIINYRTGPQVDGEEVSGTANVIFKAGGSTFPSELNSNGFQSFTKFSITLGWNMPT
MIDISISVPEGGSSRYLEIKDMSATKESARTMALSSLLSGHYARTMELSSLLSGHYARTMELSSLL
SGHYARTMX

CRISP-3- Pverdolaga:
MLEMSWHKEAQESAQRWAEECQILTYDGILGKYVEDYGSCGQNIFVSNEKVPWTFVGEAWF
AERYDFSYGSYNNSVSEVGHYTQMVWNGSHRLGCGFHYCAKDVKKPFYNYVCNYCPMGND
PKRLGMPYSSGKPCSECIKHCKYKKLCTNTCPYADLWVNCAQLNVTWNDWLCSNPEHQRH
RGCRATCLCPGKVI

Amino acid sequences of Hephaestin-like protein translated from *Pamphobeteus verdolaga*. Residues highlighted in grey indicate the signal peptide, while magenta blue highlighted residues, indicates the propeptide according to Spider|ProHMM from the arachnoserver. Red highlighted residues indicate the cysteine disposition commonly described in the ICK peptides.

Hephaestin-1- Pverdolaga:
MPTNLKIAIALTAAVMALVTAVILAVVLTSAKSVKTEAQQNVRTFYFAAEEVLWDYAPRETD
LLKERREDAGSSLEESEDPLRLGKRYKKAVFREYTDSTYSEAKSHPEWLGILGPLIETEVGDIVKI
NVYNNATHPVSLHTHGARYLKHHEGAFYQDNTPDKRDDKVFPGENYTYTWQISENRGPTES
DTPCMLWAYHSHVSGDEDIHTGLLGPLLICRQGYLDTMYEHSEFSGRRHFVLLFLIFDENQSW
YLDENIAVKRTNTTQGNTEEIKENEQFQESNLMYSVNGLMYDNLKGLRMCHGSQVTWYLMG
MGNEADIHAITFPGHPLLVREHRTDVVHLFPAKFESALMTSNREGSWPIKCEVGDAHIVGME
ARFSVRQCGNRNTSEMSSQTGQVREFFIAAEETVWNYAPTGINKMNGIPLLNDSEAAPFFERG
ENRIGGTYKKVVYRGYMDQHFTEPLHHPAHLGLLGPTIRAEVGETVRVHFFNKATKAYAFQL
TAFVCSDCEMAGQNKSVAPSGNRTYSFVIREDLGPSPKDPQCIPWRYFSAVEHLKDIYGGLNG
VLLICRPGTLKADGTQKNVDKEFTLMFAVMDENKSPYIDENIAEYTTSGDNVDKEDEDFVESN
LMHSINGLMYGHLDGLEMCVNDNVSWNLFAIGAVVDMHVVYFSGNTILRDGLYRDTVDVM
PSGHQIVSMNPDLLGKWPMTCRTNDHLRGGMKARYEVKSCNHNGDVLSGGSQDLTADDKI
RKYFIAAVDEEWDYAPFDYHIVTGENLTENENSRIFVTRDATHIGSKYMKTLYREFTDSSFSQR
KTRTPEEEHLGLLGPVIKAEVGETIQVVFRNKASRKFNIHTHGLPENSSQLYGVEPGGTQTYIW

KVPQRSGPTPGSFNCSSWMYYSAVDSIKDINTGLVGPLIICNPGILTNSGSRTDVDKEFSVLFTVF
DENESWYLEENVNNYCFEPEKVDVEDEDFKESNLKHSLNGLLYGALRGLGGTVGDTVAWYL
MGLGNEIDIHTAHFHGMSFVEKDDPLAGPKGHKADVTDLFPGIFRTVEMTFDTPGTWLFHCH
VDDHLKGGMIALVTVNDRNATTLH

Hephaestin-2- Pverdolaga:
XGSKGTAFMRLSCPGRIEPEASGQRPLHYSVNGRIYGTLEGLVAKNGTKTAWYLLGMGSEDDL
HTAHFHGQTFLQRTDTVRRGDVVDLFPGYFDTVEMINDNPGTWILHCHVDDHMRYGMAAL
FTVTP

Amino acid sequences of venom metalloproteinase translated from *Pamphobeteus verdolaga*. Residues highlighted in grey indicate the signal peptide, while magenta blue highlighted residues, indicates the propeptide according to Spider|ProHMM from the arachnoserver. Red highlighted residues indicate the cysteine disposition commonly described in the ICK peptides.

Metalloproteinase-1- Pverdolaga:
XGKHVVYKRKFPPIVKGLPTDYVTMDSEKMLNNTKHRSKRQAVPDTVWPEVLLVVDYETFIL
HGGNSRDVKRYFISFFNGVDLRYKLLHHPRVRISLAGMIVAKDRDATPYLERNRLRPPNADAV
DAAGALTDMGKYLYREHRLPTYDLAVVITKLDMCRRRFPGGRCNRGTAGFAYVGGACVVN
KRLEKVNSVAIIEDSGGFSGIIVAAHEIGHLLGCVHDGSPPPSYLGGPGATHCPWEDGFIMSDL
RHTERGFKWSSCSVEQFKHFLHGETATCLYNFPHENDLLVRVLPGTMLTLDEQCKRDRGTTA
CFKDARVCAQLFCFDTASGYCVSYRPAAEGSPCGDGQVCKNGRCLAEIENIIPDFSHVTETYVE
AEERMDRSEEEITKSTDTTTFPSRRARIRRLYRGPNRSLRSTVPKENLPTISTGFPKKNDPSRNMK
TRL

Section S2. Full-length translated sequences for putative protein ORFs corresponding to proteins matching housekeeping and cellular process proteins.

Amino acid sequences of contig c6436_g1_i1 translated from *Pamphobeteus verdolaga*.

MCDDDVAALVVDNGSGMCKAGFAGDDAPRAVFPSIVGRPRHQGVMVGMGQKDSYVGDEA
QSKRGILTLKYPIEHGIVTNWDDMEKIW

Amino acid sequences of contig c62193_g1_i1 translated from Pamphobeteus verdolaga.

TQIMFETFNTPAMYVAIQAVLSLYASGRTTGIVLDSGDGVSHTVPIYEGYALPHAILRLDLAGR
DLTDYLMKILTERGYSFTTTAEREIVRDIKEKLCYVALDFEQEMATAASSSLEKSYELPDGQVIT
IGNERFRCPEALFQPSFLGMESCGIHETTYNSIMKCDVDIRKDLYANTVLSGGTTMYPGIADRM
QKEITALAPSTMKIKIIAPPERKYSVW

Amino acid sequences of contig c13011_g1_i1 translated from *Pamphobeteus verdolaga*.

XEKIWHHTFYNELRVAPEEHPVLLTEAPLNPKANREKMTQIMFETFNTPAMYVAIQAVLSLYA
SGRTTGIVLDSGDGVSHTVPIYEGYALPHAILRLDLAGRDLTDYLMKILTERGYSFTTTAEREIV
RDIKEKLCYVALDFEQEMATAASSSSLEKSYELPDGQVITIGNERFRCPEAMFQPSFLGMESCGI
HETTYNSIMKCDVDIRKDLYANTVLSGGTTMYPGIADRMQKEITALAPSTMKIKIIAPPERKYS
VWIGGSILASLSTFQX

Amino acid sequences of contig c15096_g1_i3 translated from *Pamphobeteus verdolaga*.

MEASHVTEDHINDESRSESTNSMSHSRKYYRATQHRVFVNRSLHLEKIKFFGFDMDYTLAQYN
SPEYEALQFRLMVGRMISIGYPAELKDFEYDPTFPIRGLWFDTTYGNLLKVDAYGNILVCCHGF
QFLKTSEIYNLYPNKFIQHDESRIYILNTLFNLPEAYLLACLVDFFSNSPQYTPSKTGIKTGNIFMS
YKSIFQDVRDAIDWVHMQGSLKEETVNNIDKYVNKEDRLPMLFDRIHEVGKKTFLLTNSDYD
YTAKIMSYLFDHPKSGGRDWKSYFDYILVDARKPLFFGGGTTLRQVDTGTGALRIGIHVGPLH
PGQVYSGGSCDVFTSLIGAKGKDVLYVGDHIYGDILKSKKTRGWRTFLIVPELQREVHVWTSK
WQLFNRLQELDIQLGDTYKDLDSSCKDEPDISELRMAIREVSHELDMSYGILGSTFRSGSRQTFF
ANQICHYADLYACTFLNLMYYPFSYMFRAPPMLMPHESTVEHDEGSIVNGEAYELEEEQVQE
PMRRRSRLEHSESSVPHLFAETPEAVTHHHDTDDDEDTDKSGEH

Amino acid sequences of contig c27174_g1_i1 translated from *Pamphobeteus verdolaga*.

MAKVPAIGIDLGTTYSCVGVFQHGKVEIIANDQGNRTTPSYVAFTDTERLIGDAAKNQVAMN
PSNTVFDAKRLIGRRFEDPSVQSDMKHWPFDVVSDSGKPKIQVEYKGETKTFYPEEISSMVLTK
MKEIAEAYLGKTVTNAVVTVPAYFNDSQRQATKDAGTIAGLNVLRIINEPTAAAIAYGLDKK
GQGERNVLIFDLGGGTFDVSILTIEDGIFEVKSTAGDTHLGGEDFDNRMVNHFVQEFKRKHKK
DLTTNKRALRRLRTACERAKRTLSSSTQASIEIDSLFEGVDFYSTITRARFEELNADLFRSTLEPVE
KALRDAKLDKAQVHDIVLVGGSTRIPKIQKLLQDFFNGKDLNKSINPDEAVAYGAAVQAAIL
NGDKSEQVQDLLLLDVTPLSLGIETAGGVMTVLIKRNTTIPTRQTQTFTTYSDNQPGVLIQVYE
GERAMTKDNNLLGKFELTGIPPAPRGVPQIEVTFDIDANGILNVSAVDKSTGKENKITITNDKG
RLSKEEIERMVKDAEKYKDEDDKQKCRISSKNSLESYAFNVKSTVEDDKLKEKISEDDKKKVLD
KVNETLRWLDSNQLADKEEYEHKQKELEGVCTPIITKLYQGAGGAPGGMPGFPGAGAAPGG
GGTGSGGPTIEEVD

Amino acid sequences of contig c15743_g1_i1 translated from *Pamphobeteus verdolaga*.

MPAIGIDLGTTYSCVGVFQHGQVEIIANDQGNRTTPSYVAFTDTERLIGDPAKSQVVLNPENTV
FDAKRLIGRKHDDPKIASDLKHWPFQVCDAGGKPKISVMYKGEKKLFNPEEISAMVLTKMKE
TAEMYLGEKVTEAVITVPAYFNDSQRQATKDAGIIAGLNVLRIINEPTAAALAYGLDKNLQGE
KNVLIFDLGGGTFDVSVLTIDEGSLFEVRATAGDTHLGGEDFDNRMVNHFIEEFKRMHKKDL
QGNARALRRLRTACERAKRTLSSGTEASIEIDALYQGIDFYSKITRARFEELCMDLFRSTLEPVEK
ALTDAKMDKNSIHDVVLVGGSTRIPKIQKLLRDFFNGKELCMSINPDEAVAYGAAVQAAVLT
GDKSEKIRDVLLVDVAPLSLGIETAGGVMTKIVQRNSRIPCKTSQIFTTYSDNQPGVTIQVFEGE
RAMTKDNHLLGKFDLSGIPPAQRGVPQIEVTFNLDANGILQVSACDKGTGKLQSIQITNDKGR
LTKEEIERMLAEAEKYQQEDEKQREKVSARNSLESYVYSVKQAASSVDNNRLSETDKKKVNDT
CDDVIRWLDNNTLAEKEEIEYKMKEVQSQLSPIMTKLHQAGGSPNNYSENSSATSSGPTVEEV
D

Amino acid sequences of contig c10792_g1_i2 translated from *Pamphobeteus verdolaga*.

MAKVPAIGIDLGTTYSCVGVFQHGKVEIIANDQGNRTTPSYVAFTDTERLIGDAAKNQVAMN
PSNTVFDAKRLIGRRX

Amino acid sequences of contig c2143_g1_i2 translated from *Pamphobeteus verdolaga*.

MARTKQTARKSTGGKAPRKQLATKAARKSAPSTGGVKKPHRYRPGTVALREIRRYQKSTELLI
RKLPFQRLVREIAQDFKTDLRFQSAAIGALQEASE

Amino acid sequences of contig c16774_g1_i2 translated from *Pamphobeteus verdolaga*.

XGKQLEDGRMLSDYNIQKESTLHLVLRLRGGMQIFVKTLTGKTITLEVEPSDTIENVKAKIQDK
EGIPPDQQRLIFAGKQLEDGRTLSDYNIQKESTLHLVLRLRGGMQIFVKTLTGKTITLEVEPSDTI
ENVKAKI

Amino acid sequences of contig c17180_g4_i1 translated from *Pamphobeteus verdolaga*.

MCDDDIAALVVDNGSGMCKAGFAGDDAPRAVFPSIVGRPRHQGVMVGMGQKDSYVGDEA
QSKRGILSLKYPIEHGIITNWDDMEKIWHHTFYNELRVAPEEHPILLTEAPLNPKANREKMTQI
MFETFNAPAMYVAIQAVLSLYASGRTTGIVLDSGDGVSHTVPIYEGYALPHAILRLDLAGRDLT
DYLMKILTERGYSFVTTAEREIVRDIKEKLCYVALDFEQEMATAASSSTVEKSYELPDGQVITIG
NERFRCPETLFQPSFIGMESVGIHETTFNSIQKCDIDIRKDLYANTVLSGGTTMYPGIADRMQKE
ITALAPSTMKIKIIAPPERKYSVWIGGSILASLSTFQQMWISKQEYDESGPSIVHRKCF

Amino acid sequences of contig c16820_g1_i1 translated from *Pamphobeteus verdolaga*.

AAAIAYGLDKKGQGERNVLIFDLGGGTFDVSILTIEDGIFEVKSTAGDTHLGGEDFDNRMVNH
FVQEFKRKHKKDLTTNKRSLRRLRTACERAKRTLSSSTQASIEIDSLFEGVDFYSTITRARFEELN
ADLFRGTLEPVEKALRDAKLDKAQIHDIVLVGGSTRIPKIQKLLQDFFNGKDLNKSINPDEAVA
YGAAVQAAILNGDKSEQVQDLLLLDVTPLSLGIETA

Amino acid sequences of contig c9831_g1_i1 translated from *Pamphobeteus verdolaga*.

XLNGSPIPLLPCADSRKFLPLLVEASHSRVTVYLSGVLNYLLYNPAVQLIKSNMPAIGIDLGTTY
SCVGVFQHGNVEIIANDQGNRTTPSYVAFTDVERLIGDPAKSQAAMNPENTVFDAKRLIGRRY
DDPKIAADIKNWPFEVCNVGGKPKVGVLYKGENKMFNPEEISAMVLTKMKETAEMYLGEKV
TDAVVTVPAYFNDSQRQATKDAGAIAGLTVLRIINEPTAAALAYGLDKNLKGEKKVLIFDLGG
GTFDVSILTIDEGSLFEVKATAGDTHLGGEDFDNRMVNYFVEEFHRKHKKDLRSSPRAIRRLRT
ACERAKRTLSSSTEAGIEVDAIFEGIDLYSKISRARFEELCIDLFRSTLEPVERALSDAKMNKASIH
DVVLVGGSTRIPKIQKLLRDFFNGKELCMSINPDEAVAYGAAVQAAVLSGDTSKNIRDVLLVD
VIPLSLGIETAGGVMTKIVGRNSRIPCKTSQDFTTYSDNQTSVTVQVYEGERAMTKDNHLLGKF
NLSGIPPVPRGVPRIEVTFNLDSNGILHVSAVEKSSGKSQSIRITNDKGRLSKQEIDRMLEDADK
YQREDEIQREKVAARNTLESYLYSVKQGADSANYSSLSPSDKAKVNNICEDAIRWLDNNSLAE
KEEILYKNKEVQAQLSPIMTKLHQRSSSGPIIEEVD

Amino acid sequences of contig c16774_g1_i2 translated from *Pamphobeteus verdolaga*.

XGKQLEDGRMLSDYNIQKESTLHLVLRLRGGMQIFVKTLTGKTITLEVEPSDTIENVKAKIQDK
EGIPPDQQRLIFAGKQLEDGRTLSDYNIQKESTLHLVLRLRGGMQIFVKTLTGKTITLEVEPSDTI
ENVKAKI

Amino acid sequences of contig c14336_g1_i1 translated from *Pamphobeteus verdolaga*.

XGIETAGGVMTVLIKRNTTIPTRQTQTFTTYSDNQPGVLIQVYEGERAMTKDNNLLGKFELTGI
PPAPRGVPQIEVTFDIDA

Amino acid sequences of contig c51913_g1_i1 translated from *Pamphobeteus verdolaga*.

DIKEKLCYVALDFEQEMATAASSSSLEKSYELPDGQVITIGNERFRCPEALFQPSFLGMESCGIH
ETTYNSIMKCDVDIRKDLYANTVLSGGTTMYPGIADRMQKEITALAPSTMKIKIIAPPERKYSV
WIGGSILASLSTFQQMWISKQEYDESGPSIVHRKCF

Amino acid sequences of contig c2143_g1_i2 translated from *Pamphobeteus verdolaga*.

MARTKQTARKSTGGKAPRKQLATKAARKSAPSTGGVKKPHRYRPGTVALREIRRYQKSTELLI
RKLPFQRLVREIAQDFKTDLRFQSAAIGALQEASE

Amino acid sequences of contig c4667_g1_i1 translated from *Pamphobeteus verdolaga*.

MARTKQTARKSTGGKAPRKQLATKAARKSAPSTGGVKKPHRYRPGTVALREIRRYQKSTELLI
RKLPFQRLVREIAQDFKTDLRFQSAAIGALQEASEAYLVGLFEDTNLCAIHAKRVTIMPKDIQL
ARRIRGERA

Amino acid sequences of contig c15300_g1_i1 translated from *Pamphobeteus verdolaga*.

MFLKLPDVAQLCHAXTEENSAVKHRGHFRKWLTNPVRKLSHGRLDRSSSDNPKTSVKKPCPD
PTKVVNSKEEMGNKSSSPGPPIKHAEGDIEPEVTVENTAPEVSPEAPDVNGESTDATSTCPIVIP
SSSEAEGNSSAAVNLEPEGEEPQVEQSSEDNCPLTEQSNESKFLEHEEIEKALQKRKFVLQELVT
TEKQYVKDLGSLVEGYIELMKSGEITMPEDLKNGKDKIVFGNIEAIYEWHRDLFCAELEKCLEE
PERLGLLFRRYERRLNMYVVYCQNKPKSEYIVSEYLDTFFEEARQKLGHKLQLPDLLIKPVQRI
MKYQLLLKDILKYTEKAGIEEEAQNLRKAVHIMHVVPKAANDMMNVGRLQGFDGKITGQG
KLLQQGILFISDPSTGGKMKERQVFLFEQIIIFSDSVGPKTQFSNPVYIYKNHLQVNKMSLEERSE
ESDPTKFLLKSKDPMQQGLTFVIQGASQEERDEWVANIRAILDTQLDFLRALQSPIAYQKELTK
DISAPELGSLWNPSLRKTLSHPAAAHKTVKSGSATTGPSLTSKSLRHPGRDKKVRSADVTKPKS
VSAGTSGENLKEQTDGLVKECRAHSLAVPPLTLGDQSSDSSESCNKNNGNSTPDVTSDGQRK
HSLPIEKRSNLAQQSPPKSKRNFFEGFRNTLRPKSKTDSVLSGSGESTAVSLSSSHSLDSASAAQG
KQDLSLNNKDSDMIRRWSETNPSRSVKGRRDMRWPHTLPPRLSPALCSATEDYSASQSDEIPLS
KGDTVHLISKKHSRCFVKKYSEDDDNGTTEGWVPSYVIGQDVRKNRSFRLRKLGLSMERLGH
KWTSGGDKKTETPKLNDSKNLQTPNDLPDSVVSNDEKKSLIFIESVSNMEVPEGEDATLAGRV
APADCLVVKWTLPDNSEIESKSIESDELSDSQFSPSTSPDSPMFDIKVQAFVFSDGGFSLTIRNCA
NSYSGQYVCTVNSPFGKIQTTCHLTVLGKPTPPGRPYVVERCGDSVCLQWSPPLCTGNLQLI

Amino acid sequences of contig c34105_g1_i1 translated from *Pamphobeteus verdolaga*.

CSQLSCSFPGISSLPNFYHEVGEQHQHVGNPIVFRGKDSVSFSALSQVPPATSFSSTTTVGHMNS
HTQEWSSSHQGKAGGVLRSSKSVSDLHSLPQLEDSSSFPTSVRSHTPPPNIANQVLDNEKGLKK
DSSRENDSASLPDPYFELEESERQFVDSVTDMGFPRGQVARTVKHLGTDDKKVVEHLCQIQTL
EESGYDSLEAEAALHLHDYNSDEARKYLDLLIKIQSLGFDKTARS

Amino acid sequences of contig c5964_g1_i2 translated from *Pamphobeteus verdolaga*.

XHLVPFSPLSDRDRGLRHPFGKAQYCRGVSLQPRAGKSKIAQRAPAAGESVTAIVHSQYNSPA
AMYSMNAIADTLSAHAEMIAPGVMGINFMKPEKPINTMSEVYKLVQEEEQMKGDRTPSPAA
PSQKKPYFGSLKVAQATSPTPPSSTPPVRQVRVSAPPVKTPPPVAPKPVLKPVLKPVAPPAPAA
PTSPGVTPPQANCSDCGRFIAGIFVRLNDRTLHEECFRCSTCGTSLKNMGYVNISNKLYCDIHA
KLAAKIIPPEDVSPTPPVVNAPAAPPAPAFTPAPAFTPAPPPPAPAPAQVPPPLPMPPAAVAPK
VTQTAPSPFSPQLQQTLGGDLGNLPHTPSSSGGLPFRSISPQPFRRAAGDYHKVIAPMSPPTTGS
TAAPYFPQSGPLPFEMTPYMPPEPVPSSVFSSSSQQITQVSQTSQQTTQITQQTSQTSQFSVDYRK
VIAQQSGRTGTKFIWPPPKPGEAPVLESNVTPMPPTYRPPPNTQHYSPLAMQSTPSSLSQTLSFR
SVQPPVPAPPAPSAAPPAPAPPAPAPLPFVPPPLPSSFTKPAEPEPPKQEPPKSEPQATEAAPEQ
KETSKPEPPPPQPLPLPTSAPSGPSAPGFAPVLAPVVDPSSEPKPSAPQSGVPTGAGSRPAPKRG
RGQLKEQTPGSRIPICAVCGSPIRGPFVTALGKTWCPDHFHCNNVHCKAPLQDIGFVEEHGQL
YCENCYEAFLAPICNKCTVRIKGDCLNALDKQWHPECFICAYCSKPFGNTSFYLEDGLPYCEK
DWNELFTTKCVGCGYPIEAGDRWVEALNNNYHSQCFKCSICNKNLEGQSFYAKGGRPFCKA
HAR

Amino acid sequences of contig c14908_g1_i2 translated from *Pamphobeteus verdolaga*.

MSYGSSYGSGRYGSGYGSSSYGSGSRRTGGSSYTPSTISSSSYVPYRRSNGGSSYGLMQSKSASYIS
PTSSPRSSISSASGTTYLSSRIPSSNSYRNSTSYDSPSYGSSKGVYKTLLDSKKSDYVPDTEERCKLT
ASRSTGSVDQDDVSDSSKEEEDDEEEEEDDDSISERAKQKYGSSTSLPDTSSEVSSRGETPSRYSS
VSSSRSSLIEDSNKDYKKLYEETKKENERLREKLKKSEDELERVKQQLQKSTQNNNTRNSISETE
KKERRALERRISEMEEEVKTLSKLKAENEKLKAENRALSRVVCKLSK

Amino acid sequences of contig c4688_g1_i1 translated from *Pamphobeteus verdolaga*.

XLRELNECVGEEQSFKPVSSEVDAIRSQQDEFKHYHQDRLEPLGKQIDGMNKMGQGLIQSAAP
GVSTSVLECDLDVLNDRWNSLKQRMNERERRLDVAFLQSGKFQEAMAGVQKWLEDTEEMV
ANQKPPSADYKVVKAQLQEQKFLNKLLLDRQSSMSSLMTMGTEIMNNMEPMERIQLEAQLS
DLMQRFDELMNGAQERTDALERTIPVAKDFQDRMSPLVEWLEQTEKKLAGMATIPTDQEKIR
QRMVEHEALHEDIMDHKEAFEELTEIAQMLMGLVGDDEAQVVVEKLQEVTDHFAKVVEDSE
HIGQLLAEAYQGMGSFTVNYEDLMAWIDEMASRLSRFHVLSVYVEKLQEQLDELVELSEEIAD
HQKQVDDVASAGQDIMKHASGDDVIRMKEKLDSLSVKFTDLTSRAADRLRQAQDSLPLVQN
FHSSHEKVTAWMDSAERQLKSLENVGLTSQETSIQKLEAEIQEYRPMVDTLNHLGPQLCQMSP
GEGAAVIETQVSRVNRRFDAICEQVQRKAERIDLSKQRNVEVIGDIEDLLDWFHEVEKQMMQ
AEPLSADPDSLTALLKEQKILNEEVSSQKGRVRDILVAAKKLMRESSGDDLAEVRDKADELKD
VTNAVASLCADRLAALEQALPLAEHFFETHADLCQWLDEIESEAELLEAPALNANQIKKQQE
RNKALMQSVNDHKALVDKLNKTGEALKKLCTPEEASRVQDVMDSDNSRYSSLKDILRDRQN
ALEEALQATSQFSDKLDGMLNALSNTADQLNNAEPVSAHPEKIEEQIDENKAVLKDLDKRSN
ALEAVKRAADDVIVKAGGARDPAVKDIKQKLDKLNDLWDNIQKLARNRGRSLEDALAAAE
RFWDELTTVMKALKDLQDSLAAQEPPAIEPNAIQQQQEVLQEI

Amino acid sequences of contig c14491_g1_i2 translated from *Pamphobeteus verdolaga*.

MEFPALGKHCFEKSCNMLDFLPMKCDACGNVFCKDHIHYIKHSCERAYQKDIQVPVCPLCN
KPVPSKRSEPPDIAVGEHIDRDCQSDPAVAKRKIYTNRCAVKSCKRKEVIPLSCDNCRKNFCLK
HRHSADHECRDIKTSHESLSPSGAAALVRMQASNKTCSKTSSKQSFSNAVMTANLPGSVIETE
KSVRSASACMSEDEALALALQKSLSDCNKAPPLPKDVQEEEDRLLAQALAASEIDQRTAVSGV
QRHNXGQELYSVLAAVLSMLLN

Amino acid sequences of contig c9864_g1_i1 translated from *Pamphobeteus verdolaga*.

MSYRNQYSGSPWQQGGPPFGVGGYGQSYGGYGLNDQYAMMNSGYSRSQGGYGSMYGSSG
MSQMRSMGYGGPSPSRHYLNDRMGGRQGSRGQMQRKRLPQRSPAGGPRSKRPNLDSESRRN
RSKPRRRRDSRGGSKGRRDHSDDEKGDEPYNPAEPTDDASDINYDDYESWKSSSDDENETKEV
TQNGEEQPAIDGEETKTKTEKTDESDKAKVDAKKPLICHVCKITCSNPERFRKHTISRTHRSKM
DTLLALQREKKQVLEASMKASQQVESRDSSKKGSRKPGNWCTVCECSFSGNFLAHRRTKEHR
KKRDKKYPKCRPCRVGFSSSEEYKEHCKKEDHKLKSAEFHYLKNLASGSDDDEAAIEPIAEYIE
AEDETKKDAEMKEEKEGSEEKKEEEQEKMDEAKDGEETTDEKKDKKDDGKDSKKSSKQKDS
KESKEPKAIGQSFVVSVQGYYCKLCHKFFKDVTVAKVKHCRSIMHNGAFKKAMEEAMEQEEE
RQKLADQQEKIEREIAEAARLAADIAAVEKAAKEAAKREAAEKKAAQKAKDSETKESVDQN
ENAAVEKMETDVANGEKEEMEVTEETKESGEETKGKTEPQPPPAPVAAAEEEKPAETAQSDS
QGDEEVEEEPEKAKEEPTSSPATRRGRGRGRGQSRGKGKRR

Amino acid sequences of contig c62910_g1_i1 translated from *Pamphobeteus verdolaga*.

MFRMQNNTVVDLLHPDLLSTTVLQEILVERKIGEEHINGASREQLLSLYSKVILPLPQREFRKNR
RGAYLSKKCGRNALQEKSTESQVEVKAKSTSPVPCPETQSRLPATKNKIVGLHATEPQSRLKPP
PISTESGKIIKLNSRATNGDSIISPKRSMSPPGDSKRPKLIRLNRTLPPQNNKSKDDHCEETTDPSP
PKKKHKPILWP

Amino acid sequences of contig c29740_g1_i1 translated from *Pamphobeteus verdolaga*.

MIQTAMPGVDVWRISPEVKAKYDEQFFQLKPINGYVTGDQAKNLFLQSGLPPQILAQIWALA
DRNADGKMDSFEFAIAMHLIQMKLKGAELPKILPSSMQVAAPQSVAIAPGFRPPVAVGPPVP
AVPTSTMAPLTAGFIRPASPVDAKLQRSGSVSSQDSPTGVPPPLIEWAVPQQSKLKYTQLFNSY
DRTRTGFLTGAQARNILVQTGLSHVILAQIWSLADIDADGRLSCEEFVLAMHLTDCVKAGDTL
PPALPPDLIPPSHRRKRSTSIQSNVSISSHSNIGDMLLLGDLKEEDKLKGLPATFEDKRRENFEKG
QAELERRRLALLESQRKEQEERERKEREEQERRERIRQEQERRRQLELEKQLAKQRELEQEKEE
QRRKALEQREAARREMERQRQLEWEKQRQQELLSQRQKEQETVCHLKNVNKNLTFELEELA
GKIKDLNEKISETRKGVTAMKSSIDDMRNERDSKLKEIADVKSKIKEFNDRLLV

Amino acid sequences of contig c11572_g1_i1 translated from *Pamphobeteus verdolaga*.

MVLVSAEGSERRPPMDAPENPVLLPKQSTWTKGDEHMVYLDNARAKREQSKQPTSMYGQLV
TQDQLVAISTRTQGSQNDLVALQSPTPYKCRDPSSAPLRKLSVDLIKTYKHINEVYYAKKKRRA
QQTQGDDTSHKKERKIFNDGYDDDNHDYIIRNGEKFLDRYEIDSLIGKGSFGQVVKAYDHED
QCHVAIKIIKNKKPFLNQAQIEVKLLEMMNNHDSDGSYCPVGQDKIVKLKGHFMWRNHLCL
VFELLSYNLYDLLRNTNFRGVSLNLTRKFAQQMCTALMFLSSSDLNIIHCDLKPENILLCNPKR

SAIKIVDFGSSCQLGQRIYQYIQSRFYRSPEVLLGIPYDMAIDMWSLGCILVEMHTGEPLFSGAN
EVDQMNKIVEVLGMPPKHILDQAHKSRKYFDRLPDGTFVLKKTKDGKKYKSPGTRKLHDILG
VETGGPGGRRLGEPGHTVSDYLKFKDLILRMLDYDPKTRISPYHALQHSFFKRTSDESTNTSHS
ASTSPAMEQGSVGNTNTGAGGAGSGSSSGSSSSCAPPQVAGRARSDPTHQHPFGQLQHSYTQ
TGSYSMQFGTQVSITTAAAAAALSAMECESPITSIAGAGIFRSSQKIHAWSALGAAGTPTTLTPA
IGASTGTVTVGQHSHSSAPTSRVGHSHHHSHNHSYRRHLHHQTQSSASGQTSASTSHAMNAV
SFDPLQSFQPPATFSVVSSSRNQACSSSSVASSQLMPMDINSSNNLFASLDCTQTGSLNIQLGSSY
QPLLYTGTVYSGQTTVPSYLGTNFSQLGIHPSSTPTTQPSSVVSPDVVSQKQSTDRDESPMVGVC
VEQSPVASH

Amino acid sequences of contig c5016_g1_i1 translated from *Pamphobeteus verdolaga*.

KEVYEDDESLIPKNTSVIVARVPVVSCNKKSWERSDLPLPVDDDELSGQINFDKVVKSADLVN
ANVSEEDKVKAMISQSSQEYDPSKYLKCRSMTGPLPPSYTCFRCGKQGHWIKNCPTNNVDIKR
STGIPRSFMVPVDGPEHKGALLTSSGEYAVPLIDHVAYKEVKREKPPFVNVPEPEVEPEAQIPEE
LLCMVCRDLLQDAVLIPCCGNSFCDECVRQVLLDSDNHECPLCHETGISPDTVIPNRFLRTAVL
NFRNETGYTRVKRMPSIPFPQPSHMDSPQPSPDSTVENQAAPPEPVPEEAATVEDGTAVPITTV
PEPENVEPECNETTESTPQPPDPPVQEETEETVEPGNEDLQPGTPLADEPPVHNVPEPELAENE
QYPSHNQYDEENKFSYSHYYNENMDSDSRDEGHVSSSEINQISTIETITTRLYNYKSRSDRPSHS
FSQQRPVHHGHTRDPDSSSLPSRRGGSSHTYRSKPQEWPKGSLTVLVERDYNHDRLDYRDRYD
RRNRDRPTNTSRFPQRSHNRYERPRETRYNQSDYHSENRCEYRDYSNNSELREDVNYEIRTNS
GESTHSHAQRYNSPPASEHTQNKINVAAPPPPPPPX

Amino acid sequences of contig c17992_g1_i1 translated from *Pamphobeteus verdolaga*.

MAYLXIVISDNGPQYASAEFHNFVQQYDFQHITSSPGYPQANREVERMVRAVKDLLKRYPPLP
LILPQYAWSDWCLTSTVLYGSQASNEXCLLIIVNSSLSGQIFDSTRRKITTLKSSNKSGITAVTQP
DLFQFSTPEITLEPRFGYIRDCPQFLYYSPILQSHYRQGHSHEKELPPPGETTPAICWDITPCSLLE
PKPMPATASQVLPTHASEADNTMSDKPLEPASPAARTEIXVTDENQQRTDHEASKVPQSESEG
DVVVLTQ

Section S3. Raw material of proteomic reports.

Header --

Search title Sebastian-AS-pep-5600 (\\nucleus.ecn.purdue.edu\vhedrick\My Documents\Mascot Daemon\Sebastian_AS-peptides_5600.par), submitted from Daemon on 1212-B119PC12

Timestamp 2015-08-20T14:01:03Z
User
Email
Report URI
http://mascot.bbc.purdue.edu/mascot/cgi/master_results.pl?file=../data/20150820/F020650.dat

Peak list data path C:\ProgramData\Matrix Science\Mascot Daemon\MGF\833 Sebastian-AS-pep-5600\mascot_daemon_merge.mgf

Peak list format Mascot generic
Search type MIS
Mascot version 2.5.1
Database AS_peptides_081915

Fasta file AS_peptides_081915_20150819.fasta
Total sequences 1834
Total residues 175871

Sequences after taxonomy filter 1834
Number of queries 271399
Decoy --

Number of matches above identity threshold in search of real database 78

Number of matches above identity threshold in search of decoy database 25

Number of matches above homology threshold in search of real database 78
Number of matches above homology threshold in search of decoy database 25
Fixed modifications--

Identifier Name Delta Neutral loss

1 Ethanolyl (C) 44,026215

Variable modifications --

Identifier Name Delta Neutral loss(es)
1 Acetyl (K) 42,010565
2 Oxidation (M) 15,994915 0 63,998285

Search Parameters --

Taxonomy filter All entries
Enzyme Trypsin

Maximum Missed Cleavages 1

Fixed modifications Ethanolyl (C)

Variable modifications Acetyl (K),Oxidation (M)
Peptide Mass Tolerance 0,05
Peptide Mass Tolerance Units Da
Fragment Mass Tolerance 0,2
Fragment Mass Tolerance Units Da
Mass values Monoisotopic

Instrument type ESI-QUAD-TOF

Decoy database also searched 1
Format parameters --

Significance threshold 0,05

Max. number of hits 0

Use MudPIT protein scoring 1

Ions score cut-off 0
Include same-set proteins 0
Include sub-set proteins 1
Include unassigned 0
Require bold red 0
Use homology threshold 1
Group protein families 1

Re-score using Percolator 0
Show duplicate peptides 1
Protein hits --

prot_hit_num prot_family_member prot_acc prot_score
prot_mass prot_matches prot_matches_sig prot_sequences
prot_sequences_sig prot_cover pep_query pep_rank pep_isbold
pep_isunique pep_exp_mz pep_exp_mr pep_exp_z pep_calc_mr
pep_delta pep_miss pep_score pep_expect pep_res_before
pep_seq pep_res_after pep_var_mod pep_var_mod_pos
pep_summed_mod_pos pep_local_mod_pos

4 1 >Hyaluronidase-1-Loxosceles intermedia|sp:R4J7Z9|1899 Hyaluronidase from the venom of the spider Loxosceles intermedia 22
46697 18 4 4 1 12,8 87360 1 1 1
536,2918 2141,138 4 2141,1595 -0,0214 1 2,09
0,62 K YGMKFVPLLEQYSILVNK E

6 1 >U16-lycotoxin-Ls1a|sp:B6DD52|1075 Toxin from venom of the spider Lycosa singoriensis with unknown molecular target and function 15
2174 1 2 1 18005 1 1 1 421,7672
841,5198 2 841,5385 -0,0187 1 1,37 0,73 K
GIIRTIK D Acetyl (K) 0.0000001.0

6 1 >U16-lycotoxin-Ls1a|sp:B6DD52|1075 Toxin from venom of the spider Lycosa singoriensis with unknown molecular target and function 15
2174 1 2 1 63948 1 1 1 498,2414
994,4682 2 994,5124 -0,0441 1 2,99 0,5 R
TIKDWYK G Acetyl (K) 0.0010000.0

7 1 >SphingomyelinaseD(LlSicTox-alphaIII1i)|sp:Q8I914|132 Sphingomyelinase D (EC 3.1.4.41; LlSicTox-alphaIII1i) from the spider Loxosceles laeta 14 34985 45 3 2 1 5,1 3983 1
1 1 406,695 811,3755 2 811,3348 0,0407 0
5,38 0,29 K ESGYNDK Y

7 1 >SphingomyelinaseD(LlSicTox-alphaIII1i)|sp:Q8I914|132 Sphingomyelinase D (EC 3.1.4.41; LlSicTox-alphaIII1i) from the spider Loxosceles laeta 14 34985 45 3 2 1 5,1 67559 1
1 1 504,2342 1006,4539 2 1006,472 -0,018 0
0,06 1,7 R DSANGFINK I Acetyl (K)
0.000000001.0

8 1 >CRISP-2-Grammostola rosea|sp:M5WW7 |1844 Translation of a cysteine rich secretory protein (CRISP) from the spider Grammostola rosea
13 46078 40 1 5 1 11,5 61364 1 1 1
493,2478 984,481 2 984,524 -0,043 1 0,01 3,3
R TGPQVKGEK S Acetyl (K) 0.000001000.0

8 1 >CRISP-2-Grammostola rosea|sp:M5WW7 |1844 Translation of a cysteine rich secretory protein (CRISP) from the spider Grammostola rosea
13 46078 40 1 5 1 11,5 85926 2 0 1
533,2644 1064,5143 2 1064,5178 -0,0035 1 1,14
0,77 K DWYKEIK D 2 Acetyl (K) 0.0001001.0

8 1 >CRISP-2-Grammostola rosea|sp:M5WW7 |1844 Translation of a cysteine rich secretory protein (CRISP) from the spider Grammostola rosea
13 46078 40 1 5 1 11,5 152184 1 1
1 728,3399 1454,6653 2 1454,7075 -0,0423 1
9,26 0,12 K VATGKETQYSMPK A Oxidation (M)
0.0000000000200.0

8 1 >CRISP-2-Grammostola rosea|sp:M5WW7 |1844 Translation of a cysteine rich secretory protein (CRISP) from the spider Grammostola rosea
13 46078 40 1 5 1 11,5 67400 1 1 1
503,7584 2011,0045 4 2010,9973 0,0072 1 0,4 2,8
K SFPTVLTSSSMSFTKFTK K Oxidation (M)
0.000000000020000000.0

Section S4. Gen Ontology terms using the Panther database of the different proteins with catalytic activity.

Oxidoreductase Activity

HUMAN|HGNC=3482|UniProtKB=P38117	ETFB	Electron transfer flavoprotein subunit beta;ETFB;ortholog	ELECTRON TRANSFER FLAVOPROTEIN SUBUNIT BETA (PTHR21294:SF8)	hydroxylase(PC00122)	*Homo sapiens*
HUMAN|HGNC=7695|UniProtKB=O75438	NDUB1	NADH dehydrogenase [ubiquinone] 1 beta subcomplex subunit 1;NDUFB1;ortholog	NADH DEHYDROGENASE [UBIQUINONE] 1 BETA SUBCOMPLEX SUBUNIT 1 (PTHR15222:SF4)	dehydrogenase(PC00092);mitochondrial carrier protein(PC00158);transfer/carrier protein(PC00219)	*Homo sapiens*
HUMAN|HGNC=15987|UniProtKB=O76003	GLRX3	Glutaredoxin-3;GLRX3;ortholog	GLUTAREDOXIN-3 (PTHR10293:SF40)	reductase(PC00198)	*Homo sapiens*
HUMAN|HGNC=11180|UniProtKB=P04179	SODM	Superoxide dismutase [Mn], mitochondrial;SOD2;ortholog	SUPEROXIDE DISMUTASE [MN], MITOCHONDRIAL (PTHR11404:SF35)	oxidoreductase(PC00176)	*Homo sapiens*
HUMAN|HGNC=6052|UniProtKB=P20839	IMDH1	Inosine-5'-monophosphate dehydrogenase 1;IMPDH1;ortholog	INOSINE-5'-MONOPHOSPHATE DEHYDROGENASE 1 (PTHR11911:SF74)	dehydrogenase(PC00092);metalloprotease(PC00153);reductase (PC00198)	*Homo sapiens*
HUMAN|HGNC=10683|UniProtKB=O14521	DHSD	Succinate dehydrogenase [ubiquinone] cytochrome b small subunit, mitochondrial;SDHD;ortholog	SUCCINATE DEHYDROGENASE [UBIQUINONE] CYTOCHROME B SMALL SUBUNIT-RELATED (PTHR13337:SF6)	transfer/carrier protein(PC00219)	*Homo sapiens*
HUMAN|HGNC=30231|UniProtKB=O75663	TIPRL	TIP41-like protein;TIPRL;ortholog	TIP41-LIKE PROTEIN (PTHR21021:SF16)		*Homo sapiens*
HUMAN|HGNC=1334|UniProtKB=Q96IV6	FXDC2	Fatty acid hydroxylase domain-containing protein 2;FAXDC2;ortholog	FATTY ACID HYDROXYLASE DOMAIN-CONTAINING PROTEIN 2 (PTHR11863:SF26)	hydroxylase(PC00122);oxidase(PC00175)	*Homo sapiens*

HUMAN|HGNC=29836|UniProtKB=Q9NRX3	NUA4L	NADH dehydrogenase [ubiquinone] 1 alpha subcomplex subunit 4-like 2;NDUFA4L2;ortholog	NADH DEHYDROGENASE [UBIQUINONE] 1 ALPHA SUBCOMPLEX SUBUNIT 4-LIKE 2 (PTHR14256:SF5)	oxidoreductase(PC00176)	*Homo sapiens*
HUMAN|HGNC=23287|UniProtKB=O95571	ETHE1	Persulfide dioxygenase ETHE1, mitochondrial;ETHE1;ortholog	PERSULFIDE DIOXYGENASE ETHE1, MITOCHONDRIAL (PTHR43084:SF4)	hydrolase(PC00121)	*Homo sapiens*
HUMAN|HGNC=2638|UniProtKB=P20815	CP3A5	Cytochrome P450 3A5;CYP3A5;ortholog	CYTOCHROME P450 3A5 (PTHR24302:SF20)	oxygenase(PC00177)	*Homo sapiens*
HUMAN|HGNC=12009|UniProtKB=P60174	TPIS	Triosephosphate isomerase;TPI1;ortholog	TRIOSEPHOSPHATE ISOMERASE (PTHR21139:SF22)	isomerase(PC00135)	*Homo sapiens*
HUMAN|HGNC=2861|UniProtKB=P00374	DYR	Dihydrofolate reductase;DHFR;ortholog	DIHYDROFOLATE REDUCTASE (PTHR22778:SF46)	reductase(PC00198)	*Homo sapiens*
HUMAN|HGNC=7699|UniProtKB=O95168	NDUB4	NADH dehydrogenase [ubiquinone] 1 beta subcomplex subunit 4;NDUFB4;ortholog	NADH DEHYDROGENASE [UBIQUINONE] 1 BETA SUBCOMPLEX SUBUNIT 4 (PTHR15469:SF1)	reductase(PC00198)	*Homo sapiens*
HUMAN|HGNC=2570|UniProtKB=P00167	CYB5	Cytochrome b5;CYB5A;ortholog	CYTOCHROME B5 (PTHR19359:SF78)	oxidase(PC00175)	*Homo sapiens*
HUMAN|HGNC=12587|UniProtKB=P47985	UCRI	Cytochrome b-c1 complex subunit Rieske, mitochondrial;UQCRFS1;ortholog	CYTOCHROME B-C1 COMPLEX SUBUNIT RIESKE, MITOCHONDRIAL-RELATED (PTHR10134:SF1)		*Homo sapiens*
HUMAN|HGNC=382|UniProtKB=O60218	AK1BA	Aldo-keto reductase family 1 member B10;AKR1B10;ortholog	ALDO-KETO REDUCTASE FAMILY 1 MEMBER B10 (PTHR11732:SF278)	reductase(PC00198)	*Homo sapiens*
HUMAN|HGNC=4866|UniProtKB=Q9BQS7	HEPH	Hephaestin;HEPH;ortholog	HEPHAESTIN (PTHR11709:SF221)	oxidase(PC00175)	*Homo sapiens*

HUMAN|HGNC=2898|UniProtKB=P09622	DLDH	Dihydrolipoyl dehydrogenase, mitochondrial;DLD;ortholog	DIHYDROLIPOYL DEHYDROGENASE, MITOCHONDRIAL (PTHR22912:SF151)	dehydrogenase(PC00092);oxidase(PC00175);reductase(PC00198)	*Homo sapiens*
HUMAN|HGNC=30862|UniProtKB=O14957	QCR10	Cytochrome b-c1 complex subunit 10;UQCR11;ortholog	CYTOCHROME B-C1 COMPLEX SUBUNIT 10 (PTHR15420:SF2)		*Homo sapiens*
HUMAN|HGNC=17194|UniProtKB=Q9P0J0	NDUAD	NADH dehydrogenase [ubiquinone] 1 alpha subcomplex subunit 13;NDUFA13;ortholog	NADH DEHYDROGENASE [UBIQUINONE] 1 ALPHA SUBCOMPLEX SUBUNIT 13 (PTHR12966:SF0)	dehydrogenase(PC00092)	*Homo sapiens*
HUMAN|HGNC=17366|UniProtKB=Q9UDR5	SDH, AASS	Alpha-aminoadipic semialdehyde synthase, mitochondrial;AASS;ortholog	ALPHA-AMINOADIPIC SEMIALDEHYDE SYNTHASE, MITOCHONDRIAL (PTHR11133:SF18)		*Homo sapiens*
HUMAN|HGNC=7422|UniProtKB=P00414	COX3	Cytochrome c oxidase subunit 3;MT-CO3;ortholog	CYTOCHROME C OXIDASE SUBUNIT 3 (PTHR11403:SF7)	oxidase(PC00175)	*Homo sapiens*
HUMAN|HGNC=11476|UniProtKB=O15260	SURF4	Surfeit locus protein 4;SURF4;ortholog	SURFEIT LOCUS PROTEIN 4 (PTHR23427:SF1)	oxidase(PC00175);transmembrane receptor regulatory/adaptor protein(PC00226)	*Homo sapiens*
HUMAN|HGNC=4313|UniProtKB=P23378	GCSP	Glycine dehydrogenase (decarboxylating), mitochondrial;GLDC;ortholog	GLYCINE DEHYDROGENASE (DECARBOXYLATING), MITOCHONDRIAL (PTHR11773:SF1)	dehydrogenase(PC00092)	*Homo sapiens*
HUMAN|HGNC=29079|UniProtKB=O60341	KDM1A	Lysine-specific histone demethylase 1A;KDM1A;ortholog	LYSINE-SPECIFIC HISTONE DEMETHYLASE 1A (PTHR10742:SF350)	DNA methyltransferase(PC00013);oxidase(PC00175)	*Homo sapiens*
HUMAN|HGNC=5385|UniProtKB=O43837	IDH3B	Isocitrate dehydrogenase [NAD] subunit beta, mitochondrial;IDH3B;ortholog	ISOCITRATE DEHYDROGENASE [NAD] SUBUNIT BETA, MITOCHONDRIAL (PTHR11835:SF42)	dehydrogenase(PC00092)	*Homo sapiens*

HUMAN|HGNC=4455|UniProtKB=P21695	GPDA	Glycerol-3-phosphate dehydrogenase [NAD(+)], cytoplasmic;GPD1;ortholog	GLYCEROL-3-PHOSPHATE DEHYDROGENASE [NAD(+)], CYTOPLASMIC (PTHR11728:SF32)	dehydrogenase(PC00092)	*Homo sapiens*
HUMAN|HGNC=4801|UniProtKB=P40939	ECHA	Trifunctional enzyme subunit alpha, mitochondrial;HADHA;ortholog	TRIFUNCTIONAL ENZYME SUBUNIT ALPHA, MITOCHONDRIAL (PTHR43612:SF5)	dehydrogenase(PC00092);epimerase/racemase(PC00096);hydratase(PC00120)	*Homo sapiens*
HUMAN|HGNC=2640|UniProtKB=P24462	CP3A7	Cytochrome P450 3A7;CYP3A7;ortholog	CYP3A7-CYP3A51P READTHROUGH-RELATED (PTHR24302:SF31)	oxygenase(PC00177)	*Homo sapiens*
HUMAN|HGNC=17450|UniProtKB=Q9HB55	CP343	Cytochrome P450 3A43;CYP3A43;ortholog	CYTOCHROME P450 3A43 (PTHR24302:SF6)	oxygenase(PC00177)	*Homo sapiens*
HUMAN|HGNC=2269|UniProtKB=P10606	COX5B	Cytochrome c oxidase subunit 5B, mitochondrial;COX5B;ortholog	CYTOCHROME C OXIDASE SUBUNIT 5B, MITOCHONDRIAL (PTHR10122:SF0)	oxidase(PC00175)	*Homo sapiens*
HUMAN|HGNC=19691|UniProtKB=Q9BV79	MECR	Enoyl-[acyl-carrier-protein] reductase, mitochondrial;MECR;ortholog	ENOYL-[ACYL-CARRIER-PROTEIN] REDUCTASE, MITOCHONDRIAL (PTHR43981:SF2)		*Homo sapiens*
HUMAN|HGNC=29958|UniProtKB=Q8NEX9	DR9C7	Short-chain dehydrogenase/reductase family 9C member 7;SDR9C7;ortholog	SHORT-CHAIN DEHYDROGENASE/REDUCTASE FAMILY 9C MEMBER 7 (PTHR43313:SF5)	dehydrogenase(PC00092);reductase(PC00198)	*Homo sapiens*
HUMAN|HGNC=410|UniProtKB=P43353	AL3B1	Aldehyde dehydrogenase family 3 member B1;ALDH3B1;ortholog	ALDEHYDE DEHYDROGENASE FAMILY 3 MEMBER B1 (PTHR43570:SF2)		*Homo sapiens*
HUMAN|HGNC=976|UniProtKB=P54687	BCAT1	Branched-chain-amino-acid aminotransferase, cytosolic;BCAT1;ortholog	BRANCHED-CHAIN-AMINO-ACID AMINOTRANSFERASE, CYTOSOLIC (PTHR11825:SF70)	transaminase(PC00216)	*Homo sapiens*
HUMAN|HGNC=21492|UniProtKB=Q8N0U8	VKORL	Vitamin K epoxide reductase complex subunit 1-like protein 1;VKORC1L1;ortholog	VITAMIN K EPOXIDE REDUCTASE COMPLEX SUBUNIT 1-LIKE PROTEIN 1 (PTHR14519:SF5)	oxidoreductase(PC00176)	*Homo sapiens*

HUMAN|HGNC=7690|UniProtKB=P56556	NDUA6	NADH dehydrogenase [ubiquinone] 1 alpha subcomplex subunit 6;NDUFA6;ortholog	NADH DEHYDROGENASE [UBIQUINONE] 1 ALPHA SUBCOMPLEX SUBUNIT 6 (PTHR12964:SF2)	dehydrogenase(PC00092)	*Homo sapiens*
HUMAN|HGNC=18503|UniProtKB=Q9BYV7	BCDO2	Beta,beta-carotene 9',10'-oxygenase;BCO2;ortholog	BETA,BETA-CAROTENE 9',10'-OXYGENASE (PTHR10543:SF61)	oxygenase(PC00177)	*Homo sapiens*
HUMAN|HGNC=9353|UniProtKB=P32119	PRDX2	Peroxiredoxin-2;PRDX2;ortholog	PEROXIREDOXIN-2 (PTHR10681:SF151)	peroxidase(PC00180)	*Homo sapiens*
HUMAN|HGNC=5384|UniProtKB=P50213	IDH3A	Isocitrate dehydrogenase [NAD] subunit alpha, mitochondrial;IDH3A;ortholog	ISOCITRATE DEHYDROGENASE [NAD] SUBUNIT ALPHA, MITOCHONDRIAL (PTHR11835:SF34)	dehydrogenase(PC00092)	*Homo sapiens*
HUMAN|HGNC=9752|UniProtKB=P09417	DHPR	Dihydropteridine reductase;QDPR;ortholog	DIHYDROPTERIDINE REDUCTASE (PTHR15104:SF0)	dehydrogenase(PC00092);reductase(PC00198)	*Homo sapiens*
HUMAN|HGNC=2277|UniProtKB=P12074	CX6A1	Cytochrome c oxidase subunit 6A1, mitochondrial;COX6A1;ortholog	CYTOCHROME C OXIDASE SUBUNIT 6A1, MITOCHONDRIAL (PTHR11504:SF4)	oxidase(PC00175)	*Homo sapiens*
HUMAN|HGNC=380|UniProtKB=P14550	ALR	Alcohol dehydrogenase [NADP(+)];AKR1A1;ortholog	ALCOHOL DEHYDROGENASE [NADP(+)] (PTHR11732:SF401)	reductase(PC00198)	*Homo sapiens*
HUMAN|HGNC=30086|UniProtKB=Q6P1R4	DUS1L	tRNA-dihydrouridine(16/17) synthase [NAD(P)(+)]-like;DUS1L;ortholog	TRNA-DIHYDROURIDINE(16/17) SYNTHASE [NAD(P)(+)]-LIKE (PTHR11082:SF5)		*Homo sapiens*
HUMAN|HGNC=4795|UniProtKB=O95479	6PGL	GDH/6PGL endoplasmic bifunctional protein;H6PD;ortholog	GDH/6PGL ENDOPLASMIC BIFUNCTIONAL PROTEIN (PTHR23429:SF7)	dehydrogenase(PC00092)	*Homo sapiens*
HUMAN|HGNC=10451|UniProtKB=P23921	RIR1	Ribonucleoside-diphosphate reductase large subunit;RRM1;ortholog	RIBONUCLEOSIDE-DIPHOSPHATE REDUCTASE LARGE SUBUNIT (PTHR11573:SF6)	reductase(PC00198)	*Homo sapiens*

HUMAN|HGNC=25996|UniProtKB=Q6P6C2	ALKB5	RNA demethylase ALKBH5;ALKBH5;ortholog	RNA DEMETHYLASE ALKBH5 (PTHR32074:SF2)		*Homo sapiens*
HUMAN|HGNC=15472|UniProtKB=O94788	AL1A2	Retinal dehydrogenase 2;ALDH1A2;ortholog	RETINAL DEHYDROGENASE 2 (PTHR11699:SF102)	dehydrogenase(PC00092)	*Homo sapiens*
HUMAN|HGNC=4555|UniProtKB=P22352	GPX3	Glutathione peroxidase 3;GPX3;ortholog	GLUTATHIONE PEROXIDASE 3 (PTHR11592:SF32)	peroxidase(PC00180)	*Homo sapiens*
HUMAN|HGNC=4923|UniProtKB=P52789	HXK2	Hexokinase-2;HK2;ortholog	HEXOKINASE-2 (PTHR19443:SF4)		*Homo sapiens*
HUMAN|HGNC=15814|UniProtKB=Q9NR19	ACSA	Acetyl-coenzyme A synthetase, cytoplasmic;ACSS2;ortholog	ACETYL-COENZYME A SYNTHETASE, CYTOPLASMIC (PTHR24095:SF126)	dehydrogenase(PC00092);ligase(PC00142)	*Homo sapiens*
HUMAN|HGNC=964|UniProtKB=O75936	BODG	Gamma-butyrobetaine dioxygenase;BBOX1;ortholog	GAMMA-BUTYROBETAINE DIOXYGENASE (PTHR10696:SF33)	hydroxylase(PC00122);oxygenase(PC00177)	*Homo sapiens*
HUMAN|HGNC=7693|UniProtKB=Q16795	NDUA9	NADH dehydrogenase [ubiquinone] 1 alpha subcomplex subunit 9, mitochondrial;NDUFA9;ortholog	NADH DEHYDROGENASE [UBIQUINONE] 1 ALPHA SUBCOMPLEX SUBUNIT 9, MITOCHONDRIAL (PTHR12126:SF10)	dehydrogenase(PC00092);reductase(PC00198)	*Homo sapiens*
HUMAN|HGNC=16631|UniProtKB=P82909	RT36	28S ribosomal protein S36, mitochondrial;MRPS36;ortholog	28S RIBOSOMAL PROTEIN S36, MITOCHONDRIAL (PTHR31601:SF3)		*Homo sapiens*
HUMAN|HGNC=6666|UniProtKB=Q9Y4K0	LOXL2	Lysyl oxidase homolog 2;LOXL2;ortholog	LYSYL OXIDASE HOMOLOG 2 (PTHR45817:SF1)		*Homo sapiens*
HUMAN|HGNC=1063|UniProtKB=P30043	BLVRB	Flavin reductase (NADPH);BLVRB;ortholog	FLAVIN REDUCTASE (NADPH) (PTHR43355:SF2)	reductase(PC00198)	*Homo sapiens*

HUMAN|HGNC=25598|UniProtKB=Q9NV66	TYW1	S-adenosyl-L-methionine-dependent tRNA 4-demethylwyosine synthase;TYW1;ortholog	S-ADENOSYL-L-METHIONINE-DEPENDENT TRNA 4-DEMETHYLWYOSINE SYNTHASE (PTHR13930:SF0)	oxidoreductase(PC00176);transporter(PC00227)	*Homo sapiens*
HUMAN|HGNC=24288|UniProtKB=Q8NE62	CHDH	Choline dehydrogenase, mitochondrial;CHDH;ortholog	CHOLINE DEHYDROGENASE, MITOCHONDRIAL (PTHR11552:SF147)	dehydrogenase(PC00092)	*Homo sapiens*
HUMAN|HGNC=7459|UniProtKB=P03905	NU4M	NADH-ubiquinone oxidoreductase chain 4;MT-ND4;ortholog	NADH-UBIQUINONE OXIDOREDUCTASE CHAIN 4 (PTHR43507:SF1)	dehydrogenase(PC00092);reductase(PC00198)	*Homo sapiens*
HUMAN|HGNC=19331|UniProtKB=Q96EY8	MMAB	Cob(I)yrinic acid a,c-diamide adenosyltransferase, mitochondrial;MMAB;ortholog	COB(I)YRINIC ACID A,C-DIAMIDE ADENOSYLTRANSFERASE, MITOCHONDRIAL (PTHR12213:SF0)	dehydrogenase(PC00092)	*Homo sapiens*
HUMAN|HGNC=12582|UniProtKB=P14927	QCR7	Cytochrome b-c1 complex subunit 7;UQCRB;ortholog	CYTOCHROME B-C1 COMPLEX SUBUNIT 7 (PTHR12022:SF2)	reductase(PC00198)	*Homo sapiens*
HUMAN|HGNC=6971|UniProtKB=P40926	MDHM	Malate dehydrogenase, mitochondrial;MDH2;ortholog	MALATE DEHYDROGENASE, MITOCHONDRIAL (PTHR11540:SF16)	dehydrogenase(PC00092)	*Homo sapiens*
HUMAN|HGNC=4335|UniProtKB=P00367	DHE3	Glutamate dehydrogenase 1, mitochondrial;GLUD1;ortholog	GLUTAMATE DEHYDROGENASE 1, MITOCHONDRIAL (PTHR11606:SF25)	dehydrogenase(PC00092)	*Homo sapiens*
HUMAN|HGNC=19086|UniProtKB=Q96DB2	HDA11	Histone deacetylase 11;HDAC11;ortholog	HISTONE DEACETYLASE 11 (PTHR43497:SF2)	deacetylase(PC00087);nucleic acid binding(PC00171);reductase(PC00198)	*Homo sapiens*
HUMAN|HGNC=10603|UniProtKB=O75880	SCO1	Protein SCO1 homolog, mitochondrial;SCO1;ortholog	PROTEIN SCO1 HOMOLOG, MITOCHONDRIAL (PTHR12151:SF4)	oxidase(PC00175)	*Homo sapiens*

HUMAN|HGNC=21298|UniProtKB=Q86V21	AACS	Acetoacetyl-CoA synthetase;AACS;ortholog	ACETOACETYL-COA SYNTHETASE (PTHR42921:SF1)	dehydrogenase(PC00092);ligase(PC00142)	*Homo sapiens*
HUMAN|HGNC=20837|UniProtKB=Q6QHF9	PAOX	Peroxisomal N(1)-acetyl-spermine/spermidine oxidase;PAOX;ortholog	PEROXISOMAL N(1)-ACETYL-SPERMINE/SPERMIDINE OXIDASE (PTHR10742:SF43)	DNA methyltransferase(PC00013);oxidase(PC00175)	*Homo sapiens*
HUMAN|HGNC=2571|UniProtKB=P49447	CY561	Cytochrome b561;CYB561;ortholog	CYTOCHROME B561 (PTHR10106:SF14)	reductase(PC00198)	*Homo sapiens*
HUMAN|HGNC=18685|UniProtKB=Q7Z5P4	DHB13	17-beta-hydroxysteroid dehydrogenase 13;HSD17B13;ortholog	17-BETA-HYDROXYSTEROID DEHYDROGENASE 13 (PTHR24322:SF499)		*Homo sapiens*
HUMAN|HGNC=14966|UniProtKB=Q92626	PXDN	Peroxidasin homolog;PXDN;ortholog	PEROXIDASIN HOMOLOG (PTHR11475:SF75)	peroxidase(PC00180)	*Homo sapiens*
HUMAN|HGNC=6053|UniProtKB=P12268	IMDH2	Inosine-5'-monophosphate dehydrogenase 2;IMPDH2;ortholog	INOSINE-5'-MONOPHOSPHATE DEHYDROGENASE 2 (PTHR11911:SF121)	dehydrogenase(PC00092);metalloprotease(PC00153);reductase (PC00198)	*Homo sapiens*
HUMAN|HGNC=17169|UniProtKB=Q13162	PRDX4	Peroxiredoxin-4;PRDX4;ortholog	PEROXIREDOXIN-4 (PTHR10681:SF128)	peroxidase(PC00180)	*Homo sapiens*
HUMAN|HGNC=13280|UniProtKB=Q96HE7	ERO1L	ERO1-like protein alpha;ERO1A;ortholog	ERO1-LIKE PROTEIN ALPHA (PTHR12613:SF1)	oxidoreductase(PC00176)	*Homo sapiens*
HUMAN|HGNC=15862|UniProtKB=Q9NWM0	SMOX	Spermine oxidase;SMOX;ortholog	SPERMINE OXIDASE (PTHR10742:SF347)	DNA methyltransferase(PC00013);oxidase(PC00175)	*Homo sapiens*
HUMAN|HGNC=30141|UniProtKB=Q96Q83	ALKB3	Alpha-ketoglutarate-dependent dioxygenase alkB homolog 3;ALKBH3;ortholog	ALPHA-KETOGLUTARATE-DEPENDENT DIOXYGENASE ALKB HOMOLOG 3 (PTHR31212:SF4)		*Homo sapiens*

HUMAN|HGNC=11474|UniProtKB=Q15526	SURF1	Surfeit locus protein 1;SURF1;ortholog	SURFEIT LOCUS PROTEIN 1 (PTHR23427:SF2)	oxidase(PC00175)	*Homo sapiens*
HUMAN|HGNC=4458|UniProtKB=P06744	G6PI	Glucose-6-phosphate isomerase;GPI;ortholog	GLUCOSE-6-PHOSPHATE ISOMERASE (PTHR11469:SF4)	isomerase(PC00135)	*Homo sapiens*
HUMAN|HGNC=8891|UniProtKB=P52209	6PGD,PGDH	6-phosphogluconate dehydrogenase, decarboxylating;PGD;ortholog	6-PHOSPHOGLUCONATE DEHYDROGENASE, DECARBOXYLATING (PTHR11811:SF53)	dehydrogenase(PC00092)	*Homo sapiens*
HUMAN|HGNC=6041|UniProtKB=A1L0T0	ILVBL	Acetolactate synthase-like protein;ILVBL;ortholog	ACETOLACTATE SYNTHASE-LIKE PROTEIN (PTHR18968:SF155)	decarboxylase(PC00089);dehydrogenase(PC00092);transferase(PC00220)	*Homo sapiens*
HUMAN|HGNC=3481|UniProtKB=P13804	ETFA	Electron transfer flavoprotein subunit alpha, mitochondrial;ETFA;ortholog	ELECTRON TRANSFER FLAVOPROTEIN SUBUNIT ALPHA, MITOCHONDRIAL (PTHR43153:SF1)	dehydrogenase(PC00092);oxidase(PC00175);transferase(PC00220)	*Homo sapiens*
HUMAN|HGNC=4623|UniProtKB=P00390	GSHR	Glutathione reductase, mitochondrial;GSR;ortholog	GLUTATHIONE REDUCTASE, MITOCHONDRIAL (PTHR42737:SF2)	dehydrogenase(PC00092);oxidase(PC00175);reductase(PC00198)	*Homo sapiens*
HUMAN|HGNC=2494|UniProtKB=Q13363	CTBP	C-terminal-binding protein 1;CTBP1;ortholog	C-TERMINAL-BINDING PROTEIN 1 (PTHR46029:SF2)	transcription cofactor(PC00217)	*Homo sapiens*
HUMAN|HGNC=2596|UniProtKB=P05177	CP1A2	Cytochrome P450 1A2;CYP1A2;ortholog	CYTOCHROME P450 1A2 (PTHR24299:SF7)	oxygenase(PC00177)	*Homo sapiens*
HUMAN|HGNC=3773|UniProtKB=P49326	FMO5	Dimethylaniline monooxygenase [N-oxide-forming] 5;FMO5;ortholog	DIMETHYLANILINE MONOOXYGENASE [N-OXIDE-FORMING] 5 (PTHR23023:SF78)	oxygenase(PC00177)	*Homo sapiens*
HUMAN|HGNC=7432|UniProtKB=P11586	C1TC	C-1-tetrahydrofolate synthase, cytoplasmic;MTHFD1;ortholog	C-1-TETRAHYDROFOLATE SYNTHASE, CYTOPLASMIC (PTHR43274:SF2)	ligase(PC00142)	*Homo sapiens*

HUMAN|HGNC=23198|UniProtKB=Q6ZWL3	CP4V2	Cytochrome P450 4V2;CYP4V2;ortholog	CYTOCHROME P450 4V2 (PTHR24291:SF56)	oxygenase(PC00177)	*Homo sapiens*
HUMAN|HGNC=5386|UniProtKB=P51553	IDH3G	Isocitrate dehydrogenase [NAD] subunit gamma, mitochondrial;IDH3G;ortholog	ISOCITRATE DEHYDROGENASE [NAD] SUBUNIT GAMMA, MITOCHONDRIAL (PTHR11835:SF60)	dehydrogenase(PC00092)	*Homo sapiens*
HUMAN|HGNC=32487|UniProtKB=Q6NS38	ALKB2	DNA oxidative demethylase ALKBH2;ALKBH2;ortholog	DNA OXIDATIVE DEMETHYLASE ALKBH2 (PTHR31573:SF1)		*Homo sapiens*
HUMAN|HGNC=7473|UniProtKB=Q9UBK8	MTRR	Methionine synthase reductase;MTRR;ortholog	METHIONINE SYNTHASE REDUCTASE (PTHR19384:SF84)		*Homo sapiens*
HUMAN|HGNC=18985|UniProtKB=Q7Z4W1	DCXR	L-xylulose reductase;DCXR;ortholog	L-XYLULOSE REDUCTASE (PTHR44252:SF2)		*Homo sapiens*
HUMAN|HGNC=4892|UniProtKB=Q93099	HGD	Homogentisate 1,2-dioxygenase;HGD;ortholog	HOMOGENTISATE 1,2-DIOXYGENASE (PTHR11056:SF0)	oxygenase(PC00177)	*Homo sapiens*
HUMAN|HGNC=11179|UniProtKB=P00441	SODC	Superoxide dismutase [Cu-Zn];SOD1;ortholog	SUPEROXIDE DISMUTASE [CU-ZN] (PTHR10003:SF58)	oxidoreductase(PC00176)	*Homo sapiens*
HUMAN|HGNC=6983|UniProtKB=P48163	MAOX	NADP-dependent malic enzyme;ME1;ortholog	NADP-DEPENDENT MALIC ENZYME (PTHR23406:SF17)	acyltransferase(PC00042);decarboxylase(PC00089);dehydrogenase(PC00092)	*Homo sapiens*
HUMAN|HGNC=10293|UniProtKB=Q96AT9	RPE	Ribulose-phosphate 3-epimerase;RPE;ortholog	RIBULOSE-PHOSPHATE 3-EPIMERASE-RELATED (PTHR11749:SF9)		*Homo sapiens*
HUMAN|HGNC=28242|UniProtKB=Q96IR7	HPDL	4-hydroxyphenylpyruvate dioxygenase-like protein;HPDL;ortholog	4-HYDROXYPHENYLPYRUVATE DIOXYGENASE-LIKE PROTEIN (PTHR11959:SF10)	oxygenase(PC00177)	*Homo sapiens*

HUMAN|HGNC=26222|UniProtKB=Q8WVX9	FACR1	Fatty acyl-CoA reductase 1;FAR1;ortholog	FATTY ACYL-COA REDUCTASE 1 (PTHR11011:SF45)		*Homo sapiens*
HUMAN|HGNC=7891|UniProtKB=Q9NPH5	NOX4	NADPH oxidase 4;NOX4;ortholog	NADPH OXIDASE 4 (PTHR11972:SF78)	oxidase(PC00175)	*Homo sapiens*
HUMAN|HGNC=28697|UniProtKB=Q8N4Q0	PTGR3	Prostaglandin reductase 3;ZADH2;ortholog	PROSTAGLANDIN REDUCTASE 3 (PTHR43677:SF3)	dehydrogenase(PC00092);reductase(PC00198)	*Homo sapiens*
HUMAN|HGNC=987|UniProtKB=P21953	ODBB	2-oxoisovalerate dehydrogenase subunit beta, mitochondrial;BCKDHB;ortholog	2-OXOISOVALERATE DEHYDROGENASE SUBUNIT BETA, MITOCHONDRIAL (PTHR42980:SF1)	dehydrogenase(PC00092);lyase(PC00144);transketolase(PC00221)	*Homo sapiens*
HUMAN|HGNC=4829|UniProtKB=P02042	HBD	Hemoglobin subunit delta;HBD;ortholog	HEMOGLOBIN SUBUNIT DELTA (PTHR11442:SF50)		*Homo sapiens*
HUMAN|HGNC=4803|UniProtKB=P55084	ECHB	Trifunctional enzyme subunit beta, mitochondrial;HADHB;ortholog	TRIFUNCTIONAL ENZYME SUBUNIT BETA, MITOCHONDRIAL (PTHR18919:SF141)	acetyltransferase(PC00038)	*Homo sapiens*
HUMAN|HGNC=2577|UniProtKB=P13498	CY24A	Cytochrome b-245 light chain;CYBA;ortholog	CYTOCHROME B-245 LIGHT CHAIN (PTHR15168:SF0)		*Homo sapiens*
HUMAN|HGNC=3951|UniProtKB=Q16595	FRDA	Frataxin, mitochondrial;FXN;ortholog	FRATAXIN, MITOCHONDRIAL (PTHR16821:SF4)	cation transporter(PC00068);kinase(PC00137)	*Homo sapiens*
HUMAN|HGNC=7702|UniProtKB=P17568	NDUB7	NADH dehydrogenase [ubiquinone] 1 beta subcomplex subunit 7;NDUFB7;ortholog	NADH DEHYDROGENASE [UBIQUINONE] 1 BETA SUBCOMPLEX SUBUNIT 7 (PTHR20900:SF0)	dehydrogenase(PC00092)	*Homo sapiens*
HUMAN|HGNC=404|UniProtKB=P05091	ALDH2	Aldehyde dehydrogenase, mitochondrial;ALDH2;ortholog	ALDEHYDE DEHYDROGENASE, MITOCHONDRIAL (PTHR11699:SF233)	dehydrogenase(PC00092)	*Homo sapiens*

HUMAN|HGNC=4057|UniProtKB=P11413	G6PD	Glucose-6-phosphate 1-dehydrogenase;G6PD;ortholog	GLUCOSE-6-PHOSPHATE 1-DEHYDROGENASE (PTHR23429:SF0)	dehydrogenase(PC00092)	*Homo sapiens*
HUMAN|HGNC=7715|UniProtKB=O00217	NDUS8	NADH dehydrogenase [ubiquinone] iron-sulfur protein 8, mitochondrial;NDUFS8;ortholog	NADH DEHYDROGENASE [UBIQUINONE] IRON-SULFUR PROTEIN 8, MITOCHONDRIAL (PTHR10849:SF24)	Homo sapiens	
HUMAN|HGNC=9083|UniProtKB=O60568	PLOD3	Procollagen-lysine,2-oxoglutarate 5-dioxygenase 3;PLOD3;ortholog	PROCOLLAGEN-LYSINE,2-OXOGLUTARATE 5-DIOXYGENASE 3 (PTHR10730:SF7)	oxygenase(PC00177)	*Homo sapiens*
HUMAN|HGNC=29838|UniProtKB=Q9UHB4	NDOR1	NADPH-dependent diflavin oxidoreductase 1;NDOR1;ortholog	NADPH-DEPENDENT DIFLAVIN OXIDOREDUCTASE 1 (PTHR19384:SF10)		*Homo sapiens*
HUMAN|HGNC=26927|UniProtKB=Q96CU9	FXRD1	FAD-dependent oxidoreductase domain-containing protein 1;FOXRED1;ortholog	FAD-DEPENDENT OXIDOREDUCTASE DOMAIN-CONTAINING PROTEIN 1 (PTHR13847:SF44)	dehydrogenase(PC00092);oxidase(PC00175)	*Homo sapiens*
HUMAN|HGNC=18308|UniProtKB=Q9NVH6	TMLH	Trimethyllysine dioxygenase, mitochondrial;TMLHE;ortholog	TRIMETHYLLYSINE DIOXYGENASE, MITOCHONDRIAL (PTHR10696:SF3)		*Homo sapiens*
HUMAN|HGNC=4570|UniProtKB=Q9UBQ7	GRHPR	Glyoxylate reductase/hydroxypyruvate reductase;GRHPR;ortholog	GLYOXYLATE REDUCTASE/HYDROXYPYRUVATE REDUCTASE (PTHR10996:SF137)	dehydrogenase(PC00092)	*Homo sapiens*
HUMAN|HGNC=24475|UniProtKB=Q9UI17	M2GD	Dimethylglycine dehydrogenase, mitochondrial;DMGDH;ortholog	DIMETHYLGLYCINE DEHYDROGENASE, MITOCHONDRIAL (PTHR13847:SF187)	dehydrogenase(PC00092);oxidase(PC00175)	*Homo sapiens*
HUMAN|HGNC=4907|UniProtKB=P31937	3HIDH	3-hydroxyisobutyrate dehydrogenase, mitochondrial;HIBADH;ortholog	3-HYDROXYISOBUTYRATE DEHYDROGENASE, MITOCHONDRIAL (PTHR22981:SF7)	dehydrogenase(PC00092)	*Homo sapiens*
HUMAN|HGNC=7863|UniProtKB=Q13423	NNTM	NAD(P) transhydrogenase, mitochondrial;NNT;ortholog	NAD(P) TRANSHYDROGENASE, MITOCHONDRIAL (PTHR10160:SF22)	dehydrogenase(PC00092)	*Homo sapiens*

HUMAN|HGNC=408|UniProtKB=P51649	SSDH	Succinate-semialdehyde dehydrogenase, mitochondrial;ALDH5A1;ortholog	SUCCINATE-SEMIALDEHYDE DEHYDROGENASE, MITOCHONDRIAL (PTHR43353:SF5)	dehydrogenase(PC00092)	*Homo sapiens*
HUMAN|HGNC=253|UniProtKB=P11766	ADHX	Alcohol dehydrogenase class-3;ADH5;ortholog	ALCOHOL DEHYDROGENASE CLASS-3 (PTHR43880:SF4)	dehydrogenase(PC00092);reductase(PC00198)	*Homo sapiens*
HUMAN|HGNC=7706|UniProtKB=O95298	NDUC2	NADH dehydrogenase [ubiquinone] 1 subunit C2;NDUFC2;ortholog	NADH DEHYDROGENASE [UBIQUINONE] 1 SUBUNIT C2-RELATED (PTHR13099:SF0)	oxidoreductase(PC00176)	*Homo sapiens*
HUMAN|HGNC=1516|UniProtKB=P04040	CATA	Catalase;CAT;ortholog	CATALASE (PTHR11465:SF9)	peroxidase(PC00180)	*Homo sapiens*
HUMAN|HGNC=16232|UniProtKB=Q96KJ9	COX42	Cytochrome c oxidase subunit 4 isoform 2, mitochondrial;COX4I2;ortholog	CYTOCHROME C OXIDASE SUBUNIT 4 ISOFORM 2, MITOCHONDRIAL (PTHR10707:SF11)		*Homo sapiens*
HUMAN|HGNC=7179|UniProtKB=Q02252	MMSA	Methylmalonate-semialdehyde dehydrogenase [acylating], mitochondrial;ALDH6A1;ortholog	METHYLMALONATE-SEMIALDEHYDE DEHYDROGENASE [ACYLATING], MITOCHONDRIAL (PTHR43866:SF3)	dehydrogenase(PC00092)	*Homo sapiens*
HUMAN|HGNC=10452|UniProtKB=P31350	RIR2	Ribonucleoside-diphosphate reductase subunit M2;RRM2;ortholog	RIBONUCLEOSIDE-DIPHOSPHATE REDUCTASE SUBUNIT M2 (PTHR23409:SF20)	reductase(PC00198)	*Homo sapiens*
HUMAN|HGNC=7873|UniProtKB=P35228	NOS2	Nitric oxide synthase, inducible;NOS2;ortholog	NITRIC OXIDE SYNTHASE, INDUCIBLE (PTHR19384:SF56)		*Homo sapiens*
HUMAN|HGNC=8923|UniProtKB=O43175	SERA	D-3-phosphoglycerate dehydrogenase;PHGDH;ortholog	D-3-PHOSPHOGLYCERATE DEHYDROGENASE (PTHR42938:SF9)	dehydrogenase(PC00092)	*Homo sapiens*
HUMAN|HGNC=7455|UniProtKB=P03886	NU1M	NADH-ubiquinone oxidoreductase chain 1;MT-ND1;ortholog	NADH-UBIQUINONE OXIDOREDUCTASE CHAIN 1 (PTHR11432:SF3)	dehydrogenase(PC00092);reductase(PC00198)	*Homo sapiens*

HUMAN|HGNC=10297|UniProtKB=P49247	RPIA	Ribose-5-phosphate isomerase;RPIA;ortholog	RIBOSE-5-PHOSPHATE ISOMERASE (PTHR11934:SF0)		*Homo sapiens*
HUMAN|HGNC=2689|UniProtKB=P09172	DOPO	Dopamine beta-hydroxylase;DBH;ortholog	DOPAMINE BETA-HYDROXYLASE (PTHR10157:SF29)		*Homo sapiens*
HUMAN|HGNC=5398|UniProtKB=P13284	GILT	Gamma-interferon-inducible lysosomal thiol reductase;IFI30;ortholog	GAMMA-INTERFERON-INDUCIBLE LYSOSOMAL THIOL REDUCTASE (PTHR13234:SF8)	reductase(PC00198)	*Homo sapiens*
HUMAN|HGNC=17822|UniProtKB=Q9H7Z7	PGES2	Prostaglandin E synthase 2;PTGES2;ortholog	PROSTAGLANDIN E SYNTHASE 2 (PTHR12782:SF5)	reductase(PC00198)	*Homo sapiens*
HUMAN|HGNC=4195|UniProtKB=P35557	GCK	Glucokinase;GCK;ortholog	GLUCOKINASE (PTHR19443:SF3)		*Homo sapiens*
HUMAN|HGNC=9081|UniProtKB=Q02809	PLOD	Procollagen-lysine,2-oxoglutarate 5-dioxygenase 1;PLOD1;ortholog	PROCOLLAGEN-LYSINE,2-OXOGLUTARATE 5-DIOXYGENASE 1 (PTHR10730:SF5)	oxygenase(PC00177)	*Homo sapiens*
HUMAN|HGNC=11834|UniProtKB=P29401	TKT	Transketolase;TKT;ortholog	TRANSKETOLASE (PTHR43195:SF3)	dehydrogenase(PC00092);lyase(PC00144);transketolase(PC00221)	*Homo sapiens*
HUMAN|HGNC=412|UniProtKB=P49189	AL9A1	4-trimethylaminobutyraldehyde dehydrogenase;ALDH9A1;ortholog	4-TRIMETHYLAMINOBUTYRALDEHYDE DEHYDROGENASE (PTHR11699:SF228)	dehydrogenase(PC00092)	*Homo sapiens*
HUMAN|HGNC=7688|UniProtKB=Q16718	NDUA5	NADH dehydrogenase [ubiquinone] 1 alpha subcomplex subunit 5;NDUFA5;ortholog	NADH DEHYDROGENASE [UBIQUINONE] 1 ALPHA SUBCOMPLEX SUBUNIT 5 (PTHR12653:SF0)	oxidoreductase(PC00176)	*Homo sapiens*
HUMAN|HGNC=23316|UniProtKB=O14756	H17B6	17-beta-hydroxysteroid dehydrogenase type 6;HSD17B6;ortholog	17-BETA-HYDROXYSTEROID DEHYDROGENASE TYPE 6 (PTHR43313:SF4)	dehydrogenase(PC00092);reductase(PC00198)	*Homo sapiens*

HUMAN|HGNC=8768|UniProtKB=O95831	AIFM1	Apoptosis-inducing factor 1, mitochondrial;AIFM1;ortholog	APOPTOSIS-INDUCING FACTOR 1, MITOCHONDRIAL (PTHR43557:SF4)	dehydrogenase(PC00092);oxidase(PC00175);reductase(PC00198)	*Homo sapiens*
HUMAN|HGNC=26101|UniProtKB=Q96CM8	ACSF2	Acyl-CoA synthetase family member 2, mitochondrial;ACSF2;ortholog	ACYL-COA SYNTHETASE FAMILY MEMBER 2, MITOCHONDRIAL (PTHR43201:SF9)	dehydrogenase(PC00092);ligase(PC00142)	*Homo sapiens*
HUMAN|HGNC=28218|UniProtKB=Q9BRA2	TXD17	Thioredoxin domain-containing protein 17;TXNDC17;ortholog	THIOREDOXIN DOMAIN-CONTAINING PROTEIN 17 (PTHR12452:SF0)	transporter(PC00227)	*Homo sapiens*
HUMAN|HGNC=26920|UniProtKB=Q96G46	DUS3L	tRNA-dihydrouridine(47) synthase [NAD(P)(+)]-like;DUS3L;ortholog	TRNA-DIHYDROURIDINE(47) SYNTHASE [NAD(P)(+)]-LIKE (PTHR45846:SF1)		*Homo sapiens*
HUMAN|HGNC=20499|UniProtKB=Q9H9P8	L2HDH	L-2-hydroxyglutarate dehydrogenase, mitochondrial;L2HGDH;ortholog	L-2-HYDROXYGLUTARATE DEHYDROGENASE, MITOCHONDRIAL (PTHR43104:SF2)	dehydrogenase(PC00092);oxidase(PC00175)	*Homo sapiens*
HUMAN|HGNC=12590|UniProtKB=P07919	QCR6	Cytochrome b-c1 complex subunit 6, mitochondrial;UQCRH;ortholog	CYTOCHROME B-C1 COMPLEX SUBUNIT 6, MITOCHONDRIAL (PTHR15336:SF3)	reductase(PC00198)	*Homo sapiens*
HUMAN|HGNC=20667|UniProtKB=Q86VQ6	TRXR3	Thioredoxin reductase 3;TXNRD3;ortholog	THIOREDOXIN REDUCTASE 3 (PTHR43256:SF7)	dehydrogenase(PC00092);oxidase(PC00175);reductase(PC00198)	*Homo sapiens*
HUMAN|HGNC=11184|UniProtKB=Q00796	DHSO	Sorbitol dehydrogenase;SORD;ortholog	SORBITOL DEHYDROGENASE (PTHR43161:SF9)	dehydrogenase(PC00092);reductase(PC00198)	*Homo sapiens*
HUMAN|HGNC=9722|UniProtKB=P54886	P5CS	Delta-1-pyrroline-5-carboxylate synthase;ALDH18A1;ortholog	DELTA-1-PYRROLINE-5-CARBOXYLATE SYNTHASE (PTHR11063:SF8)	amino acid kinase(PC00045);dehydrogenase(PC00092)	*Homo sapiens*
HUMAN|HGNC=3012|UniProtKB=Q12882	DPYD	Dihydropyrimidine dehydrogenase [NADP(+)];DPYD;ortholog	DIHYDROPYRIMIDINE DEHYDROGENASE [NADP(+)] (PTHR43073:SF2)	dehydrogenase(PC00092);reductase(PC00198)	*Homo sapiens*

HUMAN|HGNC=23396|UniProtKB=Q5SRE7	PHYD1	Phytanoyl-CoA dioxygenase domain-containing protein 1;PHYHD1;ortholog	PHYTANOYL-COA DIOXYGENASE DOMAIN-CONTAINING PROTEIN 1 (PTHR20883:SF15)	oxidoreductase(PC00176)	*Homo sapiens*
HUMAN|HGNC=26506|UniProtKB=Q96MI6	PPM1E	Protein phosphatase 1M;PPM1M;ortholog	PROTEIN PHOSPHATASE 1M (PTHR13832:SF236)	kinase inhibitor(PC00139);protein phosphatase(PC00195)	*Homo sapiens*
HUMAN|HGNC=20797|UniProtKB=Q53TN4	CYBR1	Cytochrome b reductase 1;CYBRD1;ortholog	CYTOCHROME B REDUCTASE 1 (PTHR10106:SF12)	reductase(PC00198)	*Homo sapiens*
HUMAN|HGNC=30264|UniProtKB=Q8NCN5	PDPR	Pyruvate dehydrogenase phosphatase regulatory subunit, mitochondrial;PDPR;ortholog	PYRUVATE DEHYDROGENASE PHOSPHATASE REGULATORY SUBUNIT, MITOCHONDRIAL (PTHR13847:SF193)	dehydrogenase(PC00092);oxidase(PC00175)	*Homo sapiens*
HUMAN|HGNC=1613|UniProtKB=O14618	CCS	Copper chaperone for superoxide dismutase;CCS;ortholog	COPPER CHAPERONE FOR SUPEROXIDE DISMUTASE (PTHR10003:SF27)	oxidoreductase(PC00176)	*Homo sapiens*
HUMAN|HGNC=26398|UniProtKB=Q96NN9	AIFM3	Apoptosis-inducing factor 3;AIFM3;ortholog	APOPTOSIS-INDUCING FACTOR 3 (PTHR43557:SF8)	dehydrogenase(PC00092);oxidase(PC00175);reductase(PC00198)	*Homo sapiens*
HUMAN|HGNC=4236|UniProtKB=P55789	ALR	FAD-linked sulfhydryl oxidase ALR;GFER;ortholog	FAD-LINKED SULFHYDRYL OXIDASE ALR (PTHR12645:SF0)	oxidase(PC00175)	*Homo sapiens*
HUMAN|HGNC=12390|UniProtKB=Q13630	FCL	GDP-L-fucose synthase;TSTA3;ortholog	GDP-L-FUCOSE SYNTHASE (PTHR43238:SF1)	dehydratase(PC00091);epimerase/racemase(PC00096);oxidoreductase(PC00176)	*Homo sapiens*
HUMAN|HGNC=7419|UniProtKB=P00395	COX1	Cytochrome c oxidase subunit 1;MT-CO1;ortholog	CYTOCHROME C OXIDASE SUBUNIT 1 (PTHR10422:SF18)	oxidase(PC00175)	*Homo sapiens*
HUMAN|HGNC=1548|UniProtKB=P16152	CBR1	Carbonyl reductase [NADPH] 1;CBR1;ortholog	CARBONYL REDUCTASE [NADPH] 1 (PTHR43963:SF2)		*Homo sapiens*

HUMAN|HGNC=20390|UniProtKB=Q9Y6N5	SQOR	Sulfide:quinone oxidoreductase, mitochondrial;SQOR;ortholog	SULFIDE:QUINONE OXIDOREDUCTASE, MITOCHONDRIAL (PTHR10632:SF2)		*Homo sapiens*
HUMAN|HGNC=2593|UniProtKB=P05093	CP17A	Steroid 17-alpha-hydroxylase/17,20 lyase;CYP17A1;ortholog	STEROID 17-ALPHA-HYDROXYLASE/17,20 LYASE (PTHR24289:SF1)		*Homo sapiens*
HUMAN|HGNC=25812|UniProtKB=Q9H8P0	PORED	Polyprenol reductase;SRD5A3;ortholog	POLYPRENOL REDUCTASE (PTHR14624:SF0)		*Homo sapiens*
HUMAN|HGNC=7377|UniProtKB=Q9UJ68	MSRA	Mitochondrial peptide methionine sulfoxide reductase;MSRA;ortholog	MITOCHONDRIAL PEPTIDE METHIONINE SULFOXIDE REDUCTASE (PTHR42799:SF2)	reductase(PC00198)	*Homo sapiens*
HUMAN|HGNC=7714|UniProtKB=O75251	NDUS7	NADH dehydrogenase [ubiquinone] iron-sulfur protein 7, mitochondrial;NDUFS7;ortholog	NADH DEHYDROGENASE [UBIQUINONE] IRON-SULFUR PROTEIN 7, MITOCHONDRIAL (PTHR11995:SF22)	dehydrogenase(PC00092);reductase(PC00198)	*Homo sapiens*
HUMAN|HGNC=2321|UniProtKB=P36551	HEM6	Oxygen-dependent coproporphyrinogen-III oxidase, mitochondrial;CPOX;ortholog	OXYGEN-DEPENDENT COPROPORPHYRINOGEN-III OXIDASE, MITOCHONDRIAL (PTHR10755:SF0)	oxidase(PC00175)	*Homo sapiens*
HUMAN|HGNC=2267|UniProtKB=P20674	COX5A	Cytochrome c oxidase subunit 5A, mitochondrial;COX5A;ortholog	CYTOCHROME C OXIDASE SUBUNIT 5A, MITOCHONDRIAL (PTHR14200:SF13)	oxidase(PC00175)	*Homo sapiens*
HUMAN|HGNC=7872|UniProtKB=P29475	NOS1	Nitric oxide synthase, brain;NOS1;ortholog	NITRIC OXIDE SYNTHASE, BRAIN (PTHR19384:SF63)		*Homo sapiens*
HUMAN|HGNC=18583|UniProtKB=Q9ULR3	PPM1H	Protein phosphatase 1H;PPM1H;ortholog	PROTEIN PHOSPHATASE 1H (PTHR13832:SF287)	kinase inhibitor(PC00139);protein phosphatase(PC00195)	*Homo sapiens*
HUMAN|HGNC=17772|UniProtKB=Q99757	THIOM	Thioredoxin, mitochondrial;TXN2;ortholog	THIOREDOXIN, MITOCHONDRIAL (PTHR43601:SF3)	oxidoreductase(PC00176)	*Homo sapiens*

HUMAN|HGNC=7421|UniProtKB=P00403	COX2	Cytochrome c oxidase subunit 2;MT-CO2;ortholog	CYTOCHROME C OXIDASE SUBUNIT 2 (PTHR22888:SF9)	oxidoreductase(PC00176)	*Homo sapiens*
HUMAN|HGNC=20372|UniProtKB=Q9NX14	NDUBB	NADH dehydrogenase [ubiquinone] 1 beta subcomplex subunit 11, mitochondrial;NDUFB11;ortholog	NADH DEHYDROGENASE [UBIQUINONE] 1 BETA SUBCOMPLEX SUBUNIT 11, MITOCHONDRIAL (PTHR13327:SF0)	oxidoreductase(PC00176)	*Homo sapiens*
HUMAN|HGNC=30576|UniProtKB=Q9BV57	MTND	1,2-dihydroxy-3-keto-5-methylthiopentene dioxygenase;ADI1;ortholog	1,2-DIHYDROXY-3-KETO-5-METHYLTHIOPENTENE DIOXYGENASE (PTHR23418:SF0)	oxidoreductase(PC00176)	*Homo sapiens*
HUMAN|HGNC=7700|UniProtKB=O43674	NDUB5	NADH dehydrogenase [ubiquinone] 1 beta subcomplex subunit 5, mitochondrial;NDUFB5;ortholog	NADH DEHYDROGENASE [UBIQUINONE] 1 BETA SUBCOMPLEX SUBUNIT 5, MITOCHONDRIAL (PTHR13178:SF0)	oxidoreductase(PC00176)	*Homo sapiens*
HUMAN|HGNC=9021|UniProtKB=P14618	KPYM	Pyruvate kinase PKM;PKM;ortholog	PYRUVATE KINASE PKM (PTHR11817:SF15)		*Homo sapiens*
HUMAN|HGNC=406|UniProtKB=P30038	AL4A1	Delta-1-pyrroline-5-carboxylate dehydrogenase, mitochondrial;ALDH4A1;ortholog	DELTA-1-PYRROLINE-5-CARBOXYLATE DEHYDROGENASE, MITOCHONDRIAL (PTHR14516:SF3)		*Homo sapiens*
HUMAN|HGNC=10536|UniProtKB=Q9UL12	SARDH	Sarcosine dehydrogenase, mitochondrial;SARDH;ortholog	SARCOSINE DEHYDROGENASE, MITOCHONDRIAL (PTHR13847:SF200)	dehydrogenase(PC00092);oxidase(PC00175)	*Homo sapiens*
HUMAN|HGNC=30863|UniProtKB=Q9UDW1	QCR9	Cytochrome b-c1 complex subunit 9;UQCR10;ortholog	CYTOCHROME B-C1 COMPLEX SUBUNIT 9 (PTHR12980:SF0)	reductase(PC00198)	*Homo sapiens*
HUMAN|HGNC=13709|UniProtKB=O15121	DEGS1	Sphingolipid delta(4)-desaturase DES1;DEGS1;ortholog	SPHINGOLIPID DELTA(4)-DESATURASE DES1 (PTHR12879:SF2)		*Homo sapiens*
HUMAN|HGNC=28639|UniProtKB=Q6UWP2	DHR11	Dehydrogenase/reductase SDR family member 11;DHRS11;ortholog	DEHYDROGENASE/REDUCTASE SDR FAMILY MEMBER 11 (PTHR43115:SF4)	dehydrogenase(PC00092);reductase(PC00198)	*Homo sapiens*

HUMAN|HGNC=6229|UniProtKB=Q13303	KCAB2	Voltage-gated potassium channel subunit beta-2;KCNAB2;ortholog	VOLTAGE-GATED POTASSIUM CHANNEL SUBUNIT BETA-2 (PTHR43150:SF1)	reductase(PC00198);voltage-gated potassium channel(PC00242)	*Homo sapiens*
HUMAN|HGNC=877|UniProtKB=P49419	AL7A1	Alpha-aminoadipic semialdehyde dehydrogenase;ALDH7A1;ortholog	ALPHA-AMINOADIPIC SEMIALDEHYDE DEHYDROGENASE (PTHR43521:SF5)	dehydrogenase(PC00092)	*Homo sapiens*
HUMAN|HGNC=20134|UniProtKB=Q86SX6	GLRX5	Glutaredoxin-related protein 5, mitochondrial;GLRX5;ortholog	GLUTAREDOXIN-RELATED PROTEIN 5, MITOCHONDRIAL (PTHR10293:SF16)	reductase(PC00198)	*Homo sapiens*
HUMAN|HGNC=20233|UniProtKB=Q9Y2Z9	COQ6	Ubiquinone biosynthesis monooxygenase COQ6, mitochondrial;COQ6;ortholog	UBIQUINONE BIOSYNTHESIS MONOOXYGENASE COQ6, MITOCHONDRIAL (PTHR43876:SF7)	oxygenase(PC00177)	*Homo sapiens*
HUMAN|HGNC=29594|UniProtKB=O14949	QCR8	Cytochrome b-c1 complex subunit 8;UQCRQ;ortholog	CYTOCHROME B-C1 COMPLEX SUBUNIT 8 (PTHR12119:SF2)		*Homo sapiens*
HUMAN|HGNC=21577|UniProtKB=Q8NB78	KDM1B,LSD2	Lysine-specific histone demethylase 1B;KDM1B;ortholog	LYSINE-SPECIFIC HISTONE DEMETHYLASE 1B (PTHR10742:SF359)	DNA methyltransferase(PC00013);oxidase(PC00175)	*Homo sapiens*
HUMAN|HGNC=21063|UniProtKB=Q6UVY6	MOXD1	DBH-like monooxygenase protein 1;MOXD1;ortholog	DBH-LIKE MONOOXYGENASE PROTEIN 1 (PTHR10157:SF28)		*Homo sapiens*
HUMAN|HGNC=8903|UniProtKB=O95336	6PGL	6-phosphogluconolactonase;PGLS;ortholog	6-PHOSPHOGLUCONOLACTONASE (PTHR11054:SF0)	hydrolase(PC00121)	*Homo sapiens*
HUMAN|HGNC=18246|UniProtKB=Q9Y2S2	CRYL1	Lambda-crystallin homolog;CRYL1;ortholog	LAMBDA-CRYSTALLIN HOMOLOG (PTHR43492:SF2)	dehydrogenase(PC00092);epimerase/racemase(PC00096);hydratase(PC00120)	*Homo sapiens*
HUMAN|HGNC=17804|UniProtKB=Q9P0Z9	SOX	Peroxisomal sarcosine oxidase;PIPOX;ortholog	PEROXISOMAL SARCOSINE OXIDASE (PTHR10961:SF7)	oxidase(PC00175)	*Homo sapiens*

HUMAN|HGNC=7064|UniProtKB=O14880	MGST3	Microsomal glutathione S-transferase 3;MGST3;ortholog	MICROSOMAL GLUTATHIONE S-TRANSFERASE 3 (PTHR10250:SF17)	transferase(PC00220)	*Homo sapiens*
HUMAN|HGNC=16753|UniProtKB=P30041	PRDX6	Peroxiredoxin-6;PRDX6;ortholog	PEROXIREDOXIN-6 (PTHR43503:SF11)	peroxidase(PC00180)	*Homo sapiens*
HUMAN|HGNC=2595|UniProtKB=P04798	CP1A1	Cytochrome P450 1A1;CYP1A1;ortholog	CYTOCHROME P450 1A1 (PTHR24299:SF8)	oxygenase(PC00177)	*Homo sapiens*
HUMAN|HGNC=19708|UniProtKB=Q86WU2	LDHD	Probable D-lactate dehydrogenase, mitochondrial;LDHD;ortholog	D-LACTATE DEHYDROGENASE, MITOCHONDRIAL-RELATED (PTHR11748:SF35)	dehydrogenase(PC00092)	*Homo sapiens*
HUMAN|HGNC=9605|UniProtKB=P35354	COX2	Prostaglandin G/H synthase 2;PTGS2;ortholog	PROSTAGLANDIN G/H SYNTHASE 2 (PTHR11903:SF8)	oxygenase(PC00177)	*Homo sapiens*
HUMAN|HGNC=6381|UniProtKB=O15229	KMO	Kynurenine 3-monooxygenase;KMO;ortholog	KYNURENINE 3-MONOOXYGENASE (PTHR46028:SF2)		*Homo sapiens*
HUMAN|HGNC=121|UniProtKB=O15254	ACOX3	Peroxisomal acyl-coenzyme A oxidase 3;ACOX3;ortholog	PEROXISOMAL ACYL-COENZYME A OXIDASE 3 (PTHR10909:SF315)	dehydrogenase(PC00092);oxidase(PC00175);transferase(PC00220)	*Homo sapiens*
HUMAN|HGNC=13815|UniProtKB=Q9HAY6	BCDO1	Beta,beta-carotene 15,15'-dioxygenase;BCO1;ortholog	BETA,BETA-CAROTENE 15,15'-DIOXYGENASE (PTHR10543:SF34)	oxygenase(PC00177)	*Homo sapiens*
HUMAN|HGNC=8896|UniProtKB=P00558	PGK1	Phosphoglycerate kinase 1;PGK1;ortholog	PHOSPHOGLYCERATE KINASE 1 (PTHR11406:SF14)	carbohydrate kinase(PC00065)	*Homo sapiens*
HUMAN|HGNC=14355|UniProtKB=Q86YB8	ERO1B	ERO1-like protein beta;ERO1B;ortholog	ERO1-LIKE PROTEIN BETA (PTHR12613:SF2)	oxidoreductase(PC00176)	*Homo sapiens*

HUMAN|HGNC=4551|UniProtKB=Q9NZ01	TECR	Very-long-chain enoyl-CoA reductase;TECR;ortholog	VERY-LONG-CHAIN ENOYL-COA REDUCTASE (PTHR10556:SF31)	dehydrogenase(PC00092)	*Homo sapiens*
HUMAN|HGNC=3976|UniProtKB=P02794	FRIH	Ferritin heavy chain;FTH1;ortholog	FERRITIN HEAVY CHAIN (PTHR11431:SF37)	storage protein(PC00210)	*Homo sapiens*
HUMAN|HGNC=20968|UniProtKB=Q9H6W3	RIOX1	Ribosomal oxygenase 1;RIOX1;ortholog	RIBOSOMAL OXYGENASE 1 (PTHR13096:SF4)		*Homo sapiens*
HUMAN|HGNC=1027|UniProtKB=Q02338	BDH	D-beta-hydroxybutyrate dehydrogenase, mitochondrial;BDH1;ortholog	D-BETA-HYDROXYBUTYRATE DEHYDROGENASE, MITOCHONDRIAL (PTHR43313:SF25)	dehydrogenase(PC00092);reductase(PC00198)	*Homo sapiens*
HUMAN|HGNC=5211|UniProtKB=P37059	DHB2	Estradiol 17-beta-dehydrogenase 2;HSD17B2;ortholog	ESTRADIOL 17-BETA-DEHYDROGENASE 2 (PTHR43313:SF3)	dehydrogenase(PC00092);reductase(PC00198)	*Homo sapiens*
HUMAN|HGNC=16354|UniProtKB=Q8IWW8	HOT	Hydroxyacid-oxoacid transhydrogenase, mitochondrial;ADHFE1;ortholog	HYDROXYACID-OXOACID TRANSHYDROGENASE, MITOCHONDRIAL (PTHR11496:SF83)		*Homo sapiens*
HUMAN|HGNC=21497|UniProtKB=Q9H845	ACAD9	Acyl-CoA dehydrogenase family member 9, mitochondrial;ACAD9;ortholog	ACYL-COA DEHYDROGENASE FAMILY MEMBER 9, MITOCHONDRIAL (PTHR43884:SF9)	dehydrogenase(PC00092);oxidase(PC00175);transferase(PC00220)	*Homo sapiens*
HUMAN|HGNC=3062|UniProtKB=Q9NRD9	DUOX	Dual oxidase 1;DUOX1;ortholog	DUAL OXIDASE 1 (PTHR11972:SF75)	oxidase(PC00175)	*Homo sapiens*
HUMAN|HGNC=2418|UniProtKB=Q14894	CRYM	Ketimine reductase mu-crystallin;CRYM;ortholog	KETIMINE REDUCTASE MU-CRYSTALLIN (PTHR13812:SF19)	lyase(PC00144)	*Homo sapiens*
HUMAN|HGNC=14874|UniProtKB=Q96PH1	NOX5	NADPH oxidase 5;NOX5;ortholog	NADPH OXIDASE 5 (PTHR11972:SF58)	oxidase(PC00175)	*Homo sapiens*

HUMAN∣HGNC=6970∣UniProtKB=P40925	MDHC	Malate dehydrogenase, cytoplasmic;MDH1;ortholog	MALATE DEHYDROGENASE, CYTOPLASMIC (PTHR23382:SF3)	dehydrogenase(PC00092)	*Homo sapiens*
HUMAN∣HGNC=9280∣UniProtKB=P50336	PPOX	Protoporphyrinogen oxidase;PPOX;ortholog	PROTOPORPHYRINOGEN OXIDASE (PTHR42923:SF3)	DNA methyltransferase(PC00013);oxidase(PC00175)	*Homo sapiens*
HUMAN∣HGNC=2867∣UniProtKB=Q02127	PYRD	Dihydroorotate dehydrogenase (quinone), mitochondrial;DHODH;ortholog	DIHYDROOROTATE DEHYDROGENASE (QUINONE), MITOCHONDRIAL (PTHR43517:SF1)	dehydrogenase(PC00092);reductase(PC00198)	*Homo sapiens*
HUMAN∣HGNC=13273∣UniProtKB=Q9NRD8	DUOX2	Dual oxidase 2;DUOX2;ortholog	DUAL OXIDASE 2 (PTHR11972:SF67)	oxidase(PC00175)	*Homo sapiens*
HUMAN∣HGNC=2265∣UniProtKB=P13073	COX41	Cytochrome c oxidase subunit 4 isoform 1, mitochondrial;COX4I1;ortholog	CYTOCHROME C OXIDASE SUBUNIT 4 ISOFORM 1, MITOCHONDRIAL (PTHR10707:SF12)		*Homo sapiens*
HUMAN∣HGNC=20588∣UniProtKB=Q9UHG3	PCYOX	Prenylcysteine oxidase 1;PCYOX1;ortholog	PRENYLCYSTEINE OXIDASE 1 (PTHR15944:SF3)		*Homo sapiens*
HUMAN∣HGNC=17693∣UniProtKB=O75911	DHRS3	Short-chain dehydrogenase/reductase 3;DHRS3;ortholog	SHORT-CHAIN DEHYDROGENASE/REDUCTASE 3 (PTHR24322:SF483)		*Homo sapiens*
HUMAN∣HGNC=7708∣UniProtKB=O75306	NDUS2	NADH dehydrogenase [ubiquinone] iron-sulfur protein 2, mitochondrial;NDUFS2;ortholog	NADH DEHYDROGENASE [UBIQUINONE] IRON-SULFUR PROTEIN 2, MITOCHONDRIAL (PTHR11993:SF10)	dehydrogenase(PC00092);reductase(PC00198)	*Homo sapiens*

Lipases

HUMAN∣HGNC=24768∣UniProtKB=Q6ZV29	PLPL7	Patatin-like phospholipase domain-containing protein 7;PNPLA7;ortholog	PATATIN-LIKE PHOSPHOLIPASE DOMAIN-CONTAINING PROTEIN 7 (PTHR14226:SF23)	esterase(PC00097)	*Homo sapiens*

HUMAN|HGNC=16268|UniProtKB=Q8IY17	PLPL6	Neuropathy target esterase;PNPLA6;ortholog	NEUROPATHY TARGET ESTERASE (PTHR14226:SF26)	esterase(PC00097)	*Homo sapiens*
HUMAN|HGNC=9059|UniProtKB=Q15147	PLCB4	1-phosphatidylinositol 4,5-bisphosphate phosphodiesterase beta-4;PLCB4;ortholog	1-PHOSPHATIDYLINOSITOL 4,5-BISPHOSPHATE PHOSPHODIESTERASE BETA-4 (PTHR10336:SF106)	calcium-binding protein(PC00060);guanyl-nucleotide exchange factor(PC00113);phospholipase(PC00186);signaling molecule(PC00207)	*Homo sapiens*
HUMAN|HGNC=9062|UniProtKB=Q9BRC7	PLCD4	1-phosphatidylinositol 4,5-bisphosphate phosphodiesterase delta-4;PLCD4;ortholog	1-PHOSPHATIDYLINOSITOL 4,5-BISPHOSPHATE PHOSPHODIESTERASE DELTA-4 (PTHR10336:SF31)	guanyl-nucleotide exchange factor(PC00113);signaling molecule(PC00207)	*Homo sapiens*
HUMAN|HGNC=4390|UniProtKB=P50148	GNAQ	Guanine nucleotide-binding protein G(q) subunit alpha;GNAQ;ortholog	GUANINE NUCLEOTIDE-BINDING PROTEIN G(Q) SUBUNIT ALPHA (PTHR10218:SF318)	heterotrimeric G-protein(PC00117)	*Homo sapiens*
HUMAN|HGNC=18483|UniProtKB=Q8WWY8	LIPH	Lipase member H;LIPH;ortholog	LIPASE MEMBER H (PTHR11610:SF12)	esterase(PC00097);lipase(PC00143);storage protein(PC00210)	*Homo sapiens*
HUMAN|HGNC=5293|UniProtKB=P28223	5HT2A	5-hydroxytryptamine receptor 2A;HTR2A;ortholog	5-HYDROXYTRYPTAMINE RECEPTOR 2A (PTHR24247:SF30)	G-protein coupled receptor(PC00021)	*Homo sapiens*
HUMAN|HGNC=17175|UniProtKB=Q9P212	PLCE1	1-phosphatidylinositol 4,5-bisphosphate phosphodiesterase epsilon-1;PLCE1;ortholog	1-PHOSPHATIDYLINOSITOL 4,5-BISPHOSPHATE PHOSPHODIESTERASE EPSILON-1 (PTHR10336:SF6)	calcium-binding protein(PC00060);guanyl-nucleotide exchange factor(PC00113);phospholipase(PC00186);signaling molecule(PC00207)	*Homo sapiens*
HUMAN|HGNC=277|UniProtKB=P35348	ADA1A	Alpha-1A adrenergic receptor;ADRA1A;ortholog	ALPHA-1A ADRENERGIC RECEPTOR (PTHR24248:SF16)	G-protein coupled receptor(PC00021)	*Homo sapiens*
HUMAN|HGNC=9156|UniProtKB=P54315	LIPR1	Inactive pancreatic lipase-related protein 1;PNLIPRP1;ortholog	INACTIVE PANCREATIC LIPASE-RELATED PROTEIN 1 (PTHR11610:SF147)	esterase(PC00097);lipase(PC00143);storage protein(PC00210)	*Homo sapiens*

HUMAN|HGNC=6619|UniProtKB=P11150	LIPC	Hepatic triacylglycerol lipase;LIPC;ortholog	HEPATIC TRIACYLGLYCEROL LIPASE (PTHR11610:SF2)	esterase(PC00097);lipase(PC00143);storage protein(PC00210)	*Homo sapiens*
HUMAN|HGNC=17163|UniProtKB=Q8NCC3	PAG15	Group XV phospholipase A2;PLA2G15;ortholog	GROUP XV PHOSPHOLIPASE A2 (PTHR11440:SF47)	acyltransferase(PC00042);phospholipase(PC00186)	*Homo sapiens*
HUMAN|HGNC=17018|UniProtKB=Q9Y6Y8	S23IP	SEC23-interacting protein;SEC23IP;ortholog	SEC23-INTERACTING PROTEIN (PTHR23509:SF4)	membrane traffic protein(PC00150);phospholipase(PC00186)	*Homo sapiens*
HUMAN|HGNC=9155|UniProtKB=P16233	LIPP	Pancreatic triacylglycerol lipase;PNLIP;ortholog	PANCREATIC TRIACYLGLYCEROL LIPASE (PTHR11610:SF115)	esterase(PC00097);lipase(PC00143);storage protein(PC00210)	*Homo sapiens*
HUMAN|HGNC=9157|UniProtKB=P54317	LIPR2	Pancreatic lipase-related protein 2;PNLIPRP2;ortholog	PANCREATIC LIPASE-RELATED PROTEIN 2 (PTHR11610:SF85)	esterase(PC00097);lipase(PC00143);storage protein(PC00210)	*Homo sapiens*
HUMAN|HGNC=15520|UniProtKB=P43657	LPAR6	Lysophosphatidic acid receptor 6;LPAR6;ortholog	LYSOPHOSPHATIDIC ACID RECEPTOR 6 (PTHR24232:SF3)		*Homo sapiens*
HUMAN|HGNC=18718|UniProtKB=Q8WU67	ABHD3	Phospholipase ABHD3;ABHD3;ortholog	PHOSPHOLIPASE ABHD3 (PTHR10794:SF50)	serine protease(PC00203)	*Homo sapiens*
HUMAN|HGNC=278|UniProtKB=P35368	ADA1B	Alpha-1B adrenergic receptor;ADRA1B;ortholog	ALPHA-1B ADRENERGIC RECEPTOR (PTHR24248:SF17)	G-protein coupled receptor(PC00021)	*Homo sapiens*

Peptidase Activity

HUMAN|HGNC=500|UniProtKB=P15144	AMPN	Aminopeptidase N;ANPEP;ortholog	AMINOPEPTIDASE N (PTHR11533:SF172)	metalloprotease(PC00153)	*Homo sapiens*
HUMAN|HGNC=2524|UniProtKB=P40313	CTRL	Chymotrypsin-like protease CTRL-1;CTRL;ortholog	CHYMOTRYPSIN-LIKE PROTEASE CTRL-1 (PTHR24250:SF28)	serine protease(PC00203)	*Homo sapiens*
HUMAN|HGNC=9509|UniProtKB=P49810	PSN2	Presenilin-2;PSEN2;ortholog	PRESENILIN-2 (PTHR10202:SF26)	aspartic protease(PC00053);calcium-binding protein(PC00060);membrane-bound signaling molecule(PC00152)	*Homo sapiens*
HUMAN|HGNC=9344|UniProtKB=P42785	PCP	Lysosomal Pro-X carboxypeptidase;PRCP;ortholog	LYSOSOMAL PRO-X CARBOXYPEPTIDASE (PTHR11010:SF38)	serine protease(PC00203)	*Homo sapiens*
HUMAN|HGNC=25679|UniProtKB=Q9BSB4	ATGA1	Autophagy-related protein 101;ATG101;ortholog	AUTOPHAGY-RELATED PROTEIN 101 (PTHR13292:SF0)		*Homo sapiens*
HUMAN|HGNC=12623|UniProtKB=Q9UPU5	UBP24	Ubiquitin carboxyl-terminal hydrolase 24;USP24;ortholog	UBIQUITIN CARBOXYL-TERMINAL HYDROLASE 24 (PTHR24006:SF729)	cysteine protease(PC00081)	*Homo sapiens*
HUMAN|HGNC=25072|UniProtKB=Q5MNZ6	WIPI3	WD repeat domain phosphoinositide-interacting protein 3;WDR45B;ortholog	WD REPEAT DOMAIN PHOSPHOINOSITIDE-INTERACTING PROTEIN 3 (PTHR11227:SF18)		*Homo sapiens*
HUMAN|HGNC=18173|UniProtKB=Q9NZ08	ERAP1	Endoplasmic reticulum aminopeptidase 1;ERAP1;ortholog	ENDOPLASMIC RETICULUM AMINOPEPTIDASE 1 (PTHR11533:SF156)	metalloprotease(PC00153)	*Homo sapiens*
HUMAN|HGNC=589|UniProtKB=Q9H1Y0	ATG5	Autophagy protein 5;ATG5;ortholog	AUTOPHAGY PROTEIN 5 (PTHR13040:SF2)	membrane trafficking regulatory protein(PC00151)	*Homo sapiens*
HUMAN|HGNC=20635|UniProtKB=Q14997	PSME4	Proteasome activator complex subunit 4;PSME4;ortholog	PROTEASOME ACTIVATOR COMPLEX SUBUNIT 4 (PTHR32170:SF3)		*Homo sapiens*

HUMAN∣HGNC=11280∣UniProtKB=Q13501	SQSTM	Sequestosome-1;SQSTM1;ortholog	SEQUESTOSOME-1 (PTHR15090:SF0)		*Homo sapiens*
HUMAN∣HGNC=2301∣UniProtKB=O75976	CBPD	Carboxypeptidase D;CPD;ortholog	CARBOXYPEPTIDASE D (PTHR11532:SF57)	metalloprotease(PC00153)	*Homo sapiens*
HUMAN∣HGNC=20068∣UniProtKB=Q9H9J4	UBP42	Ubiquitin carboxyl-terminal hydrolase 42;USP42;ortholog	UBIQUITIN CARBOXYL-TERMINAL HYDROLASE 42 (PTHR24006:SF727)	cysteine protease(PC00081)	*Homo sapiens*
HUMAN∣HGNC=12666∣UniProtKB=P55072	TERA	Transitional endoplasmic reticulum ATPase;VCP;ortholog	TRANSITIONAL ENDOPLASMIC RETICULUM ATPASE (PTHR23077:SF69)		*Homo sapiens*
HUMAN∣HGNC=9532∣UniProtKB=P25788	PSA3	Proteasome subunit alpha type-3;PSMA3;ortholog	PROTEASOME SUBUNIT ALPHA TYPE-3 (PTHR11599:SF10)	protease(PC00190)	*Homo sapiens*
HUMAN∣HGNC=12632∣UniProtKB=Q93008	USP9X	Probable ubiquitin carboxyl-terminal hydrolase FAF-X;USP9X;ortholog	UBIQUITIN CARBOXYL-TERMINAL HYDROLASE FAF-X-RELATED (PTHR24006:SF732)	cysteine protease(PC00081)	*Homo sapiens*
HUMAN∣HGNC=588∣UniProtKB=O94817	ATG12	Ubiquitin-like protein ATG12;ATG12;ortholog	UBIQUITIN-LIKE PROTEIN ATG12 (PTHR13385:SF0)		*Homo sapiens*
HUMAN∣HGNC=12624∣UniProtKB=Q9UHP3	UBP25	Ubiquitin carboxyl-terminal hydrolase 25;USP25;ortholog	UBIQUITIN CARBOXYL-TERMINAL HYDROLASE 25 (PTHR24006:SF666)	cysteine protease(PC00081)	*Homo sapiens*
HUMAN∣HGNC=29169∣UniProtKB=Q9Y2L5	TPPC8	Trafficking protein particle complex subunit 8;TRAPPC8;ortholog	TRAFFICKING PROTEIN PARTICLE COMPLEX SUBUNIT 8 (PTHR12975:SF6)		*Homo sapiens*
HUMAN∣HGNC=1361∣UniProtKB=Q8N6M6	AMPO	Aminopeptidase O;AOPEP;ortholog	AMINOPEPTIDASE O (PTHR46627:SF1)		*Homo sapiens*

HUMAN|HGNC=9531|UniProtKB=P25787	PSA2	Proteasome subunit alpha type-2;PSMA2;ortholog	PROTEASOME SUBUNIT ALPHA TYPE-2 (PTHR11599:SF16)	protease(PC00190)	*Homo sapiens*
HUMAN|HGNC=21498|UniProtKB=Q676U5	A16L1	Autophagy-related protein 16-1;ATG16L1;ortholog	AUTOPHAGY-RELATED PROTEIN 16-1 (PTHR19878:SF6)		*Homo sapiens*
HUMAN|HGNC=20944|UniProtKB=Q9GZR1	SENP6	Sentrin-specific protease 6;SENP6;ortholog	SENTRIN-SPECIFIC PROTEASE 6 (PTHR46896:SF1)	cysteine protease(PC00081)	*Homo sapiens*
HUMAN|HGNC=30227|UniProtKB=Q8TCT8	SPP2A	Signal peptide peptidase-like 2A;SPPL2A;ortholog	SIGNAL PEPTIDE PEPTIDASE-LIKE 2A (PTHR12174:SF34)		*Homo sapiens*
HUMAN|HGNC=15859|UniProtKB=Q9H6P5	TASP1	Threonine aspartase 1;TASP1;ortholog	THREONINE ASPARTASE 1 (PTHR10188:SF8)	protease(PC00190)	*Homo sapiens*
HUMAN|HGNC=15991|UniProtKB=Q96KR4	LMLN	Leishmanolysin-like peptidase;LMLN;ortholog	LEISHMANOLYSIN-LIKE PEPTIDASE (PTHR10942:SF0)	cell adhesion molecule(PC00069);metalloprotease(PC00153)	*Homo sapiens*
HUMAN|HGNC=28430|UniProtKB=Q9BV81	EMC6	ER membrane protein complex subunit 6;EMC6;ortholog	ER MEMBRANE PROTEIN COMPLEX SUBUNIT 6 (PTHR20994:SF0)		*Homo sapiens*
HUMAN|HGNC=4248|UniProtKB=Q92820	GGH	Gamma-glutamyl hydrolase;GGH;ortholog	GAMMA-GLUTAMYL HYDROLASE (PTHR11315:SF0)	cysteine protease(PC00081)	*Homo sapiens*
HUMAN|HGNC=16702|UniProtKB=Q9BT78	CSN4	COP9 signalosome complex subunit 4;COPS4;ortholog	COP9 SIGNALOSOME COMPLEX SUBUNIT 4 (PTHR10855:SF2)		*Homo sapiens*
HUMAN|HGNC=586|UniProtKB=P13798	ACPH	Acylamino-acid-releasing enzyme;APEH;ortholog	ACYLAMINO-ACID-RELEASING ENZYME (PTHR42776:SF4)	serine protease(PC00203)	*Homo sapiens*

HUMAN|HGNC=11820|UniProtKB=P01033	TIMP1	Metalloproteinase inhibitor 1;TIMP1;ortholog	METALLOPROTEINASE INHIBITOR 1 (PTHR11844:SF20)	protease inhibitor(PC00191)	*Homo sapiens*
HUMAN|HGNC=2240|UniProtKB=Q92905	CSN5	COP9 signalosome complex subunit 5;COPS5;ortholog	COP9 SIGNALOSOME COMPLEX SUBUNIT 5 (PTHR10410:SF6)	metalloprotease(PC00153);transcription factor(PC00218)	*Homo sapiens*
HUMAN|HGNC=27912|UniProtKB=Q6ZMM2	ATL5	ADAMTS-like protein 5;ADAMTSL5;ortholog	ADAMTS-LIKE PROTEIN 5 (PTHR13723:SF173)	extracellular matrix glycoprotein(PC00100);metalloprotease(PC00153);serine protease inhibitor(PC00204)	*Homo sapiens*
HUMAN|HGNC=14348|UniProtKB=O43464	HTRA2	Serine protease HTRA2, mitochondrial;HTRA2;ortholog	SERINE PROTEASE HTRA2, MITOCHONDRIAL (PTHR22939:SF109)	chaperone(PC00072);serine protease(PC00203)	*Homo sapiens*
HUMAN|HGNC=1499|UniProtKB=P29466	CASP1	Caspase-1;CASP1;ortholog	CASPASE-1 (PTHR10454:SF216)	cysteine protease(PC00081);protease inhibitor(PC00191)	*Homo sapiens*
HUMAN|HGNC=15722|UniProtKB=Q8WXQ8	CBPA5	Carboxypeptidase A5;CPA5;ortholog	CARBOXYPEPTIDASE A5 (PTHR11705:SF16)	metalloprotease(PC00153)	*Homo sapiens*
HUMAN|HGNC=14605|UniProtKB=P58397	ATS12	A disintegrin and metalloproteinase with thrombospondin motifs 12;ADAMTS12;ortholog	A DISINTEGRIN AND METALLOPROTEINASE WITH THROMBOSPONDIN MOTIFS 12 (PTHR13723:SF189)	extracellular matrix glycoprotein(PC00100);metalloprotease(PC00153);serine protease inhibitor(PC00204)	*Homo sapiens*
HUMAN|HGNC=6746|UniProtKB=Q14596	NBR1	Next to BRCA1 gene 1 protein;NBR1;ortholog	NEXT TO BRCA1 GENE 1 PROTEIN (PTHR20930:SF2)		*Homo sapiens*
HUMAN|HGNC=24969|UniProtKB=Q8WTW4	NPRL2	GATOR complex protein NPRL2;NPRL2;ortholog	GATOR COMPLEX PROTEIN NPRL2 (PTHR12991:SF10)	nuclease(PC00170);transcription factor(PC00218)	*Homo sapiens*

HUMAN|HGNC=20492|UniProtKB=Q96IL0	APOP1	Apoptogenic protein 1, mitochondrial;APOPT1;ortholog	APOPTOGENIC PROTEIN 1, MITOCHONDRIAL (PTHR31107:SF2)		*Homo sapiens*
HUMAN|HGNC=14124|UniProtKB=Q12980	NPRL3	GATOR complex protein NPRL3;NPRL3;ortholog	GATOR COMPLEX PROTEIN NPRL3 (PTHR13153:SF5)		*Homo sapiens*
HUMAN|HGNC=9530|UniProtKB=P25786	PSA1	Proteasome subunit alpha type-1;PSMA1;ortholog	PROTEASOME SUBUNIT ALPHA TYPE-RELATED (PTHR11599:SF12)	protease(PC00190)	*Homo sapiens*
HUMAN|HGNC=3942|UniProtKB=P42345	MTOR	Serine/threonine-protein kinase mTOR;MTOR;ortholog	SERINE/THREONINE-PROTEIN KINASE MTOR (PTHR11139:SF9)	non-receptor serine/threonine protein kinase(PC00167);nucleic acid binding(PC00171);nucleotide kinase(PC00172)	*Homo sapiens*
HUMAN|HGNC=3355|UniProtKB=Q07075	AMPE	Glutamyl aminopeptidase;ENPEP;ortholog	GLUTAMYL AMINOPEPTIDASE (PTHR11533:SF269)	metalloprotease(PC00153)	*Homo sapiens*
HUMAN|HGNC=9533|UniProtKB=P25789	PSA4	Proteasome subunit alpha type-4;PSMA4;ortholog	PROTEASOME SUBUNIT ALPHA TYPE-4 (PTHR11599:SF13)	protease(PC00190)	*Homo sapiens*
HUMAN|HGNC=9559|UniProtKB=Q13200	PSMD2	26S proteasome non-ATPase regulatory subunit 2;PSMD2;ortholog	26S PROTEASOME NON-ATPASE REGULATORY SUBUNIT 2 (PTHR10943:SF15)	enzyme modulator(PC00095)	*Homo sapiens*
HUMAN|HGNC=590|UniProtKB=Q13490	BIRC2	Baculoviral IAP repeat-containing protein 2;BIRC2;ortholog	BACULOVIRAL IAP REPEAT-CONTAINING PROTEIN 2 (PTHR10044:SF79)	protease inhibitor(PC00191)	*Homo sapiens*
HUMAN|HGNC=9358|UniProtKB=P48147	PPCE, PREP, PEP	Prolyl endopeptidase;PREP;ortholog	PROLYL ENDOPEPTIDASE (PTHR42881:SF2)	serine protease(PC00203)	*Homo sapiens*

HUMAN|HGNC=7160|UniProtKB=P50281	MMP14	Matrix metalloproteinase-14;MMP14;ortholog	MATRIX METALLOPROTEINASE-14 (PTHR10201:SF24)	metalloprotease(PC00153)	*Homo sapiens*
HUMAN|HGNC=21408|UniProtKB=Q8IYS1	P20D2	Peptidase M20 domain-containing protein 2;PM20D2;ortholog	PEPTIDASE M20 DOMAIN-CONTAINING PROTEIN 2 (PTHR30575:SF0)	metalloprotease(PC00153);oxidoreductase(PC00176)	*Homo sapiens*
HUMAN|HGNC=3008|UniProtKB=Q9NY33	DPP3	Dipeptidyl peptidase 3;DPP3;ortholog	DIPEPTIDYL PEPTIDASE 3 (PTHR23422:SF11)	metalloprotease(PC00153)	*Homo sapiens*
HUMAN|HGNC=9119|UniProtKB=O75439	MPPB	Mitochondrial-processing peptidase subunit beta;PMPCB;ortholog	MITOCHONDRIAL-PROCESSING PEPTIDASE SUBUNIT BETA (PTHR11851:SF103)	metalloprotease(PC00153)	*Homo sapiens*
HUMAN|HGNC=10701|UniProtKB=Q15436	SC23A	Protein transport protein Sec23A;SEC23A;ortholog	PROTEIN TRANSPORT PROTEIN SEC23A (PTHR11141:SF7)	G-protein modulator(PC00022)	*Homo sapiens*
HUMAN|HGNC=25402|UniProtKB=Q96G74	OTUD5	OTU domain-containing protein 5;OTUD5;ortholog	OTU DOMAIN-CONTAINING PROTEIN 5 (PTHR12419:SF4)	cysteine protease(PC00081);nucleic acid binding(PC00171)	*Homo sapiens*
HUMAN|HGNC=29331|UniProtKB=Q9HCE0	EPG5	Ectopic P granules protein 5 homolog;EPG5;ortholog	ECTOPIC P GRANULES PROTEIN 5 HOMOLOG (PTHR31139:SF4)		*Homo sapiens*
HUMAN|HGNC=30627|UniProtKB=Q8TCT7	SPP2B	Signal peptide peptidase-like 2B;SPPL2B;ortholog	SIGNAL PEPTIDE PEPTIDASE-LIKE 2B (PTHR12174:SF39)		*Homo sapiens*
HUMAN|HGNC=28912|UniProtKB=Q9Y484	WIPI4	WD repeat domain phosphoinositide-interacting protein 4;WDR45;ortholog	WD REPEAT DOMAIN PHOSPHOINOSITIDE-INTERACTING PROTEIN 4 (PTHR11227:SF44)		*Homo sapiens*
HUMAN|HGNC=13557|UniProtKB=Q9BYF1	ACE2	Angiotensin-converting enzyme 2;ACE2;ortholog	ANGIOTENSIN-CONVERTING ENZYME 2 (PTHR10514:SF24)	metalloprotease(PC00153)	*Homo sapiens*

HUMAN|HGNC=20962|UniProtKB=Q9NT62	ATG3	Ubiquitin-like-conjugating enzyme ATG3;ATG3;ortholog	UBIQUITIN-LIKE-CONJUGATING ENZYME ATG3 (PTHR12866:SF2)	ligase(PC00142)	*Homo sapiens*
HUMAN|HGNC=9540|UniProtKB=P49720	PSB3	Proteasome subunit beta type-3;PSMB3;ortholog	PROTEASOME SUBUNIT BETA TYPE-3 (PTHR11599:SF62)	protease(PC00190)	*Homo sapiens*
HUMAN|HGNC=28962|UniProtKB=Q15005	SPCS2,SPC25	Signal peptidase complex subunit 2;SPCS2;ortholog	SIGNAL PEPTIDASE COMPLEX SUBUNIT 2 (PTHR13085:SF2)	enzyme modulator(PC00095)	*Homo sapiens*
HUMAN|HGNC=2482|UniProtKB=P04080	CYTB	Cystatin-B;CSTB;ortholog	CYSTATIN-B (PTHR11414:SF22)	cysteine protease inhibitor(PC00082)	*Homo sapiens*
HUMAN|HGNC=2311|UniProtKB=P14384	CBPM	Carboxypeptidase M;CPM;ortholog	CARBOXYPEPTIDASE M (PTHR11532:SF84)	metalloprotease(PC00153)	*Homo sapiens*
HUMAN|HGNC=12628|UniProtKB=P45974	UBP5	Ubiquitin carboxyl-terminal hydrolase 5;USP5;ortholog	UBIQUITIN CARBOXYL-TERMINAL HYDROLASE 5 (PTHR24006:SF655)	cysteine protease(PC00081)	*Homo sapiens*
HUMAN|HGNC=19962|UniProtKB=Q6ZNE5	BAKOR	Beclin 1-associated autophagy-related key regulator;ATG14;ortholog	BECLIN 1-ASSOCIATED AUTOPHAGY-RELATED KEY REGULATOR (PTHR13664:SF0)		*Homo sapiens*
HUMAN|HGNC=24281|UniProtKB=Q8N6M0	OTU6B	OTU domain-containing protein 6B;OTUD6B;ortholog	OTU DOMAIN-CONTAINING PROTEIN 6B (PTHR12419:SF21)	cysteine protease(PC00081);nucleic acid binding(PC00171)	*Homo sapiens*
HUMAN|HGNC=9472|UniProtKB=Q99538	LGMN	Legumain;LGMN;ortholog	LEGUMAIN (PTHR12000:SF38)	cysteine protease(PC00081)	*Homo sapiens*
HUMAN|HGNC=2088|UniProtKB=O76031	CLPX	ATP-dependent Clp protease ATP-binding subunit clpX-like, mitochondrial;CLPX;ortholog	ATP-DEPENDENT CLP PROTEASE ATP-BINDING SUBUNIT CLPX-LIKE, MITOCHONDRIAL (PTHR11262:SF4)	chaperone(PC00072)	*Homo sapiens*

HUMAN|HGNC=1509|UniProtKB=Q14790	CASP8	Caspase-8;CASP8;ortholog	CASPASE-8 (PTHR10454:SF162)	cysteine protease(PC00081);protease inhibitor(PC00191)	*Homo sapiens*
HUMAN|HGNC=9541|UniProtKB=P28070	PSB4	Proteasome subunit beta type-4;PSMB4;ortholog	PROTEASOME SUBUNIT BETA TYPE-4 (PTHR11599:SF5)	protease(PC00190)	*Homo sapiens*
HUMAN|HGNC=26212|UniProtKB=P61009	SPCS3	Signal peptidase complex subunit 3;SPCS3;ortholog	SIGNAL PEPTIDASE COMPLEX SUBUNIT 3 (PTHR12804:SF0)	protease(PC00190)	*Homo sapiens*
HUMAN|HGNC=9251|UniProtKB=P10619	PPGB	Lysosomal protective protein;CTSA;ortholog	LYSOSOMAL PROTECTIVE PROTEIN (PTHR11802:SF293)	serine protease(PC00203)	*Homo sapiens*
HUMAN|HGNC=29507|UniProtKB=Q9HB40	RISC, SCP1	Retinoid-inducible serine carboxypeptidase;SCPEP1;ortholog	RETINOID-INDUCIBLE SERINE CARBOXYPEPTIDASE (PTHR11802:SF3)	serine protease(PC00203)	*Homo sapiens*
HUMAN|HGNC=15912|UniProtKB=Q9UNZ2	NSF1C	NSFL1 cofactor p47;NSFL1C;ortholog	NSFL1 COFACTOR P47 (PTHR23333:SF24)	membrane trafficking regulatory protein(PC00151)	*Homo sapiens*
HUMAN|HGNC=2707|UniProtKB=P12821	ACE	Angiotensin-converting enzyme;ACE;ortholog	ANGIOTENSIN-CONVERTING ENZYME (PTHR10514:SF25)	metalloprotease(PC00153)	*Homo sapiens*
HUMAN|HGNC=18533|UniProtKB=Q86UV5	UBP48	Ubiquitin carboxyl-terminal hydrolase 48;USP48;ortholog	UBIQUITIN CARBOXYL-TERMINAL HYDROLASE 48 (PTHR24006:SF722)		*Homo sapiens*
HUMAN|HGNC=1508|UniProtKB=P55210	CASP7	Caspase-7;CASP7;ortholog	CASPASE-7 (PTHR10454:SF31)	cysteine protease(PC00081);protease inhibitor(PC00191)	*Homo sapiens*
HUMAN|HGNC=9534|UniProtKB=P28066	PSA5	Proteasome subunit alpha type-5;PSMA5;ortholog	PROTEASOME SUBUNIT ALPHA TYPE-5 (PTHR11599:SF14)	protease(PC00190)	*Homo sapiens*

HUMAN|HGNC=8974|UniProtKB=Q8NEB9	PK3C3	Phosphatidylinositol 3-kinase catalytic subunit type 3;PIK3C3;ortholog	PHOSPHATIDYLINOSITOL 3-KINASE CATALYTIC SUBUNIT TYPE 3 (PTHR10048:SF7)	kinase(PC00137)	*Homo sapiens*
HUMAN|HGNC=11345|UniProtKB=O95980	RECK	Reversion-inducing cysteine-rich protein with Kazal motifs;RECK;ortholog	REVERSION-INDUCING CYSTEINE-RICH PROTEIN WITH KAZAL MOTIFS (PTHR13487:SF3)	protease inhibitor(PC00191)	*Homo sapiens*
HUMAN|HGNC=20066|UniProtKB=Q70CQ2	UBP34	Ubiquitin carboxyl-terminal hydrolase 34;USP34;ortholog	UBIQUITIN CARBOXYL-TERMINAL HYDROLASE 34 (PTHR24006:SF671)	cysteine protease(PC00081)	*Homo sapiens*
HUMAN|HGNC=29028|UniProtKB=Q2TAZ0	ATG2A	Autophagy-related protein 2 homolog A;ATG2A;ortholog	AUTOPHAGY-RELATED PROTEIN 2 HOMOLOG A (PTHR13190:SF21)		*Homo sapiens*
HUMAN|HGNC=9537|UniProtKB=P20618	PSB1	Proteasome subunit beta type-1;PSMB1;ortholog	PROTEASOME SUBUNIT BETA TYPE-1 (PTHR11599:SF59)	protease(PC00190)	*Homo sapiens*
HUMAN|HGNC=30748|UniProtKB=Q9UKU6	TRHDE	Thyrotropin-releasing hormone-degrading ectoenzyme;TRHDE;ortholog	THYROTROPIN-RELEASING HORMONE-DEGRADING ECTOENZYME (PTHR11533:SF40)	metalloprotease(PC00153)	*Homo sapiens*
HUMAN|HGNC=318|UniProtKB=P20933	ASPG	N(4)-(beta-N-acetylglucosaminyl)-L-asparaginase;AGA;ortholog	N(4)-(BETA-N-ACETYLGLUCOSAMINYL)-L-ASPARAGINASE (PTHR10188:SF6)	protease(PC00190)	*Homo sapiens*
HUMAN|HGNC=12822|UniProtKB=Q9NQW7	XPP1	Xaa-Pro aminopeptidase 1;XPNPEP1;ortholog	XAA-PRO AMINOPEPTIDASE 1 (PTHR43763:SF6)	metalloprotease(PC00153);nucleic acid binding(PC00171);transcription factor(PC00218)	*Homo sapiens*
HUMAN|HGNC=12608|UniProtKB=Q14694	UBP10	Ubiquitin carboxyl-terminal hydrolase 10;USP10;ortholog	UBIQUITIN CARBOXYL-TERMINAL HYDROLASE 10 (PTHR24006:SF687)	cysteine protease(PC00081)	*Homo sapiens*
HUMAN|HGNC=3535|UniProtKB=P00734	THRB	Prothrombin;F2;ortholog	PROTHROMBIN (PTHR24254:SF10)	serine protease(PC00203)	*Homo sapiens*

HUMAN|HGNC=18667|UniProtKB=Q10713	MPPA	Mitochondrial-processing peptidase subunit alpha;PMPCA;ortholog	MITOCHONDRIAL-PROCESSING PEPTIDASE SUBUNIT ALPHA (PTHR11851:SF190)	metalloprotease(PC00153)	*Homo sapiens*
HUMAN|HGNC=15759|UniProtKB=Q16186	ADRM1	Proteasomal ubiquitin receptor ADRM1;ADRM1;ortholog	PROTEASOMAL UBIQUITIN RECEPTOR ADRM1 (PTHR12225:SF0)		*Homo sapiens*
HUMAN|HGNC=9544|UniProtKB=Q99436	PSB7	Proteasome subunit beta type-7;PSMB7;ortholog	PROTEASOME SUBUNIT BETA TYPE-7 (PTHR11599:SF42)	protease(PC00190)	*Homo sapiens*
HUMAN|HGNC=9758|UniProtKB=P62820	RAB1A	Ras-related protein Rab-1A;RAB1A;ortholog	RAS-RELATED PROTEIN RAB-1A (PTHR24073:SF999)		*Homo sapiens*
HUMAN|HGNC=20187|UniProtKB=Q96BY7	ATG2B	Autophagy-related protein 2 homolog B;ATG2B;ortholog	AUTOPHAGY-RELATED PROTEIN 2 HOMOLOG B (PTHR13190:SF20)		*Homo sapiens*
HUMAN|HGNC=7900|UniProtKB=P55786	PSA	Puromycin-sensitive aminopeptidase;NPEPPS;ortholog	PUROMYCIN-SENSITIVE AMINOPEPTIDASE-RELATED (PTHR11533:SF261)	metalloprotease(PC00153)	*Homo sapiens*
HUMAN|HGNC=20076|UniProtKB=Q96K76	UBP47	Ubiquitin carboxyl-terminal hydrolase 47;USP47;ortholog	UBIQUITIN CARBOXYL-TERMINAL HYDROLASE 47 (PTHR24006:SF702)	cysteine protease(PC00081)	*Homo sapiens*
HUMAN|HGNC=9479|UniProtKB=P36776	LONM	Lon protease homolog, mitochondrial;LONP1;ortholog	LON PROTEASE HOMOLOG, MITOCHONDRIAL (PTHR43718:SF2)	serine protease(PC00203)	*Homo sapiens*
HUMAN|HGNC=9543|UniProtKB=P28072	PSB6	Proteasome subunit beta type-6;PSMB6;ortholog	PROTEASOME SUBUNIT BETA TYPE-6 (PTHR11599:SF46)	protease(PC00190)	*Homo sapiens*
HUMAN|HGNC=23080|UniProtKB=Q7RTY7	OVCH1	Ovochymase-1;OVCH1;ortholog	OVOCHYMASE-1 (PTHR24251:SF21)	serine protease(PC00203)	*Homo sapiens*

HUMAN|HGNC=16889|UniProtKB=O00487	PSDE	26S proteasome non-ATPase regulatory subunit 14;PSMD14;ortholog	26S PROTEASOME NON-ATPASE REGULATORY SUBUNIT 14 (PTHR10410:SF5)	metalloprotease(PC00153);transcription factor(PC00218)	*Homo sapiens*
HUMAN|HGNC=22408|UniProtKB=Q7Z3C6	ATG9A	Autophagy-related protein 9A;ATG9A;ortholog	AUTOPHAGY-RELATED PROTEIN 9A (PTHR13038:SF13)		*Homo sapiens*
HUMAN|HGNC=24554|UniProtKB=Q6UXH9	PAMR1	Inactive serine protease PAMR1;PAMR1;ortholog	INACTIVE SERINE PROTEASE PAMR1 (PTHR24254:SF9)	serine protease(PC00203)	*Homo sapiens*
HUMAN|HGNC=20069|UniProtKB=Q9NVE5	UBP40	Ubiquitin carboxyl-terminal hydrolase 40;USP40;ortholog	UBIQUITIN CARBOXYL-TERMINAL HYDROLASE 40 (PTHR24006:SF704)		*Homo sapiens*
HUMAN|HGNC=7162|UniProtKB=P51512	MMP16	Matrix metalloproteinase-16;MMP16;ortholog	MATRIX METALLOPROTEINASE-16 (PTHR10201:SF26)	metalloprotease(PC00153)	*Homo sapiens*
HUMAN|HGNC=32583|UniProtKB=Q6UB28	MAP12	Methionine aminopeptidase 1D, mitochondrial;METAP1D;ortholog	METHIONINE AMINOPEPTIDASE 1D, MITOCHONDRIAL (PTHR43330:SF8)	metalloprotease(PC00153);nucleic acid binding(PC00171);transcription factor(PC00218)	*Homo sapiens*
HUMAN|HGNC=16435|UniProtKB=Q8TCT9	HM13	Minor histocompatibility antigen H13;HM13;ortholog	MINOR HISTOCOMPATIBILITY ANTIGEN H13 (PTHR12174:SF23)	membrane-bound signaling molecule(PC00152)	*Homo sapiens*
HUMAN|HGNC=3273|UniProtKB=O15372	EIF3H	Eukaryotic translation initiation factor 3 subunit H;EIF3H;ortholog	EUKARYOTIC TRANSLATION INITIATION FACTOR 3 SUBUNIT H (PTHR10410:SF3)	metalloprotease(PC00153);transcription factor(PC00218)	*Homo sapiens*
HUMAN|HGNC=19168|UniProtKB=Q9GZM7	TINAL	Tubulointerstitial nephritis antigen-like;TINAGL1;ortholog	TUBULOINTERSTITIAL NEPHRITIS ANTIGEN-LIKE (PTHR12411:SF270)	cysteine protease(PC00081)	*Homo sapiens*
HUMAN|HGNC=9570|UniProtKB=P61289	PSME3	Proteasome activator complex subunit 3;PSME3;ortholog	PROTEASOME ACTIVATOR COMPLEX SUBUNIT 3 (PTHR10660:SF4)		*Homo sapiens*

HUMAN|HGNC=1480|UniProtKB=P20807	NCL1	Calpain-3;CAPN3;ortholog	CALPAIN-3 (PTHR10183:SF329)	annexin(PC00050);calmodulin(PC00061);cysteine protease(PC00081)	*Homo sapiens*
HUMAN|HGNC=7157|UniProtKB=P24347	MMP11	Stromelysin-3;MMP11;ortholog	STROMELYSIN-3 (PTHR10201:SF20)	metalloprotease(PC00153)	*Homo sapiens*
HUMAN|HGNC=16856|UniProtKB=Q14674	ESPL1	Separin;ESPL1;ortholog	SEPARIN (PTHR12792:SF0)	cysteine protease(PC00081);nucleic acid binding(PC00171)	*Homo sapiens*
HUMAN|HGNC=19262|UniProtKB=O95428	PPN	Papilin;PAPLN;ortholog	PAPILIN (PTHR13723:SF179)	extracellular matrix glycoprotein(PC00100);metalloprotease(PC00153);serine protease inhibitor(PC00204)	*Homo sapiens*
HUMAN|HGNC=9542|UniProtKB=P28074	PSB5	Proteasome subunit beta type-5;PSMB5;ortholog	PROTEASOME SUBUNIT BETA TYPE-5 (PTHR11599:SF51)	protease(PC00190)	*Homo sapiens*
HUMAN|HGNC=9536|UniProtKB=O14818	PSA7	Proteasome subunit alpha type-7;PSMA7;ortholog	PROTEASOME SUBUNIT ALPHA TYPE-7 (PTHR11599:SF40)	protease(PC00190)	*Homo sapiens*
HUMAN|HGNC=29091|UniProtKB=O75143	ATG13	Autophagy-related protein 13;ATG13;ortholog	AUTOPHAGY-RELATED PROTEIN 13 (PTHR13430:SF4)		*Homo sapiens*
HUMAN|HGNC=19703|UniProtKB=Q6PHR2	ULK3	Serine/threonine-protein kinase ULK3;ULK3;ortholog	SERINE/THREONINE-PROTEIN KINASE ULK3 (PTHR24348:SF43)	non-receptor serine/threonine protein kinase(PC00167)	*Homo sapiens*
HUMAN|HGNC=9554|UniProtKB=Q99460	PSMD1	26S proteasome non-ATPase regulatory subunit 1;PSMD1;ortholog	26S PROTEASOME NON-ATPASE REGULATORY SUBUNIT 1 (PTHR10943:SF2)	enzyme modulator(PC00095)	*Homo sapiens*

HUMAN|HGNC=32225|UniProtKB=Q9Y4P8	WIPI2	WD repeat domain phosphoinositide-interacting protein 2;WIPI2;ortholog	WD REPEAT DOMAIN PHOSPHOINOSITIDE-INTERACTING PROTEIN 2 (PTHR11227:SF27)		*Homo sapiens*
HUMAN|HGNC=23400|UniProtKB=Q9BY50	SC11C	Signal peptidase complex catalytic subunit SEC11C;SEC11C;ortholog	SIGNAL PEPTIDASE COMPLEX CATALYTIC SUBUNIT SEC11C (PTHR10806:SF12)	serine protease(PC00203)	*Homo sapiens*
HUMAN|HGNC=2303|UniProtKB=P16870	CBPE	Carboxypeptidase E;CPE;ortholog	CARBOXYPEPTIDASE E (PTHR11532:SF62)	metalloprotease(PC00153)	*Homo sapiens*
HUMAN|HGNC=4067|UniProtKB=O95166	GBRAP	Gamma-aminobutyric acid receptor-associated protein;GABARAP;ortholog	GABARAP-A-RELATED (PTHR10969:SF20)	non-motor microtubule binding protein(PC00166)	*Homo sapiens*
HUMAN|HGNC=15574|UniProtKB=Q8TDY2	RBCC1	RB1-inducible coiled-coil protein 1;RB1CC1;ortholog	RB1-INDUCIBLE COILED-COIL PROTEIN 1 (PTHR13222:SF1)		*Homo sapiens*
HUMAN|HGNC=16935|UniProtKB=O95352	ATG7	Ubiquitin-like modifier-activating enzyme ATG7;ATG7;ortholog	UBIQUITIN-LIKE MODIFIER-ACTIVATING ENZYME ATG7 (PTHR10953:SF3)	ligase(PC00142);transfer/carrier protein(PC00219)	*Homo sapiens*
HUMAN|HGNC=15789|UniProtKB=P53582	MAP11	Methionine aminopeptidase 1;METAP1;ortholog	METHIONINE AMINOPEPTIDASE 1 (PTHR43330:SF7)	metalloprotease(PC00153);nucleic acid binding(PC00171);transcription factor(PC00218)	*Homo sapiens*
HUMAN|HGNC=1478|UniProtKB=Q9UMQ6	CAN11	Calpain-11;CAPN11;ortholog	CALPAIN-11 (PTHR10183:SF322)	annexin(PC00050);calmodulin(PC00061);cysteine protease(PC00081)	*Homo sapiens*
HUMAN|HGNC=8982|UniProtKB=Q99570	PI3R4	Phosphoinositide 3-kinase regulatory subunit 4;PIK3R4;ortholog	PHOSPHOINOSITIDE 3-KINASE REGULATORY SUBUNIT 4 (PTHR17583:SF0)		*Homo sapiens*
HUMAN|HGNC=2312|UniProtKB=P15169	ACBP	Carboxypeptidase N catalytic chain;CPN1;ortholog	CARBOXYPEPTIDASE N CATALYTIC CHAIN (PTHR11532:SF80)	metalloprotease(PC00153)	*Homo sapiens*

HUMAN|HGNC=7104|UniProtKB=Q99797	MIPEP	Mitochondrial intermediate peptidase;MIPEP;ortholog	MITOCHONDRIAL INTERMEDIATE PEPTIDASE (PTHR11804:SF5)	metalloprotease(PC00153)	*Homo sapiens*
HUMAN|HGNC=12877|UniProtKB=O75844	FACE1	CAAX prenyl protease 1 homolog;ZMPSTE24;ortholog	CAAX PRENYL PROTEASE 1 HOMOLOG (PTHR10120:SF24)	metalloprotease(PC00153)	*Homo sapiens*
HUMAN|HGNC=1034|UniProtKB=Q14457	BECN1	Beclin-1;BECN1;ortholog	BECLIN-1 (PTHR12768:SF6)	protease inhibitor(PC00191)	*Homo sapiens*
HUMAN|HGNC=9535|UniProtKB=P60900	PSA6	Proteasome subunit alpha type-6;PSMA6;ortholog	PROTEASOME SUBUNIT ALPHA TYPE-6 (PTHR11599:SF11)	protease(PC00190)	*Homo sapiens*
HUMAN|HGNC=24185|UniProtKB=P46736	BRCC3	Lys-63-specific deubiquitinase BRCC36;BRCC3;ortholog	LYS-63-SPECIFIC DEUBIQUITINASE BRCC36 (PTHR10410:SF19)	metalloprotease(PC00153);transcription factor(PC00218)	*Homo sapiens*
HUMAN|HGNC=20315|UniProtKB=Q9H0Y0	ATG10	Ubiquitin-like-conjugating enzyme ATG10;ATG10;ortholog	UBIQUITIN-LIKE-CONJUGATING ENZYME ATG10 (PTHR14957:SF1)		*Homo sapiens*
HUMAN|HGNC=14263|UniProtKB=Q9ULC3	RAB23	Ras-related protein Rab-23;RAB23;ortholog	RAS-RELATED PROTEIN RAB-23 (PTHR24073:SF209)		*Homo sapiens*
HUMAN|HGNC=9539|UniProtKB=P49721	PSB2	Proteasome subunit beta type-2;PSMB2;ortholog	PROTEASOME SUBUNIT BETA TYPE-2 (PTHR11599:SF6)	protease(PC00190)	*Homo sapiens*
HUMAN|HGNC=223|UniProtKB=Q9UKP4	ATS7	A disintegrin and metalloproteinase with thrombospondin motifs 7;ADAMTS7;ortholog	A DISINTEGRIN AND METALLOPROTEINASE WITH THROMBOSPONDIN MOTIFS 7 (PTHR13723:SF142)	extracellular matrix glycoprotein(PC00100);metalloprotease(PC00153);serine protease inhibitor(PC00204)	*Homo sapiens*

HUMAN|HGNC=12630|UniProtKB=Q93009	UBP7	Ubiquitin carboxyl-terminal hydrolase 7;USP7;ortholog	UBIQUITIN CARBOXYL-TERMINAL HYDROLASE 7 (PTHR24006:SF753)	cysteine protease(PC00081)	*Homo sapiens*
HUMAN|HGNC=11793|UniProtKB=P52888	THOP1	Thimet oligopeptidase;THOP1;ortholog	THIMET OLIGOPEPTIDASE (PTHR11804:SF50)	metalloprotease(PC00153)	*Homo sapiens*
HUMAN|HGNC=8840|UniProtKB=P12955	PEPD	Xaa-Pro dipeptidase;PEPD;ortholog	XAA-PRO DIPEPTIDASE (PTHR43226:SF1)	metalloprotease(PC00153);nucleic acid binding(PC00171);transcription factor(PC00218)	*Homo sapiens*
HUMAN|HGNC=16448|UniProtKB=Q7L266	ASGL1	Isoaspartyl peptidase/L-asparaginase;ASRGL1;ortholog	ISOASPARTYL PEPTIDASE/L-ASPARAGINASE (PTHR10188:SF30)	protease(PC00190)	*Homo sapiens*
HUMAN|HGNC=20062|UniProtKB=Q9P275	UBP36	Ubiquitin carboxyl-terminal hydrolase 36;USP36;ortholog	UBIQUITIN CARBOXYL-TERMINAL HYDROLASE 36 (PTHR24006:SF653)	cysteine protease(PC00081)	*Homo sapiens*

Hydrolase Activity, ester bonds

HUMAN|HGNC=33911|UniProtKB=Q6P5S7	RNK	Ribonuclease kappa;RNASEK;ortholog	RIBONUCLEASE KAPPA (PTHR31733:SF1)		*Homo sapiens*
HUMAN|HGNC=12379|UniProtKB=Q15631	TSN	Translin;TSN;ortholog	TRANSLIN (PTHR10741:SF2)		*Homo sapiens*
HUMAN|HGNC=23287|UniProtKB=O95571	ETHE1	Persulfide dioxygenase ETHE1, mitochondrial;ETHE1;ortholog	PERSULFIDE DIOXYGENASE ETHE1, MITOCHONDRIAL (PTHR43084:SF4)	hydrolase(PC00121)	*Homo sapiens*
HUMAN|HGNC=24616|UniProtKB=Q8N1G1	REXO1	RNA exonuclease 1 homolog;REXO1;ortholog	RNA EXONUCLEASE 1 HOMOLOG (PTHR12801:SF62)	exoribonuclease(PC00099)	*Homo sapiens*

HUMAN|HGNC=24768|UniProtKB=Q6ZV29	PLPL7	Patatin-like phospholipase domain-containing protein 7;PNPLA7;ortholog	PATATIN-LIKE PHOSPHOLIPASE DOMAIN-CONTAINING PROTEIN 7 (PTHR14226:SF23)	esterase(PC00097)	*Homo sapiens*
HUMAN|HGNC=4056|UniProtKB=P35575	G6PC	Glucose-6-phosphatase;G6PC;ortholog	GLUCOSE-6-PHOSPHATASE (PTHR12591:SF3)		*Homo sapiens*
HUMAN|HGNC=8773|UniProtKB=Q9HCR9	PDE11	Dual 3',5'-cyclic-AMP and -GMP phosphodiesterase 11A;PDE11A;ortholog	DUAL 3',5'-CYCLIC-AMP AND -GMP PHOSPHODIESTERASE 11A (PTHR11347:SF130)		*Homo sapiens*
HUMAN|HGNC=3606|UniProtKB=P09467	FBP1,F16P1	Fructose-1,6-bisphosphatase 1;FBP1;ortholog	FRUCTOSE-1,6-BISPHOSPHATASE 1 (PTHR11556:SF11)	carbohydrate phosphatase(PC00066)	*Homo sapiens*
HUMAN|HGNC=26911|UniProtKB=Q8N2G6	ZCH24	Zinc finger CCHC domain-containing protein 24;ZCCHC24;ortholog	ZINC FINGER CCHC DOMAIN-CONTAINING PROTEIN 24 (PTHR15439:SF4)	nuclease(PC00170)	*Homo sapiens*
HUMAN|HGNC=16268|UniProtKB=Q8IY17	PLPL6	Neuropathy target esterase;PNPLA6;ortholog	NEUROPATHY TARGET ESTERASE (PTHR14226:SF26)	esterase(PC00097)	*Homo sapiens*
HUMAN|HGNC=3465|UniProtKB=P10768	ESTD	S-formylglutathione hydrolase;ESD;ortholog	S-FORMYLGLUTATHIONE HYDROLASE (PTHR10061:SF0)	esterase(PC00097);serine protease(PC00203)	*Homo sapiens*
HUMAN|HGNC=7618|UniProtKB=O14974	MYPT1	Protein phosphatase 1 regulatory subunit 12A;PPP1R12A;ortholog	PROTEIN PHOSPHATASE 1 REGULATORY SUBUNIT 12A (PTHR24179:SF20)		*Homo sapiens*
HUMAN|HGNC=7619|UniProtKB=O60237	MYPT2	Protein phosphatase 1 regulatory subunit 12B;PPP1R12B;ortholog	PROTEIN PHOSPHATASE 1 REGULATORY SUBUNIT 12B (PTHR24179:SF18)		*Homo sapiens*
HUMAN|HGNC=20604|UniProtKB=Q9Y2L1	RRP44	Exosome complex exonuclease RRP44;DIS3;ortholog	EXOSOME COMPLEX EXONUCLEASE RRP44 (PTHR23355:SF35)	endoribonuclease(PC00094);exoribonuclease(PC00099);hydrolase(PC00121)	*Homo sapiens*

HUMAN|HGNC=8779|UniProtKB=Q13370	PDE3B	cGMP-inhibited 3',5'-cyclic phosphodiesterase B;PDE3B;ortholog	PHOSPHODIESTERASE (PTHR11347:SF29)		*Homo sapiens*
HUMAN|HGNC=9989|UniProtKB=Q15493	RGN	Regucalcin;RGN;ortholog	REGUCALCIN (PTHR10907:SF54)	calcium-binding protein(PC00060);esterase(PC00097)	*Homo sapiens*
HUMAN|HGNC=17851|UniProtKB=Q9Y3B8	ORN	Oligoribonuclease, mitochondrial;REXO2;ortholog	OLIGORIBONUCLEASE, MITOCHONDRIAL (PTHR11046:SF0)	exoribonuclease(PC00099);hydrolase(PC00121)	*Homo sapiens*
HUMAN|HGNC=16219|UniProtKB=Q8TEA8	DTD1	D-aminoacyl-tRNA deacylase 1;DTD1;ortholog	D-AMINOACYL-TRNA DEACYLASE 1 (PTHR10472:SF5)	esterase(PC00097)	*Homo sapiens*
HUMAN|HGNC=17689|UniProtKB=Q969H6	POP5	Ribonuclease P/MRP protein subunit POP5;POP5;ortholog	RIBONUCLEASE P/MRP PROTEIN SUBUNIT POP5 (PTHR10993:SF12)		*Homo sapiens*
HUMAN|HGNC=22197|UniProtKB=O43299	AP5Z1	AP-5 complex subunit zeta-1;AP5Z1;ortholog	AP-5 COMPLEX SUBUNIT ZETA-1 (PTHR46488:SF1)		*Homo sapiens*
HUMAN|HGNC=2158|UniProtKB=P09543	CN37	2',3'-cyclic-nucleotide 3'-phosphodiesterase;CNP;ortholog	2',3'-CYCLIC-NUCLEOTIDE 3'-PHOSPHODIESTERASE (PTHR10156:SF0)	phosphodiesterase(PC00185)	*Homo sapiens*
HUMAN|HGNC=29170|UniProtKB=Q9Y2M0	FAN1	Fanconi-associated nuclease 1;FAN1;ortholog	FANCONI-ASSOCIATED NUCLEASE 1 (PTHR15749:SF4)		*Homo sapiens*
HUMAN|HGNC=23292|UniProtKB=P60321	NOS2	Nanos homolog 2;NANOS2;ortholog	NANOS HOMOLOG 2 (PTHR12887:SF14)	nuclease(PC00170)	*Homo sapiens*
HUMAN|HGNC=17687|UniProtKB=Q9Y2P8	RCL1,RPC2	RNA 3'-terminal phosphate cyclase-like protein;RCL1;ortholog	RNA 3'-TERMINAL PHOSPHATE CYCLASE-LIKE PROTEIN (PTHR11096:SF1)	RNA binding protein(PC00031);cyclase(PC00079)	*Homo sapiens*

HUMAN|HGNC=3367|UniProtKB=O75356	ENTP5	Ectonucleoside triphosphate diphosphohydrolase 5;ENTPD5;ortholog	ECTONUCLEOSIDE TRIPHOSPHATE DIPHOSPHOHYDROLASE 5 (PTHR11782:SF35)	lyase(PC00144);nucleotide phosphatase(PC00173)	*Homo sapiens*
HUMAN|HGNC=18884|UniProtKB=Q9NUW8	TYDP1	Tyrosyl-DNA phosphodiesterase 1;TDP1;ortholog	TYROSYL-DNA PHOSPHODIESTERASE 1 (PTHR12415:SF0)	phosphodiesterase(PC00185)	*Homo sapiens*
HUMAN|Ensembl=ENSG00000005189|UniProtKB=Q96IC2	REXO5	RNA exonuclease 5;REXO5;ortholog	RNA EXONUCLEASE 5 (PTHR12801:SF82)	exoribonuclease(PC00099)	*Homo sapiens*
HUMAN|HGNC=20154|UniProtKB=Q8TB40	ABHD4	Protein ABHD4;ABHD4;ortholog	PROTEIN ABHD4 (PTHR42886:SF21)		*Homo sapiens*
HUMAN|HGNC=17059|UniProtKB=Q9NTJ5	SAC1	Phosphatidylinositide phosphatase SAC1;SACM1L;ortholog	PHOSPHATIDYLINOSITIDE PHOSPHATASE SAC1 (PTHR45662:SF2)		*Homo sapiens*
HUMAN|HGNC=8782|UniProtKB=Q08493	PDE4C	cAMP-specific 3',5'-cyclic phosphodiesterase 4C;PDE4C;ortholog	CAMP-SPECIFIC 3',5'-CYCLIC PHOSPHODIESTERASE 4C (PTHR11347:SF135)		*Homo sapiens*
HUMAN|HGNC=20614|UniProtKB=Q8NAT2	TDRD5	Tudor domain-containing protein 5;TDRD5;ortholog	TUDOR DOMAIN-CONTAINING PROTEIN 5 (PTHR22948:SF19)	nuclease(PC00170)	*Homo sapiens*
HUMAN|HGNC=15594|UniProtKB=Q9UK59	DBR1	Lariat debranching enzyme;DBR1;ortholog	LARIAT DEBRANCHING ENZYME (PTHR12849:SF0)	endoribonuclease(PC00094);hydrolase(PC00121)	*Homo sapiens*
HUMAN|HGNC=15506|UniProtKB=Q9BSV6	SEN34	tRNA-splicing endonuclease subunit Sen34;TSEN34;ortholog	TRNA-SPLICING ENDONUCLEASE SUBUNIT SEN34 (PTHR13070:SF0)	endoribonuclease(PC00094)	*Homo sapiens*
HUMAN|HGNC=25671|UniProtKB=Q5TBB1	RNH2B	Ribonuclease H2 subunit B;RNASEH2B;ortholog	RIBONUCLEASE H2 SUBUNIT B (PTHR13383:SF11)		*Homo sapiens*

HUMAN|HGNC=24599|UniProtKB=Q9UHY7	ENOPH	Enolase-phosphatase E1;ENOPH1;ortholog	ENOLASE-PHOSPHATASE E1 (PTHR20371:SF2)	phosphatase(PC00181)	*Homo sapiens*
HUMAN|HGNC=12269|UniProtKB=Q9NSU2	TREX1	Three-prime repair exonuclease 1;TREX1;ortholog	THREE-PRIME REPAIR EXONUCLEASE 1 (PTHR13058:SF19)		*Homo sapiens*
HUMAN|HGNC=9059|UniProtKB=Q15147	PLCB4	1-phosphatidylinositol 4,5-bisphosphate phosphodiesterase beta-4;PLCB4;ortholog	1-PHOSPHATIDYLINOSITOL 4,5-BISPHOSPHATE PHOSPHODIESTERASE BETA-4 (PTHR10336:SF106)	calcium-binding protein(PC00060);guanyl-nucleotide exchange factor(PC00113);phospholipase(PC00186);signaling molecule(PC00207)	*Homo sapiens*
HUMAN|HGNC=8792|UniProtKB=Q9NP56	PDE7B	cAMP-specific 3',5'-cyclic phosphodiesterase 7B;PDE7B;ortholog	CAMP-SPECIFIC 3',5'-CYCLIC PHOSPHODIESTERASE 7B (PTHR11347:SF72)		*Homo sapiens*
HUMAN|HGNC=20097|UniProtKB=Q96LQ0	PPR36	Protein phosphatase 1 regulatory subunit 36;PPP1R36;ortholog	PROTEIN PHOSPHATASE 1 REGULATORY SUBUNIT 36 (PTHR21055:SF3)		*Homo sapiens*
HUMAN|HGNC=9062|UniProtKB=Q9BRC7	PLCD4	1-phosphatidylinositol 4,5-bisphosphate phosphodiesterase delta-4;PLCD4;ortholog	1-PHOSPHATIDYLINOSITOL 4,5-BISPHOSPHATE PHOSPHODIESTERASE DELTA-4 (PTHR10336:SF31)	guanyl-nucleotide exchange factor(PC00113);signaling molecule(PC00207)	*Homo sapiens*
HUMAN|HGNC=18344|UniProtKB=Q9BX68	HINT2	Histidine triad nucleotide-binding protein 2, mitochondrial;HINT2;ortholog	HISTIDINE TRIAD NUCLEOTIDE-BINDING PROTEIN 2, MITOCHONDRIAL (PTHR23089:SF18)	nucleotide phosphatase(PC00173)	*Homo sapiens*
HUMAN|HGNC=27696|UniProtKB=Q2TAA2	IAH1	Isoamyl acetate-hydrolyzing esterase 1 homolog;IAH1;ortholog	ISOAMYL ACETATE-HYDROLYZING ESTERASE 1 HOMOLOG (PTHR14209:SF9)		*Homo sapiens*
HUMAN|HGNC=12380|UniProtKB=Q99598	TSNAX	Translin-associated protein X;TSNAX;ortholog	TRANSLIN-ASSOCIATED PROTEIN X (PTHR10741:SF7)		*Homo sapiens*

HUMAN|HGNC=14099|UniProtKB=Q9ULM6	CCR4A	CCR4-NOT transcription complex subunit 6;CNOT6;ortholog	CCR4-NOT TRANSCRIPTION COMPLEX SUBUNIT 6 (PTHR12121:SF33)	exoribonuclease(PC00099)	*Homo sapiens*
HUMAN|HGNC=14101|UniProtKB=Q9UIV1	CNOT7,CAF1	CCR4-NOT transcription complex subunit 7;CNOT7;ortholog	CCR4-NOT TRANSCRIPTION COMPLEX SUBUNIT 7 (PTHR10797:SF2)	transcription factor(PC00218)	*Homo sapiens*
HUMAN|HGNC=18466|UniProtKB=O60930	RNH1	Ribonuclease H1;RNASEH1;ortholog	RIBONUCLEASE H1 (PTHR10642:SF0)		*Homo sapiens*
HUMAN|HGNC=9040|UniProtKB=Q13093	PAFA	Platelet-activating factor acetylhydrolase;PLA2G7;ortholog	PLATELET-ACTIVATING FACTOR ACETYLHYDROLASE (PTHR10272:SF12)	esterase(PC00097)	*Homo sapiens*
HUMAN|HGNC=28417|UniProtKB=Q9BTE6	AASD1	Alanyl-tRNA editing protein Aarsd1;AARSD1;ortholog	ALANYL-TRNA EDITING PROTEIN AARSD1 (PTHR43462:SF1)	RNA binding protein(PC00031)	*Homo sapiens*
HUMAN|HGNC=21396|UniProtKB=Q8WTS1	ABHD5	1-acylglycerol-3-phosphate O-acyltransferase ABHD5;ABHD5;ortholog	1-ACYLGLYCEROL-3-PHOSPHATE O-ACYLTRANSFERASE ABHD5 (PTHR42886:SF34)		*Homo sapiens*
HUMAN|HGNC=4390|UniProtKB=P50148	GNAQ	Guanine nucleotide-binding protein G(q) subunit alpha;GNAQ;ortholog	GUANINE NUCLEOTIDE-BINDING PROTEIN G(Q) SUBUNIT ALPHA (PTHR10218:SF318)	heterotrimeric G-protein(PC00117)	*Homo sapiens*
HUMAN|HGNC=30831|UniProtKB=Q8NHU6	TDRD7	Tudor domain-containing protein 7;TDRD7;ortholog	TUDOR DOMAIN-CONTAINING PROTEIN 7 (PTHR22948:SF14)	nuclease(PC00170)	*Homo sapiens*
HUMAN|HGNC=4422|UniProtKB=P15586	GNS	N-acetylglucosamine-6-sulfatase;GNS;ortholog	N-ACETYLGLUCOSAMINE-6-SULFATASE (PTHR43108:SF5)	hydrolase(PC00121)	*Homo sapiens*
HUMAN|HGNC=28698|UniProtKB=Q8TF46	DI3L1	DIS3-like exonuclease 1;DIS3L;ortholog	DIS3-LIKE EXONUCLEASE 1 (PTHR23355:SF30)	endoribonuclease(PC00094);exoribonuclease(PC00099);hydrolase(PC00121)	*Homo sapiens*

HUMAN|HGNC=12791|UniProtKB=Q14191	WRN	Werner syndrome ATP-dependent helicase;WRN;ortholog	WERNER SYNDROME ATP-DEPENDENT HELICASE (PTHR13710:SF120)	DNA helicase(PC00011)	*Homo sapiens*
HUMAN|HGNC=18483|UniProtKB=Q8WWY8	LIPH	Lipase member H;LIPH;ortholog	LIPASE MEMBER H (PTHR11610:SF12)	esterase(PC00097);lipase(PC00143);storage protein(PC00210)	*Homo sapiens*
HUMAN|HGNC=2326|UniProtKB=Q9UKF6	CPSF3	Cleavage and polyadenylation specificity factor subunit 3;CPSF3;ortholog	CLEAVAGE AND POLYADENYLATION SPECIFICITY FACTOR SUBUNIT 3 (PTHR11203:SF11)	endoribonuclease(PC00094);mRNA polyadenylation factor(PC00146)	*Homo sapiens*
HUMAN|HGNC=24860|UniProtKB=Q5MY95	ENTP8	Ectonucleoside triphosphate diphosphohydrolase 8;ENTPD8;ortholog	ECTONUCLEOSIDE TRIPHOSPHATE DIPHOSPHOHYDROLASE 8 (PTHR11782:SF31)	lyase(PC00144);nucleotide phosphatase(PC00173)	*Homo sapiens*
HUMAN|HGNC=9889|UniProtKB=Q7Z6E9	RBBP6	E3 ubiquitin-protein ligase RBBP6;RBBP6;ortholog	E3 UBIQUITIN-PROTEIN LIGASE RBBP6 (PTHR15439:SF0)	nuclease(PC00170)	*Homo sapiens*
HUMAN|HGNC=19909|UniProtKB=Q8N5L8	RP25L	Ribonuclease P protein subunit p25-like protein;RPP25L;ortholog	RIBONUCLEASE P PROTEIN SUBUNIT P25-LIKE PROTEIN (PTHR13516:SF8)	nuclease(PC00170)	*Homo sapiens*
HUMAN|HGNC=11407|UniProtKB=Q9BXU1	STK31	Serine/threonine-protein kinase 31;STK31;ortholog	SERINE/THREONINE-PROTEIN KINASE 31 (PTHR12302:SF3)	nucleic acid binding(PC00171);transcription cofactor(PC00217)	*Homo sapiens*
HUMAN|HGNC=18042|UniProtKB=Q96LI5	CNO6L	CCR4-NOT transcription complex subunit 6-like;CNOT6L;ortholog	CCR4-NOT TRANSCRIPTION COMPLEX SUBUNIT 6-LIKE (PTHR12121:SF35)	exoribonuclease(PC00099)	*Homo sapiens*
HUMAN|HGNC=19949|UniProtKB=O75817	POP7	Ribonuclease P protein subunit p20;POP7;ortholog	RIBONUCLEASE P PROTEIN SUBUNIT P20 (PTHR15314:SF1)	endoribonuclease(PC00094);hydrolase(PC00121)	*Homo sapiens*
HUMAN|HGNC=8021|UniProtKB=P21589	5NTD	5'-nucleotidase;NT5E;ortholog	5'-NUCLEOTIDASE (PTHR11575:SF25)	phosphodiesterase(PC00185)	*Homo sapiens*

HUMAN|HGNC=13236|UniProtKB=O94763	RMP	Unconventional prefoldin RPB5 interactor 1;URI1;ortholog	UNCONVENTIONAL PREFOLDIN RPB5 INTERACTOR 1 (PTHR15111:SF0)	transcription factor(PC00218)	*Homo sapiens*
HUMAN|HGNC=30802|UniProtKB=Q96AD5	PLPL2	Patatin-like phospholipase domain-containing protein 2;PNPLA2;ortholog	PATATIN-LIKE PHOSPHOLIPASE DOMAIN-CONTAINING PROTEIN 2 (PTHR12406:SF29)	acyltransferase(PC00042);phospholipase(PC00186)	*Homo sapiens*
HUMAN|HGNC=5293|UniProtKB=P28223	5HT2A	5-hydroxytryptamine receptor 2A;HTR2A;ortholog	5-HYDROXYTRYPTAMINE RECEPTOR 2A (PTHR24247:SF30)	G-protein coupled receptor(PC00021)	*Homo sapiens*
HUMAN|HGNC=16650|UniProtKB=Q9H9J2	RM44	39S ribosomal protein L44, mitochondrial;MRPL44;ortholog	39S RIBOSOMAL PROTEIN L44, MITOCHONDRIAL (PTHR11207:SF5)	endoribonuclease(PC00094)	*Homo sapiens*
HUMAN|HGNC=23845|UniProtKB=Q8IY92	SLX4	Structure-specific endonuclease subunit SLX4;SLX4;ortholog	STRUCTURE-SPECIFIC ENDONUCLEASE SUBUNIT SLX4 (PTHR21541:SF3)		*Homo sapiens*
HUMAN|HGNC=2484|UniProtKB=P33240	CSTF2	Cleavage stimulation factor subunit 2;CSTF2;ortholog	CLEAVAGE STIMULATION FACTOR SUBUNIT 2 (PTHR45735:SF6)	mRNA splicing factor(PC00148)	*Homo sapiens*
HUMAN|HGNC=10293|UniProtKB=Q96AT9	RPE	Ribulose-phosphate 3-epimerase;RPE;ortholog	RIBULOSE-PHOSPHATE 3-EPIMERASE-RELATED (PTHR11749:SF9)		*Homo sapiens*
HUMAN|HGNC=17175|UniProtKB=Q9P212	PLCE1	1-phosphatidylinositol 4,5-bisphosphate phosphodiesterase epsilon-1;PLCE1;ortholog	1-PHOSPHATIDYLINOSITOL 4,5-BISPHOSPHATE PHOSPHODIESTERASE EPSILON-1 (PTHR10336:SF6)	calcium-binding protein(PC00060);guanyl-nucleotide exchange factor(PC00113);phospholipase(PC00186);signaling molecule(PC00207)	*Homo sapiens*
HUMAN|HGNC=21300|UniProtKB=Q9H633	RPP21	Ribonuclease P protein subunit p21;RPP21;ortholog	RIBONUCLEASE P PROTEIN SUBUNIT P21 (PTHR14742:SF0)	hydrolase(PC00121);nuclease(PC00170)	*Homo sapiens*

HUMAN|HGNC=17641|UniProtKB=Q9H816	DCR1B	5' exonuclease Apollo;DCLRE1B;ortholog	5' EXONUCLEASE APOLLO (PTHR23240:SF27)		*Homo sapiens*
HUMAN|HGNC=3650|UniProtKB=P39748	FEN1	Flap endonuclease 1;FEN1;ortholog	FLAP ENDONUCLEASE 1 (PTHR11081:SF54)	damaged DNA-binding protein(PC00086);endodeoxyribonuclease(PC00093);exodeoxyribonuclease(PC00098);hydrolase(PC00121)	*Homo sapiens*
HUMAN|HGNC=277|UniProtKB=P35348	ADA1A	Alpha-1A adrenergic receptor;ADRA1A;ortholog	ALPHA-1A ADRENERGIC RECEPTOR (PTHR24248:SF16)	G-protein coupled receptor(PC00021)	*Homo sapiens*
HUMAN|HGNC=21339|UniProtKB=O60522	TDRD6	Tudor domain-containing protein 6;TDRD6;ortholog	TUDOR DOMAIN-CONTAINING PROTEIN 6 (PTHR22948:SF15)	nuclease(PC00170)	*Homo sapiens*
HUMAN|HGNC=26019|UniProtKB=Q9NX62	IMPA3	Inositol monophosphatase 3;IMPAD1;ortholog	INOSITOL MONOPHOSPHATASE 3 (PTHR43028:SF6)	phosphatase(PC00181)	*Homo sapiens*
HUMAN|HGNC=21416|UniProtKB=Q92485	ASM3B	Acid sphingomyelinase-like phosphodiesterase 3b;SMPDL3B;ortholog	ACID SPHINGOMYELINASE-LIKE PHOSPHODIESTERASE 3B (PTHR10340:SF25)	phosphodiesterase(PC00185)	*Homo sapiens*
HUMAN|HGNC=17660|UniProtKB=Q6PJP8	SNM1,DCR1A	DNA cross-link repair 1A protein;DCLRE1A;ortholog	DNA CROSS-LINK REPAIR 1A PROTEIN (PTHR23240:SF6)		*Homo sapiens*
HUMAN|HGNC=17889|UniProtKB=Q9UBZ4	APEX2	DNA-(apurinic or apyrimidinic site) lyase 2;APEX2;ortholog	DNA-(APURINIC OR APYRIMIDINIC SITE) LYASE 2 (PTHR22748:SF4)		*Homo sapiens*
HUMAN|HGNC=21185|UniProtKB=Q8NA58	PNDC1	Poly(A)-specific ribonuclease PNLDC1;PNLDC1;ortholog	POLY(A)-SPECIFIC RIBONUCLEASE PNLDC1 (PTHR15092:SF22)	mRNA polyadenylation factor(PC00146)	*Homo sapiens*
HUMAN|HGNC=25044|UniProtKB=Q587J7	TDR12	Putative ATP-dependent RNA helicase TDRD12;TDRD12;ortholog	ATP-DEPENDENT RNA HELICASE TDRD12-RELATED (PTHR22948:SF31)	nuclease(PC00170);signaling molecule(PC00207)	*Homo sapiens*

HUMAN|HGNC=24763|UniProtKB=Q6ZVT6	CC067	Uncharacterized protein C3orf67;C3orf67;ortholog	ZGC:162324 (PTHR12458:SF7)	nuclease(PC00170);transcription factor(PC00218)	*Homo sapiens*
HUMAN|HGNC=17098|UniProtKB=Q9UPY3	DICER	Endoribonuclease Dicer;DICER1;ortholog	ENDORIBONUCLEASE DICER (PTHR14950:SF37)	endodeoxyribonuclease(PC00093)	*Homo sapiens*
HUMAN|HGNC=12836|UniProtKB=Q9H0D6	XRN2	5'-3' exoribonuclease 2;XRN2;ortholog	5'-3' EXORIBONUCLEASE 2 (PTHR12341:SF59)	exoribonuclease(PC00099)	*Homo sapiens*
HUMAN|HGNC=20074|UniProtKB=Q504Q3	PAN2	PAN2-PAN3 deadenylation complex catalytic subunit PAN2;PAN2;ortholog	PAN2-PAN3 DEADENYLATION COMPLEX CATALYTIC SUBUNIT PAN2 (PTHR15728:SF0)	cysteine protease(PC00081);esterase(PC00097);exoribonuclease(PC00099)	*Homo sapiens*
HUMAN|HGNC=24660|UniProtKB=Q8IX06	GOR	Putative exonuclease GOR;REXO1L1P;ortholog	EXONUCLEASE GOR-RELATED (PTHR12801:SF22)	exoribonuclease(PC00099)	*Homo sapiens*
HUMAN|HGNC=25026|UniProtKB=Q8NEB5	PLPP5	Phospholipid phosphatase 5;PLPP5;ortholog	PHOSPHOLIPID PHOSPHATASE 5 (PTHR10165:SF87)	phosphatase(PC00181);pyrophosphatase(PC00196)	*Homo sapiens*
HUMAN|HGNC=10297|UniProtKB=P49247	RPIA	Ribose-5-phosphate isomerase;RPIA;ortholog	RIBOSE-5-PHOSPHATE ISOMERASE (PTHR11934:SF0)		*Homo sapiens*
HUMAN|HGNC=9156|UniProtKB=P54315	LIPR1	Inactive pancreatic lipase-related protein 1;PNLIPRP1;ortholog	INACTIVE PANCREATIC LIPASE-RELATED PROTEIN 1 (PTHR11610:SF147)	esterase(PC00097);lipase(PC00143);storage protein(PC00210)	*Homo sapiens*
HUMAN|HGNC=6619|UniProtKB=P11150	LIPC	Hepatic triacylglycerol lipase;LIPC;ortholog	HEPATIC TRIACYLGLYCEROL LIPASE (PTHR11610:SF2)	esterase(PC00097);lipase(PC00143);storage protein(PC00210)	*Homo sapiens*
HUMAN|HGNC=6737|UniProtKB=O75608	LYPA1	Acyl-protein thioesterase 1;LYPLA1;ortholog	ACYL-PROTEIN THIOESTERASE 1 (PTHR10655:SF22)		*Homo sapiens*

HUMAN|HGNC=23044|UniProtKB=Q8WY41	NOS1	Nanos homolog 1;NANOS1;ortholog	NANOS HOMOLOG 1 (PTHR12887:SF6)	nuclease(PC00170)	*Homo sapiens*
HUMAN|HGNC=17163|UniProtKB=Q8NCC3	PAG15	Group XV phospholipase A2;PLA2G15;ortholog	GROUP XV PHOSPHOLIPASE A2 (PTHR11440:SF47)	acyltransferase(PC00042);phospholipase(PC00186)	*Homo sapiens*
HUMAN|HGNC=3066|UniProtKB=O75319	DUS11	RNA/RNP complex-1-interacting phosphatase;DUSP11;ortholog	RNA/RNP COMPLEX-1-INTERACTING PHOSPHATASE (PTHR10367:SF9)	nucleotidyltransferase(PC00174);phosphatase(PC00181)	*Homo sapiens*
HUMAN|HGNC=25364|UniProtKB=Q9H0R4	HDHD2	Haloacid dehalogenase-like hydrolase domain-containing protein 2;HDHD2;ortholog	HALOACID DEHALOGENASE-LIKE HYDROLASE DOMAIN-CONTAINING PROTEIN 2 (PTHR19288:SF43)	Homo sapiens	
HUMAN|HGNC=24220|UniProtKB=Q6P1N9	TATD1	Putative deoxyribonuclease TATDN1;TATDN1;ortholog	DEOXYRIBONUCLEASE TATDN1-RELATED (PTHR10060:SF15)		*Homo sapiens*
HUMAN|HGNC=17086|UniProtKB=Q9H0L4	CSTFT	Cleavage stimulation factor subunit 2 tau variant;CSTF2T;ortholog	CLEAVAGE STIMULATION FACTOR SUBUNIT 2 TAU VARIANT (PTHR45735:SF3)	mRNA splicing factor(PC00148)	*Homo sapiens*
HUMAN|HGNC=9827|UniProtKB=Q99638	RAD9A	Cell cycle checkpoint control protein RAD9A;RAD9A;ortholog	CELL CYCLE CHECKPOINT CONTROL PROTEIN RAD9A (PTHR15237:SF1)	kinase activator(PC00138)	*Homo sapiens*
HUMAN|HGNC=11120|UniProtKB=P17405	ASM	Sphingomyelin phosphodiesterase;SMPD1;ortholog	SPHINGOMYELIN PHOSPHODIESTERASE (PTHR10340:SF34)	phosphodiesterase(PC00185)	*Homo sapiens*
HUMAN|HGNC=17018|UniProtKB=Q9Y6Y8	S23IP	SEC23-interacting protein;SEC23IP;ortholog	SEC23-INTERACTING PROTEIN (PTHR23509:SF4)	membrane traffic protein(PC00150);phospholipase(PC00186)	*Homo sapiens*

HUMAN|HGNC=24644|UniProtKB=Q9UPR3	SMG5	Protein SMG5;SMG5;ortholog	PROTEIN SMG5 (PTHR15696:SF7)		*Homo sapiens*
HUMAN|HGNC=33882|UniProtKB=Q9BT40	PPS,INP5K	Inositol polyphosphate 5-phosphatase K;INPP5K;ortholog	INOSITOL POLYPHOSPHATE 5-PHOSPHATASE K (PTHR11200:SF117)	phosphatase(PC00181)	*Homo sapiens*
HUMAN|HGNC=14254|UniProtKB=Q9UK39	NOC	Nocturnin;NOCT;ortholog	NOCTURNIN (PTHR12121:SF45)	exoribonuclease(PC00099)	*Homo sapiens*
HUMAN|HGNC=28142|UniProtKB=Q9BT23	LIMD2	LIM domain-containing protein 2;LIMD2;ortholog	LIM DOMAIN-CONTAINING PROTEIN 2 (PTHR24206:SF58)	actin family cytoskeletal protein(PC00041);nuclease(PC00170);transcription factor(PC00218)	*Homo sapiens*
HUMAN|HGNC=587|UniProtKB=P27695	APEX1	DNA-(apurinic or apyrimidinic site) lyase;APEX1;ortholog	DNA-(APURINIC OR APYRIMIDINIC SITE) LYASE (PTHR22748:SF6)		*Homo sapiens*
HUMAN|HGNC=9207|UniProtKB=Q9UFF9	CAF1B	CCR4-NOT transcription complex subunit 8;CNOT8;ortholog	CCR4-NOT TRANSCRIPTION COMPLEX SUBUNIT 8 (PTHR10797:SF1)	transcription factor(PC00218)	*Homo sapiens*
HUMAN|HGNC=9155|UniProtKB=P16233	LIPP	Pancreatic triacylglycerol lipase;PNLIP;ortholog	PANCREATIC TRIACYLGLYCEROL LIPASE (PTHR11610:SF115)	esterase(PC00097);lipase(PC00143);storage protein(PC00210)	*Homo sapiens*
HUMAN|HGNC=28424|UniProtKB=Q9BQ61	TRIR	Telomerase RNA component interacting RNase;TRIR;ortholog	TELOMERASE RNA COMPONENT INTERACTING RNASE (PTHR34753:SF1)		*Homo sapiens*
HUMAN|HGNC=8778|UniProtKB=Q14432	PDE3A	cGMP-inhibited 3',5'-cyclic phosphodiesterase A;PDE3A;ortholog	PHOSPHODIESTERASE (PTHR11347:SF104)		*Homo sapiens*
HUMAN|HGNC=15984|UniProtKB=Q7Z2E3	APTX	Aprataxin;APTX;ortholog	APRATAXIN (PTHR12486:SF4)	damaged DNA-binding protein(PC00086)	*Homo sapiens*

HUMAN|HGNC=9177|UniProtKB=Q07864	DPOE1	DNA polymerase epsilon catalytic subunit A;POLE;ortholog	DNA POLYMERASE EPSILON CATALYTIC SUBUNIT A (PTHR10670:SF0)		*Homo sapiens*
HUMAN|HGNC=20999|UniProtKB=Q9NPJ3	ACO13	Acyl-coenzyme A thioesterase 13;ACOT13;ortholog	ACYL-COENZYME A THIOESTERASE 13 (PTHR21660:SF1)		*Homo sapiens*
HUMAN|HGNC=11712|UniProtKB=Q9BXT4	TDRD1	Tudor domain-containing protein 1;TDRD1;ortholog	TUDOR DOMAIN-CONTAINING PROTEIN 1 (PTHR22948:SF4)	nuclease(PC00170)	*Homo sapiens*
HUMAN|HGNC=9891|UniProtKB=Q99708	CTIP,SAE2	DNA endonuclease RBBP8;RBBP8;ortholog	DNA ENDONUCLEASE RBBP8 (PTHR15107:SF4)	transcription cofactor(PC00217)	*Homo sapiens*
HUMAN|HGNC=12270|UniProtKB=Q9BQ50	TREX2	Three prime repair exonuclease 2;TREX2;ortholog	THREE PRIME REPAIR EXONUCLEASE 2 (PTHR13058:SF24)		*Homo sapiens*
HUMAN|HGNC=28422|UniProtKB=Q8NCE0	SEN2	tRNA-splicing endonuclease subunit Sen2;TSEN2;ortholog	TRNA-SPLICING ENDONUCLEASE SUBUNIT SEN2 (PTHR21227:SF0)	endodeoxyribonuclease(PC00093);hydrolase(PC00121)	*Homo sapiens*
HUMAN|HGNC=23994|UniProtKB=Q8IV48	ERI1	3'-5' exoribonuclease 1;ERI1;ortholog	3'-5' EXORIBONUCLEASE 1 (PTHR23044:SF27)	esterase(PC00097);exoribonuclease(PC00099)	*Homo sapiens*
HUMAN|HGNC=16205|UniProtKB=Q9BQP7	MGME1	Mitochondrial genome maintenance exonuclease 1;MGME1;ortholog	MITOCHONDRIAL GENOME MAINTENANCE EXONUCLEASE 1 (PTHR31340:SF3)		*Homo sapiens*
HUMAN|HGNC=8909|UniProtKB=A6NDG6	PGP	Glycerol-3-phosphate phosphatase;PGP;ortholog	GLYCEROL-3-PHOSPHATE PHOSPHATASE (PTHR19288:SF69)		*Homo sapiens*
HUMAN|HGNC=9229|UniProtKB=O14495	PLPP3	Phospholipid phosphatase 3;PLPP3;ortholog	PHOSPHOLIPID PHOSPHATASE 3 (PTHR10165:SF79)	phosphatase(PC00181);pyrophosphatase(PC00196)	*Homo sapiens*

HUMAN|HGNC=9157|UniProtKB=P54317	LIPR2	Pancreatic lipase-related protein 2;PNLIPRP2;ortholog	PANCREATIC LIPASE-RELATED PROTEIN 2 (PTHR11610:SF85)	esterase(PC00097);lipase(PC00143);storage protein(PC00210)	*Homo sapiens*
HUMAN|HGNC=20217|UniProtKB=Q9NVH0	EXD2	Exonuclease 3'-5' domain-containing protein 2;EXD2;ortholog	EXONUCLEASE 3'-5' DOMAIN-CONTAINING PROTEIN 2 (PTHR13620:SF0)	nuclease(PC00170)	*Homo sapiens*
HUMAN|HGNC=23719|UniProtKB=Q96FM1	PGAP3	Post-GPI attachment to proteins factor 3;PGAP3;ortholog	POST-GPI ATTACHMENT TO PROTEINS FACTOR 3 (PTHR13148:SF0)		*Homo sapiens*
HUMAN|HGNC=4912|UniProtKB=P49773	HINT1,HINT	Histidine triad nucleotide-binding protein 1;HINT1;ortholog	HISTIDINE TRIAD NUCLEOTIDE-BINDING PROTEIN 1 (PTHR23089:SF44)	nucleotide phosphatase(PC00173)	*Homo sapiens*
HUMAN|HGNC=25499|UniProtKB=Q9NW75	GPTC2	G patch domain-containing protein 2;GPATCH2;ortholog	G PATCH DOMAIN-CONTAINING PROTEIN 2 (PTHR14195:SF4)	nucleic acid binding(PC00171)	*Homo sapiens*
HUMAN|HGNC=9179|UniProtKB=P54098	DPOG1	DNA polymerase subunit gamma-1;POLG;ortholog	DNA POLYMERASE SUBUNIT GAMMA-1 (PTHR10267:SF0)	DNA-directed DNA polymerase(PC00018);exodeoxyribonuclease(PC00098);nucleotidyltransferase(PC00174)	*Homo sapiens*
HUMAN|HGNC=30081|UniProtKB=O95707	RPP29	Ribonuclease P protein subunit p29;POP4;ortholog	RIBONUCLEASE P PROTEIN SUBUNIT P29 (PTHR13348:SF0)		*Homo sapiens*
HUMAN|HGNC=26023|UniProtKB=Q8N9H8	MUT7	Exonuclease mut-7 homolog;EXD3;ortholog	EXONUCLEASE MUT-7 HOMOLOG (PTHR13620:SF42)	nuclease(PC00170)	*Homo sapiens*
HUMAN|HGNC=9035|UniProtKB=P47712	PA24A	Cytosolic phospholipase A2;PLA2G4A;ortholog	CYTOSOLIC PHOSPHOLIPASE A2 (PTHR10728:SF13)	phospholipase(PC00186)	*Homo sapiens*
HUMAN|HGNC=19124|UniProtKB=Q6UN15	FIP1	Pre-mRNA 3'-end-processing factor FIP1;FIP1L1;ortholog	PRE-MRNA 3'-END-PROCESSING FACTOR FIP1 (PTHR13484:SF5)		*Homo sapiens*

HUMAN|HGNC=29523|UniProtKB=Q9Y6A4	CFA20	Cilia- and flagella-associated protein 20;CFAP20;ortholog	CILIA- AND FLAGELLA-ASSOCIATED PROTEIN 20 (PTHR12458:SF8)	nuclease(PC00170);transcription factor(PC00218)	*Homo sapiens*
HUMAN|HGNC=15520|UniProtKB=P43657	LPAR6	Lysophosphatidic acid receptor 6;LPAR6;ortholog	LYSOPHOSPHATIDIC ACID RECEPTOR 6 (PTHR24232:SF3)		*Homo sapiens*
HUMAN|HGNC=19958|UniProtKB=O15091	MRPP3	Mitochondrial ribonuclease P catalytic subunit;KIAA0391;ortholog	MITOCHONDRIAL RIBONUCLEASE P CATALYTIC SUBUNIT (PTHR13547:SF1)		*Homo sapiens*
HUMAN|HGNC=367|UniProtKB=Q92667	AKAP1	A-kinase anchor protein 1, mitochondrial;AKAP1;ortholog	A-KINASE ANCHOR PROTEIN 1, MITOCHONDRIAL (PTHR22948:SF48)	nuclease(PC00170);signaling molecule(PC00207)	*Homo sapiens*
HUMAN|HGNC=8903|UniProtKB=O95336	6PGL	6-phosphogluconolactonase;PGLS;ortholog	6-PHOSPHOGLUCONOLACTONASE (PTHR11054:SF0)	hydrolase(PC00121)	*Homo sapiens*
HUMAN|HGNC=9577|UniProtKB=P78330	SERB	Phosphoserine phosphatase;PSPH;ortholog	PHOSPHOSERINE PHOSPHATASE (PTHR43344:SF2)	phosphatase(PC00181)	*Homo sapiens*
HUMAN|HGNC=9228|UniProtKB=O14494	LPP1	Phospholipid phosphatase 1;PLPP1;ortholog	PHOSPHOLIPID PHOSPHATASE 1 (PTHR10165:SF26)	phosphatase(PC00181);pyrophosphatase(PC00196)	*Homo sapiens*
HUMAN|HGNC=14451|UniProtKB=Q9BQK8	LPIN3	Phosphatidate phosphatase LPIN3;LPIN3;ortholog	PHOSPHATIDATE PHOSPHATASE LPIN3 (PTHR12181:SF60)		*Homo sapiens*
HUMAN|HGNC=108|UniProtKB=P22303	ACES	Acetylcholinesterase;ACHE;ortholog	ACETYLCHOLINESTERASE (PTHR11559:SF393)	lipase(PC00143);serine protease(PC00203)	*Homo sapiens*
HUMAN|HGNC=23409|UniProtKB=Q6UWR7	ENPP6	Ectonucleotide pyrophosphatase/phosphodiesterase family member 6;ENPP6;ortholog	ECTONUCLEOTIDE PYROPHOSPHATASE/PHOSPHODIESTERASE FAMILY MEMBER 6 (PTHR10151:SF66)	nucleotide phosphatase(PC00173);pyrophosphatase(PC00196)	*Homo sapiens*

HUMAN|HGNC=4908|UniProtKB=Q6NVY1	HIBCH	3-hydroxyisobutyryl-CoA hydrolase, mitochondrial;HIBCH;ortholog	3-HYDROXYISOBUTYRYL-COA HYDROLASE, MITOCHONDRIAL (PTHR43176:SF13)	acetyltransferase(PC00038);acyltransferase(PC00042);dehydrogenase(PC00092);epimerase/racemase(PC00096);hydratase(PC00120);ligase(PC00142)	*Homo sapiens*
HUMAN|HGNC=6071|UniProtKB=P49441	IPP	Inositol polyphosphate 1-phosphatase;INPP1;ortholog	INOSITOL POLYPHOSPHATE 1-PHOSPHATASE (PTHR43028:SF3)	phosphatase(PC00181)	*Homo sapiens*
HUMAN|HGNC=3363|UniProtKB=P49961	ENTP1	Ectonucleoside triphosphate diphosphohydrolase 1;ENTPD1;ortholog	ECTONUCLEOSIDE TRIPHOSPHATE DIPHOSPHOHYDROLASE 1 (PTHR11782:SF32)	lyase(PC00144);nucleotide phosphatase(PC00173)	*Homo sapiens*
HUMAN|HGNC=17904|UniProtKB=Q9NRR4	RNC	Ribonuclease 3;DROSHA;ortholog	RIBONUCLEASE 3 (PTHR11207:SF0)	endoribonuclease(PC00094)	*Homo sapiens*
HUMAN|HGNC=25792|UniProtKB=Q9BQ65	USB1	U6 snRNA phosphodiesterase;USB1;ortholog	U6 SNRNA PHOSPHODIESTERASE (PTHR13522:SF3)		*Homo sapiens*
HUMAN|HGNC=8781|UniProtKB=Q07343	PDE4B	cAMP-specific 3',5'-cyclic phosphodiesterase 4B;PDE4B;ortholog	CAMP-SPECIFIC 3',5'-CYCLIC PHOSPHODIESTERASE 4B (PTHR11347:SF108)		*Homo sapiens*
HUMAN|HGNC=15925|UniProtKB=Q9Y3Z3	SAMH1	Deoxynucleoside triphosphate triphosphohydrolase SAMHD1;SAMHD1;ortholog	DEOXYNUCLEOSIDE TRIPHOSPHATE TRIPHOSPHOHYDROLASE SAMHD1 (PTHR11373:SF4)		*Homo sapiens*
HUMAN|HGNC=18718|UniProtKB=Q8WU67	ABHD3	Phospholipase ABHD3;ABHD3;ortholog	PHOSPHOLIPASE ABHD3 (PTHR10794:SF50)	serine protease(PC00203)	*Homo sapiens*
HUMAN|HGNC=1864|UniProtKB=O00748	CES2	Cocaine esterase;CES2;ortholog	COCAINE ESTERASE (PTHR11559:SF394)	lipase(PC00143);serine protease(PC00203)	*Homo sapiens*

HUMAN|HGNC=6050|UniProtKB=P29218	IMPA1	Inositol monophosphatase 1;IMPA1;ortholog	INOSITOL MONOPHOSPHATASE 1 (PTHR20854:SF26)	phosphatase(PC00181)	*Homo sapiens*
HUMAN|HGNC=25386|UniProtKB=Q6L8Q7	PDE12	2',5'-phosphodiesterase 12;PDE12;ortholog	2',5'-PHOSPHODIESTERASE 12 (PTHR12121:SF37)	exoribonuclease(PC00099)	*Homo sapiens*
HUMAN|HGNC=8022|UniProtKB=P49902	5NTC	Cytosolic purine 5'-nucleotidase;NT5C2;ortholog	CYTOSOLIC PURINE 5'-NUCLEOTIDASE (PTHR12103:SF17)	nucleotide phosphatase(PC00173)	*Homo sapiens*
HUMAN|HGNC=29991|UniProtKB=Q58A45	PAN3	PAN2-PAN3 deadenylation complex subunit PAN3;PAN3;ortholog	PAN2-PAN3 DEADENYLATION COMPLEX SUBUNIT PAN3 (PTHR12272:SF11)		*Homo sapiens*
HUMAN|HGNC=29540|UniProtKB=Q9ULX3	NOB1	RNA-binding protein NOB1;NOB1;ortholog	RNA-BINDING PROTEIN NOB1 (PTHR12814:SF2)		*Homo sapiens*
HUMAN|HGNC=30646|UniProtKB=Q7KZF4	SND1	Staphylococcal nuclease domain-containing protein 1;SND1;ortholog	STAPHYLOCOCCAL NUCLEASE DOMAIN-CONTAINING PROTEIN 1 (PTHR12302:SF2)	nucleic acid binding(PC00171);transcription cofactor(PC00217)	*Homo sapiens*
HUMAN|HGNC=8609|UniProtKB=O95453	PARN	Poly(A)-specific ribonuclease PARN;PARN;ortholog	POLY(A)-SPECIFIC RIBONUCLEASE PARN (PTHR15092:SF26)	mRNA polyadenylation factor(PC00146)	*Homo sapiens*
HUMAN|HGNC=25495|UniProtKB=Q9NW82	WDR70	WD repeat-containing protein 70;WDR70;ortholog	WD REPEAT-CONTAINING PROTEIN 70 (PTHR16017:SF0)		*Homo sapiens*
HUMAN|HGNC=278|UniProtKB=P35368	ADA1B	Alpha-1B adrenergic receptor;ADRA1B;ortholog	ALPHA-1B ADRENERGIC RECEPTOR (PTHR24248:SF17)	G-protein coupled receptor(PC00021)	*Homo sapiens*
HUMAN|HGNC=18518|UniProtKB=O75792	RNH2A	Ribonuclease H2 subunit A;RNASEH2A;ortholog	RIBONUCLEASE H2 SUBUNIT A (PTHR10954:SF7)	endoribonuclease(PC00094);hydrolase(PC00121)	*Homo sapiens*

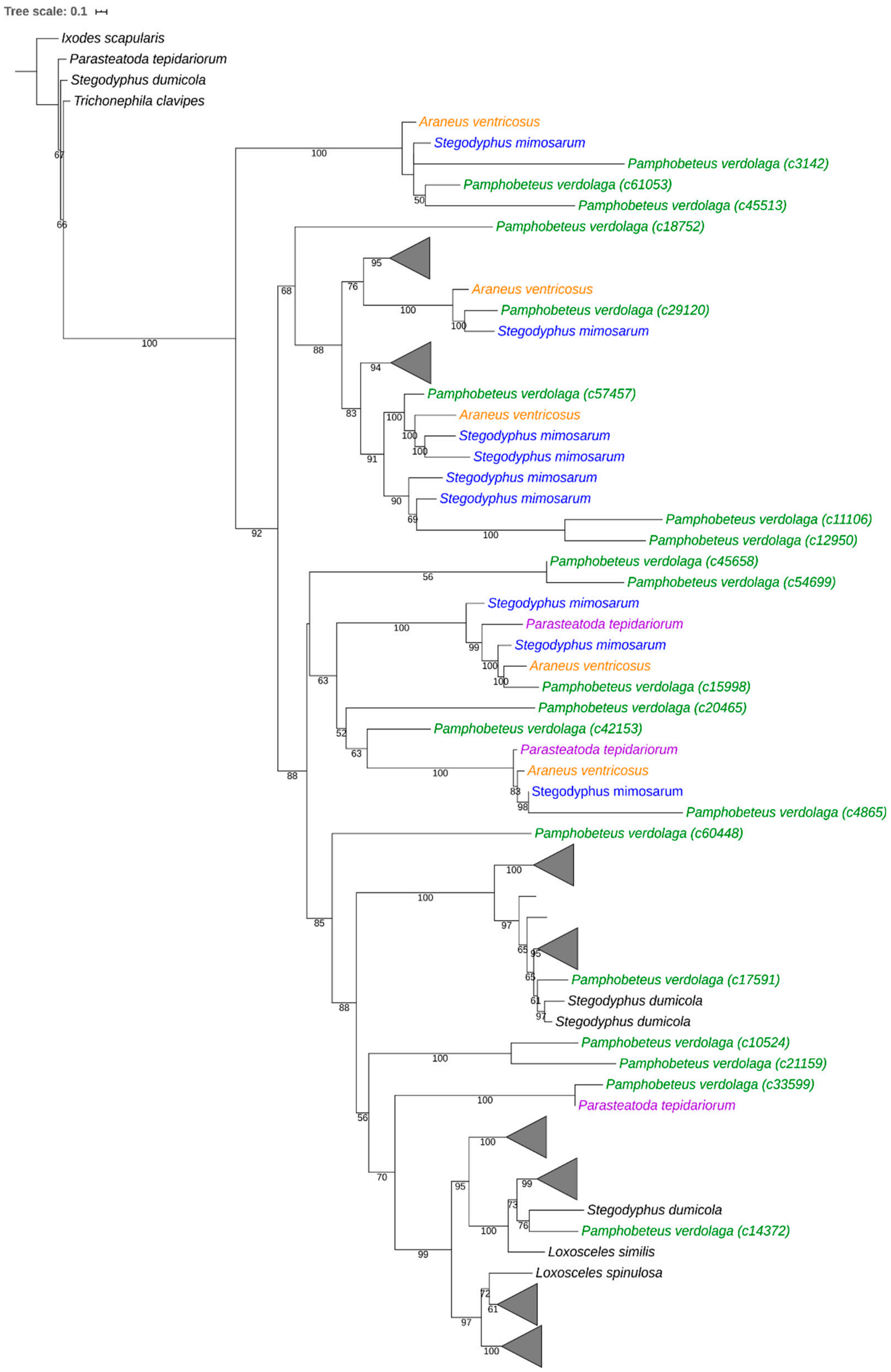

Figure S1. Phylogenetic tree of phospholipase A_2. The tree includes sequences of phospholipase A_2 reported for different spider families. *P. verdolaga* sequences (green) are orthologous with different species, grouped with good branch supports (> 50%), multiples duplication event underwent during the evolution of phospholipase A_2 in *P. verdolaga*. The triangle represents collapsed sequences from other species used to build the tree. Phospholipase A_2 from *Ixodes scapularis* was used as outgroup.

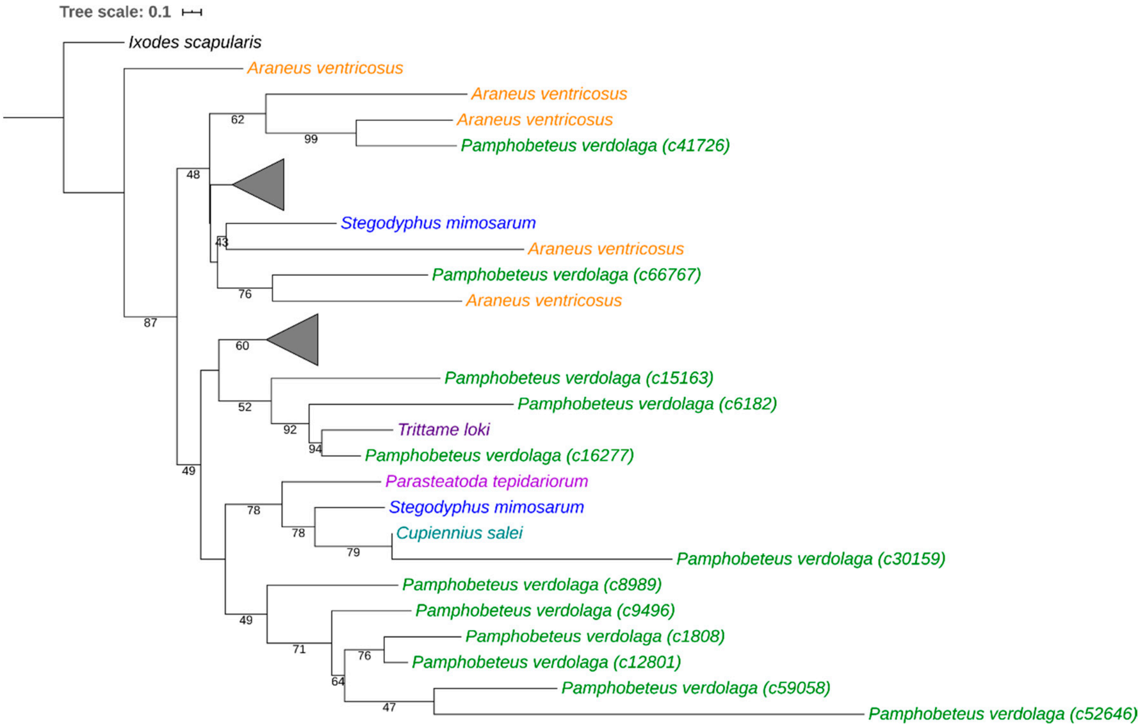

Figure S2. Phylogenetic tree of Kunitz-like protein family. The tree includes the Kunitz reported in data bases for different spider families. The Phylogenetic tree show that *P. verdolaga* sequences (green) are orthologous with different species, grouped with good branch supports (> 50%). The triangle represents collapsed sequences from other species used to build the tree. Kunitz from *Ixodes scapularis* was used as outgroup.

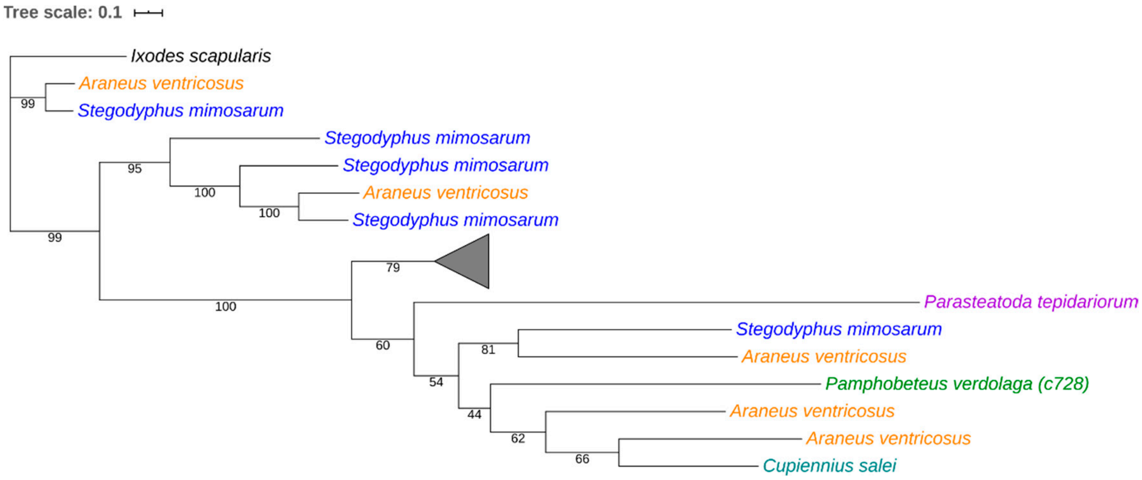

Figure S3. Phylogenetic tree of the metalloproteinase. The tree includes the metalloproteinase reported in data bases for different spider families. The Phylogenetic tree show that *P. verdolaga* sequence (green) is orthologous with the sequences reported for *Araneus ventricosus* with a branch support of 44%. The triangle represents collapsed sequences from other species used to build the tree. Metalloproteinase sequence form *Ixodes scapularis* was used as outgroup.

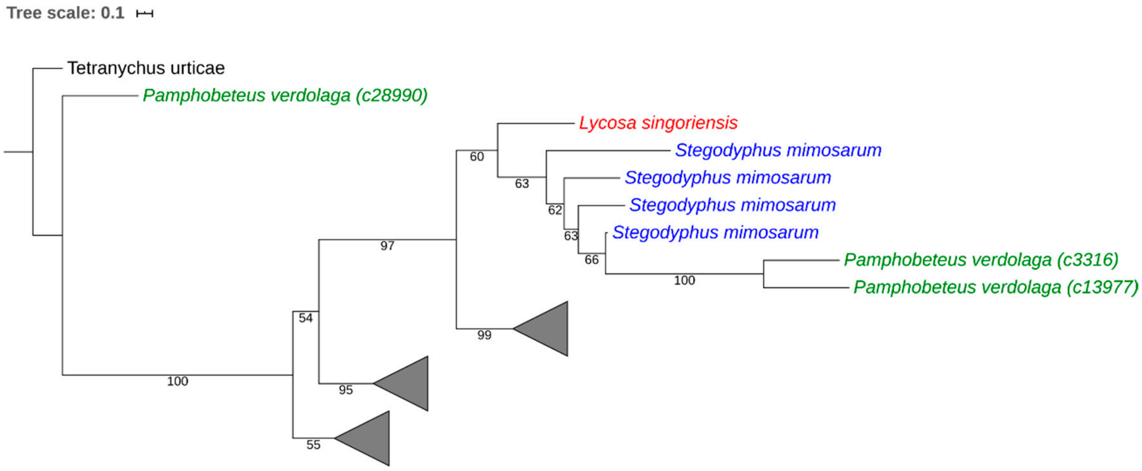

Figure S4. Phylogenetic tree of Lycotoxin-like protein. The tree includes the Lycotoxin-like proteins reported in data bases for different spider families. The Phylogenetic tree supports (branch support 100%) that *P. verdolaga* sequence c28990 is a basal protein that belong to the Lycotoxin-like family in spiders and multiples duplication events allow the diversification of the protein in different spider species. The triangle represents collapsed sequences from other species used to build the tree. Lycotoxin from *Tentranychus urticae* was used as outgroup.
