# Supplementary material for: Analysis of High Molecular Mass Compounds from the Spider Pamphobeteus verdolaga Venom Gland. A Transcriptomic and MS ID Approach
Source: Toxins (Basel). 2021 Jun 29;13(7):453. doi: 10.3390/toxins13070453 (PMC8309857; doi:10.3390/toxins13070453)
Supplement: Supplementary file 1 [file toxins-13-00453-s001.zip › toxins-1210969 supplementary 3.pdf]

Supplementary Materials: Analysis of High Molecular Mass Compounds from the Spider *Pamphobeteus verdolaga* Venom Gland. A Transcriptomic and MS ID Approach

Sebastian Estrada-Gomez, Leidy Johana Vargas-Muñoz, Cesar Segura Latorre, Monica Maria Saldarriaga-Cordoba and Claudia Marcela Arenas-Gómez

Supplementary material 3: Prediction of Cys-Cys formation of putative protein ORFs corresponding to phospholipases A_2, phospholipases D, phospholipases B, kunitz-type, hyaluronidases, lycotoxins toxins, CRISP proteins, Hephaestin-like protein and venom metalloproteinase. Residues highlighted in grey indicate the signal peptide, while magenta blue highlighted residues, indicates the propeptide according to Spider|ProHMM from the arachnoserver. Residues highlighted in yellow shows cysteines potentially forming disulfide bridges.

PhospholipaseA_2-1-Pverdolaga:
XLGTGKSPVVAVETIDVFRPDSLWDACKMVALGVSAIGQLLIDQATQTDGRVIGRAQAMCSM
LNIPYYRLNPQLTENVGLDTTDNKTLVKMLWETTAYMHSMRQELEQLCNNYEDSGLD

Sequence **inputSeq** Length **119** residues

Cysteines in this sequence: **3**

Disulfide Connectivity prediction

Step 1: Running PSI-BLAST with input sequence; click here to see the output

Step 2: Predicting secondary structure using PSIPRED; click here to see the output

Step 3: Disulfide Oxidation State Prediction; click here to see the results

Warning! The number of predicted half-*cystines* is lower than 2.

Step 4: Disulfide Bonds Prediction using a trained Neural Network

Disulfide bond scores

Cysteine sequence position	Distance	Bond	Score
27 - 60	33	SLWDACKMVAL-RAQAMCSMLNI	0.01102
27 - 110	83	SLWDACKMVAL-ELEQLCNNYED	0.75862
60 - 110	50	RAQAMCSMLNI-ELEQLCNNYED	0.89632

Step 5: Weighted matching

Predicted bonds

60 - 110	RAQAMCSMLNI - ELEQLCNNYED

Predicted connectivity

2-3

Introduction Instructions References About Help! Contacts

PhospholipaseA2-2-Pverdolaga:
MAAESVADDVLRCCVNLSSEFRDFYCPYDIPSVEGPPTPLEFARNWVSPNKPVIFRNAVKHWP
ALKKWTVSYLR

Sequence **inputSeq** Length **74** residues

Cysteines in this sequence: **3**

Disulfide Connectivity prediction

Step 1: Running PSI-BLAST with input sequence; click here to see the output

Step 2: Predicting secondary structure using PSIPRED; click here to see the output

Step 3: Disulfide Oxidation State Prediction; click here to see the results

Warning! The number of predicted half-*cystines* is lower than 2.

Step 4: Disulfide Bonds Prediction using a trained Neural Network

Disulfide bond scores

Cysteine sequence position	Distance	Bond	Score
13 - 14	1	DDVLRCCVNLS-DVLRCCVNLSS	0.01037
13 - 26	13	DDVLRCCVNLS-FRDFYCPYDIP	0.03657
14 - 26	12	DVLRCCVNLSS-FRDFYCPYDIP	0.82076

Step 5: Weighted matching

Predicted bonds

14 - 26	DVLRCCVNLSS - FRDFYCPYDIP

Predicted connectivity

2-3

Introduction | Instructions | References | About | Help! | Contacts

© Boston College

PhospholipaseA$_2$-3-Pverdolaga:
MLSLSTHSRFVVSVFTLLSLLISSTTSYRIPKRSVRPSPVIFVPGDGGSQLQAKLNKPETVHYYCN
KKTDYYFDLWLNLELLVPYVLDCWIDNMRLIYDNVTRKTTNAPGVDIRVPGFGNTSTVEWLD
PSQIAPSAYFVRIVQGLVDEGYTRGVDLKGAPYDYRKAPNEMANYYKNVKQMTEEMYFKLN
KTRITYVCHSMGCPVMLYFFNRQTQDWKDTHVKALITLGGAWGGAVKAMKAFASGENLGV
YVINHLLLRKEQRTSPSLAYMTPSDTFWKKDEILVVTEKQNYTIGNYYDFFQDIRFPVGWEMW
KDTYNLTRDLIPPGVEVHCMHGVNVSTIERLVYKHLEFPDSNPTLIQGDGDGTVNLRSLEGCL
RWKGNQKQKVVHKPLNNVDHMGVLYDDDVIQYIKQVVSS

Sequence **inputSeq** Length **415** residues

Cysteines in this sequence: **6**

Disulfide Connectivity prediction

Step 1: Running PSI-BLAST with input sequence; click here to see the output

Step 2: Predicting secondary structure using PSIPRED; click here to see the output

Step 3: Disulfide Oxidation State Prediction; click here to see the results

Warning! The number of predicted half-*cystines* is lower than 2.

Step 4: Disulfide Bonds Prediction using a trained Neural Network

Step 5: Weighted matching

Predicted bonds

65 - 89	TVHYYCNKKTD - PYVLDCWIDNM
197 - 202	RITYVCHSMGC - CHSMGCPVMLY
331 - 374	GVEVHCMHGVN - RSLEGCLRWKG

Predicted connectivity

1-2, 3-4, 5-6

Introduction | Instructions | References | About | Help! | Contacts

© Boston College

PhospholipaseA$_2$-4-Pverdolaga:
XFVNNICTSSVLDTRKGRAGLILNPLRGLSLIPCFNFSPFSPTSPSDDMLFKGLTEAVPTQSKTLY
LVDGGLTFNLPFPLLLRPQRGIDVYLAFDFSSRDADH

Sequence **inputSeq** Length **103** residues

Cysteines in this sequence: **2**

Disulfide Connectivity prediction

Step 1: Running PSI-BLAST with input sequence; click here to see the output

Step 2: Predicting secondary structure using PSIPRED; click here to see the output

Step 3: Disulfide Oxidation State Prediction; click here to see the results

Warning! The number of predicted half-*cystines* is lower than 2.

Step 4: Disulfide Bonds Prediction using a trained Neural Network

Disulfide bond scores

Cysteine sequence position	Distance	Bond	Score
7 - 34	27	FVNNICTSSVL-LSLIPCFNFSP	0.15031

Step 5: Weighted matching

Predicted bonds

7 - 34	FVNNICTSSVL - LSLIPCFNFSP

Predicted connectivity

1-2

Introduction Instructions References About Help! Contacts

© Boston College

PhospholipaseA$_2$-5-Pverdolaga:
XPSQSKKCTCSFDCSGGDSMEIVPQNCRVLCLDGGGIRGLVLIQLLDQLEKVLGIPVNLCFDWI
AGTSTGGVLALLLAQGKSVKECRCLYFRLKDRVFVGMRPYDAEPLEKILQKELGYETMMSDV
TGARVMVTATKSDRHPAELHVFRNYDSPMEILTQEDLDPFHNTPLPKPSEQLVWKVARATGS
APTYFRAFGAFLDGGLISNNPTLDALTEIHQCNQAYRVTHQEEKIKEIDX

Sequence **inputSeq** Length **239** residues
Cysteines in this sequence: **9**
**Disulfide Connectivity prediction**
**Step 1:** Running PSI-BLAST with input sequence; click here to see the output
**Step 2:** Predicting secondary structure using PSIPRED; click here to see the output
**Step 3:** Disulfide Oxidation State Prediction; click here to see the results
**Step 4:** Disulfide Bonds Prediction using a trained Neural Network

**Step 5:** Weighted matching	
**Predicted bonds**	
8 - 10	SQSKKCTCSFD - SKKCTCSFDCS
14 - 27	TCSFDCSGGDS - IVPQNCRVLCL
31 - 88	NCRVLCLDGGG - VKECRCLYFRL
60 - 86	IPVNLCFDWIA - KSVKECRCLYF
**Predicted connectivity**	
1-2, 3-4, 5-8, 6-7	

Introduction | Instructions | References | About | Help! | Contacts

© Boston College

PhospholipaseA₂-6-Pverdolaga:

MTICTSNSKYLFVLQILWIILPFSLAFNVRKWANNSDDCNYDLQVENDGPVTLDVPITFYAVLE
CASAEEHYVYIFQDNAVPPHRIQVDGSTGANVSFVYNANIYRPGVYILKVSVFSGMWSPVLVG
IASTSSTFVISEYIPGSLNISDIKVRNQGGSLYISSGSVTNLTINLHYPSSVYPLLETSYSWNVEKDQ
FITVDPFIIYNFTQPGTYRISVSAVARVPVYNVLAVPTQIMKYKWGYFNTVATVKDSMTAVNLT
GNTYLKHGQLLNLDVSCTGSGPFEYCWKIFQPFENVTDLTCPSPIVTTKCSFPIIYYFQESGNYQ
VAILVDNYITSIQRNIEVHVYDVSLKPQLSTVILPLVCAVLAIFIITIGIVIHIRENQQFDIETADFD
FLQSDVIVVETFWEKMYHSILQVLCLRREVQSNYYLRIVSPDASSSSHYGSVPXIGAVRRIIIVTV
WMIGRCHHVSKSLTAEFLYWFLRLRFQHTVKEKLFDSPNASTWNWPHFRNCNYLLVL

Sequence **inputSeq** Length **516** residues

Cysteines in this sequence: **11**

Disulfide Connectivity prediction

Step 1: Running PSI-BLAST with input sequence; click here to see the output

Step 2: Predicting secondary structure using PSIPRED; click here to see the output

Step 3: Disulfide Oxidation State Prediction; click here to see the results

Step 4: Disulfide Bonds Prediction using a trained Neural Network

Step 5: Weighted matching

Predicted bonds

4 - 39	XXMTICTSNSK – NNSDDCNYDLQ
65 - 309	YAVLECASAEE – IVTTKCSFPII
276 - 464	NLDVSCTGSGP – WMIGRCHHVSK
300 - 362	VTDLTCPSPIV – ILPLVCAVLAI
417 - 509	ILQVLCLRREV – PHFRNCNYLLV

Predicted connectivity

1-2, 3-7, 4-10, 6-8, 9-11

Introduction | Instructions | References | About | Help! | Contacts

© Boston College

PhospholipaseA2-7-Pverdolaga:
MTLGAPNIRRKIKRDLLDLADMFEELTQLDPTEYIPYGNWCGYGGDGEILDRIDRCCEIHDRC
YGKVSENVCSNEQVHIINYQWNRENDTITCDGNTSKCEMEACMCDRDVVLCIHKHNGDYSH
EVRYVDSTKPKTSGVPQWSSMNKDCTKRSDGSEIPLMGRMGNMSLIFX

Sequence **inputSeq** Length **173** residues

Cysteines in this sequence: **11**

Disulfide Connectivity prediction

Step 1: Running PSI-BLAST with input sequence; click here to see the output

Step 2: Predicting secondary structure using PSIPRED; click here to see the output

Step 3: Disulfide Oxidation State Prediction; click here to see the results

Step 4: Disulfide Bonds Prediction using a trained Neural Network

Step 5: Weighted matching

Predicted bonds

56 - 72	DRIDRCCEIHD - VSENVCSNEQV
57 - 100	RIDRCCEIHDR - GNTSKCEMEAC
63 - 93	EIHDRCYGKVS - NDTITCDGNTS
105 - 149	CEMEACMCDRD - SMNKDCTKRSD
107 - 114	MEACMCDRDVV - RDVVLCIHKHN

Predicted connectivity

2-5, 3-7, 4-6, 8-11, 9-10

Introduction | Instructions | References | About | Help! | Contacts

© Boston College

PhospholipaseA2-8-Pverdolaga:
MGFLLTAALTFILLAGYSPFSAEKPLQIRRNKRSLFDLNDMIKQLTGRSGLDFIGYGNYCGFGGE
GKPVDDIDRCCKMHDICYDFAQNDDCAEDPNVVYKIKYGWQQKSFGVQCSFSQSKCMKVV
CICDVRFAKCLKNYINEYNNSNKHEKDLQELLEEVQQMSK

Sequence **inputSeq** Length **166** residues

Cysteines in this sequence: **10**

Disulfide Connectivity prediction

Step 1: Running PSI-BLAST with input sequence; click here to see the output

Step 2: Predicting secondary structure using PSIPRED; click here to see the output

Step 3: Disulfide Oxidation State Prediction; click here to see the results

Step 4: Disulfide Bonds Prediction using a trained Neural Network

Step 5: Weighted matching

Predicted bonds

60 - 126	GYGNYCGFGGE – CMKVVCICDVR
75 - 91	DDIDRCCKMHD – AQNDDCAEDPN
76 - 121	DIDRCCKMHDI – FSQSKCMKVVC
82 - 114	KMHDICYDFAQ – SFGVQCSFSQS
128 - 135	KVVCICDVRFA – VRFAKCLKNYI

Predicted connectivity

1-8, 2-5, 3-7, 4-6, 9-10

Introduction | Instructions | References | About | Help! | Contacts

© Boston College

Mature protein disulfide pattern

Step 5: Weighted matching

Predicted bonds	
11 - 77	GYGNYCGFGGE – CMKVVCICDVR
26 - 42	DDIDRCCKMHD – AQNDDCAEDPN
27 - 72	DIDRCCKMHDI – FSQSKCMKVVC
33 - 65	KMHDICYDFAQ – SFGVQCSFSQS
79 - 86	KVVCICDVRFA – VRFAKCLKNYI

Predicted connectivity

1-8, 2-5, 3-7, 4-6, 9-10

Introduction | Instructions | References | About | Help! | Contacts

© Boston College

PhospholipaseA2-9-Pverdolaga:
MERKYLLYCAVVLGSFIITYPQSLFAGVKNVLDSVNAIVEEVSLGLRSLSAGLDFVDQFVQTAG
SEECLFHCPSGKKLVPNQKYKPVPSGCGAYGVTLSVKNSPQKEFTECCNYHDICYGTCLSKKEI
CDEKFDKCLNKACAKQAKEIGEKKFGDCKMAAKVFYAGTVALGCKAFLDAQAEACICPEA
WSVRLCVRSTRQICVVYGEDAIAPRTA

Sequence **inputSeq** Length **216** residues

Cysteines in this sequence: **17**

Disulfide Connectivity prediction

Step 1: Running PSI-BLAST with input sequence; click here to see the output

Step 2: Predicting secondary structure using PSIPRED; click here to see the output

Step 3: Disulfide Oxidation State Prediction; click here to see the results

Step 4: Disulfide Bonds Prediction using a trained Neural Network

Step 5: Weighted matching

Predicted bonds

9 - 68	KYLLYCAVVLG - AGSEECLFHCP
72 - 172	ECLFHCPSGKK - TVALGCKAFLD
91 - 111	PVPSGCGAYGV - KEFTECCNYHD
112 - 202	EFTECCNYHDI - STRQICVVYGE
118 - 183	NYHDICYGTCL - AQAEACICPEA
122 - 141	ICYGTCLSKKE - CLNKACAKQAK
136 - 156	EKFDKCLNKAC - KKFGDCKMAAK
185 - 194	AEACICPEAWS - WSVRLCVRSTR

Predicted connectivity

1-2, 3-13, 4-5, 6-17, 7-14, 8-11, 10-12, 15-16

Introduction | Instructions | References | About | Help! | Contacts

© Boston College

Mature protein disulfide pattern

Sequence **inputSeq** Length **189** residues

Cysteines in this sequence: **16**

Disulfide Connectivity prediction

Step 1: Running PSI-BLAST with input sequence; click here to see the output

Step 2: Predicting secondary structure using PSIPRED; click here to see the output

Step 3: Disulfide Oxidation State Prediction; click here to see the results

Step 4: Disulfide Bonds Prediction using a trained Neural Network

Step 5: Weighted matching

Predicted bonds

42 - 103	AGSEECLFHCP - SKKEICDEKFD
46 - 146	ECLFHCPSGKK - TVALGCKAFLD
65 - 85	PVPSGCGAYGV - KEFTECCNYHD
86 - 176	EFTECCNYHDI - STRQICVVYGE
92 - 157	NYHDICYGTCL - AQAEACICPEA
96 - 115	ICYGTCLSKKE - CLNKACAKQAK
110 - 130	EKFDKCLNKAC - KKFGDCKMAAK
159 - 168	AEACICPEAWS - WSVRLCVRSTR

Predicted connectivity

1-8, 2-12, 3-4, 5-16, 6-13, 7-10, 9-11, 14-15

Introduction | Instructions | References | About | Help! | Contacts

© Boston College

PhospholipaseA2-10-Pverdolaga:
FGYIMVLKNGSLSNDSEEVATGENLTMIAKILDGNGYLKDADIRCTWSIDMERFDLEGTSLNY
TYYDPGMSYIAVAVFATLPSSKTVFGLFTKELVVKVPVSDITISGNPFIHHNEVLNLNVSWTGTP
PFEYCWDIINSNETVEGNFTCMVIVTYDTSFPVTRYFQKNGTYTMAIHVSNDVKLVKRNMEIIV
FSVLPKSQLSTVIIPIVCSLLTLVIIAIGIAYYRQQRRQLIVEVASFDFHDNSDYRERTFFEQLWDS
FRCRGCCSPSLSVRSDCLPSENEPLLT

Sequence **inputSeq** Length **288** residues

Cysteines in this sequence: **8**

Disulfide Connectivity prediction

Step 1: Running PSI-BLAST with input sequence; click here to see the output

Step 2: Predicting secondary structure using PSIPRED; click here to see the output

Step 3: Disulfide Oxidation State Prediction; click here to see the results

Step 4: Disulfide Bonds Prediction using a trained Neural Network

Step 5: Weighted matching

Predicted bonds

45 - 277	DADIRCTWSID - SVRSDCLPSEN
133 - 263	PPFEYCWDIIN - WDSFRCRGCCS
149 - 266	EGNFTCMVIVT - FRCRGCCSPSL
210 - 267	IIPIVCSLLTL - RCRGCCSPSLS

Predicted connectivity

1-8, 2-5, 3-6, 4-7

Introduction | Instructions | References | About | Help! | Contacts

© Boston College

PhospholipaseA2-11-Pverdolaga:
XGDLVDTPDPYVVLKVPGSPNGKKRTKYFNNTINPTWKETFTFVLDPEKNYELEVILMDANYT
IDQRLGX

No disulfide bonds are possible for this sequence, the prediction will not be attempted

Clote's structural bioinformatics Lab - Author's homepage

PhospholipaseA$_2$-12-Pverdolaga:
MSIIRDILGGFRKVSAQVDDPFRVLEVNVEDYLTYDVVCREDCLVLYKANDRGVMKLEIVVQL
HINHSSNKNNKVYSLHRSEDETNCQILFSQMCQKIPILIDYVPEVGLSKMALQNVSQVIRENLA
WNAAHIAAHFGYTDCFKYKTMASEISEPCEGTLQTPLHVAIKASQFPSVVALVALDVVMDIV
DCNGDSIFHYAATTTKEIIQALSVKPCVPVINMLNHDGHTPLHLACMADKPECVKELLRAGA
DVNMASIVDVDEVDRAQAAEMPSKLLSDVMHTHAQRLYMDDMKTGGTPLHWSKTSELTAI
LIEYGCHIDAKNFEGNTALHVMVLRNRISCAVTLLSHGANVDIQGADGNTPLHLAVKSGDIY
LVYAFVAFGANVNAINNKGETPRHILATEKRPGFEEMLYALHIVGAERCQRRTPWCKDGCEP
GQHFNGIPSENPPVLNKTTLLDDLLGAT

Sequence **inputSeq** Length **464** residues

Cysteines in this sequence: **15**

Disulfide Connectivity prediction

Step 1: Running PSI-BLAST with input sequence; click here to see the output

Step 2: Predicting secondary structure using PSIPRED; click here to see the output

Step 3: Disulfide Oxidation State Prediction; click here to see the results

Warning! The number of predicted half-*cystines* is lower than 2.

Step 4: Disulfide Bonds Prediction using a trained Neural Network

Step 5: Weighted matching

Predicted bonds

39 - 341	TYDVVCREDCL - RNRISCAVTLL
43 - 429	VCREDCLVLYK - RRTPWCKDGCE
87 - 235	EDETNCQILFS - PLHLACMADKP
95 - 422	LFSQMCQKIPI - VGAERCQRRTP
142 - 433	FGYTDCFKYKT - WCKDGCEPGQH
191 - 317	MDIVDCNGDSI - LIEYGCHIDAK
216 - 242	LSVKPCVPVIN - ADKPECVKELL

Predicted connectivity

1-12, 2-14, 3-9, 4-13, 5-15, 7-11, 8-10

Introduction | Instructions | References | About | Help! | Contacts

© Boston College

PhospholipaseA_2-13-Pverdolaga:
XRLHELDEPADAEPLIQNSGLPAKYPSFKMMLELINNCSQLELPVHKVASSIATNQSEPNNTIS
KWSPWALWNGIVPGTKWCGVGDIASTFEELGSQAVVDSCCRAHDHCPVKLKAFRVGYGMI
NLSFYTKSHCDCDRLFHSCLKQTKNKLANAVGNFYFNFIRVQCLKERKVYVCVENRTDVDGL
NECIRWSVDPDSRKX

Sequence **inputSeq** Length **202** residues
Cysteines in this sequence: **11**
**Disulfide Connectivity prediction**
**Step 1:** Running PSI-BLAST with input sequence; click here to see the output
**Step 2:** Predicting secondary structure using PSIPRED; click here to see the output
**Step 3:** Disulfide Oxidation State Prediction; click here to see the results
**Step 4:** Disulfide Bonds Prediction using a trained Neural Network

**Step 5:** Weighted matching	
**Predicted bonds**	
38 - 110	ELINNCSQLEL – RAHDHCPVKLK
82 - 104	PGTKWCGVGDI – VVDSCCRAHDH
103 - 134	AVVDSCCRAHD – YTKSHCDCDRL
136 - 143	KSHCDCDRLFH – RLFHSCLKQTK
176 - 189	RKVYVCVENRT – DGLNECIRWSV
**Predicted connectivity**	
1-5, 2-4, 3-6, 7-8, 10-11	

Introduction | Instructions | References | About | Help! | Contacts

© Boston College

PhospholipaseA$_2$-14-Pverdolaga:
XCRAHDLCDDTLAPGETKHNLTNRSTFTKLNCQCDQEFYECLQKVDSLVSNSIGNLYFNVLR
RGCYEYDHPLTKCKSYRT

Sequence **inputSeq** Length **81** residues

Cysteines in this sequence: 7

Disulfide Connectivity prediction

Step 1: Running PSI-BLAST with input sequence; click here to see the output

Step 2: Predicting secondary structure using PSIPRED; click here to see the output

Step 3: Disulfide Oxidation State Prediction; click here to see the results

Warning! The number of predicted half-*cystines* is lower than 2.

Step 4: Disulfide Bonds Prediction using a trained Neural Network

Step 5: Weighted matching

Predicted bonds

2 - 32	XXXXXCRAHDL - FTKLNCQCDQE
8 - 65	RAHDLCDDTLA - VLRRGCYEYDH
34 - 41	KLNCQCDQEFY - QEFYECLQKVD

Predicted connectivity

1-3, 2-6, 4-5

Introduction | Instructions | References | About | Help! | Contacts

© Boston College

PhospholipaseA2-15-Pverdolaga:
MPRSQNAFSRNSSIYSSLYTRSVNISKEKRKLLLHKTYCTSKRSSDLNVSKPSSDKIPGNSIFSTIW
KSVVVASSMLRPGLSVTPPKSIPIREFISKVGNVVSTEKYSKLLQPYLKLRSVNSEENVRQTTEKS
RTCSKPGVSQAGNVISGEKESVFEQAAAEWITSANLQEKKQENLSNQKEKKSENLPKVLISKAS
LASRSRFLVRSLSCASSCSSQMLRLEEVCKHLLQHPQEKGTLVKEGLIRVALRLRRKSSNTDIQT
QACVALTLLGYHEPPGGQGIRILSIDGGGTRGLMAIEILRQLQARTGKTVHEMFDYICGVSSGA
ILTFLLGGLRLSPDECESLYRELSLEVFKASGIWGAGRLMWYHAYYDTSMWVDVLRKTFGDK
MLIDSVKEKSSPKLAAISAVMNLPALRAFVFRNYDYPIRVQSQYIGSANYRMWEAIRASGAAP
GYFEEFHLNHLLHQDGGIMINNPTALAIHEARLLWPSDYIQCVFSLGSGRFTPATNTAFTSTTL
KTKVQKVIDSATDTEAVHISMNDLLSPGTYFRFNPYLTEFLHLDENRPDKLHQLKMDAQMYL
RRNEHKLEQSIKVLTRPRSTLKKINDWIQLQKTLLL

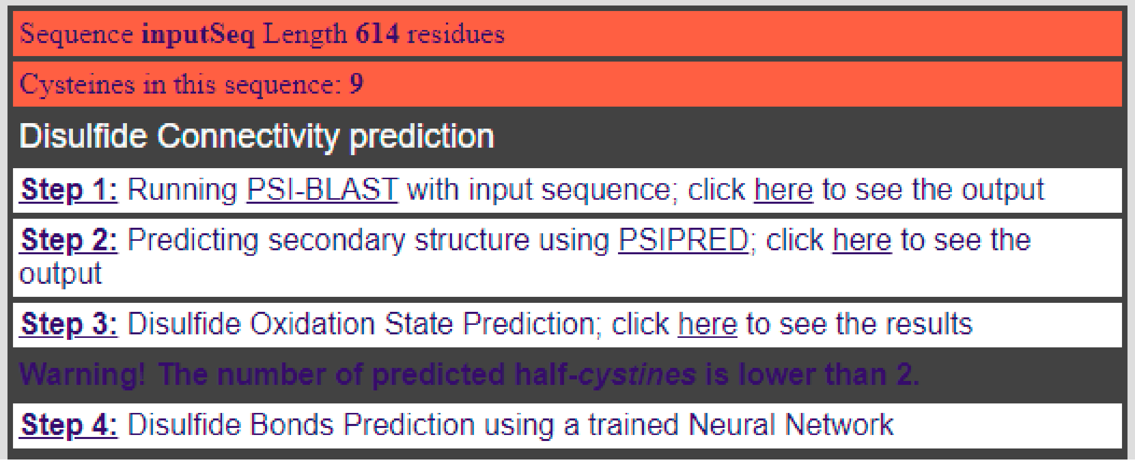
Sequence **inputSeq** Length **614** residues

Cysteines in this sequence: **9**

Disulfide Connectivity prediction

Step 1: Running PSI-BLAST with input sequence; click here to see the output

Step 2: Predicting secondary structure using PSIPRED; click here to see the output

Step 3: Disulfide Oxidation State Prediction; click here to see the results

Warning! The number of predicted half-*cystines* is lower than 2.

Step 4: Disulfide Bonds Prediction using a trained Neural Network

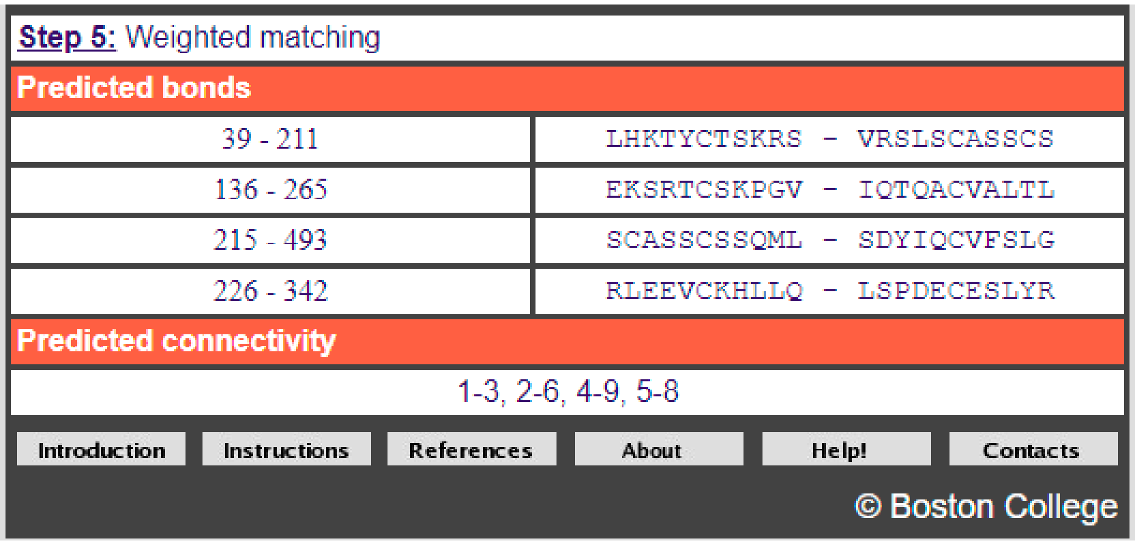
Step 5: Weighted matching

Predicted bonds

39 - 211	LHKTYCTSKRS - VRSLSCASSCS
136 - 265	EKSRTCSKPGV - IQTQACVALTL
215 - 493	SCASSCSSQML - SDYIQCVFSLG
226 - 342	RLEEVCKHLLQ - LSPDECESLYR

Predicted connectivity

1-3, 2-6, 4-9, 5-8

Introduction Instructions References About Help! Contacts

© Boston College

PhospholipaseA$_2$-16-Pverdolaga:

MAEEEYHLMCSILAHGSDIRSVTTSYVPLGGIVTGSRDKTIKLWRPTGTTFTEEHCMRGASHFIS
SLCALPPSDQYPDGLILAGSNDCAIYGFSLDSSEPILKLLGHSENVCALVAGNLGTIVSGSWDKT
ARVWHGQRCVATLSGHTQAVWAVALLPDHALVLTGSADKAVFLWNNGKCERKFIGHEDC
VRGLTVISDLEFLSCSNDTTVRRWQTSGECLGIYTGHTDYVYDICLSSCREYFISCSEDQTVKVW
KENVCVQTIKLPAKSLWAVTYLYNGDIAVGGSDGSVRVFTKDKSRRASPAEEARFNEEIVSMN
SKNMKQNIGDLELDDVPGPDALLQDGTSDGQTQLCKVGNEVSVFQWSVKEHKWLKLGKVL
DALDNRPAKGKTVYEGKEYDYVFTIDVAEGKLLKLPYNDTEDPWLVAHKFIEKHDLNPMFLD
QIANFIINNSKSAGVQAESMSEFSDPFTGASRYIPSNVGKPSSLASNHGDNSSIQELPKSNPTGN
GDIEKASTGAHFPLLTYVTFDTANTNGIRAKLCEFTEKIEKSQQLSIEKIEHMLLLLDYPQAITD
DQMLSLEKALSWPAEFVFPALDVLRLAVRAEPVNSRVSKDGGVGLINHLLRYVSTGNPVSNQ
MLVLRTLSNFFVCPSGEQLLVSQAKKVLSLTRSCCASKNKHVQIALATLYANYSVAFQKSTSSE
DTYCKDMYLNDAVEALKQFNEPEALFRLIVCIGTAVQDKYCLQVAKALKIGEIVQSVLERCEV
SKIQDFGATLIDIVSN

Sequence **inputSeq** Length **775** residues

Cysteines in this sequence: **23**

Disulfide Connectivity prediction

Step 1: Running PSI-BLAST with input sequence; click here to see the output

Step 2: Predicting secondary structure using PSIPRED; click here to see the output

Step 3: Disulfide Oxidation State Prediction; click here to see the results

Warning! The number of predicted half-*cystines* is lower than 2.

Step 4: Disulfide Bonds Prediction using a trained Neural Network

Step 5: Weighted matching

Predicted bonds

10 - 88	EYHLMCSILAH - AGSNDCAIYGF
56 - 139	FTEEHCMRGAS - WHGQRCVATLS
68 - 179	FISSLCALPPS - WNNGKCERKFI
112 - 644	HSENVCALVAG - SNFFVCPSGEQ
204 - 259	LEFLSCSNDTT - WKENVCVQTIK
219 - 665	QTSGECLGIYT - SLTRSCCASKN
234 - 238	YVYDICLSSCR - ICLSSCREYFI
245 - 726	EYFISCSEDQT - FRLIVCIGTAV
352 - 736	GQTQLCKVGNE - VQDKYCLQVAK
537 - 756	IRAKLCEFTEK - SVLERCEVSKI
666 - 699	LTRSCCASKNK - SEDTYCKDMYL

Predicted connectivity

1-4, 2-6, 3-7, 5-17, 9-14, 10-18, 11-12, 13-21, 15-22, 16-23, 19-20

Introduction | Instructions | References | About | Help! | Contacts

© Boston College

PhospholipaseD-1-Pverdolaga:
MKILKFLGCLIWYQVCVADEVDWRRPVWNIAHMVNANYQIDYYLDMGANSIEFDVAFDNS
GNARFTFHGVPCDCFRSCVRHEEIENYLEYMRHLTTPGDPKFQEKLVLLFMDLKVKGLSSRAR
TNAGFSIARKLVRHYWQNGTSAARAHVLMSIPSVDHMEVVRGFRDGLRVEGLSGYINKVGV
DFSGNEDLNSIRRALMSESISDRIWQGDGITNCLPRGTGRLREAIQRRDQPGLTHIEKVYWWTV
DKMSTMRATLRLSVDAMITNYPERLVSVLDEDEFSGRFRMATIDDNPWSKHELRTSALYALDE
GPTARGGNITTYFDKEDDELLIIASTITQTMAGINFTRGLEESIPX

Sequence **inputSeq** Length **358** residues

Cysteines in this sequence: **6**

Disulfide Connectivity prediction

Step 1: Running PSI-BLAST with input sequence; click here to see the output

Step 2: Predicting secondary structure using PSIPRED; click here to see the output

Step 3: Disulfide Oxidation State Prediction; click here to see the results

Step 4: Disulfide Bonds Prediction using a trained Neural Network

Step 5: Weighted matching

Predicted bonds

9 - 72	LKFLGCLIWYQ – FHGVPCDCFRS
16 - 74	IWYQVCVADEV – GVPCDCFRSCV
78 - 217	DCFRSCVRHEE – DGITNCLPRGT

Predicted connectivity

1-3, 2-4, 5-6

Introduction | Instructions | References | About | Help! | Contacts

© Boston College

Mature peptide disulfide pattern

Sequence **inputSeq** Length **332** residues

Cysteines in this sequence: **4**

Disulfide Connectivity prediction

Step 1: Running PSI-BLAST with input sequence; click here to see the output

Step 2: Predicting secondary structure using PSIPRED; click here to see the output

Step 3: Disulfide Oxidation State Prediction; click here to see the results

Step 4: Disulfide Bonds Prediction using a trained Neural Network

Step 5: Weighted matching

Predicted bonds

47 - 49	FHGVPCDCFRS – GVPCDCFRSCV
53 - 192	DCFRSCVRHEE – DGITNCLPRGT

Predicted connectivity

1-2, 3-4

Introduction | Instructions | References | About | Help! | Contacts

© Boston College

PhospholipaseD-2-Pverdolaga:
MANDISDAIYLLDQGANALEFDISFFNNGTVNRVYHGVPCDCFRVCTHEASLPDYLSTIRKITD
PQTGKYSQQMTFQFFDLKLQEVTPWGKYVAGLEIANHVIDYLWGNDTKRQLVRVLIFINDES
DKDVVLGVRNAFLQRGMKKFLDQVGFDGGTGTMKSIRDMWDSLGIRGNLWQGDGIFNCLS
EVYKDDRLREALHIRDSPNGFIDKVYHWTIDSRGRMRMSLRLGVDGMITNLPKDLIDVLNEDP
YSNIFRLATAKDDPFSRFHPSKSFK

Sequence **inputSeq** Length **275** residues

Cysteines in this sequence: **4**

Disulfide Connectivity prediction

Step 1: Running PSI-BLAST with input sequence; click here to see the output

Step 2: Predicting secondary structure using PSIPRED; click here to see the output

Step 3: Disulfide Oxidation State Prediction; click here to see the results

Step 4: Disulfide Bonds Prediction using a trained Neural Network

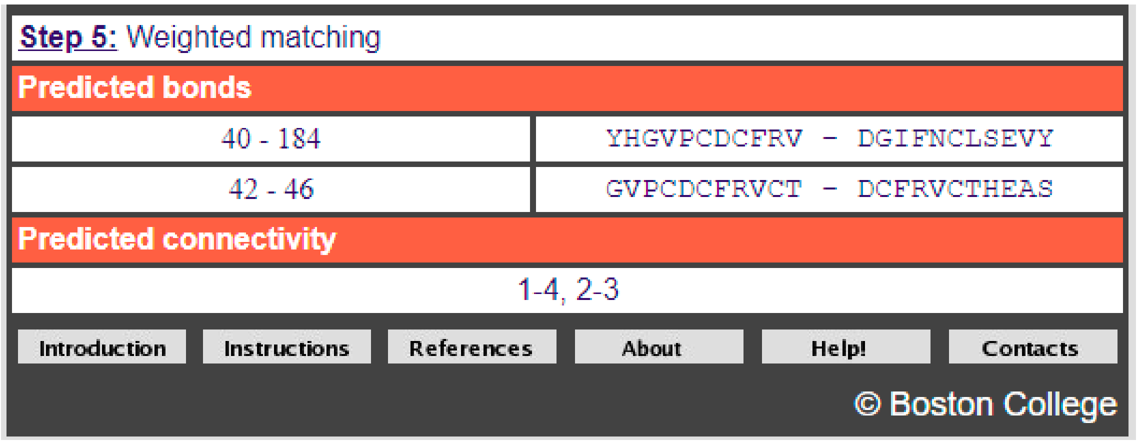

Step 5: Weighted matching

Predicted bonds	
40 - 184	YHGVPCDCFRV - DGIFNCLSEVY
42 - 46	GVPCDCFRVCT - DCFRVCTHEAS

Predicted connectivity

1-4, 2-3

Introduction Instructions References About Help! Contacts

© Boston College

PhospholipaseD-3-Pverdolaga:

EKRSGGYAVTSFVKSFRGIFTECCGNWKTRWLLVKDNFVAYIKPSDGQLKCVLLMDHDFSVK
SGKAETGKSNSLFISNMSRHLRLKCRSERQATEWAAEIERVVEKSGFEFTKVSRHGSFAPPRPH
SPCRWIIDGATYFDSVASALDRAKEEIFIADWWLTPEIYLKRPTFHGHYWQLDHILK

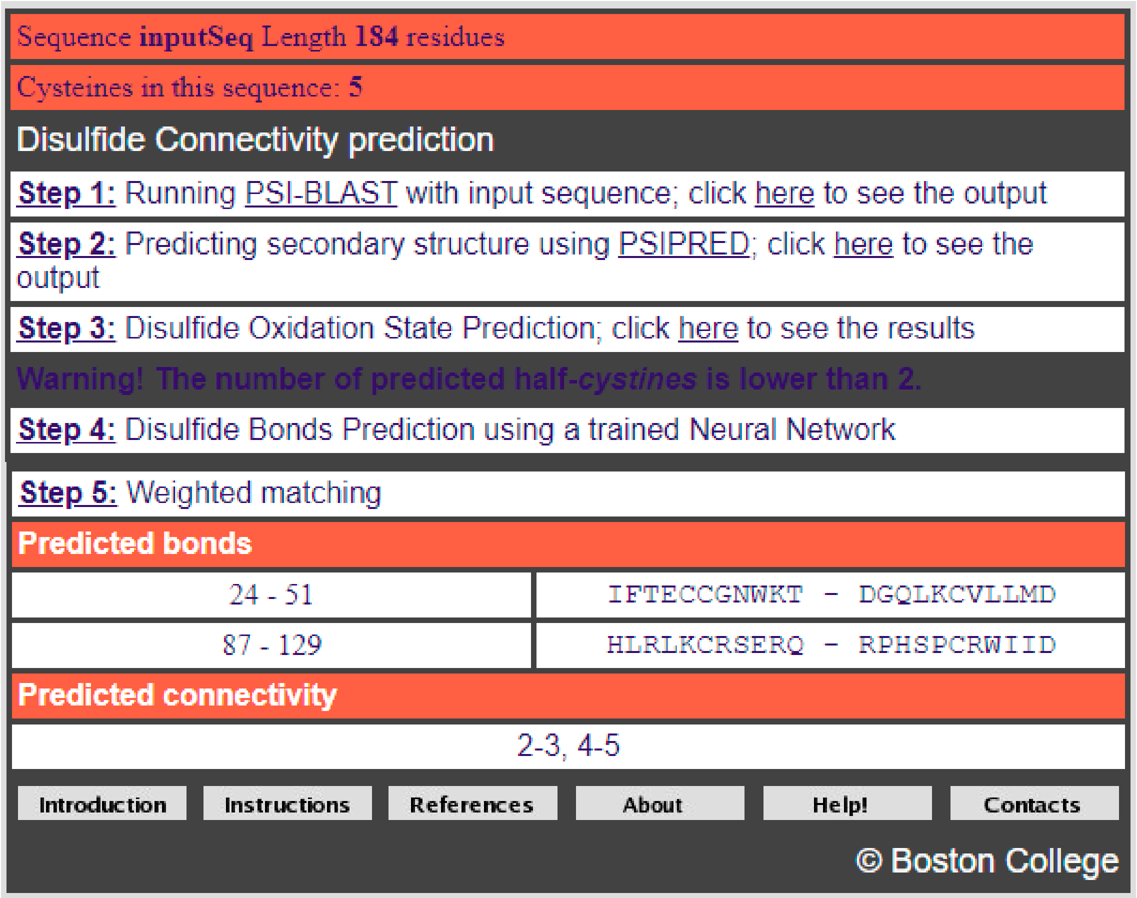

Sequence **inputSeq** Length **184** residues

Cysteines in this sequence: **5**

Disulfide Connectivity prediction

Step 1: Running PSI-BLAST with input sequence; click here to see the output

Step 2: Predicting secondary structure using PSIPRED; click here to see the output

Step 3: Disulfide Oxidation State Prediction; click here to see the results

Warning! The number of predicted half-*cystines* is lower than 2.

Step 4: Disulfide Bonds Prediction using a trained Neural Network

Step 5: Weighted matching

Predicted bonds	
24 - 51	IFTECCGNWKT - DGQLKCVLLMD
87 - 129	HLRLKCRSERQ - RPHSPCRWIID

Predicted connectivity

2-3, 4-5

Introduction Instructions References About Help! Contacts

© Boston College

PhospholipaseD-4-Pverdolaga:
XALPISKLDIKRHTSSIVPPPLFPVHKRSRSDSNVMTVPKKNQEFYQSRPHDDYHIKTLLKNIPS
KPQQPVHKLQAKVKHDENRAKQRWRVAVKKIQAISAFQNLESQVLLEQVRDGAHQDVCLS
SLPTPADVHRTIQEIALVHLGLERTYRLWHGKDYSNFIFKDLNKLNEPYTDSVNRYETPRMP
WHDVSCFLQGPAARDVARHFIQRWNFTKLRTAKFDDVYPLLLPKCYEFPDPIPPILSSEVGSIL
MADCQVLRSTSMWSAGIITTEYSILNAYKDAIMKAEHFIYIENQFFVSLQHGNKDVFNDISEC
LYQRIMKAHQENKRFCVYVIMPLLPAFEGEVGTGTGTLIQAVTHWNYSSICRGPRSLCQRLA
KSIQDPLSYISFYGLRNFGVLNNKLVTELIYVHSKLMIVDDKKAIIGSANINDRSLLGRRDSEIA
VLVNDSVFVESVMDGKPYKAGHFCSSLRKALFKEHLGLLGEKHSKVEX

Sequence **inputSeq** Length **490** residues

Cysteines in this sequence: **9**

Disulfide Connectivity prediction

Step 1: Running PSI-BLAST with input sequence; click here to see the output

Step 2: Predicting secondary structure using PSIPRED; click here to see the output

Step 3: Disulfide Oxidation State Prediction; click here to see the results

Warning! The number of predicted half-*cystines* is lower than 2.

Step 4: Disulfide Bonds Prediction using a trained Neural Network

Step 5: Weighted matching

Predicted bonds

123 - 193	AHQDVCLSSLP – WHDVSCFLQGP
232 - 255	LLLPKCYEFPD – ILMADCQVLRS
314 - 330	NDISECLYQRI – ENKRFCVYVIM
365 - 372	NYSSICRGPRS – GPRSLCQRLAK

Predicted connectivity

1-2, 3-4, 5-6, 7-8

Introduction | Instructions | References | About | Help! | Contacts

© Boston College

PhospholipaseB-1-Pverdolaga:
LEAIVLSLFRDNVVFLYGVFAPPRLVSASCSSVSLCMKNLSPEASDWKFCMMLTLWVAIALLV
HVCAASTEQKAYVTWDSSSYKFTVHSDPVENFVAYATFTNEINATGWSYLEVWTNETFPDSV
QAYSAGLAEGVLTADLLKKHWYNTVATYCDGEESYCDRLKIFLETNLDFMNYNIARRRKYV
PYWHQVALALEQLSGLEDGYNNVSGKPHTKLNVTGVLMVNIFGDLEDLEGILNKTVSSRPL
GSGSCSGLIKVLPNNEDLYVSQDSWNTYSSMLRVLKKYNISVHSGMDRGSPVIPGQVMSFSSY
PGLICSGDDFYTISSGLATMETTIGNGNSSLWKYIRAKGTVLEWLRSIVANRMARSGREWSRW
FSIMNSGTYNNQWMVVDYNKFLPGAPLQNDLLWVLEQLPGYIHSDDLTDVLRKQGYWPSY
NTPYFKDIFNLSGSQENADKYGDWFTYDKTPRALIFKRDHGTVTDVKSMIKLMRYNDYTHD
PLSRCNCTPPYSAENAISARCDLNPANGTYPFGALGHRSHGGIDMKLTTGSLFKNFEFVAFG
GPTYDSLPPFKWSESDFRTTERHEGHPDLWKFEPIVRKWSQ

Sequence **inputSeq** Length **598** residues

Cysteines in this sequence: **11**

Disulfide Connectivity prediction

Step 1: Running PSI-BLAST with input sequence; click here to see the output

Step 2: Predicting secondary structure using PSIPRED; click here to see the output

Step 3: Disulfide Oxidation State Prediction; click here to see the results

Step 4: Disulfide Bonds Prediction using a trained Neural Network

Step 5: Weighted matching

Predicted bonds

30 - 252	LVSASCSSVSL - LGSGSCSGLIK
36 - 315	SSVSLCMKNLS - YPGLICSGDDF
50 - 499	SDWKFCMMLTL - DPLSRCNCTPP
66 - 161	LLVHVCAASTE - GEESYCDRLKI
501 - 515	LSRCNCTPPYS - AISARCDLNPA

Predicted connectivity

1-7, 2-8, 3-9, 4-6, 10-11

Introduction | Instructions | References | About | Help! | Contacts

© Boston College

Kunitz-1-Pverdolaga:
MGIRNICIVISVFTVLFVLTVPPVFSADEKMDICKLPPESCIIDLRAQLKIPRWYFNGRLCTRFVYS GCDASENNFISRAQCRAKCGQARRRIRVPKT

Sequence **inputSeq** Length **99** residues

Cysteines in this sequence: 7

Disulfide Connectivity prediction

Step 1: Running PSI-BLAST with input sequence; click here to see the output

Step 2: Predicting secondary structure using PSIPRED; click here to see the output

Step 3: Disulfide Oxidation State Prediction; click here to see the results

Step 4: Disulfide Bonds Prediction using a trained Neural Network

Step 5: Weighted matching

Predicted bonds

7 - 69	GIRNICIVISV - FVYSGCDASEN
34 - 86	EKMDICKLPPE - QCRAKCGQARR
61 - 82	FNGRLCTRFVY - ISRAQCRAKCG

Predicted connectivity

1-5, 2-7, 4-6

Introduction Instructions References About Help! Contacts

© Boston College

Kunitz-2-Pverdolaga:
MGVRTVFLLFVAFSFAAGDLQNPIRDEVCSLKAEPGIPGKNIMCLAYFPKYYFNSAVGYCEKFI
FGGCGGNANSFSTKEECEKFCGSDDKR

Sequence **inputSeq** Length **92** residues

Cysteines in this sequence: **6**

Disulfide Connectivity prediction

Step 1: Running PSI-BLAST with input sequence; click here to see the output

Step 2: Predicting secondary structure using PSIPRED; click here to see the output

Step 3: Disulfide Oxidation State Prediction; click here to see the results

Step 4: Disulfide Bonds Prediction using a trained Neural Network

Step 5: Weighted matching

Predicted bonds

29 - 85	IRDEVCSLKAE - ECEKFCGSDDK
44 - 68	GKNIMCLAYFP - FIFGGCGGNAN
60 - 81	SAVGYCEKFIF - STKEECEKFCG

Predicted connectivity

1-6, 2-4, 3-5

Introduction | Instructions | References | About | Help! | Contacts

© Boston College

Kunitz-3-Pverdolaga:
MGIRNICSVISVFTVLFALTFPPVFLAGYHLDICRQRPDRGMCLVNMERWFFNGRFCSTFVYGG CGGNGNNFISKAQCMARCARG

Sequence **inputSeq** Length **86** residues
Cysteines in this sequence: 7
Disulfide Connectivity prediction
Step 1: Running PSI-BLAST with input sequence; click here to see the output
Step 2: Predicting secondary structure using PSIPRED; click here to see the output
Step 3: Disulfide Oxidation State Prediction; click here to see the results
Step 4: Disulfide Bonds Prediction using a trained Neural Network

Step 5: Weighted matching	
**Predicted bonds**	
34 - 82	YHLDICRQRPD – QCMARCARGXX
43 - 65	PDRGMCLVNME – FVYGGCGGNGN
57 - 78	FNGRFCSTFVY – ISKAQCMARCA
**Predicted connectivity**	
2-7, 3-5, 4-6	

Introduction | Instructions | References | About | Help! | Contacts

© Boston College

Mature peptide disulfide pattern

Step 5: Weighted matching	
**Predicted bonds**	
7 - 55	YHLDICRQRPD – QCMARCARGXX
16 - 38	PDRGMCLVNME – FVYGGCGGNGN
30 - 51	FNGRFCSTFVY – ISKAQCMARCA
**Predicted connectivity**	
1-6, 2-4, 3-5	

Introduction | Instructions | References | About | Help! | Contacts

© Boston College

Kunitz-4-Pverdolaga:
MLLMILVVASFQHSNADANGIDLCELDKEPGSCTSVINRYYFNRYSRRCERFIYTDCGGNSNNF
HYEFECERTCPGDLYIGDVCSLPKKVGPCRAAMPRYYFNKETGRCETFTYGGCSGNYNNFETK
EQCNSYCFQG

Sequence **inputSeq** Length **138** residues
Cysteines in this sequence: **12**
Disulfide Connectivity prediction
**Step 1:** Running PSI-BLAST with input sequence; click here to see the output
**Step 2:** Predicting secondary structure using PSIPRED; click here to see the output
**Step 3:** Disulfide Oxidation State Prediction; click here to see the results
**Step 4:** Disulfide Bonds Prediction using a trained Neural Network

**Step 5:** Weighted matching	
**Predicted bonds**	
24 - 74	NGIDLCELDKE - ECERTCPGDLY
33 - 57	KEPGSCTSVIN - FIYTDCGGNSN
49 - 70	RYSRRCERFIY - HYEFECERTCP
84 - 134	YIGDVCSLPKK - QCNSYCFQGXX
93 - 117	KKVGPCRAAMP - FTYGGCSGNYN
109 - 130	KETGRCETFTY - ETKEQCNSYCF
**Predicted connectivity**	
1-6, 2-4, 3-5, 7-12, 8-10, 9-11	

Introduction | Instructions | References | About | Help! | Contacts

© Boston College

Kunitz-5-Pverdolaga:
XYFLRNGCPSSTVCETTHSLGISRGNCCDSINSCHLIAKFPNXAEMHFTLACVFSLFCGICFANH
NVPDTDCSLPPDAGMCYAYFPMFFYDAPSGACINFIYGGCGGNANRFWTEEECMNRCAGVG
GTTKEPADEEKGPIFLGPVDEGKGTISQKPIDEGKEVIIQKPVDEGKGAIYPQPTGGGKGVIFPQ
PVAGGKGVIFQQPVGGGKGVTVQHAPQADICNQEKQPGNCSSQIIRYYFDKDSKKCDTFMYS
GCGKNDNNFNYKFECERTCSGEHDIGDTCNFKQDSGPCRAFFPRFYFSGESGQCEQFIYGGCQ
GNHNNFKTKEECLQFCTSGKGSPLP

Sequence **inputSeq** Length **342** residues

Cysteines in this sequence: **26**

Disulfide Connectivity prediction

Step 1: Running PSI-BLAST with input sequence; click here to see the output

Step 2: Predicting secondary structure using PSIPRED; click here to see the output

Step 3: Disulfide Oxidation State Prediction; click here to see the results

Step 4: Disulfide Bonds Prediction using a trained Neural Network

Step 5: Weighted matching

Predicted bonds

8 - 122	FLRNGCPSSTV - ECMNRCAGVGG
14 - 72	PSSTVCETTHS - VPDTDCSLPPD
27 - 255	ISRGNCCDSIN - FMYSGCGKNDN
28 - 52	SRGNCCDSINS - HFTLACVFSLF
34 - 58	DSINSCHLIAK - VFSLFCGICFA
61 - 268	LFCGICFANHN - NYKFECERTCS
81 - 105	PDAGMCYAYFP - FIYGGCGGNAN
97 - 118	APSGACINFIY - WTEEECMNRCA
222 - 272	PQADICNQEKQ - ECERTCSGEHD
231 - 315	KQPGNCSSQII - FIYGGCQGNHN
247 - 291	KDSKKCDTFMY - QDSGPCRAFFP
282 - 332	DIGDTCNFKQD - ECLQFCTSGKG
307 - 328	GESGQCEQFIY - KTKEECLQFCT

Predicted connectivity

1-14, 2-9, 3-18, 4-6, 5-7, 8-19, 10-12, 11-13, 15-20, 16-24, 17-22, 21-26, 23-25

Introduction | Instructions | References | About | Help! | Contacts

© Boston College

Kunitz-6-Pverdolaga:
VISVFTVLLALTFPPLFSADHHLDICELPADSGTCFVRLHRWYFNGESCTKFLYRGCGGNENHF
MTEVECMAKCGGA

Sequence **inputSeq** Length **78** residues
Cysteines in this sequence: **6**
**Disulfide Connectivity prediction**
**Step 1:** Running PSI-BLAST with input sequence; click here to see the output
**Step 2:** Predicting secondary structure using PSIPRED; click here to see the output
**Step 3:** Disulfide Oxidation State Prediction; click here to see the results
**Step 4:** Disulfide Bonds Prediction using a trained Neural Network

**Step 5:** Weighted matching	
**Predicted bonds**	
26 - 74	HHLDICELPAD – ECMAKCGGAXX
35 - 57	ADSGTCFVRLH – FLYRGCGGNEN
49 - 70	FNGESCTKFLY – MTEVECMAKCG
**Predicted connectivity**	
1-6, 2-4, 3-5	

Introduction | Instructions | References | About | Help! | Contacts

© Boston College

Kunitz-7-Pverdolaga:
XTSCKGIKESCTLDRAEAKEICMMPKEIGPCRGYFHRWYFDVNTLTCVTFVYGGCRGNNNNF
EFQRDCVRTCEPLFKASGNEDPSNVSTHDVAQGNSPIDCMVTPWX

Sequence **inputSeq** Length **108** residues
Cysteines in this sequence: **9**
Disulfide Connectivity prediction
**Step 1:** Running PSI-BLAST with input sequence; click here to see the output
**Step 2:** Predicting secondary structure using PSIPRED; click here to see the output
**Step 3:** Disulfide Oxidation State Prediction; click here to see the results
**Step 4:** Disulfide Bonds Prediction using a trained Neural Network

**Step 5:** Weighted matching	
**Predicted bonds**	
11 - 101	GIKESCTLDRA - NSPIDCMVTPW
22 - 72	EAKEICMMPKE - DCVRTCEPLFK
31 - 55	KEIGPCRGYFH - FVYGGCRGNNN
47 - 68	VNTLTCVTFVY - EFQRDCVRTCE
**Predicted connectivity**	
2-9, 3-8, 4-6, 5-7	

Introduction | Instructions | References | About | Help! | Contacts

© Boston College

Kunitz-8-Pverdolaga:
DAGTCFASIPRWYFTGSKCRSFIYGGCGGNANNFDTELXMPKEMWKEMKRVTSHFTTETGTA QNPSSGYLEVFEEVRIV

Sequence **inputSeq** Length **80** residues

Cysteines in this sequence: **3**

Disulfide Connectivity prediction

Step 1: Running PSI-BLAST with input sequence; click here to see the output

Step 2: Predicting secondary structure using PSIPRED; click here to see the output

Step 3: Disulfide Oxidation State Prediction; click here to see the results

Step 4: Disulfide Bonds Prediction using a trained Neural Network

Disulfide bond scores

Cysteine sequence position	Distance	Bond	Score
5 - 19	14	XDAGTCFASIP-FTGSKCRSFIY	0.0108
5 - 27	22	XDAGTCFASIP-FIYGGCGGNAN	0.99974
19 - 27	8	FTGSKCRSFIY-FIYGGCGGNAN	0.01567

Step 5: Weighted matching

Predicted bonds

5 - 27	XDAGTCFASIP - FIYGGCGGNAN

Predicted connectivity

1-3

Introduction Instructions References About Help! Contacts

© Boston College

Kunitz-9-Pverdolaga:
QVATLHPPEAEKENLRNYVQRFPTGVVMKSSLGVCELEKNSGPCAKSYKRWYYDAISKDCLPFSYGGCLGNENRFRTKAACEETCKN

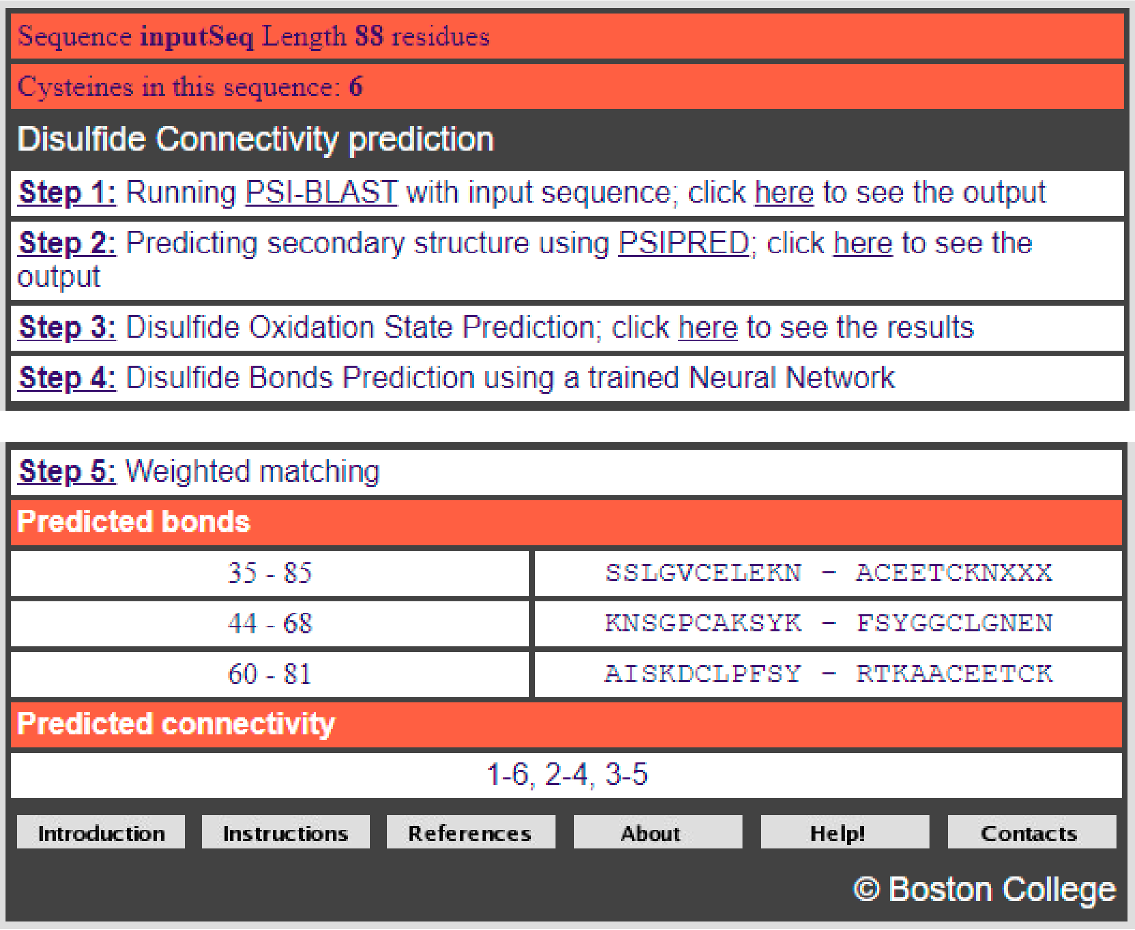
Sequence **inputSeq** Length **88** residues

Cysteines in this sequence: **6**

Disulfide Connectivity prediction

Step 1: Running PSI-BLAST with input sequence; click here to see the output

Step 2: Predicting secondary structure using PSIPRED; click here to see the output

Step 3: Disulfide Oxidation State Prediction; click here to see the results

Step 4: Disulfide Bonds Prediction using a trained Neural Network

Step 5: Weighted matching

Predicted bonds

35 - 85	SSLGVCELEKN – ACEETCKNXXX
44 - 68	KNSGPCAKSYK – FSYGGCLGNEN
60 - 81	AISKDCLPFSY – RTKAACEETCK

Predicted connectivity

1-6, 2-4, 3-5

Introduction Instructions References About Help! Contacts

© Boston College

Kunitz-10-Pverdolaga:
XLKLPPKMMKLLWVNLLLLVLATCLCSEKTDKTNNGICNQRMDSGNGNQRITHFYYDTGRQ
KCHPFPYSGRGGNKNNFSTMQECKKRMP

Sequence **inputSeq** Length **90** residues

Cysteines in this sequence: **5**

Disulfide Connectivity prediction

Step 1: Running PSI-BLAST with input sequence; click here to see the output

Step 2: Predicting secondary structure using PSIPRED; click here to see the output

Step 3: Disulfide Oxidation State Prediction; click here to see the results

Warning! The number of predicted half-*cystines* is lower than 2.

Step 4: Disulfide Bonds Prediction using a trained Neural Network

Step 5: Weighted matching

**Predicted bonds**	
26 - 38	LATCLCSEKTD – TNNGICNQRMD
63 - 84	TGRQKCHPFPY – STMQECKKRMP

Predicted connectivity

2-3, 4-5

Introduction Instructions References About Help! Contacts

© Boston College

Kunitz-11-Pverdolaga: MRKSIMGTQVQGFRSSSPGALMSRVMASTTLCFFSGKVYLQA

No disulfide bonds are possible for this sequence, the prediction will not be attempted

Clote's structural bioinformatics Lab - Author's homepage

Kunitz-12-Pverdolaga:
MLLRNDVSRRRIMIKVSRQRFSLLTFDLEDFCTAKDENNTDCQTLPAGDPTCYYDNCPMFYYD
TSSSTCMNVEYGGCGGKANRFWTEKECTDQCKNKEDDVANGAASEYSGIVSWLKVKYSPFL

Sequence **inputSeq** Length **124** residues
Cysteines in this sequence: **8**
Disulfide Connectivity prediction
**Step 1:** Running PSI-BLAST with input sequence; click here to see the output
**Step 2:** Predicting secondary structure using PSIPRED; click here to see the output
**Step 3:** Disulfide Oxidation State Prediction; click here to see the results
**Step 4:** Disulfide Bonds Prediction using a trained Neural Network

Step 5: Weighted matching

**Predicted bonds**	
32 - 94	DLEDFCTAKDE – ECTDQCKNKED
42 - 69	ENNTDCQTLPA – TSSSTCMNVEY
52 - 77	AGDPTCYYDNC – VEYGGCGGKAN
57 - 90	CYYDNCPMFYY – WTEKECTDQCK

Predicted connectivity

1-8, 2-5, 3-6, 4-7

Introduction | Instructions | References | About | Help! | Contacts

© Boston College

Kunitz-13-Pverdolaga:
MGIARIFSVVSLFSVFLALTFPPLFSADHHEGTDICYLPPERGVCKAYSEQWHFNGRRCAKFVF
GGCGGNANRFPTKDECIRRCRKA

Sequence **inputSeq** Length **87** residues

Cysteines in this sequence: **6**

Disulfide Connectivity prediction

Step 1: Running PSI-BLAST with input sequence; click here to see the output

Step 2: Predicting secondary structure using PSIPRED; click here to see the output

Step 3: Disulfide Oxidation State Prediction; click here to see the results

Step 4: Disulfide Bonds Prediction using a trained Neural Network

Step 5: Weighted matching

Predicted bonds

36 - 84	EGTDICYLPPE - ECIRRCRKAXX
45 - 67	PERGVCKAYSE - FVFGGCGGNAN
59 - 80	FNGRRCAKFVF - PTKDECIRRCR

Predicted connectivity

1-6, 2-4, 3-5

Introduction | Instructions | References | About | Help! | Contacts

© Boston College

Amino acid sequences of Hyaluronidases translated from *Pamphobeteus verdolaga*. Yellow highlighted residues indicate the cysteine position on hyaluronidase-like sequence (Cys17, Cys176, Cys183, Cys196, Cys218, Cys307, Cys332, Cys337, Cys343, Cys372, Cys374 and Cys383).

Hyaluronidase-1-Pverdolaga:
PRMYQSKLLQGLFPNSKDLPDSVLLLDESEGAVKVIGHSRLSRVLEDNRGCWIASVIISKEKRG
QGLGKFLMMKTEEYAKVLGLTTAYLNTRDKQGFYEHLGYSYCNPVSPHKGSFSMNGVGHLS
NFHRQVLRRCEEGETNHSGSPVGDPKIAKSSSVSSAKTSTPPLPPPPPPPPSSSNVKTDCFTSATG
HNWMKKYL

Sequence **inputSeq** Length **199** residues

Cysteines in this sequence: **4**

Disulfide Connectivity prediction

Step 1: Running PSI-BLAST with input sequence; click here to see the output

Step 2: Predicting secondary structure using PSIPRED; click here to see the output

Step 3: Disulfide Oxidation State Prediction; click here to see the results

Warning! The number of predicted half-*cystines* is lower than 2.

Step 4: Disulfide Bonds Prediction using a trained Neural Network

Step 5: Weighted matching

Predicted bonds

51 - 135	EDNRGCWIASV - QVLRRCEEGET
106 - 185	LGYSYCNPVSP - NVKTDCFTSAT

Predicted connectivity

1-3, 2-4

Introduction Instructions References About Help! Contacts

© Boston College

Hyaluronidase-2-Pverdolaga:
MSVTLFLLLLLPCYTRQAEDPTVFTVRWNVPTIQCRKTYGMDFVPLLKSYGILVNSGDEFKGEV
NTIFYESQLGLYPHLDQSGQRVNGGIPQLGDLPEHLKNAREDINKAIPDINFNGLGIIDWESWR
PVWNFNWGALKKYQDESFQEALKQHPGWTNDSLWQLAQQEWETSAKNFMLETLRLAQTM
RPNSLWCYYLFPDCYNYNGQTPREFRCPSIVVTGNNQLSWLWHESKAVCPSLYVADGYLQKY
TFEQRTWYVDGRLKEALRVAPNSQLYPYIGYGYGVTPGAMVPEDDFWRILAQVASAGSSGTVI
WGASATLRSKDNCQLLQKYVKDILGPSVTIVKENAERCAKTMCNGKGRCTWLNDPNVIAWR
VYLDRNKHPFQRSEITCHCVEGYSGRYCDVQRRVINQTKLRVSFKLSDLYTYLRRLLDN

Sequence **inputSeq** Length **432** residues

Cysteines in this sequence: **13**

Disulfide Connectivity prediction

Step 1: Running PSI-BLAST with input sequence; click here to see the output

Step 2: Predicting secondary structure using PSIPRED; click here to see the output

Step 3: Disulfide Oxidation State Prediction; click here to see the results

Step 4: Disulfide Bonds Prediction using a trained Neural Network

Step 5: Weighted matching

Predicted bonds

13 - 392	LLLLPCYTRQA - EITCHCVEGYS
35 - 236	VPTIQCRKTYG - ESKAVCPSLYV
194 - 201	PNSLWCYYLFP - YLFPDCYNYNG
214 - 325	PREFRCPSIVV - RSKDNCQLLQK
350 - 361	ENAERCAKTMC - NGKGRCTWLND
355 - 390	CAKTMCNGKGR - RSEITCHCVEG

Predicted connectivity

1-12, 2-6, 3-4, 5-7, 8-10, 9-11

Introduction | Instructions | References | About | Help! | Contacts

© Boston College
